# Hydrogen Atom Transfer-Driven Enantioselective Minisci Reaction of Amides

**DOI:** 10.1021/jacs.1c01556

**Published:** 2021-03-29

**Authors:** Rupert S. J. Proctor, Padon Chuentragool, Avene C. Colgan, Robert J. Phipps

**Affiliations:** Yusuf Hamied Department of Chemistry, University of Cambridge, Lensfield Road, Cambridge CB2 1EW, United Kingdom

## Abstract

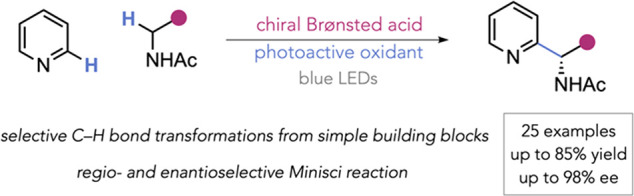

Minisci-type reactions constitute one of the most powerful methods for building up complexity around basic heteroarenes. The most desirable variants involve formal oxidative coupling of a C–H bond on each partner, leading back to the simplest possible starting materials. We herein disclose a method that enables such a coupling of linear amides and heteroarenes with full control of enantioselectivity at the newly formed stereocenter as well as site selectivity on both the heteroarene and the amide. This is achieved by the use of a chiral phosphoric acid catalyst in conjunction with diacetyl as a combined hydrogen atom transfer reagent and oxidant. Diacetyl is directly photoexcitable, and thus, no extraneous photocatalyst is required: an added feature that contributes to the simplicity and practicality of the protocol.

Methods for the selective conversion of C–H bonds to new functional groups are in great demand due to the efficiency and diversity that they can impart, with vast progress being made through a variety of mechanisms. Within this broad area is a subclass of reactions that couple two components together *via* functionalization of a C–H bond on each partner. In such a coupling, the complexity increase arising in a single chemical step is enormous, as the lack of a requirement for existing functional groups on each partner means they are often trivial to access. Often referred to as oxidative coupling or cross-dehydrogenative coupling reactions, these processes encompass a great breadth of mechanistic diversity, the common feature being that the formal loss of hydrogen necessitates an oxidant.^1−12^ In many cases a stereocenter is created, and exerting control over its formation represents an appealing way to increase the value added by the methodology even further. However, it is difficult enough to engineer a single reaction system to permit the coupling of two C–H bonds, to superimpose a catalytic strategy for the control of enantioselectivity represents a daunting challenge.^13−17^ A significant proportion of enantioselective variants developed thus far is based on *in situ* generated iminium ions and carbocations being trapped with nucleophiles.^18−27^ Though further types have been reported, for example, processes proceeding via oxo-carbenium ions,^28,29^ metal-catalyzed cross-coupling of (hetero)arenes to form biaryls,^30−34^ and others,^35,36^ there is still a paucity of enantioselective methods considering the synthetic attractiveness afforded by the formal coupling of two C–H bonds.

Minisci-type reactions have become one of the leading methods for heteroarene functionalization.^37−42^ While there are now myriad protocols for radical generation in Minisci-type reactions, those based on hydrogen atom transfer (HAT) represent a particular type of cross-dehydrogenative coupling reaction. Numerous examples have been reported, typically involving HAT from the α-position of ethers but also increasingly from simpler alkanes.^41,43−48^ Recently, we developed a strategy for controlling enantioselectivity when a prochiral radical bearing an acetamido group participates in a Minisci-type reaction.^49^ The hypothesis was that, following protonative activation of the heteroarene by a chiral phosphoric acid (CPA), the chiral conjugate anion remains associated after radical addition, enabling enantiocontrol to be exerted in deprotonation of the resulting radical cation intermediate (Figure 1a). Computational modeling supported this hypothesis and revealed an unexpected internal mode of deprotonation (as shown).^50^ In our original work, we utilized redox-active esters (RAEs) derived from *N*-acetyl amino acids as radical precursors, the reduction of which formed *N*-acyl, α-amino radicals (Figure 1b, upper box).^51,52^ In addition to providing excellent control of enantioselectivity, the CPA catalyst was able to impart high regioselectivity for the C2 position of the heteroarenes, whereas typically mixtures of regioisomers would be expected in many cases.^53,54^ Further related developments have since been made by ourselves and others.^55−57^ However, there are significant drawbacks to our original protocol. While the RAEs constituted very effective radical precursors, they required synthesis from the corresponding *N*-acetyl amino acid, which was often low-yielding. More problematically, the number of readily commercially available amino acids is largely restricted to those that are naturally occurring and a protracted synthesis is required for most others. A further practical limitation was that some amino acid-derived RAEs exhibited poor stability upon purification and/or storage. In considering these drawbacks, we speculated whether it may be possible to combine HAT-driven radical formation with our CPA-catalyzed strategy for the control of selectivity. Such an approach would obviate the need for prefunctionalization of the radical precursor, constituting a formal coupling of two C–H bonds with control over both enantioselectivity and regioselectivity in the product (Figure 1b, lower box).

**Figure 1:**
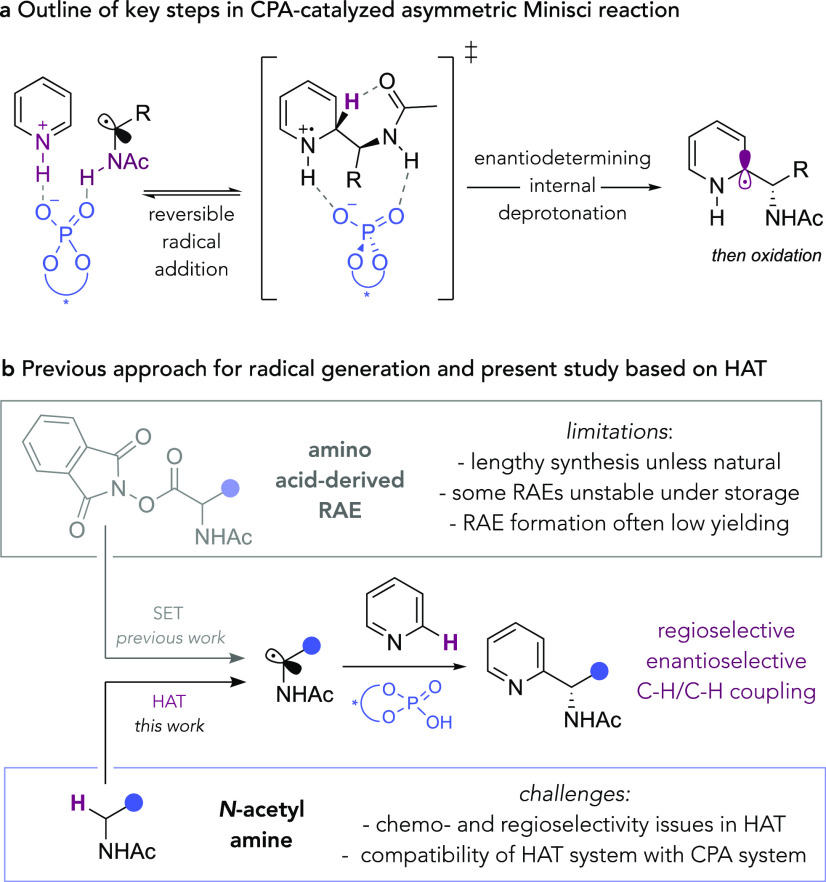
Background to the CPA-catalyzed Minisci reaction and the aims of this study.

Several major obstacles must be overcome in order to realize this goal. While Minisci-type reactions involving HAT from ethers are numerous and typically facile, those involving HAT from amides to generate α-amino radicals are far fewer.^58−68^ Furthermore, it is notable that most of these involve amides derived from secondary amines, which bear no N–H on the resulting radical. These are unlikely to be conducive to selectivity in our protocol, as experimental and computational studies have firmly established that the N–H functionality plays a crucial role in interacting with the catalyst (Figure 1a).^50^ Only a handful of HAT-driven Minisci reaction reports contain examples of successful HAT from the α-position of *N*-acylated primary amines, causing concern that this may be problematic.^61,62^ We anticipated that a major challenge would be to achieve this in the presence of other abstractable hydrogen atoms. Encouragingly, a number of recent studies involving nickel catalysis have achieved selective HAT from the α-position of secondary amides in which the bromine radical is implicated as HAT reagent.^69,70^ Other important studies have demonstrated site-selective HAT from the α-position of secondary carbamates.^71,72^ This gave us optimism that a selective HAT reagent may be identifiable. A potential complication in our case is that a stoichiometric oxidant must be compatible with the HAT system; ideally, a single reagent would perform both functions.

We commenced our studies with lepidine (**1**) and *N*-acetylphenethylamine (**2**). The latter is a challenging radical precursor due to the possibility for HAT to occur from the benzylic position. However, as part of our optimization, we particularly sought to identify a HAT protocol that would be selective for the position α to amines even in the presence of other weak C–H bonds.^72^ We initially examined peroxides as a combined HAT reagent source and oxidant. In the presence of a photocatalyst and irradiation with blue LEDs, we envisaged that photosensitized peroxide cleavage may occur (Table 1).^73,74^ Although 1,4-dioxane had been the optimal solvent previously, its liability to undergo HAT prompted us to switch to ^*t*^BuOAc. Encouraging initial results were obtained using di-*tert*-butyl peroxide (DTBP), Ir[dF(CF_3_)ppy]_2_(dtbpy)PF_6_ (**Ir-cat**) as photocatalyst, and (*R*)-**TRIP** as the CPA (entry 1). While the product yield was low (15%), we were pleased to observe that the enantiomeric excess was excellent (96%), suggesting that the crucial parts of the CPA cycle were not being disrupted by the HAT process. We next evaluated the organic dye photocatalyst **4CzIPN** and obtained comparable results (entry 2). Whereas dicumyl peroxide (DCP) gave similar results to DTBP (entry 3), dibenzoyl peroxide gave no discernable product (entry 4). Although the enantiomeric excesses were excellent, we were unable to increase the chemical yields to levels >20% using peroxides as oxidants, despite extensive efforts. In most cases, mass balance was poor, and we concluded that the peroxide was inducing multiple decomposition pathways, possibly *via* nonselective HAT or overoxidation of various intermediates. In attempting to overcome this, our attention was drawn to an interesting recent study from Li and co-workers, in which HAT-induced Minisci reactions from ethers are carried out simply by visible light irradiation of diacetyl ((CH_3_CO)_2_) in the presence of acid.^75^ Diacetyl is a cheap, low molecular weight oxidant that absorbs in the 380–460 nm region, and Li and co-workers’ report demonstrated that it is competent at performing HAT on certain ethers possessing easily cleavable α-C–H bonds. A particularly attractive feature is that no added photocatalyst is required due to diacetyl’s ability to be directly excited using visible light. Replacing the peroxide and photocatalyst with 25 equiv of diacetyl, according to Li and co-workers’ protocol, we were very happy to observe that a yield of 77% could be obtained with little loss of enantioselectivity (entry 5). We next found that the equivalents of diacetyl could be reduced to ten (entry 6) and in this case even five (entry 7); although for the scope exploration, we preferred to use 10 equivalents, as for some less reactive substrates this was found to be superior. The use of an equimolar amount of benzil in place of an excess of diacetyl was not viable (see the Supporting Information).^75^ The reduction of the equivalents of amide to five led to an unacceptable drop in yield (entry 8) as did the use of EtOAc as solvent (entry 9). The performance of the optimal reaction in the dark led to no product formation, suggesting that photoexcitation of diacetyl is crucial (entry 10).

**Table 1:**
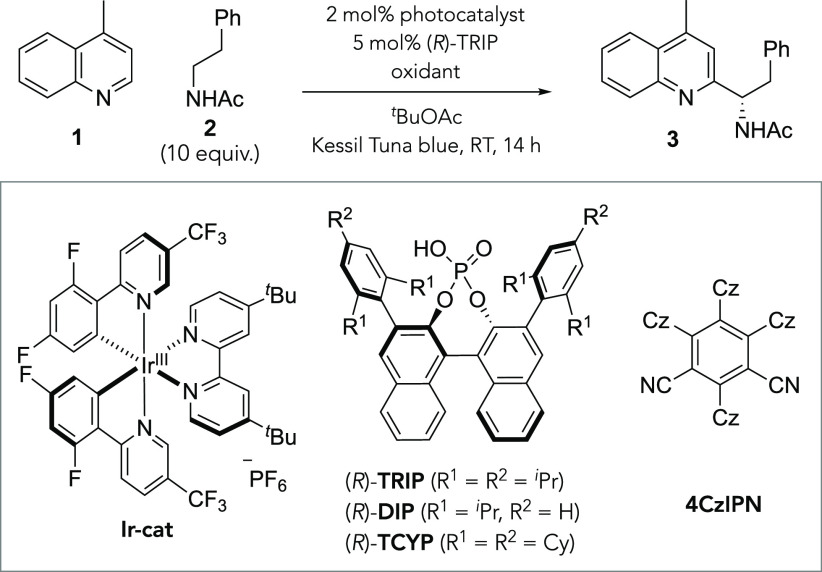
Optimization of the HAT-Driven Enantioselective Minisci Reaction^a^

entry	photocatalyst	oxidant/equiv.	solvent	yield/%	ee/%
1	Ir-cat	(^*t*^BuO)_2_ (3)	^*t*^BuOAc	15	96
2	4CzIPN	(^*t*^BuO)_2_ (3)	^*t*^BuOAc	13	96
3	4CzIPN	DCP (3)	^*t*^BuOAc	7	92
4	4CzIPN	(BzO)_2_ (3)	^*t*^BuOAc	<5	nd
5	none	(CH_3_CO)_2_ (25)	^*t*^BuOAc	77	93
6	none	(CH_3_CO)_2_ (10)	^*t*^BuOAc	85	93
7	none	(CH_3_CO)_2_ (5)	^*t*^BuOAc	85	93
8^b^	none	(CH_3_CO)_2_ (10)	^*t*^BuOAc	66	94
9	none	(CH_3_CO)_2_ (10)	EtOAc	65	93
10^c^	none	(CH_3_CO)_2_ (10)	^*t*^BuOAc	0	

a Yield determined by ^1^H NMR with reference to 1,3,5-trimethoxybenzene. ee determined by SFC.

b 5 equiv of amide.

c Reaction run in the dark.

We first evaluated the substrate scope of *N*-acetylated primary amines (Scheme 1). As well as simple *N*-acetylphenethylamine (**3**), a number of other phenethylamine-derived amides were effective. Fluoro- (**4**) and trifluoromethyl (**5**) substituents could be smoothly incorporated into the arene *para* position, although a methoxy group at this position gave a complex mixture, possibly a result of competing HAT at the benzylic position (**6**). A bromo substituent at the *meta* position was accommodated with no side reactions occurring at the bromine (**7**), and a 3,4-dichloro substitution pattern was equally well tolerated (**8**). It is important to note that the synthesis of RAE precursors for these products *via* the amino acid would be lengthy, whereas the acetylamine can be simply prepared by acetylation of the phenethylamine or in two easy steps from the corresponding nitrile. We found that homologated amides with the phenyl ring one (**9**) and two (**10**) methylene units further away than in phenethylamine still function as effective radical precursors and the presence of these more distant benzylic hydrogen atoms was not problematic. The removal of the aromatic ring was tolerated: simple *N*-acetylethylamine performed very well, giving both good yield and ee (**11**), and a related longer alkyl chain amide was also effective (**12**). We were pleased to discover that, under our optimized conditions, HAT could be carried out selectively adjacent to the amide even in the presence of a weak tertiary alkyl C–H bond (**13**) with the product formed in excellent enantiomeric excess. That said, we did find that exchanging the isopropyl group for a cyclohexyl group resulted in a complex mixture, suggesting that excessive HAT from the cycloalkane portion may have been occurring in this case (**14**). A remote ester could be smoothly incorporated into the amide precursor (**15**) and an enantiopure lysine-derived amide, featuring two differentially protected amines, gave the product with a high diastereomeric ratio (**16**). For the amides with longer chain lengths, the CPA (*R*)-**DIP**, in which the *i*Pr groups at the 4 and 4′ positions are removed, was found to provide optimal enantioselectivity (**9**, **10**, **12**, **15**, **16**). The remaining mass balance of the heteroarene was mostly accounted for by the starting material in all cases, and any moderate yields are not due to the formation of isomeric products. We investigated whether the addition of a photocatalyst could improve a moderate yielding example (**10**), but this was found not to be effective. Additionally, we have successfully carried out the reaction to give **3** on a 1 mmol scale with no detriment to yield or ee (see the Supporting Information for details).

**Scheme 1:**
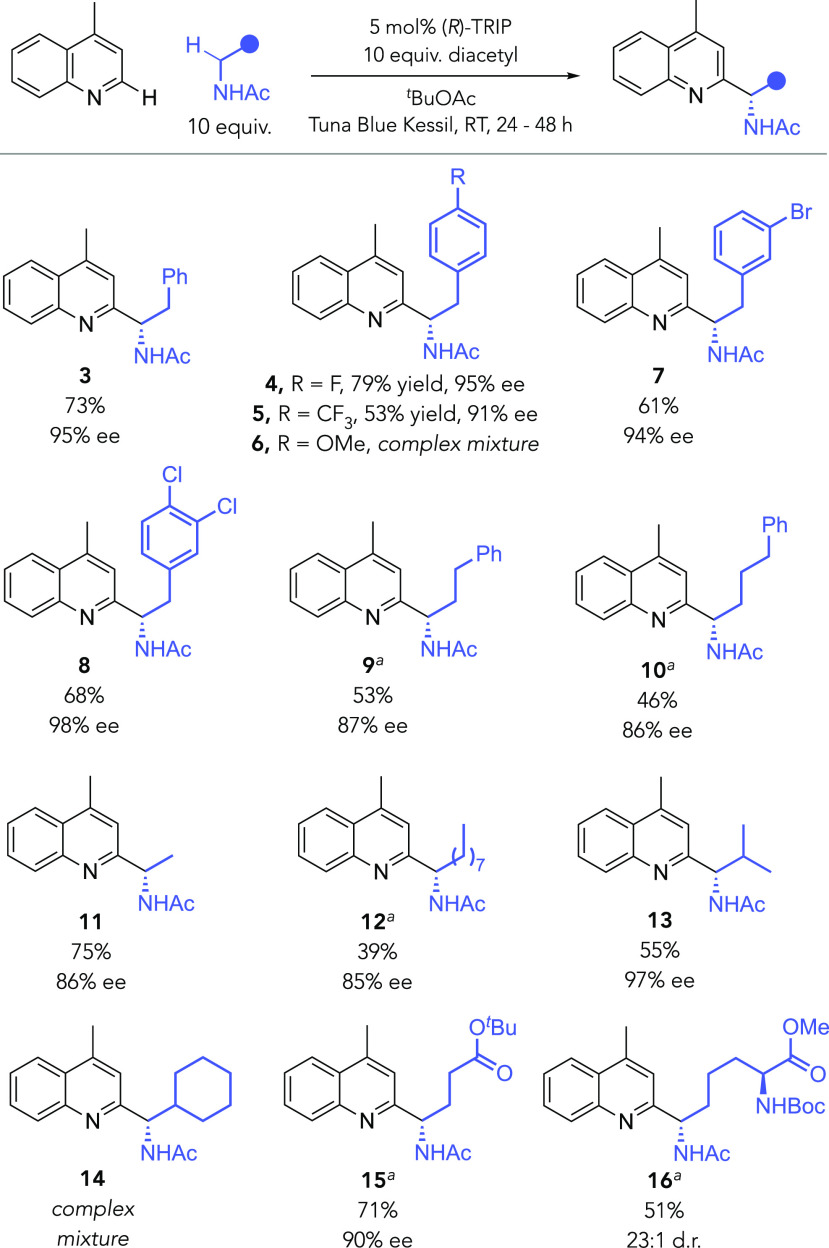
Scope of *N*-Acetylated Primary Amines Using (*R*)-DIP as catalyst.

We next examined the scope of the heteroarene reaction component (Scheme 2). Using *N*-acetylphenethylamine, we evaluated a number of quinolines. When simple quinoline, bearing no substituent at the 4-position, was used as a substrate, >20:1 regioselectivity was obtained for the reaction at the 2-position (**17**). This result is in line with our original study and demonstrates that the extremely high levels of regiocontrol imparted by the CPA are maintained in this new HAT-driven protocol. Various functionality is tolerated at the 6-position of the quinoline including methoxy (**18**) and chloro (**19**) as well as a methyl at the 3-position (**20**) with no adverse effect on regioselectivity or enantioselectivity. Phenanthridine (**21**) and a 4-aryloxy quinoline (**22**) were also effective. A range of pyridines was explored in addition, with an electron-withdrawing substituent required in order to obtain reactivity. Nicotinic acid esters reacted well with methyl groups at the 2-, 4-, and 5-positions tolerated (**23**–**26**). Versatile ketone (**27**) and nitrile (**28**) functionalities were also incorporated smoothly. For the pyridines, the bulkier CPA (*R*)-**TCYP** was found to give the highest enantioselectivity.^76^

**Scheme 2:**
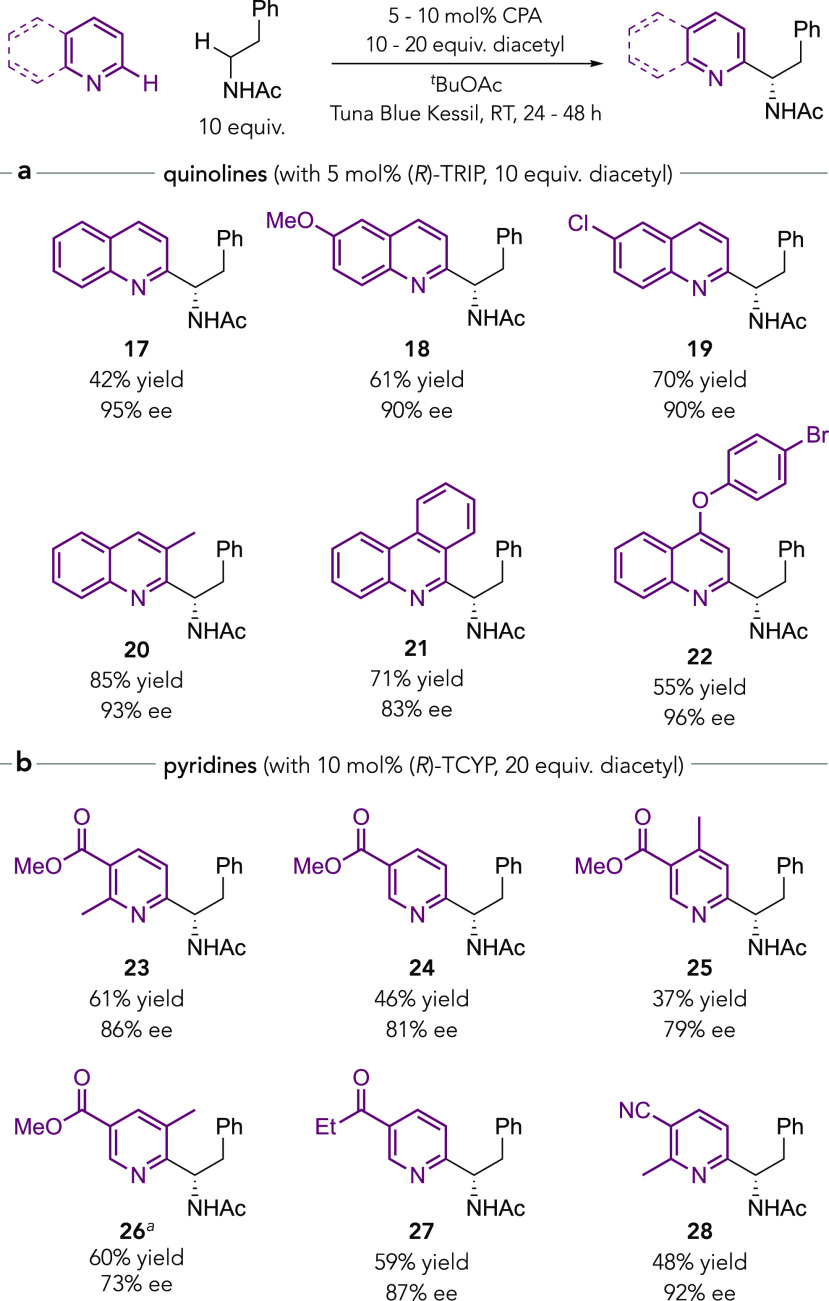
Scope of Quinolines and Pyridines With (*R*)-TRIP.

In a prior study, we found diazines to also be effective substrates for the asymmetric Minisci reaction using RAEs. However, they typically exhibited rather lower reactivity than quinolines and pyridines.^56^ Nevertheless, we were encouraged to find that 2-methyl-4-phenylpyrimidine successfully underwent the Minisci reaction with excellent enantioselectivity using the HAT-driven protocol (Scheme 3a). While the yield was relatively low under the present conditions, we are optimistic that future refinements may be able to improve on this and expand the scope of the process further. We have also demonstrated that the *N*-acetyl group of a representative product can be readily deprotected under acidic conditions without the loss of stereochemical integrity (Scheme 3b).

**Scheme 3:**
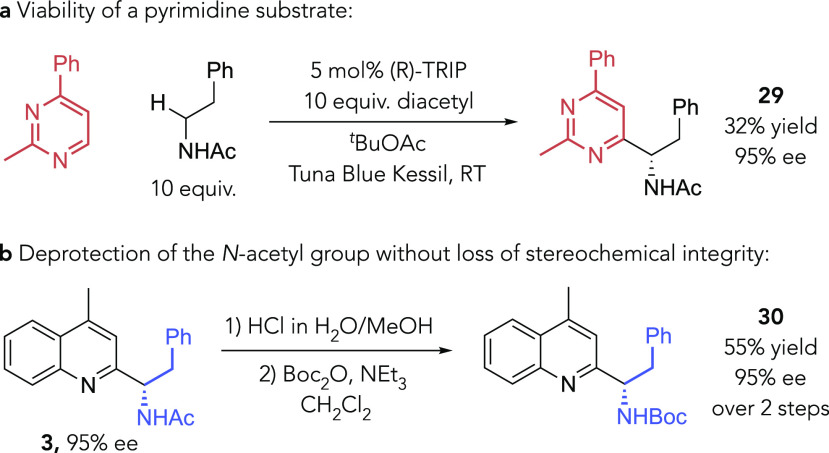
Miscellaneous Experiments

Mechanistically, we anticipate that the photoexcited diacetyl is operating in a similar manner to that proposed by Li and co-workers.^75^ Once photoexcited, the diacetyl initiates HAT from the *N*-acetyl amine (**31**) (Scheme 4). The α-amino radical produced (**32**) then takes part in CPA-catalyzed radical addition, which is reversible, as determined previously.^49,50^ Computational studies demonstrated that deprotonation of the resulting radical cation **33** is the stereodetermining step and occurs through an internal deprotonation mode effected by the amide carbonyl.^50^ This proton is rapidly transferred to a new molecule of starting material (not shown) so that the CPA cycle can continue. In accordance with Li and coworkers’ mechanistic evidence, we suspect that the resultant neutral radical **34** is then oxidized by ketyl radical **35** with an accompanied proton transfer, giving the Minisci product together with acetoin as the byproduct.

**Scheme 4:**
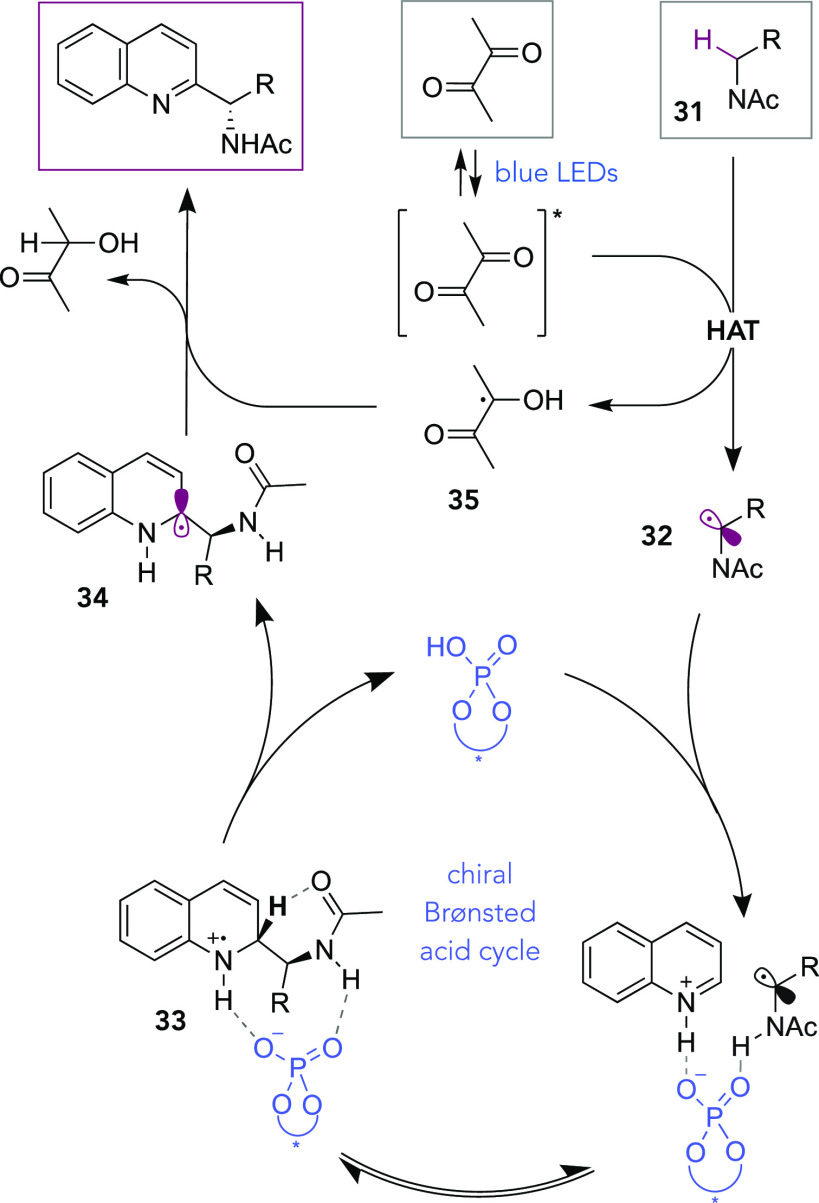
Plausible Mechanistic Scenario

In summary, we have realized an enantioselective Minisci reaction that proceeds through the formal coupling of two C–H bonds via a successful combination of HAT-driven radical generation and a CPA-catalyzed Minisci reaction. The catalyst controlled both regioselectivity and enantioselectivity. Crucial to the success was the identification of diacetyl as a mild, chemoselective reagent for the generation of α-aminoalkyl radicals, which precluded the need for an added photocatalyst. This protocol builds up a remarkable amount of complexity in a single chemical step using very simple reaction conditions and should be directly relevant to the synthesis of small molecules of medicinal interest.

## Supporting Information Available

Experimental procedures and spectral data (PDF)
