# Supplementary material for: Hydrogen Atom Transfer-Driven Enantioselective Minisci Reaction of Amides
Source: J Am Chem Soc. 2021 Mar 29;143(13):4928–34. doi: 10.1021/jacs.1c01556 (PMC8033566; doi:10.1021/jacs.1c01556)
Supplement: Supplementary file 1 — ja1c01556_si_001.pdf [file ja1c01556_si_001.pdf]

Hydrogen Atom Transfer-Driven Enantioselective Minisci Reaction of Amides

Rupert S. J. Proctor, Padon Chuentragool, Avene C. Colgan and Robert J. Phipps*

Yusuf Hamied Department of Chemistry, University of Cambridge, Lensfield Road, Cambridge, CB2 1EW, United Kingdom.

*Correspondence to: rjp71@cam.ac.uk

Table of Contents

General Information: 3

General Procedures: 4

General Procedure A for HAT-driven Enantioselective Minisci reaction 4

General Workup Procedure A 4

General Workup Procedure B 4

Experiments to probe addition of a photocatalyst 6

Experiments to probe replacement of diacetyl with benzil 7

Synthesis of Amides 8

Synthesis of N-(4-(trifluoromethyl)phenethyl)acetamide 8

Synthesis of methyl *N*-acetyl-*N*-(tert-butoxycarbonyl)-L-lysinate 9

Synthesis of Products 10

1 mmol scale: (S)-N-(1-(4-methylquinolin-2-yl)-2-henylethyl)acetamide (3) 27

Deprotection of acetyl protecting group without loss of stereochemical integrity 28

Spectra of starting materials 29

Spectra of products 32

HPLC and SFC Traces 84

References 111

General Information:

NMR spectra: ^{1}H NMR spectra were recorded on a 400 MHz Bruker Avance III spectrometer or 400 MHz Bruker Neo Prodigy Cryoprobe. Chemical shifts are reported in parts per million (ppm) and the spectra are calibrated to the resonance resulting from incomplete deuteration of the solvent ($CDCl_3$: 7.26 ppm, $(CD_3)_2CO$: 2.05 ppm). ^{13}C NMR spectra were recorded with the same spectrometer with complete proton decoupling. Chemical shifts are reported in ppm with the solvent resonance as the internal standard ($^{13}CDCl_3$: 77.16 ppm, t; $(CD_3)_2CO$: 2.05). Data are reported as follows: chemical shift δ/ppm, integration (^{1}H only), multiplicity (s = singlet, d = doublet, t = triplet, q = quartet, oct = octet, br = broad, m = multiplet) or combinations thereof; ^{13}C signals are singlets unless otherwise stated), coupling constants *J* in Hz, assignment. ^{1}H-COSY, DEPT-135, HMQC, HMBC and NOESY were used where appropriate to facilitate structural determination of regioisomers. ^{19}F NMR spectra were recorded on a 400 MHz Bruker Avance III HD Spectrometer.

High Resolution Mass Spectrometry (HRMS): Some were recorded on a Waters Micromass LCT Premier spectrometer using a positive electrospray ionization (ESI+). Measured values are reported to 4 decimal places are within ±5 ppm of the calculated value. The calculated values are based on the most abundant isotope.

Chromatography: Analytical thin layer chromatography was performed using precoated Merck glass backed silica gel plates (Silicagel 60 F254). Visualisation was by ultraviolet fluorescence (λ = 254 nm) and/or staining with Dragendorff's reagent or potassium permanganate ($KMnO_4$). Flash column chromatography was performed using silica gel 60 (0.040-0.063 µm) from Material Harvest.

Optical rotations were measured in $CHCl_3$ on a Perkin Elmer 343 Polarimeter using a sodium lamp (λ 589 nm, D-line). [α]. D values are reported at a given temperature (° C) in degrees cm^2 g^{-1} with concentration in mg mL^{-1}.

Chiral HPLC analysis was performed either: On a Shimadzu XR-LC apparatus with Chiralpak (IC) in a mixed solvent system of *n*-hexane and iso-propanol *or* a Waters Acquity UPC^2 with Chiralpak (IG) or YMC CHIRAL ART (SC) columns in a mixed solvent system of supercritical carbon dioxide and methanol.

Reagents, unless otherwise stated, were used as supplied from commercial sources without further purification. Tert-butyl acetate was purchased from Fluorochem and sparged with argon for 30 mins before use. CH_2Cl_2 and THF were purified by distillation on site under inert atmosphere via the following processes: THF was pre-dried over sodium wire then distilled from calcium hydride and lithium aluminium hydride. CH_2Cl_2 and *n*-hexane were distilled from calcium hydride. The syntheses of TRIP,[1] (*R*)-TCYP,[2] and DIP[3,4] are previously described. $[Ir(dF(CF_3)ppy)_2(dtbpy)]PF_6$ was prepared as described in the literature.[5]

General Procedures:

General Procedure A for HAT-driven Enantioselective Minisci reaction

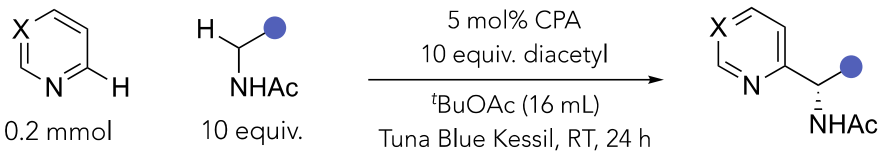

Sequentially, *N*-heteroarene (0.20 mmol, 1.0 equiv.), amide (2.0 mmol, 10 equiv.) and chiral phosphoric acid (CPA, 0.010 mmol, 5 mol%) were added to a 6-dram vial containing a stirrer bar. The vial was sealed with a septum, evacuated and refilled with argon three times. Anhydrous, freshly argon-sparged diacetyl (10 equiv., 174 μL unless otherwise specified) and *tert*-butyl acetate (16 mL) were then added *via* syringe. The reaction mixture was stirred under irradiation with Kessil Tuna blue lamp (100% intensity, 100% Ocean Blue colour) at a distance of 2 cm for 24 hours. The apparatus was maintained at approximately room temperature by use of a desk fan close to the vials. The reaction was quenched with a few drops of triethylamine and the solvent was removed *in vacuo* and the crude residue was purified as indicated.

General Workup Procedure A

The crude residue was dissolved in 10% EtOAc in diethyl ether (5 mL) and extracted with 1 M HCl (5 mL). The aqueous phase was washed with 10% EtOAc in diethyl ether (2 × 5 mL). The combined aqueous phases were basified to pH 8 and extracted with EtOAc (3 x 5 mL). The combined organic phases were dried over $MgSO_4$ and concentrated *in vacuo*.

General Workup Procedure B

The crude residue was dissolved in diethyl ether (5 mL) and extracted with 1 M HCl (5 mL). The aqueous phase was washed with diethyl ether (2 × 5 mL). The combined aqueous phases were basified to pH 8 and extracted with diethyl ether (4 x 5 mL). The combined organic phases were dried over $MgSO_4$ and concentrated *in vacuo*.

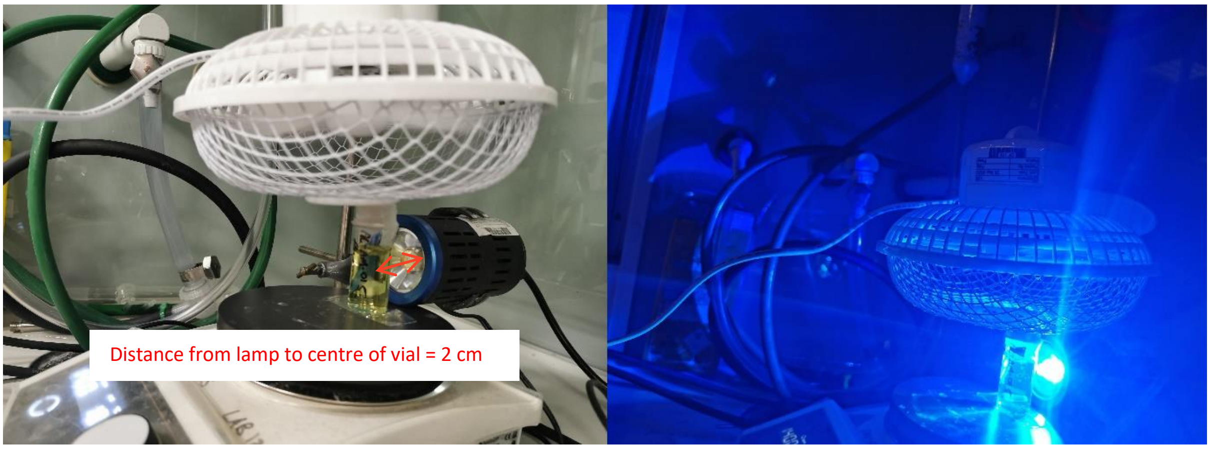

Figure 1: Photo of typical reaction set-up on 0.2 mmol scale

Experiments to probe addition of a photocatalyst

For the moderately yielding example **10** we explored whether addition of a photocatalyst might boost the yield:

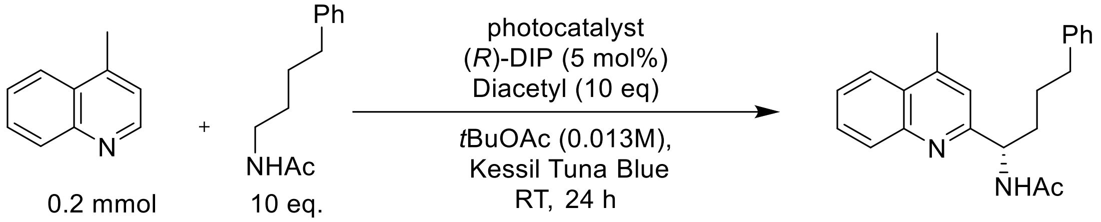

entry	photocatalyst	NMR yield / %
1	none	46*
2	$Ir[dF(CF_3)ppy]_2(dtbpy)PF_6$	42
3	4CzIPN	33

*isolated yield. NMR yield determined by ^{1}H NMR with reference to 1,3,5-trimethoxybenzene

Experiments to probe replacement of diacetyl with benzil

We investigated whether the excess of diacetyl could be replaced with an equimolar amount of benzil:

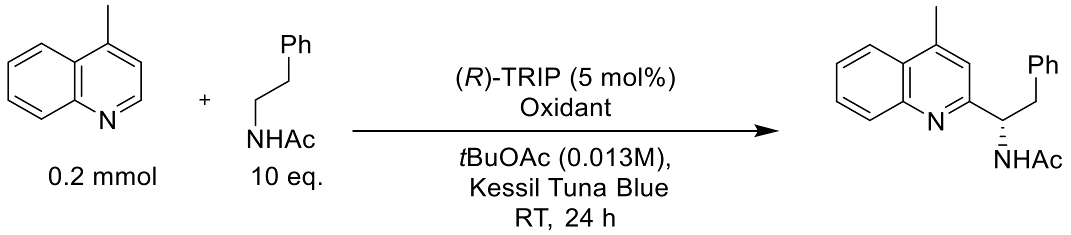

entry	Oxidant (equiv.)	NMR yield / %
1	Diacetyl (10)	73*
2	Benzil (1)	10
3	Benzil (5)	37
4	Benzil (10)	41

*isolated yield. NMR yield determined by ^{1}H NMR with reference to 1,3,5-trimethoxybenzene

Synthesis of Amides

The amide starting materials were generally prepared by acetylation of the parent amines using standard protocols. All compounds have been previously reported in the literature. In some cases, substituted phenethylamines were conveniently prepared from the corresponding benzyl nitriles using the representative procedure shown for 4-trifluoromethylbenzyl cyanide.

Synthesis of N-(4-(trifluoromethyl)phenethyl)acetamide

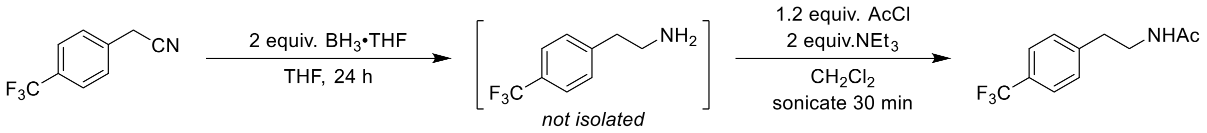

To a solution of 4-trifluoromethylbenzyl cyanide (20.0 g, 20 mmol) in THF (25 mL) was added 1M BH_3·THF (40 mL, 40 mmol) dropwise at 0 ºC over 30 min and then stirred at room temperature for 24 h. The reaction mixture was quenched with 3 M HCl (20 mL) at 0 ºC and then neutralised with 10% NaOH (30 mL). The aqueous layer was then neutralized by 15% NaOH and extracted with EA (75mL × 2). The combined organic layer was dried over Mg_2SO_4 and concentrated under reduced pressure to afford the crude amine.

The crude amine residue was dissolved in CH_2Cl_2 (50 mL) and triethylamine (5.6 mL, 40 mmol) was added. The reaction mixture was cooled to 0 ºC in an ice bath and acetyl chloride (1.7 mL, 24 mmol) was added dropwise with stirring. The ice bath was removed, and the reaction mixture was agitated in an ultrasonic bath for 30 minutes. The reaction mixture was then washed with 1 M HCl (2 × 50 mL) and the organic phase was dried over Mg_2SO_4 and concentrated *in vacuo*. The crude residue was purified first on silica, eluting with 1.5% MeOH in CH_2Cl_2. The product-containing fractions[6] were collected and repurified on silica, eluting with a gradient of 60% to 100% EtOAc in hexanes to give the title compound as a white amorphous solid (1.80 g, 7.8 mmol, 39%).

^{1}H NMR (400 MHz, $CDCl_3$) δ 7.56 (t, *J* = 8.0 Hz, 2H), 7.31 (d, *J* = 8.0 Hz, 2H), 5.54 (br. s, 1H), 3.52 (q, *J* = 6.6 Hz, 2H), 2.88 (t, *J* = 7.5 Hz, 2H), 1.95 (s, 3H); **^{13}C NMR** (101 MHz, $CDCl_3$) δ 170.2, 143.2 (q, *J* = 1.4 Hz), 129.2, 129.1 (q, *J* = 32.4 Hz), 125.7 (q, *J* = 3.8 Hz), 123.0, 40.6, 35.7, 23.4. **^{19}F NMR** (377 MHz, $CDCl_3$) δ -63.4. **HRMS m/z:** $[M+H]^+$ calculated for $[C_{11}H_{13}F_3NO]^+$ 232.0944, found: 232.0945.

Synthesis of methyl *N*-acetyl-*N*-(tert-butoxycarbonyl)-L-lysinate

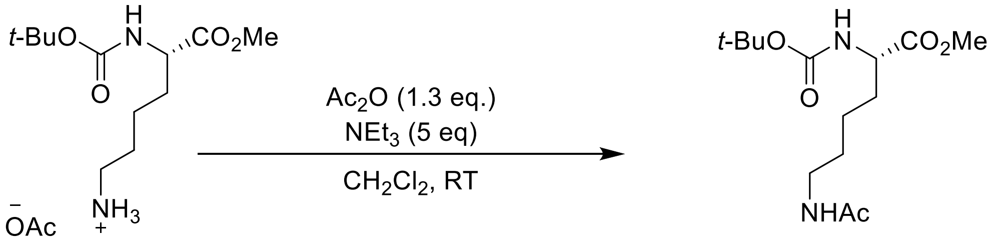

Under nitrogen atmosphere, *N*-(*tert*-butoxycarbonyl)-L-lysine methyl ester acetate salt (1.0 g, 3.1 mmol) was dissolved in anhydrous CH_2Cl_2. The mixture was cooled to 0 °C and NEt_3 (2.2 mL, 16 mmol, 5.2 equiv.) and acetic anhydride (0.38 mL, 4.0 mmol, 1.3 equiv.) were added *via* syringe. The reaction mixture was warmed to RT and stirred overnight. The crude reaction mixture was transferred to a separating funnel and the organic phase washed with H_2O (2 x 20 mL). The organic phase was dried over Na_2SO_4, filtered and concentrated *in vacuo*. The crude material was purified *via* flash chromatography, eluting with CH_2Cl_2:MeOH (100:0 to 98:2), to give the title compound as a colourless oil (631 mg, 67% yield).

^{1}H NMR (700 MHz, $CDCl_3$) δ 5.69 (br s, 1H), 5.09 (d, *J* = 6.7 Hz, 1H), 4.31 (app d, *J* = 4.5 Hz, 1H), 3.76 (s, 3H), 3.30 – 3.20 (m, 2H), 2.06 (s, 3H), 1.86 – 1.80 (m, 1H), 1.69 – 1.63 (m, 1H), 1.59 – 1.53 (m, 2H), 1.47 (s, 9H), 1.44 – 1.47 (m, 2H); **^{13}C NMR** (101 MHz, $CDCl_3$) δ 173.3, 170.6, 155.7, 80.1, 53.2, 52.5, 39.5, 32.7, 28.9, 28.4, 23.2, 22.7. **HRMS m/z:** $[M+H]^+$ calculated for $[C_{14}H_{27}N_2O_5]^+$ 303.1914, found: 303.1914. **$[\alpha]_D^{25.0}$** = +7.2 (*c* 1.0, $CHCl_3$).

Synthesis of Products

(*S*)-*N*-(1-(4-methylquinolin-2-yl)-2-phenylethyl)acetamide (3)

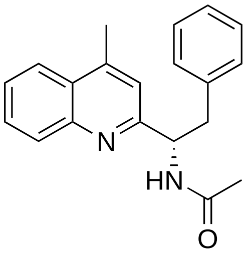

General procedure A was followed with 4-methylquinoline (28.6 mg, 0.20 mmol, 1.0 equiv.), *N*-acetylphenethylamine (326 mg, 2.0 mmol, 10 equiv.) and (*R*)-TRIP (7.5 mg, 0.01 mmol, 5 mol%). The crude material was subjected to work-up procedure A and the resultant material was purified *via* flash chromatography (eluting with 50% EtOAc in hexanes) to yield the title compound as a white amorphous solid (44.4 mg, 73%, 0.146 mmol, 95% ee).

^{1}H NMR (600 MHz, $CDCl_3$) δ 8.02 (d, *J* = 8.3 Hz, 1H), 7.96 (dd, *J* = 8.3, 0.9 Hz, 1H), 7.71-7.68 (m, 1H), 7.56-7.53 (m, *J* = 2.7 Hz, 1H), 7.25 (m, 1H, signal overlapping with solvent), 7.16-7.14 (m, 3H), 6.96-6.94 (m, 2H), 6.80 (s, 1H), 5.40-5.37 (m, 1H), 3.34 (d, *J* = 13.5, 5.3 Hz, 1H), 3.16 (q, *J* = 13.5, 8.1 Hz, 1H), 2.58 (s, 3H), 2.08 (s, 3H); **^{13}C NMR** (151 MHz, $CDCl_3$) δ 169.5, 158.9, 147.2, 144.7, 137.3, 129.8, 129.5, 129.4, 128.2, 127.5, 126.5, 126.3, 123.9, 121.6, 55.6, 42.3, 23.7, 18.8; **HPLC Analysis:** Chiralpak IC (Hexane/^{i}PrOH = 70/30, 1.0 mL min^{-1}, 30 °C) t_R = 9.0 (minor), 10.4 (major) minutes; **$[\alpha]_D^{25.0}$** = +30.7 (*c* 1.0, $CHCl_3$). Data are in accordance with the literature.[7]

(*S*)-*N*-(2-(4-fluorophenyl)-1-(4-methylquinolin-2-yl)ethyl)acetamide (4)

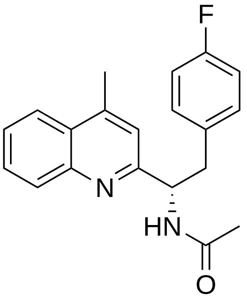

Following general procedure A with 4-methyl quinoline (28.6 mg, 0.20 mmol, 1.0 equiv.), N-(4-fluorophenethyl)acetamide (362, 2.0 mmol, 10 equiv.) and (*R*)-TRIP (7.5 mg, 0.01 mmol, 5 mol%). The crude material was subjected to work-up procedure A and the resultant material was purified *via* flash chromatography (eluting with 50% EtOAc in hexanes) to yield the title compound as a white amorphous solid (51.2 mg, 79%, 0.158 mmol, 95% ee).

^{1}H NMR (400 MHz, $CDCl_3$) δ 8.03 (d, *J* = 8.4 Hz, 1H), 7.97 (d, *J* = 8.2 Hz, 1H), 7.71 (t, *J* = 7.6 Hz, 1H), 7.56 (t, *J* = 7.5 Hz, 1H), 7.25 (d, *J* = 6.6 Hz, 1H), 6.90-6.81 (m, 5H), 5.35 (td, J = 7.6, 5.1 Hz, 1H), 3.30 (dd, *J* = 13.4, 5.4 Hz, 1H), 3.17 (dd, *J* = 13.4, 7.9 Hz, 1H), 2.61 (s, 3H), 2.08 (s, 3H); **^{13}C NMR** (101 MHz, $CDCl_3$) δ 169.6, 163.0, 160.6, 158.6, 147.1, 145.0, 133.0 (d, $J_{C\text{-}F}$ = 3.2 Hz), 131.2 (d, $J_{C\text{-}F}$ = 7.9 Hz), 129.6, 129.5, 127.6, 126.4, 124.0, 121.6, 115.0 (d, $J_{C\text{-}F}$ = 21.1 Hz), 55.5, 41.4, 23.7, 18.8; **^{19}F NMR** (377 MHz, $CDCl_3$) δ -117.7. **HRMS m/z:** $[M+H]^+$ calculated for $[C_{20}H_{20}FN_2O]^+$ 323.1554, found: 323.1557. **SFC Analysis:** Chiralpak SC (CO_2/MeOH = 85/15, 2.5 mL min^{-1}, 20 °C) t_R = 4.2 (minor), 4.8 (major) minutes. **$[\alpha]_D^{25.0}$** = +19.5 (*c* 1.0, $CHCl_3$).

(*S*)-*N*-(1-(4-methylquinolin-2-yl)-2-(4-(trifluoromethyl)phenyl)ethyl)acetamide (5)

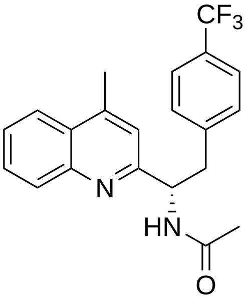

Following general procedure A with 4-methyl quinoline (28.6 mg, 0.20 mmol), N-(4-trifluoromethylphenethyl)acetamide (462 mg, 2.0 mmol, 10 equiv.) and (*R*)-TRIP (7.5 mg, 0.01 mmol, 5 mol%). The crude material was subjected to work-up procedure A and the resultant material was purified *via* flash chromatography (eluting with 50% EtOAc in hexanes) to yield the title compound as a white amorphous solid (46.4 mg, 61%, 0.122 mmol, 91% ee).

^{1}H NMR (400 MHz, $CDCl_3$) δ 8.02 (d, *J* = 8.4 Hz, 1H), 7.97 (d, *J* = 8.3 Hz, 1H), 7.71 (t, *J* = 7.6 Hz, 1H), 7.56 (t, *J* = 7.6 Hz, 1H), 7.40 (d, *J* = 8.1 Hz, 2H), 7.05 (d, *J* = 8.0 Hz, 2H), 6.83 (s, 1H), 5.41 (dt, *J* = 5.2, 7.5 Hz, 1H), 3.38 (dd, *J* = 13.2, 5.1 Hz, 1H), 3.28 (dd, *J* = 13.3, 7.8 Hz, 1H), 2.60 (s, 3H), 2.08 (s, 3H); **^{13}C NMR** (101 MHz, $CDCl_3$) δ 169.7, 158.2, 147.2, 145.2, 141.5 (q, *J* = 1.4 Hz), 130.1, 129.6, 129.5, 128.9 (q, *J* = 32.4 Hz), 127.6, 126.5, 125.7, 125.1 (q, *J* = 3.8 Hz), 124.0, 123.0, 121.4, 55.2, 41.8, 23.6, 18.8. **^{19}F NMR** (377 MHz, $CDCl_3$) δ -63.4. **HRMS m/z:** $[M+H]^+$ calculated for $[C_{21}H_{20}F_3N_2O]^+$ 323.1554, found: 323.1557. **SFC Analysis:** Chiralpak SC (CO_2/MeOH = 85/15, 2.5 mL min^{-1}, 20 °C) t_R = 2.7 (minor), 3.0 (major) minutes. **$[\alpha]_D^{25.0}$** = +24.2 (*c* 1.0, $CHCl_3$).

(*S*)-*N*-(2-(3-bromophenyl)-1-(4-methylquinolin-2-yl)ethyl)acetamide (7)

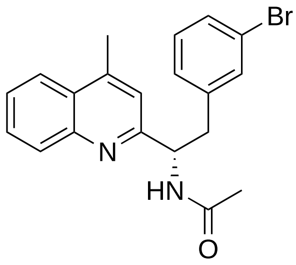

Following general procedure A with 4-methyl quinoline (28.6 mg, 0.20 mmol), N-(3-bromophenethyl)acetamide (484 mg, 2.0 mmol, 10 equiv.) and (*R*)-TRIP (7.5 mg, 0.01 mmol, 5 mol%). The crude material was subjected to work-up procedure A and the resultant material was purified *via* flash chromatography (eluting with 50% EtOAc in hexanes) to yield the title compound as a white amorphous solid (39.5 mg, 53%, 0.106 mmol, 94% ee).

^{1}H NMR (400 MHz, $CDCl_3$) δ 8.04 (d, *J* = 8.4 Hz, 1H), 7.97 (d, *J* = 8.2 Hz, 1H), 7.71 (t, *J* = 7.5 Hz, 1H), 7.56 (t, *J* = 7.6 Hz, 1H), 7.28 (t, *J* = 7.3 Hz, 1H), 7.12 (s, 1H), 7.01 (t, *J* = 7.8 Hz, 1H), 6.86 (d, *J* = 7.6 Hz, 1H), 6.82 (s, 1H), 5.35 (dt, *J* = 5.3, 7.6 Hz, 1H), 3.29 (dd, *J* = 5.2, 13.3 Hz, 1H), 3.13 (dd, *J* = 7.9, 13.3 Hz, 1H), 2.62 (s, 1H), 2.08 (s, 1H); **^{13}C NMR** (101 MHz, $CDCl_3$) δ 169.6, 158.3, 147.1, 145.1, 139.7, 132.9, 129.8, 129.7, 129.6, 129.5, 128.4, 127.6, 126.5, 124.0, 122.2, 121.5, 55.4, 41.9, 23.7, 18.8. **HRMS m/z:** $[M+H]^+$ calculated for $[C_{20}H_{20}BrN_2O]^+$ 383.0754, found: 383.0776. **SFC Analysis:** Chiralpak SC (CO_2/MeOH = 85/15, 2.5 mL min^{-1}, 20 °C) t_R = 6.4 (minor), 7.4 (major) minutes. **$[\alpha]_D^{25.0}$** = +31.4 (*c* 1.0, $CHCl_3$).

(*S*)-*N*-(2-(3,4-dichlorophenyl)-1-(4-methylquinolin-2-yl)ethyl)acetamide (8)

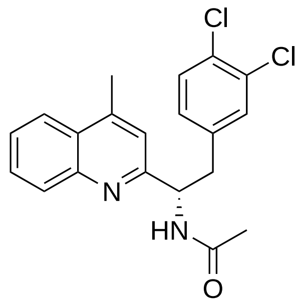

Following general procedure A with 4-methyl quinoline (28.6 mg, 0.20 mmol), N-(3,4-dichlorophenethyl)acetamide (464 mg, 2.0 mmol, 10 equiv.) and (*R*)-TRIP (7.5 mg, 0.01 mmol, 5 mol%). The crude material was subjected to work-up procedure A and the resultant material was purified via flash chromatography (eluting with 50% EtOAc in hexanes) to yield the title compound as a white amorphous solid (50.8 mg, 68%, 0.136 mmol, 98% ee).

^{1}H NMR (400 MHz, $CDCl_3$) δ 8.02 (d, *J* = 8.4 Hz, 1H), 7.97 (d, *J* = 8.0 Hz, 1H), 7.71 (t, *J* = 7.7 Hz, 1H), 7.57 (t, *J* = 7.5 Hz, 1H), 7.23 (d, *J* = 7.1 Hz, 1H), 7.19 (d, *J* = 8.2 Hz, 1H), 7.05 (d, *J* = 1.9 Hz, 1H), 6.89 (s, 1H), 6.74 (dd, *J* = 8.2, 2.0 Hz, 1H), 5.35 (td, *J* = 7.4, 5.3 Hz, 1H), 3.27-3.16 (m, 2H), 2.64 (s, 3H), 2.08 (s, 3H); **^{13}C NMR** (101 MHz, $CDCl_3$) δ 147.2, 145.3, 137.7, 132.1, 131.8, 130.6, 130.1, 129.7, 129.5, 129.2, 127.6, 126.5, 124.0, 121.3, 55.1, 41.1, 23.6, 18.9. **HRMS m/z:** $[M+H]^+$ calculated for $[C_{20}H_{19}Cl_2N_2O]^+$ 373.0869, found: 383.0877. **SFC Analysis:** Chiralpak SC (CO_2/MeOH = 85/15, 2.5 mL min^{-1}, 20 °C) t_R = 6.8 (minor), 7.8 (major) minutes. **$[\alpha]_D^{25.0}$** = +56.1 (*c* 1.0, $CHCl_3$).

(*S*)-*N*-(1-(4-methylquinolin-2-yl)-3-phenylpropyl)acetamide (9)

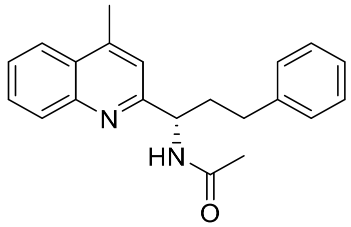

Following general procedure A with 4-methyl quinoline (28.6 mg, 0.20 mmol), *N*-(3-phenylpropyl)acetamide (354 mg, 2.0 mmol, 10 equiv.) and (*R*)-DIP (7.5 mg, 0.01 mmol, 5 mol%). The crude material was subjected to work-up procedure B and the resultant material was purified *via* flash chromatography (eluting with 50% EtOAc in hexanes) to yield the title compound as a white amorphous solid (33.8 mg, 53%, 0.106 mmol, 87% ee).

^{1}H NMR (400 MHz, $CDCl_3$) δ 8.08 (d, *J* = 8.3 Hz, 1H), 7.99 (d, *J* = 8.3 Hz, 1H), 7.72 (t, *J* = 8.2 Hz, 1H), 7.56 (ddd, *J* = 8.2 Hz, 1H), 7.27-7.21 (m, 3H), 7.16-7.12 (m, 4H), 5.29 (q, *J* = 6.6 Hz, 1H), 2.71-2.61 (m, 4H), 2.59-2.53 (m, 1H), 2.41-2.32 (m, 1H), 2.24-2.13 (m, 1H), 2.08 (s, 3H); **^{13}C NMR** (151 MHz, $CDCl_3$) δ 169.8, 159.6, 147.2, 145.4, 141.8, 129.6, 129.5, 128.5, 128.4, 127.6, 126.3, 125.9, 124.0, 121.1, 54.0, 37.8, 31.7, 23.7, 18.9; **HPLC Analysis:** Chiralpak IC (Hexane/^{i}PrOH = 70/30, 1.0 mL min^{-1}, 30 °C) t_R = 8.7 (minor), 11.5 (major) minutes; **$[\alpha]_D^{25.0}$** = -0.3 (*c* 1.0, $CHCl_3$). Data are in accordance with the literature.[7]

(*S*)-*N*-(1-(4-methylquinolin-2-yl)-4-phenylbutyl)acetamide (10)

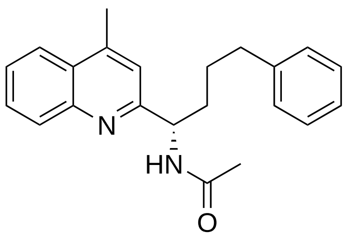

Following general procedure A with 4-methyl quinoline (28.6 mg, 0.20 mmol), *N*-(4-phenylbutyl)acetamide (383 mg, 2.0 mmol, 10 equiv.) and (*R*)-DIP (7.5 mg, 0.01 mmol, 5 mol%). The crude material was subjected to work-up procedure B and the resultant material was purified *via* flash chromatography (eluting with 50% EtOAc in hexanes) yield the title compound as a white amorphous solid (30.6 mg, 46%, 0.092 mmol, 86% ee).

^{1}H NMR (400 MHz, $CDCl_3$) δ 8.05 (d, *J* = 8.4 Hz, 1H), 7.97 (d, *J* = 8.2 Hz, 1H), 7.70 (t, *J* = 7.7 Hz, 1H), 7.55 (t, *J* = 7.5 Hz, 1H), 7.24-7.20 (m, 3H), 7.16-7.08 (m, 4H), 5.22 (q, *J* = 6.8 Hz, 1H), 2.68 (s, 3H), 2.65-2.52 (m, 2H), 2.08-2.01 (m, 4H), 1.93-1.84 (m, 1H), 1.64-1.55 (m, 2H); **^{13}C NMR** (101 MHz, $CDCl_3$) δ 169.7, 159.8, 147.2, 145.3, 142.3, 129.5, 128.5, 128.4, 127.6, 126.3, 125.8, 123.9, 121.1, 54.0, 36.1, 35.9, 27.2, 23.7, 18.9. **HRMS m/z:** $[M+H]^+$ calculated for $[C_{25}H_{22}N_2O]^+$ 333.1961, found: 333.1984. **SFC Analysis:** Chiralpak SC (CO_2/MeOH = 85/15, 2.5 mL min^{-1}, 40 °C) t_R = 6.5 (minor), 7.1 (major) minutes. **$[\alpha]_D^{25.0}$** = -27.7 (*c* 1.0, $CHCl_3$).

***N*-(1-(4-methylquinolin-2-yl)ethyl)acetamide (11)**

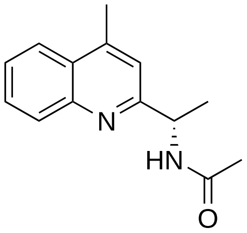

General procedure A was followed with 4-methylquinoline (28.6 mg, 0.20 mmol, 1.0 equiv.), (R)-TRIP (7.5 mg, 0.01 mmol, 5 mol%) and *N*-ethylacetamide (189 μL, 2.0 mmol, 10 equiv.). The crude material was subjected to work-up procedure A and the resultant material was purified *via* flash chromatography (eluting with 50% EtOAc in hexanes) yield the title compound as a white amorphous solid (34.2 mg, 75%, 0.150 mmol, 86% ee).

^{1}H NMR (600 MHz, $CDCl_3$) δ 8.05 (d, *J* = 8.5 Hz, 1H), 7.97 (dd, *J* = 8.3, 1.1 Hz, 1H), 7.72-7.69 (m, 1H), 7.57-7.49 (m, 2H), 7.16 (s, 1H), 5.21 (quint., *J* = 6.8 Hz, 1H), 2.69 (d, *J* = 0.5 Hz, 3H), 2.10 (s, 3H), 1.54 (d, *J* = 6.8 Hz, 3H). **^{13}C NMR** (151 MHz, $CDCl_3$) δ 169.6, 160.6, 147.0, 145.5, 129.5, 129.4, 127.5, 126.3, 123.9, 120.4, 50.1, 23.7, 22.8, 18.9. **HPLC Analysis:** Chiralpak IC (Hexane/^{i}PrOH = 90/10, 1.0 mL min^{-1}, 30 °C) t_R = 10.6 (minor), 13.1 (major) minutes. **$[\alpha]_D^{25.0}$** = -105.0 (*c* 1.0, $CHCl_3$). Data are in accordance with the literature.[7]

(*S*)-*N*-(1-(4-methylquinolin-2-yl)nonyl)acetamide (12)

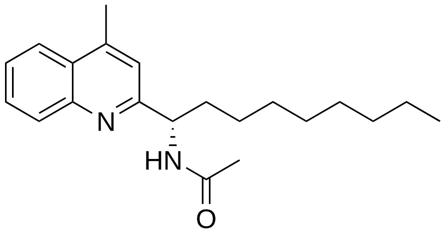

General procedure A was followed for 48 h reaction time with 4-methylquinoline (28.6 mg, 0.20 mmol, 1.0 equiv.), (*R*)-DIP (13.4 mg, 0.02 mmol, 10 mol%) and *N*-nonylacetamide (370 mg, 2.0 mmol, 10 equiv.). The crude material was subjected to work-up procedure B and the resultant material was purified *via* flash chromatography (eluting with 30% EtOAc in hexanes) to yield the title compound as a white amorphous solid (25.5 mg, 39%, 0.078 mmol, 85% ee).

^{1}H NMR (400 MHz, $CDCl_3$) δ 8.06 (d, *J* = 8.4 Hz, 1H), 7.98 (d, *J* = 8.3 Hz, 1H), 7.70 (t, *J* = 7.5 Hz, 1H), 7.55 (t, *J* = 7.6 Hz, 1H), 7.20 (d, *J* = 7.1 Hz, 1H), 7.16 (s, 1H), 5.18 (q, *J* = 6.8 Hz, 1H), 2.70 (s, 3H), 2.08 (s, 3H), 2.02-1.92 (m, 1H), 1.87-1.80 (m, 1H), 1.25-1.19 (m, 12H), 0.84 (t, *J* = 6.9 Hz, 1H); **^{13}C NMR** (101 MHz, $CDCl_3$) δ 169.6, 160.2, 147.2, 145.2, 129.5, 127.6, 126.2, 123.9, 121.2, 54.2, 36.5, 31.9, 29.7, 29.5, 29.4, 25.4, 23.8, 22.8, 19.0, 14.2. **HRMS m/z:** $[M+H]^+$ calculated for $[C_{21}H_{31}N_2O]^+$ 327.2431, found: 327.2440. **SFC Analysis:** Chiralpak SC (CO_2/MeOH = 90/10, 2.5 mL min^{-1}, 20 °C) t_R = 3.9 (minor), 4.5 (major) minutes. **$[\alpha]_D^{25.0}$** = -67.0 (c 1.0, $CHCl_3$).

(*S*)-*N*-(2-methyl-1-(4-methylquinolin-2-yl)propyl)acetamide (13)

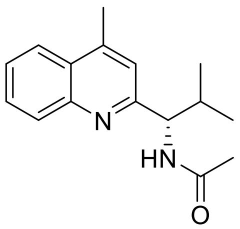

General procedure A was followed with 4-methylquinoline (28.6 mg, 0.20 mmol, 1.0 equiv.), (R)-TRIP (7.5 mg, 0.01 mmol, 5 mol%) and *N*-isobutylacetamide (130 mg, 2.0 mmol, 10 equiv.). The crude material was subjected to work-up procedure A and the resultant material was purified *via* flash chromatography (eluting with 50% EtOAc in hexanes) to yield the title compound as a white amorphous solid (28.2 mg, 55%, 0.11 mmol, 97% ee).

^{1}H NMR (600 MHz, $CDCl_3$) δ 8.05 (d, *J* = 8.4 Hz, 1H), 7.97 (d, *J* = 8.4 Hz, 1H), 7.71 (t, *J* = 8.1, 7.1, 1.0 Hz, 1H), 7.54 (ddd, *J* = 8.1, 7.1, 1.0 Hz, 1H), 7.15 (s, 1H), 7.10 (br d, *J* = 8.2 Hz, 1H), 5.05 (dd, *J* = 8.7, 6.4 Hz, 1H), 2.69 (s, 3H), 2.24 (sep, *J* = 6.4 Hz, 1H), 2.08 (s, 3H), 0.94 (d, *J* = 6.8 Hz, 3H), 0.88 (d, *J* = 6.8 Hz, 3H); **^{13}C NMR** (151 MHz, $CDCl_3$) δ 169.8, 159.6, 147.3, 144.6, 129.6, 129.4, 127.5, 126.1, 123.9, 122.1, 59.1, 34.3, 23.8, 19.5, 18.9, 18.5; **HPLC Analysis:** Chiralpak IC (Hexane/^{i}PrOH = 70/30, 1.0 mL min^{-1}, 30 °C) t_R = 7.1 (minor), 10.3 (major) minutes. **$[\alpha]_D^{25.0}$** = -133.7 (*c* 1.0, $CHCl_3$). Data are in accordance with the literature.[7]

***tert*-butyl (*S*)-4-acetamido-4-(4-methylquinolin-2-yl)butanoate (15)**

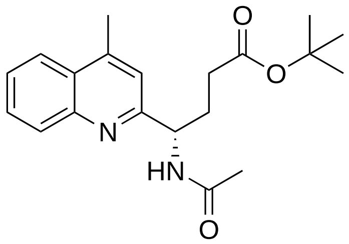

General procedure A was followed for 48 h reaction time with 4-methylquinoline (28.6 mg, 0.20 mmol, 1.0 equiv.), (*R*)-DIP (6.7 mg, 0.01 mmol, 5 mol%) and *tert*-butyl 4-acetamidobutanoate (403 mg, 2.0 mmol, 10 equiv.). The crude material was subjected to work-up procedure A and the resultant material was purified via flash chromatography (eluting with 50% EtOAc in hexanes) to yield the title compound as a white amorphous solid (42.7 mg, 71%, 0.142 mmol, 90% ee).

^{1}H NMR (400 MHz, $CDCl_3$) δ 8.05 (d, *J* = 8.4 Hz, 1H), 7.97 (d, *J* = 8.3 Hz, 1H), 7.70 (t, *J* = 7.8 Hz, 1H), 7.54 (t, *J* = 7.7 Hz, 1H), 7.33 (d, *J* = 7.6 Hz, 1H), 7.20 (s, 1H), 5.24 (td, *J* = 7.2, 5.0, 1H), 2.69 (s, 3H), 2.35-2.03 (m, 7H), 1.38 (s, 9H); **^{13}C NMR** (101 MHz, $CDCl_3$) δ 172.7, 169.9, 159.2, 147.1, 145.6, 129.6, 129.5, 127.6, 126.4, 123.9, 120.9, 80.5, 53.4, 31.5, 31.4, 28.1, 23.7, 18.9. **HPLC Analysis:** Chiralpak IC (Hexane/^{i}PrOH = 70/30, 1.0 mL min^{-1}, 30 °C) t_R = 7.1 (minor), 10.3 (major) minutes **$[\alpha]_D^{25.0}$** = -41.0 (*c* 1.0, $CHCl_3$). Data are in accordance with the literature.[7]

Methyl (2*S*,6*S*)-6-acetamido-2-((*t*-butoxycarbonyl)amino)-6-(4-methylquinolin-2-yl)hexanoate (16)

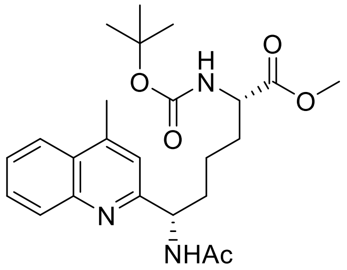

General procedure A was followed with 4-methylquinoline (21.5 mg, 0.15 mmol, 1.0 equiv.), (*R*)-DIP (5.0 mg, 0.008 mmol, 5 mol%), diacetyl (130 µL, 1.5 mmol, 10 equiv.) and methyl *N*-acetyl-*N*-(tert-butoxycarbonyl)-L-lysinate (454 mg, 1.5 mmol, 10 equiv.). The solvent was concentrated *in vacuo* and the crude material was purified via flash chromatography (eluting with 50% EtOAc in hexanes) followed by subsequent flash chromatography (eluting with 2% MeOH in dichloromethane) to yield the title compound as a yellow oil (34 mg, 51% yield, 0.076 mmol, 23:1 d.r. as determined by SFC).

Note: Despite purification twice *via* flash column chromatography, it was not possible to completely isolate compound **16** 100% free from small amounts of the closely eluting impurities, particularly the amide starting material.

^{1}H NMR (400 MHz, $CDCl_3$) δ 8.04 (d, *J* = 8.4 Hz, 1H), 7.97 (d, *J* = 8.3 Hz, 1H), 7.70 (app t, *J* = 7.7 Hz, 1H), 7.55 (app t, *J* = 7.7 Hz, 1H), 7.21 (d, J = 7.5 Hz, 1H), 7.14 (s, 1H), 5.22 – 5.14 (m, 1H), 5.08 (d, *J* = 8.1 Hz, 1H), 4.24 – 4.16 (m, 1H), 3.66 (s, 3H), 2.70 (s, 3H), 2.10 (s, 3H), 1.99 – 1.91 (m, 2H), 1.89 – 1.78 (m, 2H), 1.43 – 1.32 (m, 2H overlapping with singlet δ 1.40), 1.40 (s, 9H, overlapping with multiplet δ 1.43 – 1.32); **^{13}C NMR** (101 MHz, $CDCl_3$) δ 173.5, 169.9, 159.7, 155.6, 147.3, 145.3, 129.6, 129.5, 127.6, 126.3, 123.9, 120.9, 79.8, 53.6, 53.3, 52.3, 36.0, 32.5, 28.4, 23.7, 21.3, 18.9. **HRMS m/z:** $[M+H]^+$ calculated for $[C_{24}H_{34}N_3O_5]^+$ 444.2493, found: 444.2494. **SFC Analysis:** Chiralpak SC (CO_2/MeOH = 80/20, 2.5 mL min^{-1}, 20 °C) t_R = 6.1 (minor diastereomer), 7.2 (major diastereomer) minutes. **$[\alpha]_D^{25.0}$** = -20.7 (c 1.0, $CHCl_3$).

(S)-*N*-(2-phenyl-1-(quinolin-2-yl)ethyl)acetamide (17)

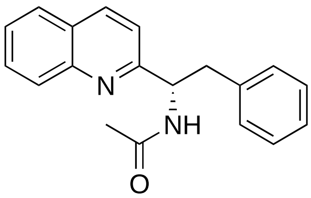

General procedure A was followed for 24 h reaction time with quinoline (26.4 mg, 0.204 mmol, 1.0 equiv.), (*R*)-TRIP (7.5 mg, 0.01 mmol, 5 mol%) and *N*-phenethylacetamide (326.4 mg, 2.0 mmol, 10 equiv.). The crude material was subjected to work-up procedure B and the resultant material was purified *via* flash chromatography (eluting with 30% EtOAc in hexanes) to yield the title compound as a white amorphous solid (25 mg, 42% yield, 0.086 mmol, 95% ee).

^{1}H NMR (400 MHz, $CDCl_3$) δ 8.14 (br s, 1H), 8.03 (d, *J* = 8.4 Hz, 1H), 7.78 (d, *J* = 7.8 Hz, 1H), 7.75 (t, *J* = 7.4 Hz, 1H), 7.56 (t, *J* = 7.4 Hz, 1H), 7.48-7.31 (m, 1H), 7.17-7.11 (m, 3H), 7.01-6.93 (m, 3H), 5.50-5.43 (m, 1H), 3.39 (dd, *J* = 13.5, 5.1 Hz, 1H), 3.18 (dd, *J* = 13.5, 8.2 Hz, 1H), 2.09 (s, 3H); **^{13}C NMR** (101 MHz, $CDCl_3$) δ 169.5, 159.2, 147.5, 137.2, 136.3, 129.8, 129.7, 129.1, 128.3, 127.8, 127.5, 126.6, 126.5, 121.0, 55.7, 42.5, 23.7; **SFC Analysis**: Chiralpak IG (CO_2/MeOH = 85/15, 2.5 mL min^{-1}, 40 °C) t_R = 8.1 (minor), 9.1 (major) minutes. **$[\alpha]_D^{25.0}$** = +60.8 (c 1.0, $CHCl_3$). Data are in accordance with the literature.[7]

(*S*)-*N*-(1-(6-methoxyquinolin-2-yl)-2-phenylethyl)acetamide (18)

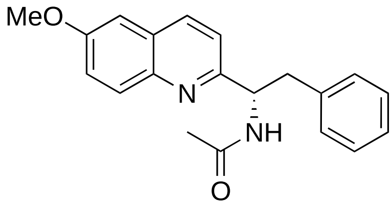

General procedure A was followed for 24 h reaction time with 6-methoxyquinoline (31.8 mg, 0.20 mmol, 1.0 equiv.), (*R*)-TRIP (7.5 mg, 0.01 mmol, 5 mol%) and *N*-phenethylacetamide (326.4 mg, 2.0 mmol, 10 equiv.). The crude material was subjected to work-up procedure B and the resultant material was purified *via* flash chromatography (eluting with 30% EtOAc in hexanes) yield the title compound as a white amorphous solid (39.1 mg, 61%, 0.122 mmol, 90% ee).

^{1}H NMR (400 MHz, $CDCl_3$) δ 8.12 (d, *J* = 9.2 Hz, 1H), 7.95 (d, *J* = 8.4 Hz, 1H), 7.64-7.46 (m, 1H), 7.41 (dd, *J* = 9.2, 2.7 Hz, 1H), 7.17-7.12 (m, 3H), 7.07 (d, *J* = 2.7 Hz, 1H), 7.00-6.93 (m, 3H), 5.48-5.40 (m, 1H), 3.93 (s, 3H), 3.39 (dd, *J* = 13.2, 5.3 Hz, 1H), 3.19 (dd, *J* = 13.2, 8.2 Hz, 1H), 2.08 (s, 3H); **^{13}C NMR** (101 MHz, $CDCl_3$) δ 169.7, 158.1, 156.5, 136.9, 129.7, 129.6, 128.6, 128.4, 128.2, 126.5, 123.3, 121.3, 105.1, 55.6, 55.3, 42.2, 23.5; **SFC Analysis**: Chiralpak SC (CO_2/MeOH = 90/10, 2.5 mL min^{-1}, 40 °C) t_R = 12.5 (minor), 13.2 (major) minutes. **$[\alpha]_D^{25.0}$** = +33.6 (c 1.0, $CHCl_3$). Data are in accordance with the literature.[7]

(S)-*N*-(1-(6-chloroquinolin-2-yl)-2-phenylethyl)acetamide (19)

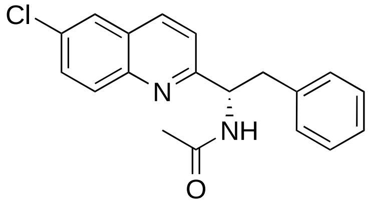

General procedure A was followed for 24 h reaction time with 6-chloroquinoline (32.7 mg, 0.20 mmol, 1.0 equiv.), (*R*)-TRIP (7.5 mg, 0.01 mmol, 5 mol%) and *N*-phenethylacetamide (326.4 mg, 2.0 mmol, 10 equiv.). The crude material was subjected to work-up procedure B and the resultant material was purified *via* flash chromatography (eluting with 30% EtOAc in hexanes) yield the title compound as a white amorphous solid (45.5 mg, 70% yield, 0.140 mmol, 90% ee).

^{1}H NMR (400 MHz, $CDCl_3$) δ 7.99 (d, *J* = 8.9 Hz, 1H), 7.90 (d, *J* = 8.5 Hz, 1H), 7.77 (d, *J* = 2.2 Hz, 1H), 7.65 (dd, *J* = 8.9, 2.2 Hz, 1H), 7.21-7.12 (m, 4H), 6.98 (d, *J* = 8.5 Hz, 1H), 6.95-6.89 (m, 2H), 5.48-5.40 (m, 1H), 3.35 (dd, *J* = 13.2, 5.2 Hz, 1H), 3.14 (dd, *J* = 13.2, 8.2 Hz, 1H), 2.09 (s, 3H); **^{13}C NMR** (101 MHz, $CDCl_3$) δ 169.5, 159.6, 145.6, 136.8, 135.4, 132.2, 130.6, 130.4, 129.6, 128.2, 127.9, 126.6, 126.3, 121.7, 55.6, 42.3, 23.5; **SFC Analysis**: Chiralpak SC (CO_2/MeOH = 90/10, 2.5 mL min^{-1}, 40 °C) t_R = 10.3 (minor), 11.0 (major) minutes. **$[\alpha]_D^{25.0}$** = +30.2 (c 1.0, $CHCl_3$). Data are in accordance with the literature.[7]

(S)-*N*-(1-(3-methylquinolin-2-yl)-2-phenylethyl)acetamide (20)

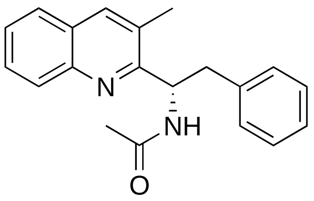

General procedure A was followed for 24 h reaction time with 3-methylquinoline (28.6 mg, 0.20 mmol, 1.0 equiv.), (*R*)-TRIP (7.5 mg, 0.01 mmol, 5 mol%) and *N*-phenethylacetamide (326.4 mg, 2.0 mmol, 10 equiv.). The crude material was subjected to work-up procedure B and the resultant material was purified *via* flash chromatography (eluting with 30% EtOAc in hexanes) yield the title compound as a white amorphous solid (51.7 mg, 85% yield, 0.170 mmol, 93% ee).

^{1}H NMR (400 MHz, $CDCl_3$) δ 8.03 (d, *J* = 8.3 Hz, 1H), 7.80 (s, 1H), 7.73 (d, *J* = 8.1 Hz, 1H), 7.66 (t, *J* = 7.6 Hz, 1H), 7.51 (t, *J* = 7.5 Hz, 1H), 7.25-7.17 (m, 1H), 7.16-7.07 (m, 3H), 6.91-6.86 (m, 2H), 5.71-5.64 (m, 1H), 3.30-3.16 (m, 2H), 2.15 (s, 3H), 2.05 (s, 3H); **^{13}C NMR** (101 MHz, $CDCl_3$) δ 169.2, 159.4, 145.9, 137.0, 136.5, 129.7, 129.3, 128.8, 128.3, 128.1, 127.6, 126.9, 126.5, 126.4, 51.7, 42.4, 23.5, 18.4; **SFC Analysis**: Chiralpak SC (CO_2/MeOH = 90/10, 2.5 mL min^{-1}, 40 °C) t_R = 6.6 (minor), 7.1 (major) minutes. **$[\alpha]_D^{25.0}$** = +42.9 (c 1.0, $CHCl_3$). Data are in accordance with the literature.[7]

(S)-*N*-(1-(phenanthridin-6-yl)-2-phenylethyl)acetamide (21)

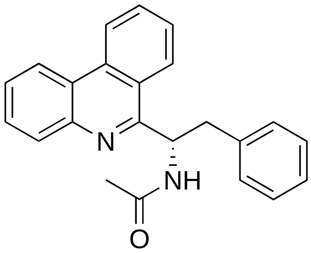

General procedure A was followed for 24 h reaction time with phenanthridine (35.8 mg, 0.20 mmol, 1.0 equiv.), (*R*)-TRIP (7.5 mg, 0.01 mmol, 5 mol%) and *N*-phenethylacetamide (326.4 mg, 2.0 mmol, 10 equiv.). The crude material was subjected to work-up procedure B and the resultant material was purified *via* flash chromatography (eluting with 30% EtOAc in hexanes) yield the title compound as a white amorphous solid (48.3 mg, 71%, 0.142 mmol, 83% ee).

^{1}H NMR (400 MHz, $CDCl_3$) δ 8.64 (d, *J* = 8.3 Hz, 1H), 8.57 (d, *J* = 7.9 Hz, 1H), 8.17 (d, *J* = 8.3 Hz, 1H), 8.05 (d, *J* = 7.6 Hz, 1H), 7.86-7.79 (m, 1H), 7.76-7.59 (m, 3H), 7.36 (d, *J* = 7.7 Hz, 1H), 7.09-7.02 (m, 3H), 6.88-6.81 (m, 2H), 6.33-6.26 (m, 1H), 3.45 (dd, *J* = 13.4, 6.7 Hz, 1H), 3.31 (dd, *J* = 13.4, 5.2 Hz, 1H), 2.10 (s, 3H); **^{13}C NMR** (101 MHz, $CDCl_3$) δ 169.2, 158.5, 142.7, 136.9, 132.9, 130.7, 129.8, 129.6, 128.7, 127.9, 127.5, 127.0, 126.3, 125.5, 123.9, 123.8, 122.4, 122.1, 50.8, 42.0, 23.6; **SFC Analysis**: Chiralpak SC (CO_2/MeOH = 85/15, 2.5 mL min^{-1}, 40 °C) t_R = 8.1 (minor), 8.7 (major) minutes. **$[\alpha]_D^{25.0}$** = -14.0 (c 1.0, $CHCl_3$). Data are in accordance with the literature.[7]

(*S*)-*N*-(1-(4-(4-bromophenoxy)quinolin-2-yl)-2-phenylethyl)acetamide (22)

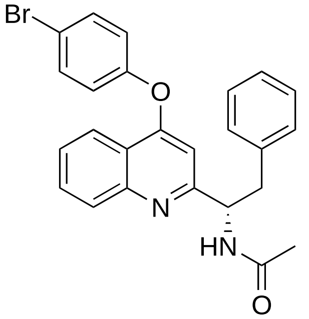

General procedure A was followed for 24 h reaction time with 4-(4-bromophenoxy)quinoline (60.0 mg, 0.20 mmol, 1.0 equiv.), (*R*)-TRIP (7.5 mg, 0.01 mmol, 5 mol%) and *N*-phenethylacetamide (326.4 mg, 2.0 mmol, 10 equiv.). The crude material was subjected to work-up procedure B and the resultant

material was purified *via* flash chromatography (eluting with 30% EtOAc in hexanes) yield the title compound as a white amorphous solid (50.7 mg, 55%, 0.110 mmol, 96% ee).

Note: It was impossible to get compound **20** 100% free from very small amounts of co-running impurities that are believed to originate_from by-products arising from the excess of diacetyl and amide.

^{1}H NMR (400 MHz, $CDCl_3$) δ 8.24 (d, *J* = 8.2 Hz, 1H), 8.07 (d, *J* = 8.5 Hz, 1H), 7.77 (t, *J* = 7.9 Hz, 1H), 7.56 (t, *J* = 7.9 Hz, 1H), 7.46 (d, *J* = 8.7 Hz, 2H), 7.36-7.31 (m, 1H), 7.19-7.11 (m, 3H), 6.95-6.89 (m, 2H), 6.75 (d, *J* = 8.7 Hz, 2H), 5.94 (s, 1H), 5.21-5.14 (m, 1H), 3.35 (dd, *J* = 13.0, 5.0 Hz, 1H), 2.92 (dd, *J* = 13.0, 9.3 Hz, 1H), 2.06 (s, 3H). **^{13}C NMR** (101 MHz, $CDCl_3$) δ 169.4, 160.9, 160.3, 152.8, 148.8, 137.3, 133.2, 130.5, 129.5, 128.4, 128.3, 126.4, 126.1, 122.6, 121.8, 120.4, 118.5, 104.1, 56.1, 42.8, 23.5 ; **SFC Analysis**: Chiralpak SJ (CO_2/MeOH = 80/20, 2.5 mL min^{-1}, 40 °C) t_R = 2.7 (major), 2.9 (minor) minutes. **$[\alpha]_D^{25.0}$** = +81.7 (c 1.0, $CHCl_3$). Data are in accordance with the literature.[7]

methyl (*S*)-6-(1-acetamido-2-phenylethyl)-2-methylnicotinate (23)

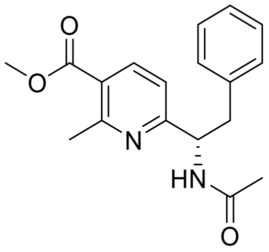

General procedure A was followed for 48 h reaction time with methyl 2-methylnicotinate (30.2 mg, 0.20 mmol, 1.0 equiv.), (*R*)-TCYP (19.8 mg, 0.02 mmol, 10 mol%), diacetyl (348 μL, 4.0 mmol, 20 equiv.) and *N*-phenethylacetamide (326.4 mg, 2.0 mmol, 10 equiv.). The crude material was subjected to work-up procedure B and the resultant material was purified *via* flash chromatography (eluting with 30% EtOAc in hexanes) yield the title compound as a white amorphous solid (38.1 mg, 61% yield, 0.122 mmol, 86% ee).

^{1}H NMR (400 MHz, $CDCl_3$) δ 7.99 (d, *J* = 8.0 Hz, 1H), 7.21-7.13 (m, 3H), 6.96-6.87 (m, 3H), 6.69 (d, *J* = 8.0 Hz, 1H), 5.31-5.23 (m, 1H), 3.88 (s, 3H), 3.25 (dd, *J* = 13.4, 5.4 Hz, 1H), 2.99 (dd, *J* = 13.2, 8.4 Hz, 1H), 2.81 (s, 3H), 2.03 (s, 3H); **^{13}C NMR** (101 MHz, $CDCl_3$) δ 169.3, 166.7, 161.0, 159.7, 138.7, 137.0, 129.5, 128.2, 126.5, 123.9, 119.8, 55.2, 52.2, 42.4, 24.8, 23.5; **HRMS** m/z: $[M + H]^+$ calc'd for

$[C_{18}H_{21}N_2O_3]^+$ expect 313.1547; found 313.1552; **SFC Analysis**: Chiralpak SC (CO_2/MeOH = 90/10, 2.5 mL min^{-1}, 40 °C) t_R = 6.4 (minor), 7.1 (major) minutes. **[α]$_D^{25.0}$** = +22.0 (c 1.0, $CHCl_3$).

methyl (*S*)-6-(1-acetamido-2-phenylethyl)nicotinate (24)

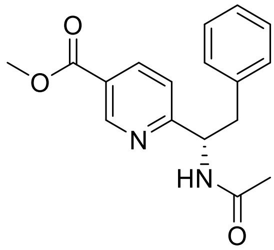

General procedure A was followed for 48 h reaction time with methyl methyl nicotinate (27.4 mg, 0.20 mmol, 1.0 equiv.), (*R*)-TCYP (19.8 mg, 0.02 mmol, 10 mol%), diacetyl (348 μL, 4.0 mmol, 20 equiv.) and *N*-phenethylacetamide (326.4 mg, 2.0 mmol, 10 equiv.). The crude material was subjected to work-up procedure B and the resultant material was purified *via* flash chromatography (eluting with 30% EtOAc in hexanes) yield the title compound as a white amorphous solid (27.4 mg, 46%, 0.092 mmol, 81% ee).

^{1}H NMR (400 MHz, $CDCl_3$) δ 9.13 (d, *J* = 1.8 Hz, 1H), 8.10 (dd, *J* = 8.1, 2.1 Hz, 1H), 7.20-7.15 (m, 3H), 6.95-6.89 (m, 3H), 6.86-6.79 (m, 1H), 5.37-5.30 (m, 1H), 3.95 (s, 3H), 3.26 (dd, *J* = 13.2, 5.2 Hz, 1H), 3.03 (dd, *J* = 13.2, 8.4 Hz, 1H), 2.03 (s, 3H); **^{13}C NMR** (101 MHz, $CDCl_3$) δ 169.4, 165.5, 163.0, 150.3, 137.4, 136.6, 129.4, 128.3, 126.7, 124.9, 122.4, 55.4, 52.4, 42.3, 23.4; **HRMS** m/z: $[M + H]^+$ calc'd for $[C_{17}H_{19}N_2O_3]^+$ expect 299.1390; found 299.1392; **SFC Analysis**: Chiralpak SC (CO_2/MeOH = 93/7, 2.5 mL min^{-1}, 40 °C) t_R = 12.4 (minor), 13.3 (major) minutes. **[α]$_D^{25.0}$** = +7.3 (c 1.0, $CHCl_3$).

methyl (*S*)-6-(1-acetamido-2-phenylethyl)-4-methylnicotinate (25)

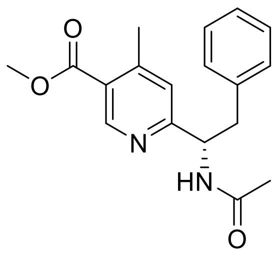

General procedure A was followed for 48 h reaction time with methyl methyl 4-methylnicotinate (30.2 mg, 0.20 mmol, 1.0 equiv.), (*R*)-TCYP (19.8 mg, 0.02 mmol, 10 mol%), diacetyl (348 μL, 4.0 mmol, 20 equiv.) and *N*-phenethylacetamide (326.4 mg, 2.0 mmol, 10 equiv.). The crude material was subjected to work-up procedure B and the resultant material was purified *via* flash chromatography (eluting with

30% EtOAc in hexanes) yield the title compound as a white amorphous solid (23.1 mg, 37%, 0.074 mmol, 79% ee).

^{1}H NMR (400 MHz, $CDCl_3$) δ 8.98 (s, 1H), 7.22-7.15 (m, 3H), 6.96-6.90 (m, 2H), 6.80-6.74 (m, 1H), 6.73 (s, 1H), 5.29-5.22 (m, 1H), 3.92 (s, 3H), 3.22 (dd, *J* = 13.5, 5.5 Hz, 1H), 3.03 (dd, *J* = 13.5, 8.2 Hz, 1H), 2.48 (s, 3H), 2.00 (s, 3H); **^{13}C NMR** (101 MHz, $CDCl_3$) δ 169.2, 165.7, 161.9, 147.7, 138.9, 136.6, 131.4, 129.5, 128.2, 126.6, 124.6, 52.3, 51.4, 42.1, 23.4, 17.7; **HRMS** m/z: $[M + H]^+$ calc'd for $[C_{18}H_{21}N_2O_3]^+$ expect 313.1547; found 313.1552; **SFC Analysis**: Chiralpak SC (CO_2/MeOH = 93/7, 2.5 mL min^{-1}, 40 °C) t_R = 10.7 (minor), 11.4 (major) minutes. **$[\alpha]_D^{25.0}$** = -2.6 (c 1.0, $CHCl_3$).

methyl (*S*)-6-(1-acetamido-2-phenylethyl)-5-methylnicotinate (26)

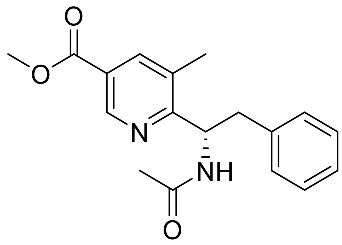

General procedure A was followed for 48 h reaction time with methyl methyl 5-methylnicotinate (30.2 mg, 0.20 mmol, 1.0 equiv.), (*R*)-TRIP (15.0 mg, 0.02 mmol, 10 mol%), diacetyl (348 μL, 4.0 mmol, 20 equiv.) and *N*-phenethylacetamide (326.4 mg, 2.0 mmol, 10 equiv.). The crude material was subjected to work-up procedure B and the resultant material was purified *via* flash chromatography (eluting with 30% EtOAc in hexanes) yield the title compound as a white amorphous solid (37.5 mg, 60% yield, 0.120 mmol, 73% ee).

^{1}H NMR (400 MHz, $CDCl_3$) δ 8.97 (d, *J* = 1.4 Hz, 1H), 7.95-7.91 (m, 1H), 7.18-7.10 (m, 3H), 6.90-6.80 (m, 3H), 5.59-5.50 (m, 1H), 3.94 (s, 3H), 3.18 (dd, *J* = 13.2, 5.2 Hz, 1H), 3.04 (dd, *J* = 13.2, 8.6 Hz, 1H), 2.00 (s, 3H), 1.99 (s, 3H); **^{13}C NMR** (101 MHz, $CDCl_3$) δ 169.2, 165.7, 161.9, 147.7, 138.9, 136.6, 131.4, 129.5, 128.2, 126.6, 124.6, 52.3, 51.4, 42.1, 23.4, 17.7; **HRMS** m/z: $[M + H]^+$ calc'd for $[C_{18}H_{21}N_2O_3]^+$ expect 313.1547; found 313.1550; **SFC Analysis**: Chiralpak SC (CO_2/MeOH = 93/7, 2.5 mL min^{-1}, 40 °C) t_R = 9.3 (minor), 10.2 (major) minutes. **$[\alpha]_D^{25.0}$** = +8.4 (c 1.0, $CHCl_3$).

(*S*)-*N*-(2-phenyl-1-(5-propionylpyridin-2-yl)ethyl)acetamide (27)

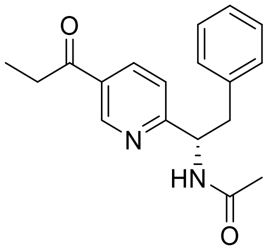

General procedure A was followed for 48 h reaction time with methyl 1-(pyridin-3-yl)propan-1-one (27.0 mg, 0.20 mmol, 1.0 equiv.), (*R*)-TCYP (19.8 mg, 0.02 mmol, 10 mol%), diacetyl (348 μL, 4.0 mmol, 20 equiv.) and *N*-phenethylacetamide (326.4 mg, 2.0 mmol, 10 equiv.). The crude material was subjected to work-up procedure B and the resultant material was purified *via* flash chromatography (eluting with 30% EtOAc in hexanes) yield the title compound as a white amorphous solid (35.0 mg, 59% yield, 0.118 mmol, 87% ee).

^{1}H NMR (400 MHz, $CDCl_3$) δ 9.09-9.07 (m, 1H), 8.06-8.01 (m, 1H), 7.21-7.14 (m, 3H), 6.98-6.89 (m, 3H), 6.86-6.77 (m, 1H), 5.38-5.30 (m, 1H), 3.29-3.22 (m, 1H), 3.08-2.94 (m, 3H), 2.03 (s, 3H), 1.23 (t, *J* = 7.2 Hz, 3H); **^{13}C NMR** (101 MHz, $CDCl_3$) δ 199.0, 169.4, 162.9, 148.9, 136.6, 135.7, 131.0, 129.4, 128.3, 126.7, 122.7, 55.4, 42.2, 32.1, 23.4, 7.8; **HRMS** m/z: $[M + H]^+$ calc'd for $[C_{18}H_{21}N_2O_2]^+$ expect 297.1598; found 297.1601; **SFC Analysis**: Chiralpak SC (CO_2/MeOH = 90/10, 2.5 mL min^{-1}, 40 °C) t_R = 9.9 (minor), 10.8 (major) minutes. **$[\alpha]_D^{25.0}$** = +6.8 (c 1.0, $CHCl_3$).

(S)-*N*-(1-(5-cyano-6-methylpyridin-2-yl)-2-phenylethyl)acetamide (28)

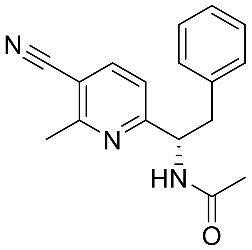

General procedure A was followed for 48 h reaction time with 2-methylnicotinonitrile (23.6 mg, 0.20 mmol, 1.0 equiv.), (*R*)-TCYP (19.8 mg, 0.02 mmol, 10 mol%), diacetyl (348 μL, 4.0 mmol, 20 equiv.) and *N*-phenethylacetamide (326.4 mg, 2.0 mmol, 10 equiv.). The crude material was subjected to work-up procedure B and the resultant material was purified *via* flash chromatography (eluting with 30% EtOAc in hexanes) yield the title compound as a white amorphous solid (26.8 mg, 48% yield, 0.096 mmol, 92% ee).

^{1}H NMR (400 MHz, $CDCl_3$) δ 7.68 (d, *J* = 8.0 Hz, 1H), 7.23-7.17 (m, 3H), 6.96-6.91 (m, 2H), 6.75 (d, J = 8.0 Hz, 1H), 6.69-6.62 (m, 1H), 5.33-5.26 (m, 1H), 3.24 (dd, *J* = 13.8, 5.7 Hz, 1H), 2.99 (dd, *J* = 13.5, 8.3 Hz, 1H), 2.77 (s, 3H), 2.03 (s, 3H); **^{13}C NMR** (101 MHz, $CDCl_3$) δ 169.4, 162.3, 161.4, 140.1, 136.5, 129.3, 128.4, 126.8, 119.9, 116.8, 107.7, 55.4, 42.2, 23.6, 23.4; **HRMS** m/z: $[M + H]^+$ calc'd for $[C_{17}H_{17}N_3O]^+$ expect 280.1444; found 280.1450; **SFC Analysis**: Chiralpak SC (CO_2/MeOH = 90/10, 2.5 mL min^{-1}, 40 °C) t_R = 6.9 (minor), 7.6 (major) minutes. **$[\alpha]_D^{25.0}$** = +6.1 (c 1.0, $CHCl_3$).

(*S*)-*N*-(1-(2-methyl-6-phenylpyrimidin-4-yl)-2-phenylethyl)acetamide (29)

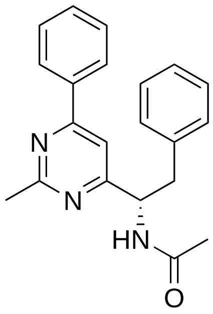

General procedure A was followed for 48 h reaction time with 2-methyl-4-methylpyrimidine (28.6 mg, 0.20 mmol, 1.0 equiv.), (*R*)-TRIP (7.5 mg, 0.01 mmol, 5 mol%) and *tert*-butyl 4-acetamidobutanoate (403 mg, 2.0 mmol, 10 equiv.). The crude material was subjected to work-up procedure A and the resultant material was purified via flash chromatography (eluting with 50% EtOAc in hexanes) yield the title compound as a white amorphous solid (21.0 mg, 32%, 0.64 mmol, 95% ee).

^{1}H NMR (400 MHz, $CDCl_3$) δ 7.83-7.83 (m, 2H), 7.48-7.41 (m, 3H), 7.32-7.18, (m, 3H, overlapped with solvent peak), 7.03-7.01 (m, 2H), 6.92 (s, 1H), 6.87 (d, *J* = 7.6 Hz, 1H), 5.26 (td, J = 8.1, 5.7 Hz, 1H), 3.31 (dd, *J* = 13.3, 5.7 Hz, 1H), 2.98 (dd, *J* = 13.3, 8.5 Hz, 1H), 2.78 (s, 3H, 2.06 (s, 3H). **^{13}C NMR** (101 MHz, $CDCl_3$) δ 169.6, 168.2, 167.3, 164.2, 136.9, 136.9, 130.9, 129.7, 129.3, 129.3, 129.0, 128.8, 128.5, 127.4, 126.9, 112.7, 55.4, 42.1, 26.4, 23.6.; **SFC Analysis**: Chiralpak SC (CO_2/MeOH = 90/10, 2.5 mL min^{-1}, 40 °C) t_R = 8.1 (minor), 8.9 (major) minutes. Data are in accordance with the literature.[8] **$[\alpha]_D^{25.0}$** = -15.7 (c 1.0, $CHCl_3$).

1 mmol scale: (*S*)-*N*-(1-(4-methylquinolin-2-yl)-2-phenylethyl)acetamide (3)

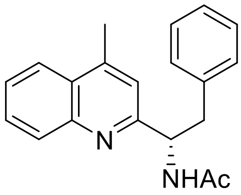

In a slight modification to the General procedure, diacetyl and *tert*-butyl acetate were thoroughly degassed *via* freeze-pump-thaw procedure prior to use. Sequentially, 4-methylquinoline (143 mg, 1 mmol, 1.0 equiv.), *N*-acetylphenethylamine (1.63 g, 10.0 mmol, 10 equiv.) and (*R*)-TRIP (38 mg, 0.05 mmol, 5 mol%) were weighed into a 100 mL Schlenk tube containing a stirrer bar. The flask was sealed with a septum, evacuated and refilled with argon three times. Under flow of inert gas, diacetyl (0.87 mL, 10.0 mmol, 10 equiv.) and anhydrous *tert*-butyl acetate (80 mL) were then added *via* syringe. The flask was then sealed with a PTFE Schlenk cap and the reaction mixture was stirred under irradiation of two Kessil Tuna blue lamps mounted within 2 cm to the edge of the reaction flask (100% intensity, 100% Ocean Blue colour) for 24 hours. The apparatus was maintained at approximately room temperature by mounting two desk fans close to the reaction flask. The crude material was subjected to work-up procedure A and the resultant material was purified *via* flash chromatography (eluting with 50% EtOAc in hexanes) to yield the title compound as a white solid (223 mg, 73% yield, 0.733 mmol, 95% ee).

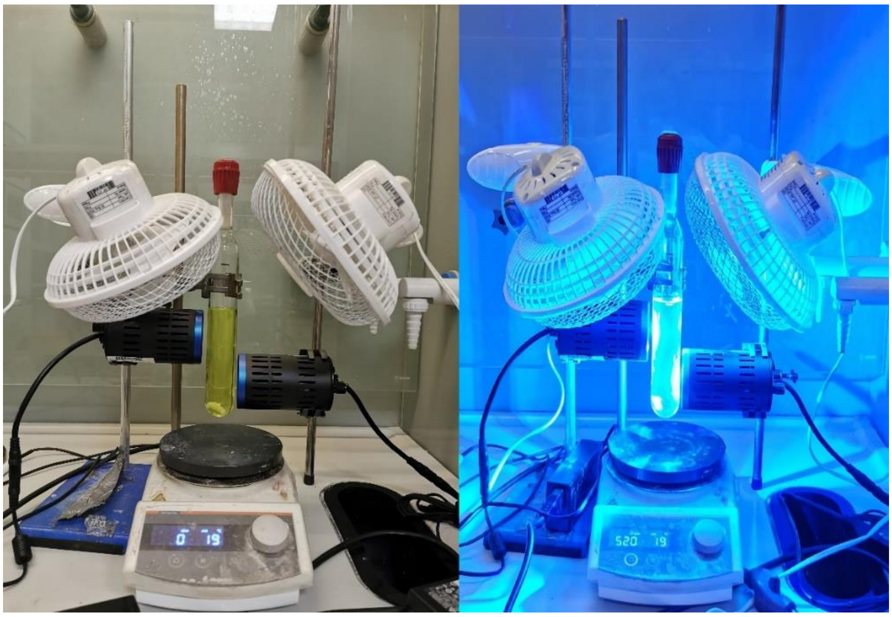

Figure 2: Photo of reaction set-up on 1 mmol scale

Deprotection of acetyl protecting group without loss of stereochemical integrity

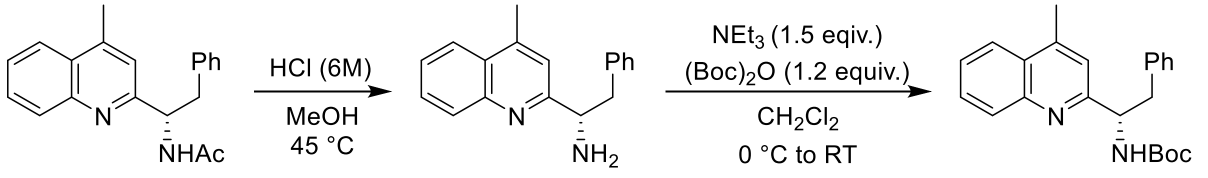

3
95% ee

30
95% ee

A mixture of (*S*)-*N*-(1-(4-methylquinolin-2-yl)-2-phenylethyl)acetamide (**3**) (80 mg, 0.26 mmol), methanol (8 mL) and 6M HCl (8 mL) was heated to 45 °C for 36 h. The reaction mixture was then basified to pH = 8 and extracted with dichloromethane (3 x 30 ml). The combined organic phases were dried over Na_2SO_4, filtered and concentrated *in vacuo.* The crude material was then dissolved in CH_2Cl_2 (12 mL) and cooled to 0 °C. Triethylamine (55 µL, 0.40 mmol, 1.5 equiv.) was added to the reaction mixture followed by di-*tert*-butyl dicarbonate (69 mg, 0.32 mmol, 1.2 equiv.). The flask was warmed to RT and stirred for 4 h. The solvent was concentrated *in vacuo* and the resultant material was purified via flash chromatography (eluting with 5% EtOAc in hexanes) to give **29** as a white solid (52 mg, 55% yield over two steps, 95% ee).

***tert*-butyl (*S*)-(1-(4-methylquinolin-2-yl)-2-phenylethyl)carbamate (30)**

^{1}H NMR (400 MHz, $CDCl_3$) δ 8.05 (d, *J* = 8.0 Hz, 1H), 7.95 (d, *J* = 8.2 Hz, 1H), 7.69 (t, *J* = 7.4 Hz, 1H), 7.54 (t, *J* = 7.4 Hz, 1H), 7.16 (br s, 3H), 7.01 (br s, 2H), 6.82 (br s, 1H), 6.13 (br s, 0.84H), 5.80 (br s, 0.15H), 5.15 – 4.91 (m, 1H), 3.34 – 3.24 (m, 1H), 3.22 – 3.10 (m, 1H), 2.58 (s, 3H), 1.45 (br s, 9H). **^{13}C NMR** (101 MHz, $CDCl_3$) δ 159.5, 155.5, 147.4, 144.4, 137.6, 129.8 (2 x C), 129.3, 128.2, 127.5, 126.4, 126.1, 123.8, 121.4, 79.4, 57.0, 42.8, 28.6, 18.8. Data are in accordance with the literature.[7] **SFC Analysis**: Chiralpak SC (CO_2/MeOH = 95/05, 2.5 mL min^{-1}, 40 °C) t_R = 9.5(minor), 10.8 (major) minutes **$[\alpha]_D^{25.0}$** = +22.8 (c 1.0, $CHCl_3$).

Spectra of starting materials

^{1}H NMR (400 MHz, $CDCl_3$) *N*-(4-(trifluoromethyl)phenethyl)acetamide

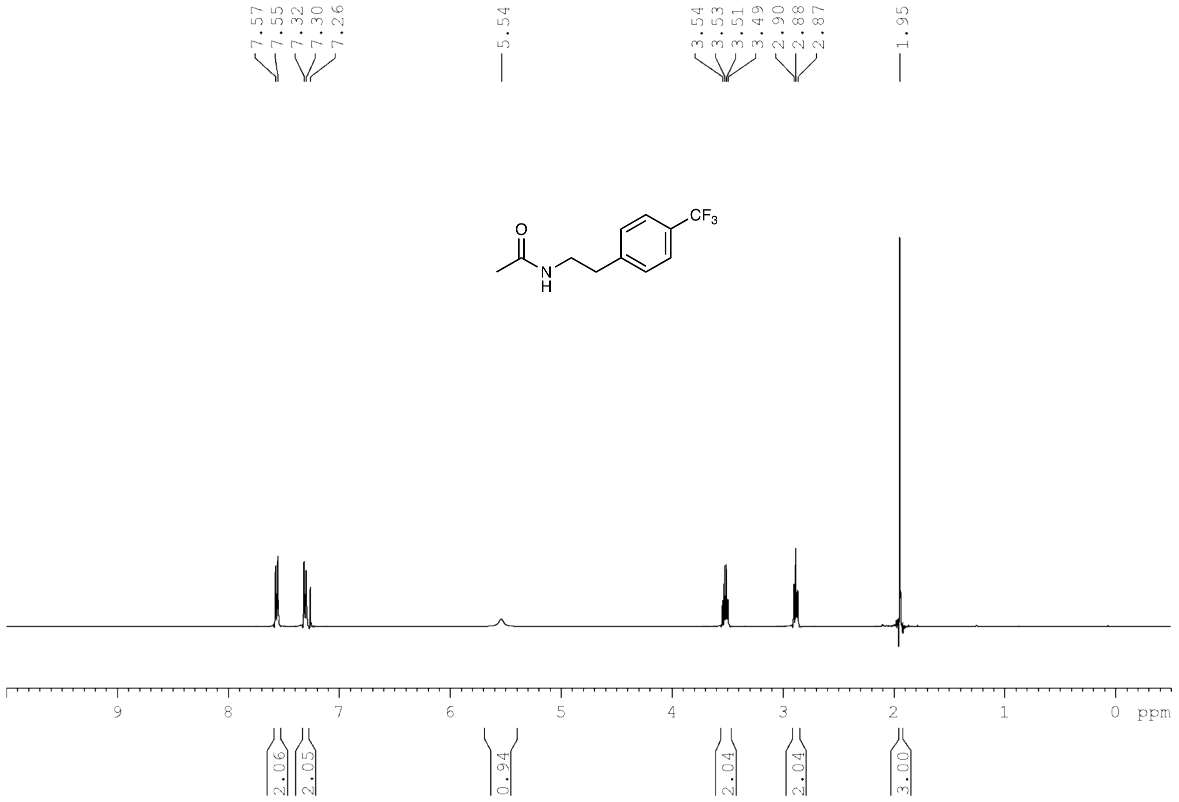

^{13}C NMR (101 MHz, $CDCl_3$) *N*-(4-(trifluoromethyl)phenethyl)acetamide

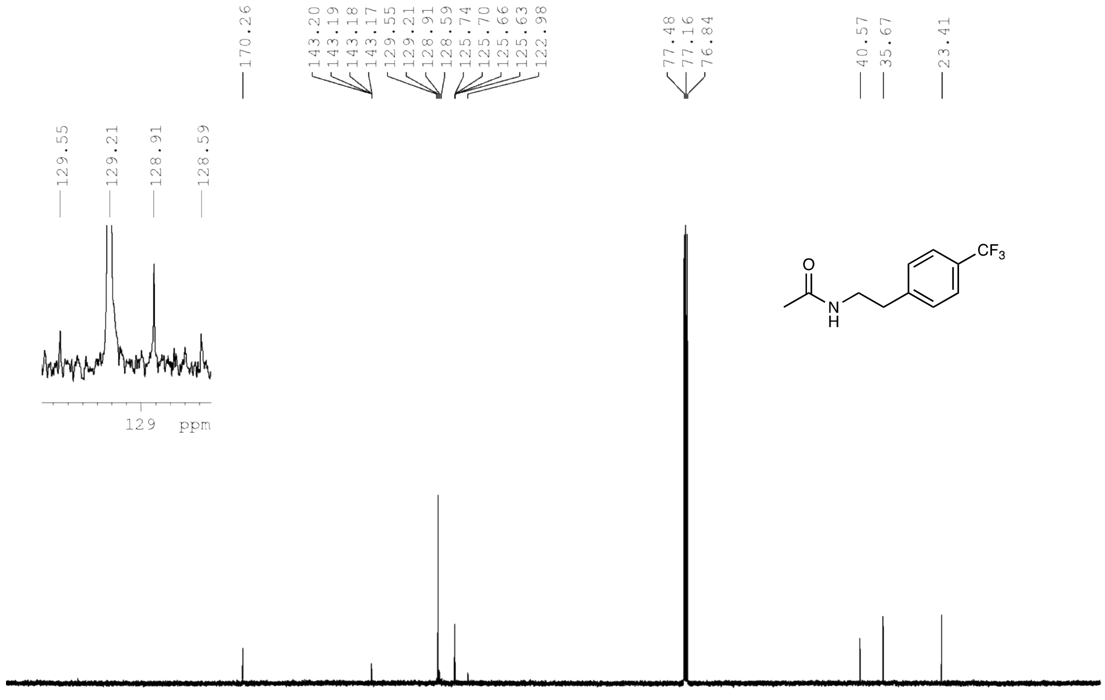

^{19}F NMR (377 MHz, $CDCl_3$) *N*-(4-(trifluoromethyl)phenethyl)acetamide

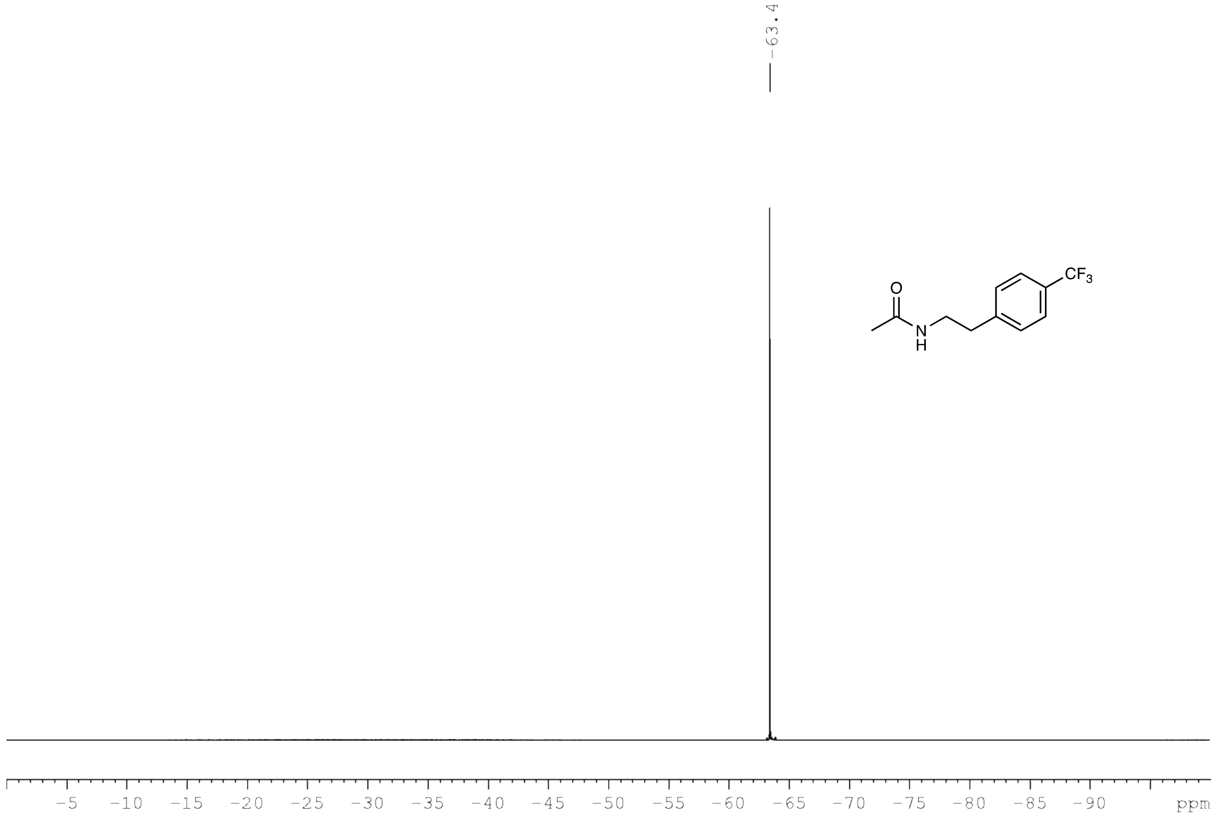

^{1}H NMR (400 MHz, $CDCl_3$) methyl *N*-acetyl-*N*-(tert-butoxycarbonyl)-L-lysinate

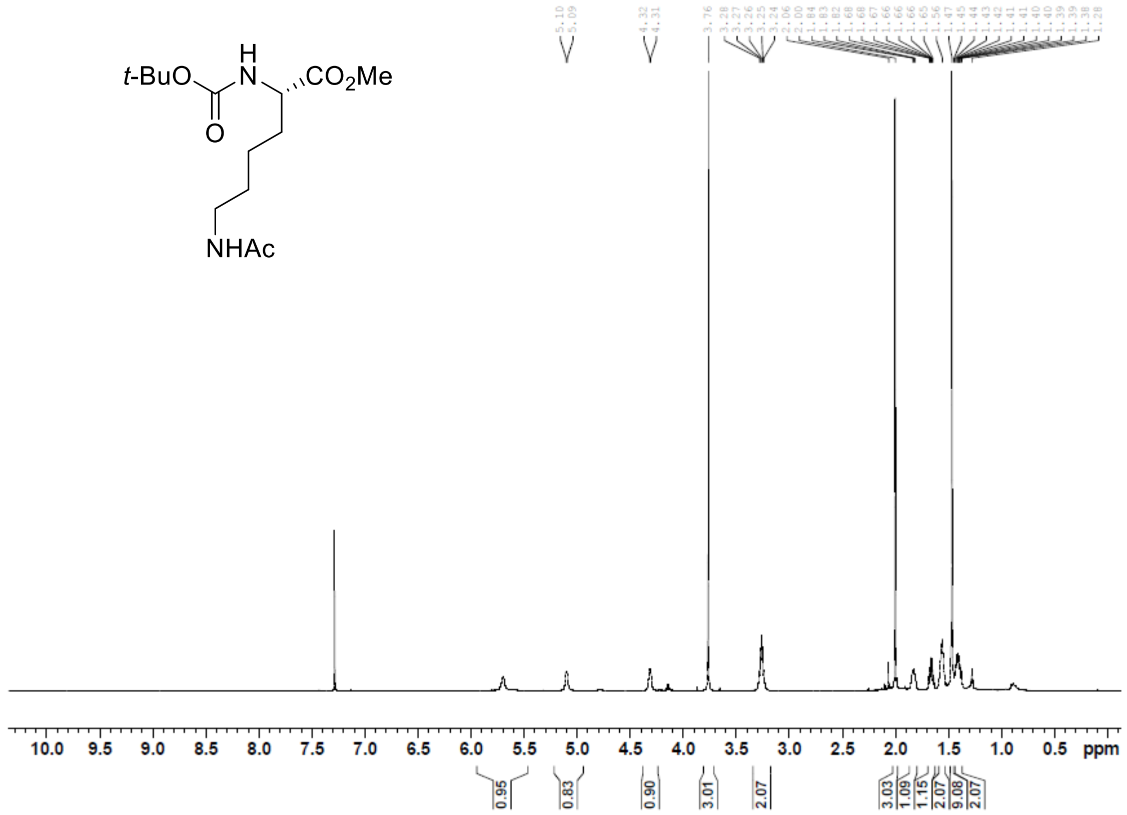

^{13}C NMR (101 MHz, $CDCl_3$) methyl *N*-acetyl-*N*-(tert-butoxycarbonyl)-L-lysinate

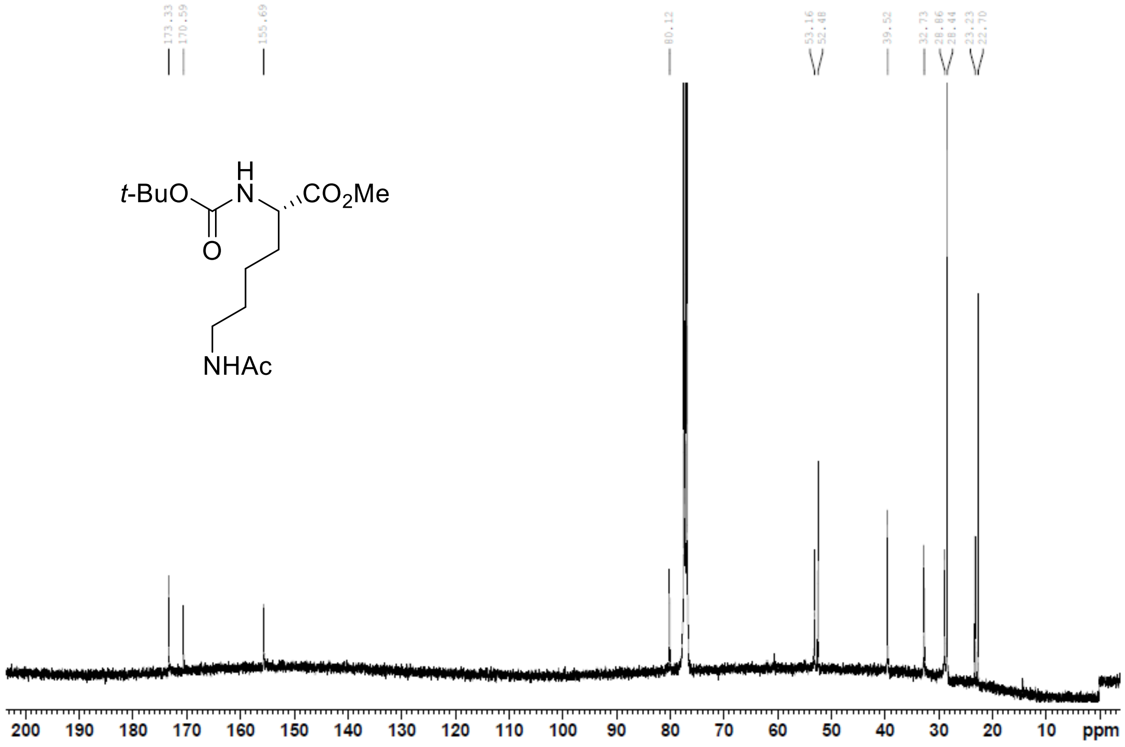

Spectra of products

^{1}H NMR (400 MHz, $CDCl_3$) (*S*)-*N*-(1-(4-methylquinolin-2-yl)-2-phenylethyl)acetamide (3)

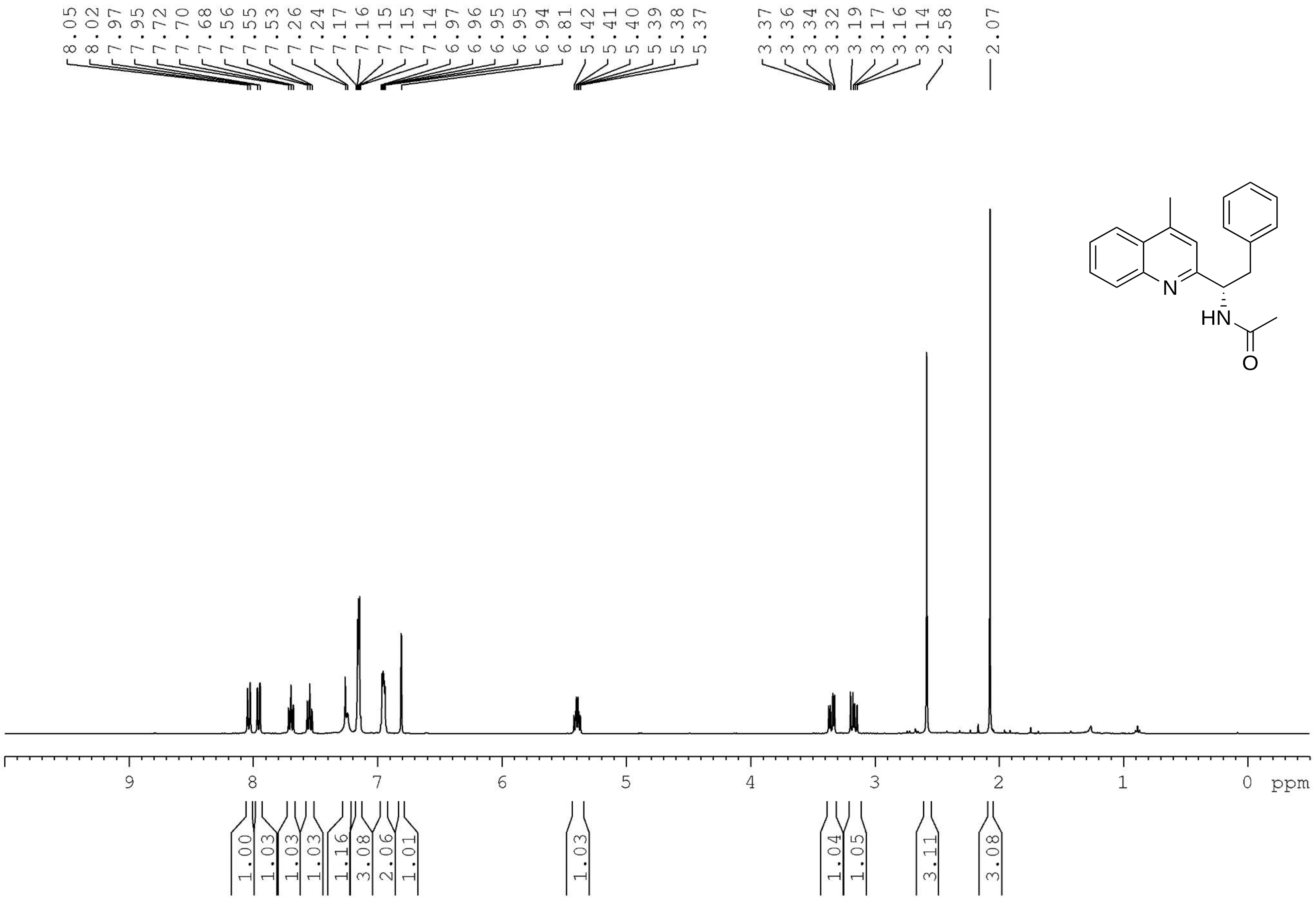

^{13}C NMR (101 MHz, CDCl$_3$) (*S*)-*N*-(1-(4-methylquinolin-2-yl)-2-phenylethyl)acetamide (3)

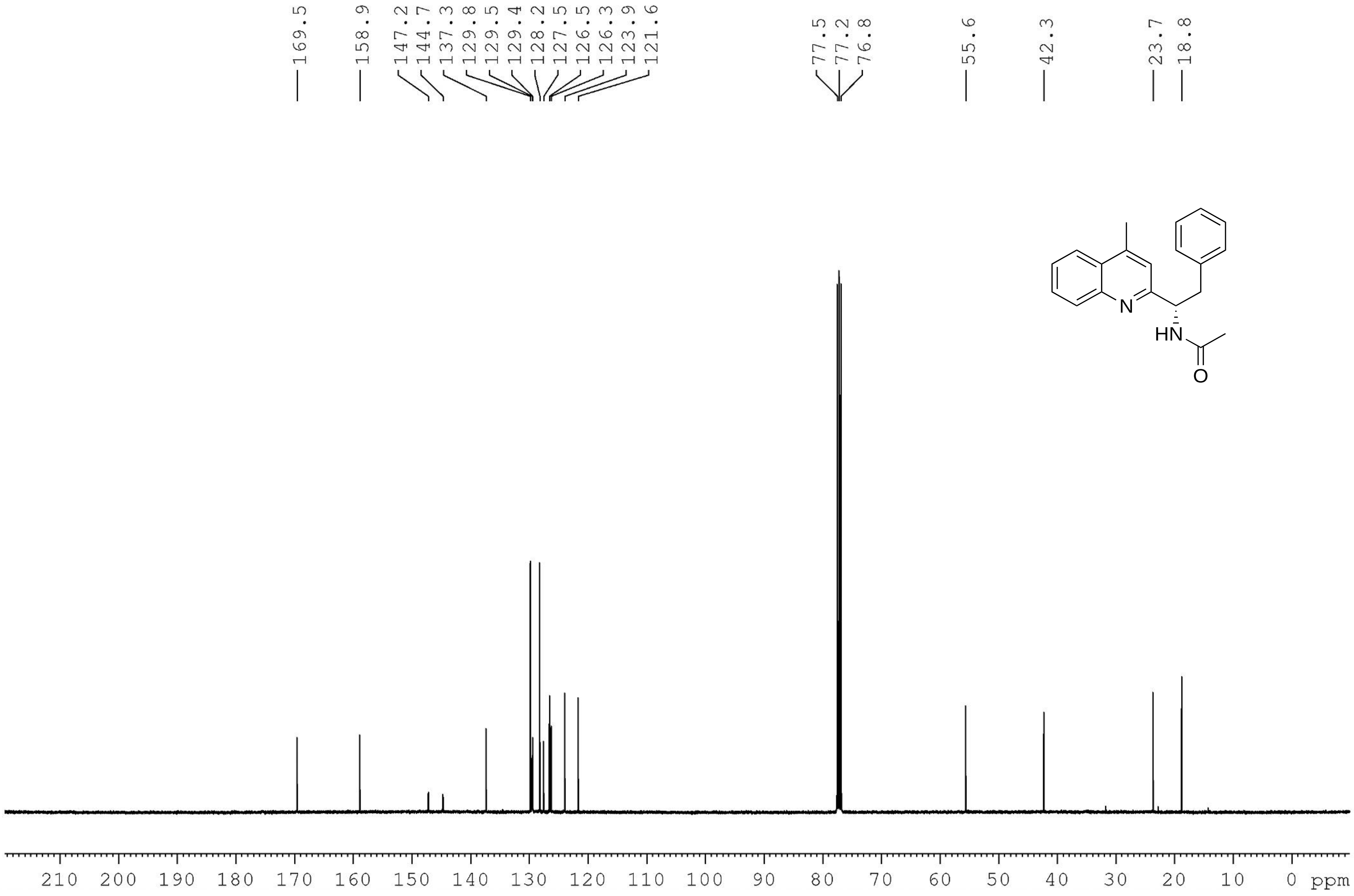

^{1}H NMR (400 MHz, $CDCl_3$) (*S*)-*N*-(2-(4-fluorophenyl)-1-(4-methylquinolin-2-yl)ethyl)acetamide (4)

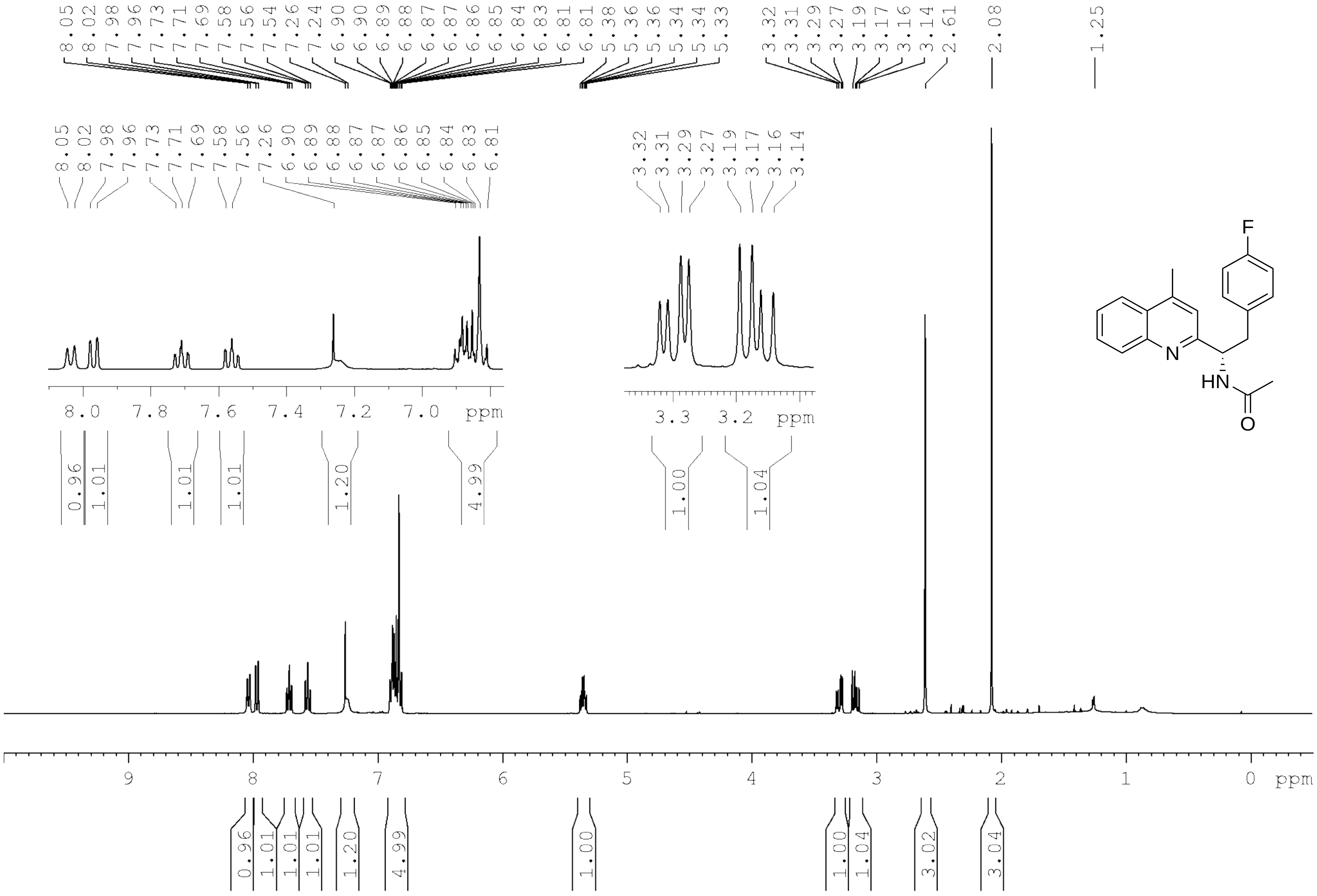

^{13}C NMR (101 MHz, CDCl$_3$) (*S*)-*N*-(2-(4-fluorophenyl)-1-(4-methylquinolin-2-yl)ethyl)acetamide (4)

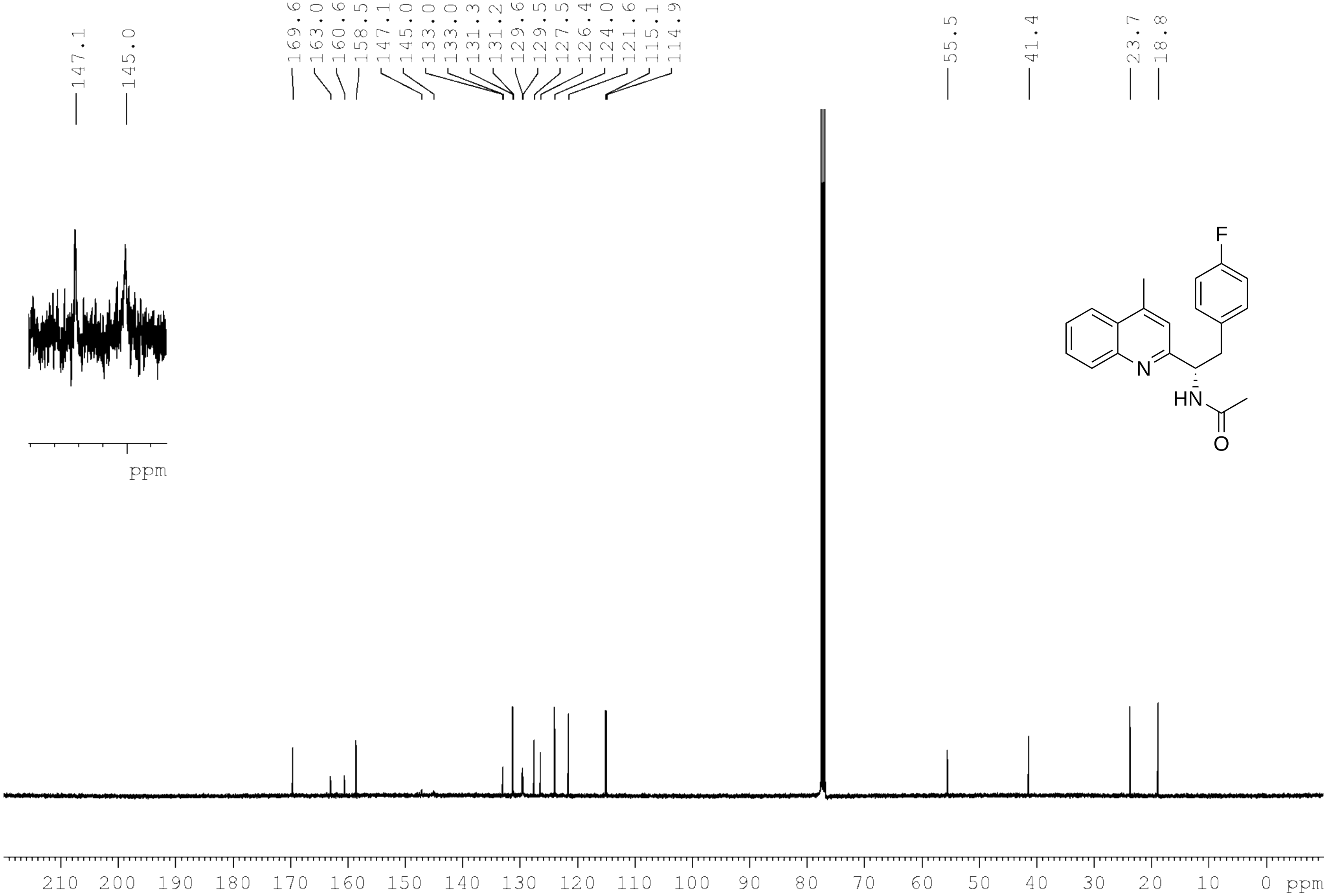

^{1}H NMR (400 MHz, $CDCl_3$) (*S*)-*N*-(1-(4-methylquinolin-2-yl)-2-(4-(trifluoromethyl)phenyl)ethyl)acetamide (5)

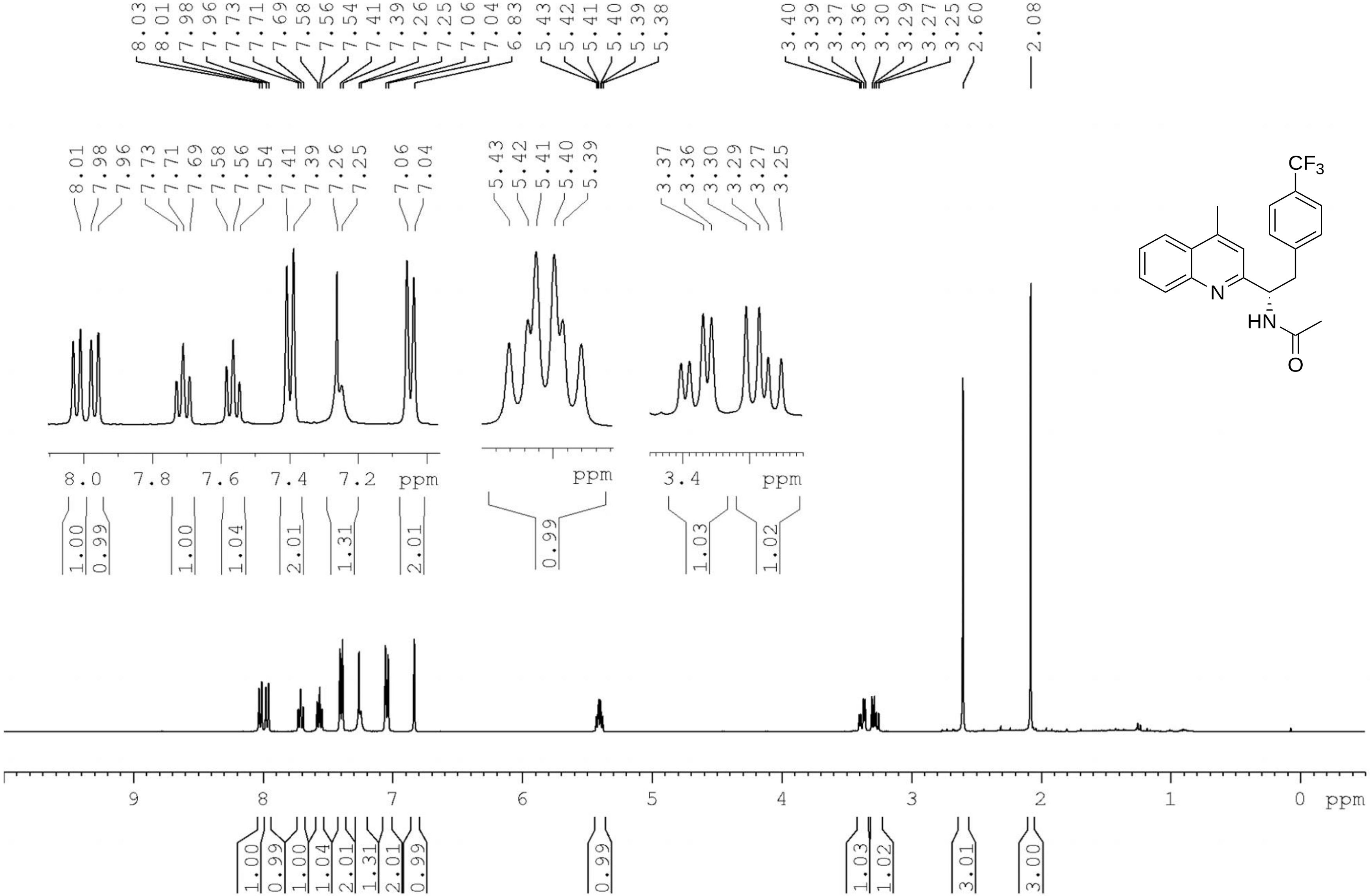

^{13}C NMR (101 MHz, CDCl$_3$) (*S*)-*N*-(1-(4-methylquinolin-2-yl)-2-(4-(trifluoromethyl)phenyl)ethyl)acetamide (5)

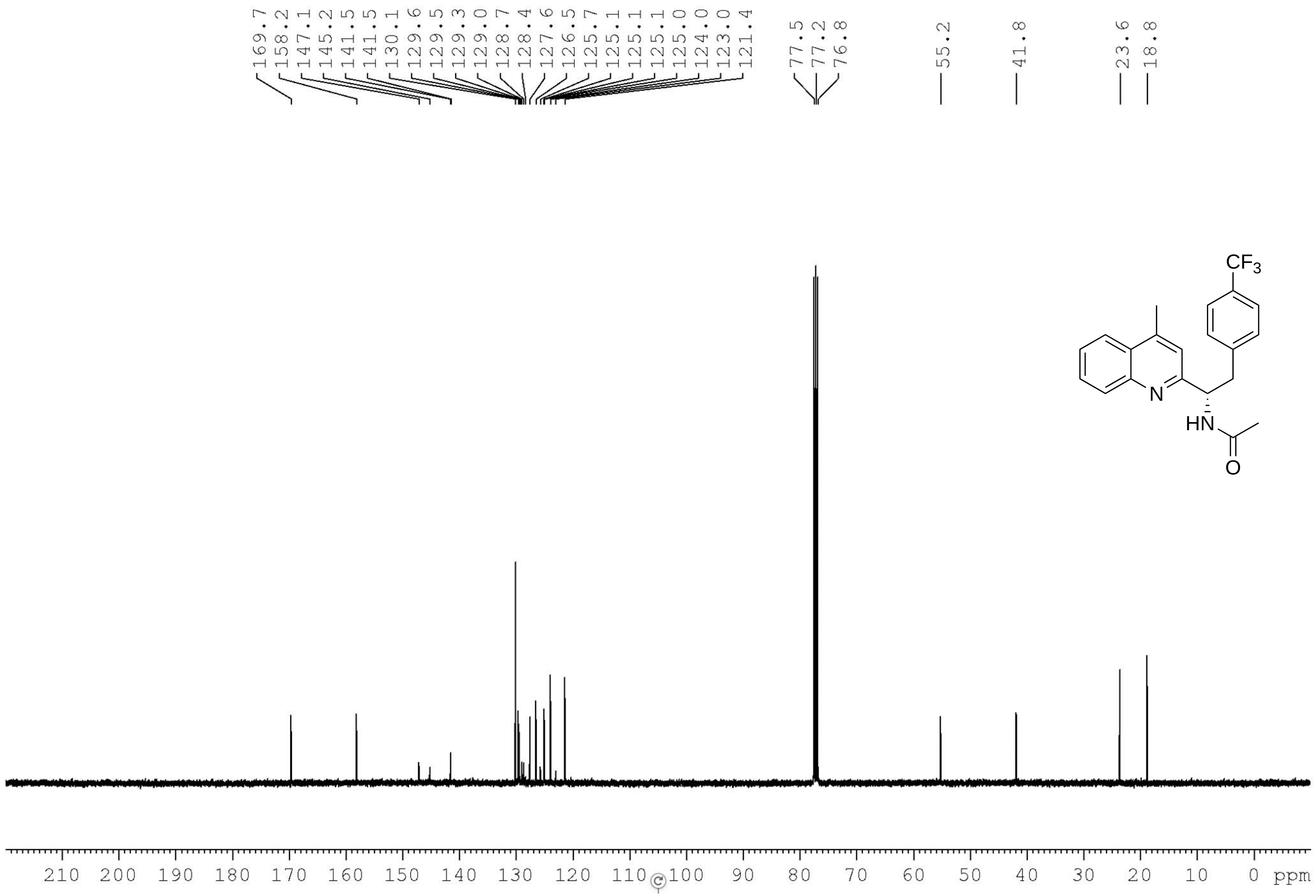

^1H NMR (400 MHz, $CDCl_3$) (S)-N-(2-(3-bromophenyl)-1-(4-methylquinolin-2-yl)ethyl)acetamide (7)

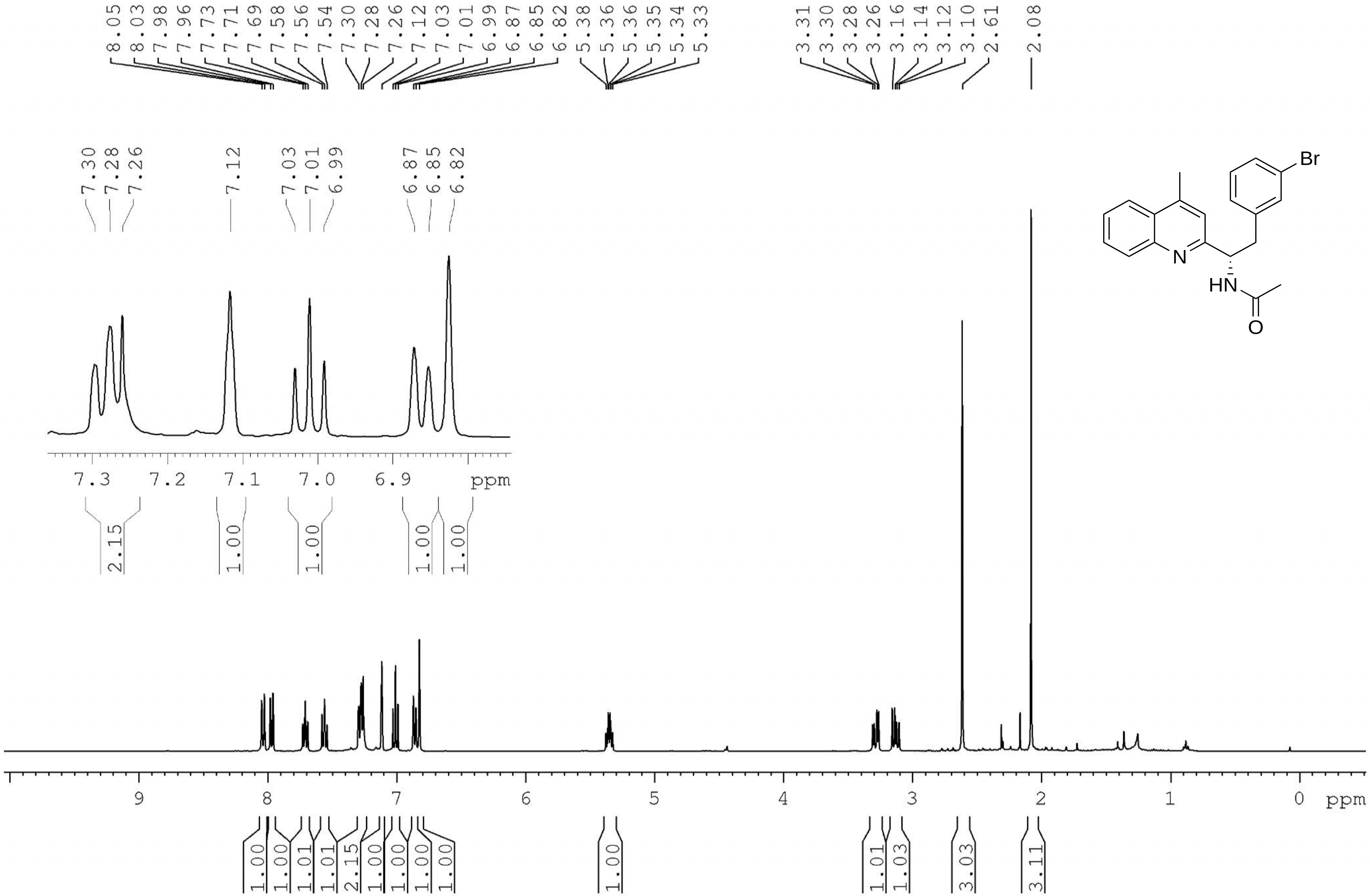

^{13}C NMR (101 MHz, $CDCl_3$) (S)-N-(2-(3-bromophenyl)-1-(4-methylquinolin-2-yl)ethyl)acetamide (7)

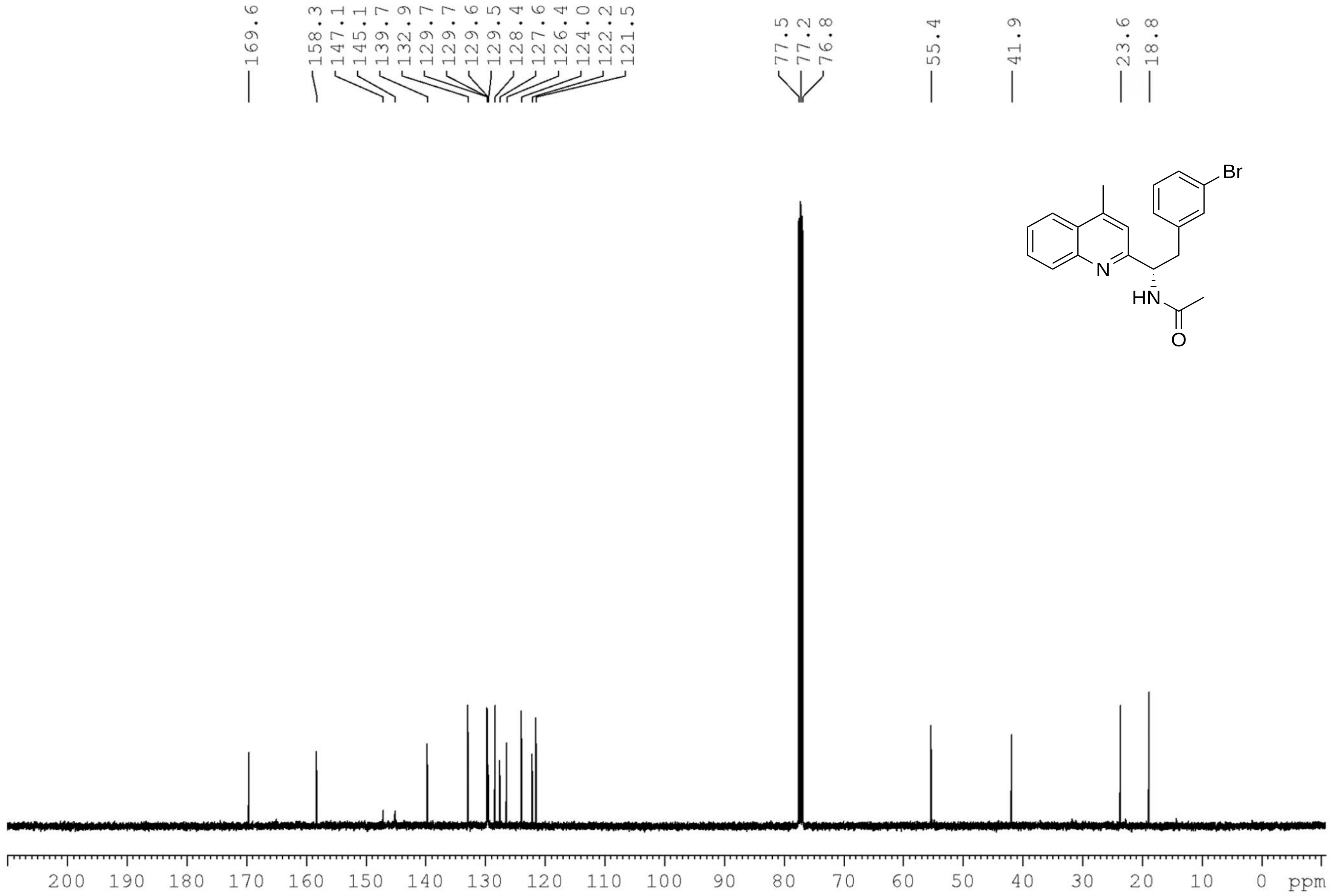

^{1}H NMR (400 MHz, $CDCl_3$) (S)-N-(2-(3,4-dichlorophenyl)-1-(4-methylquinolin-2-yl)ethyl)acetamide (8)

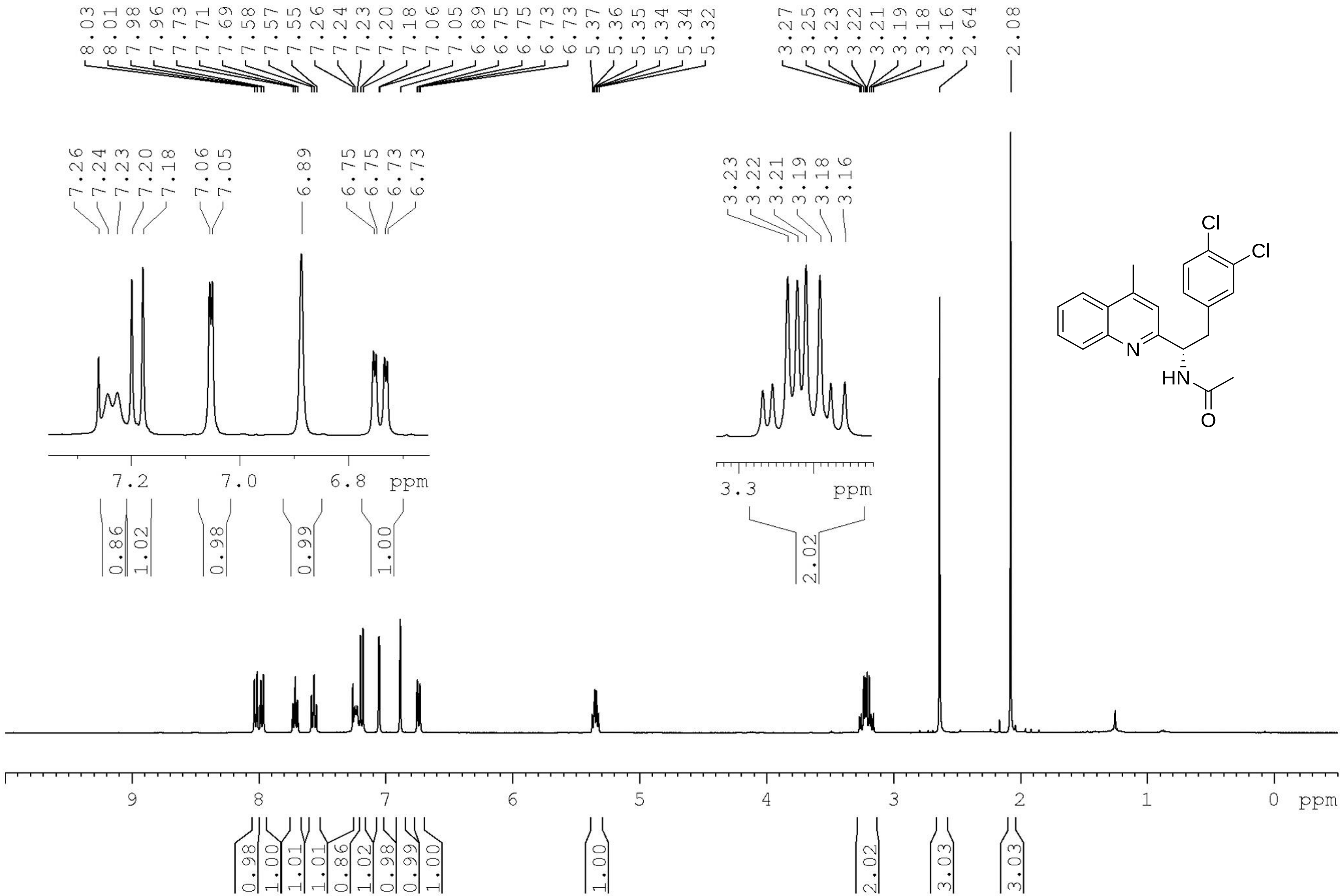

^{13}C NMR (101 MHz, $CDCl_3$) (S)-N-(2-(3,4-dichlorophenyl)-1-(4-methylquinolin-2-yl)ethyl)acetamide (8)

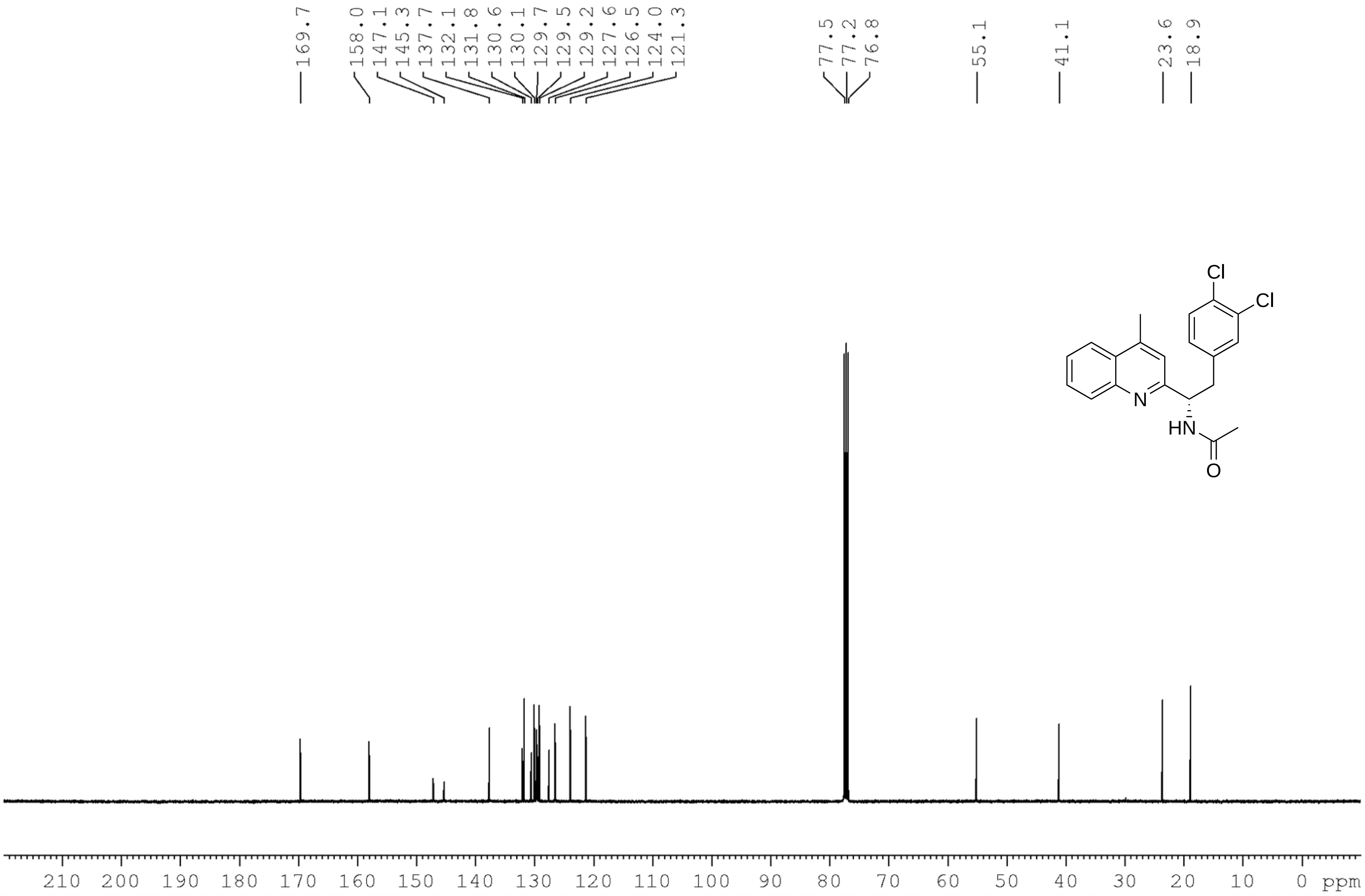

^{1}H NMR (400 MHz, $CDCl_3$) (*S*)-*N*-(1-(4-methylquinolin-2-yl)-3-phenylpropyl)acetamide (9)

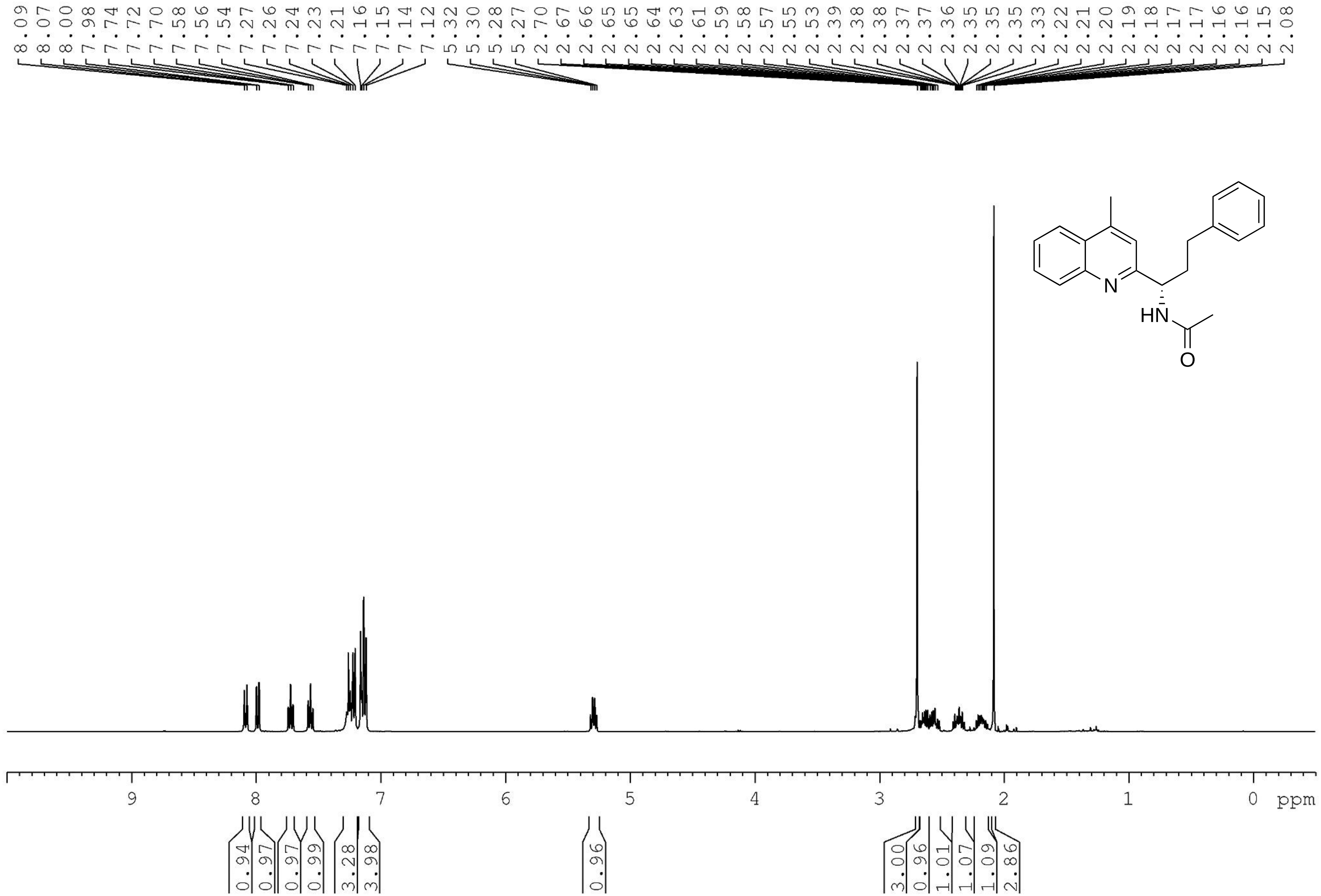

^{13}C NMR (101 MHz, $CDCl_3$) (*S*)-*N*-(1-(4-methylquinolin-2-yl)-3-phenylpropyl)acetamide (9)

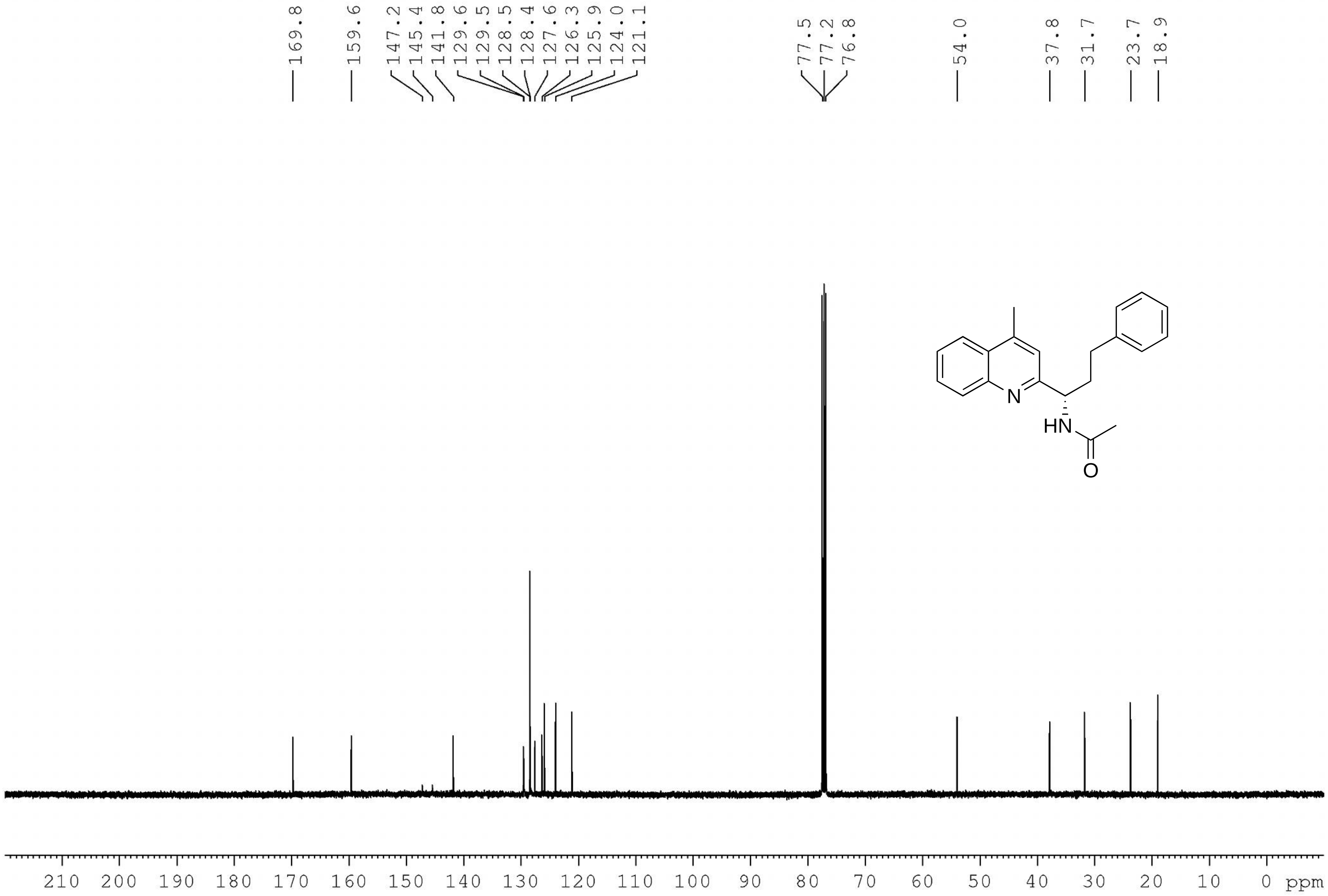

^{1}H NMR (400 MHz, $CDCl_3$) (S)-N-(1-(4-methylquinolin-2-yl)-4-phenylbutyl)acetamide (10)

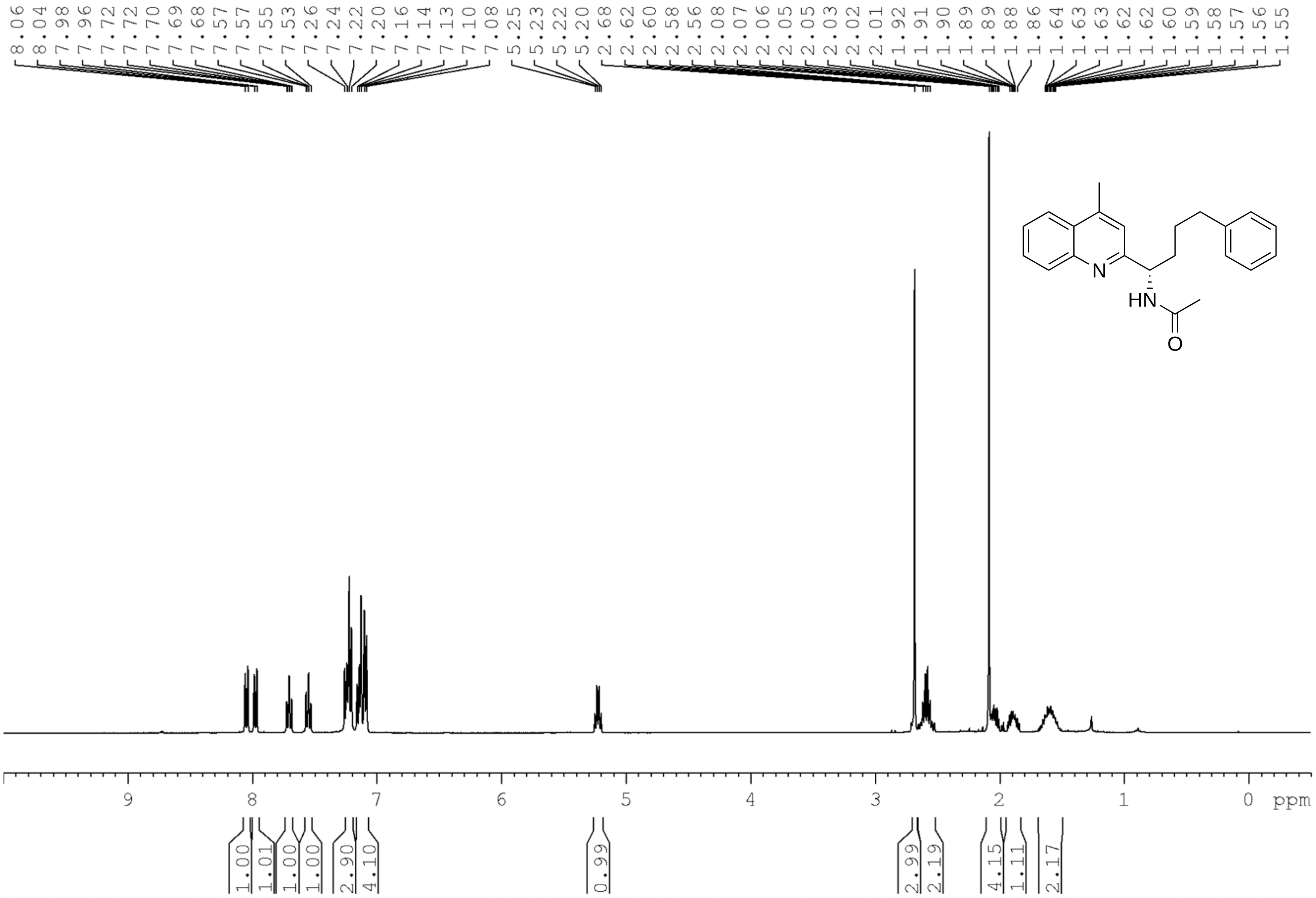

^{13}C NMR (101 MHz, CDCl$_3$) (S)-N-(1-(4-methylquinolin-2-yl)-4-phenylbutyl)acetamide (10)

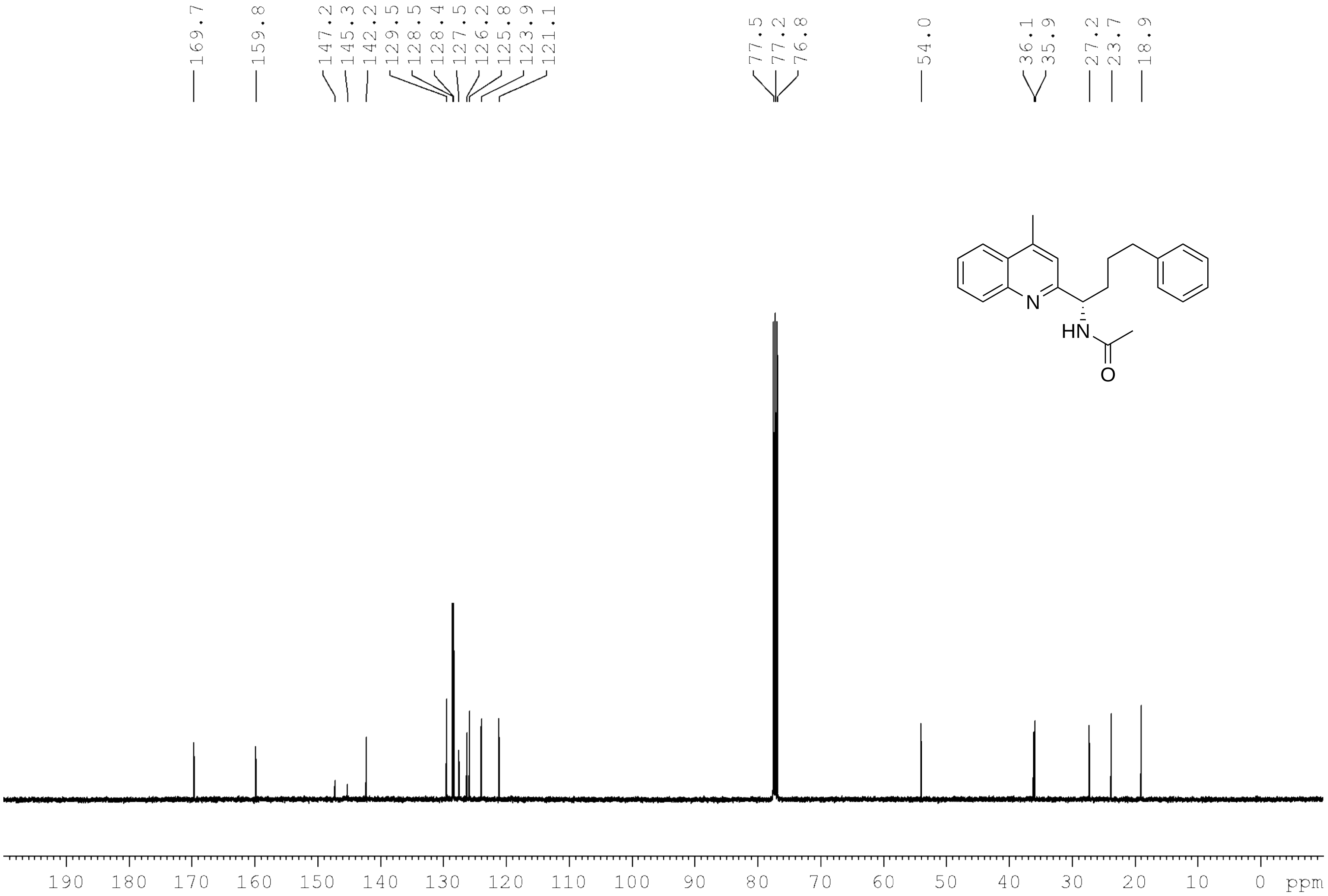

^{1}H NMR (400 MHz, $CDCl_3$) (*S*)-*N*-(1-(4-methylquinolin-2-yl)ethyl)acetamide (11)

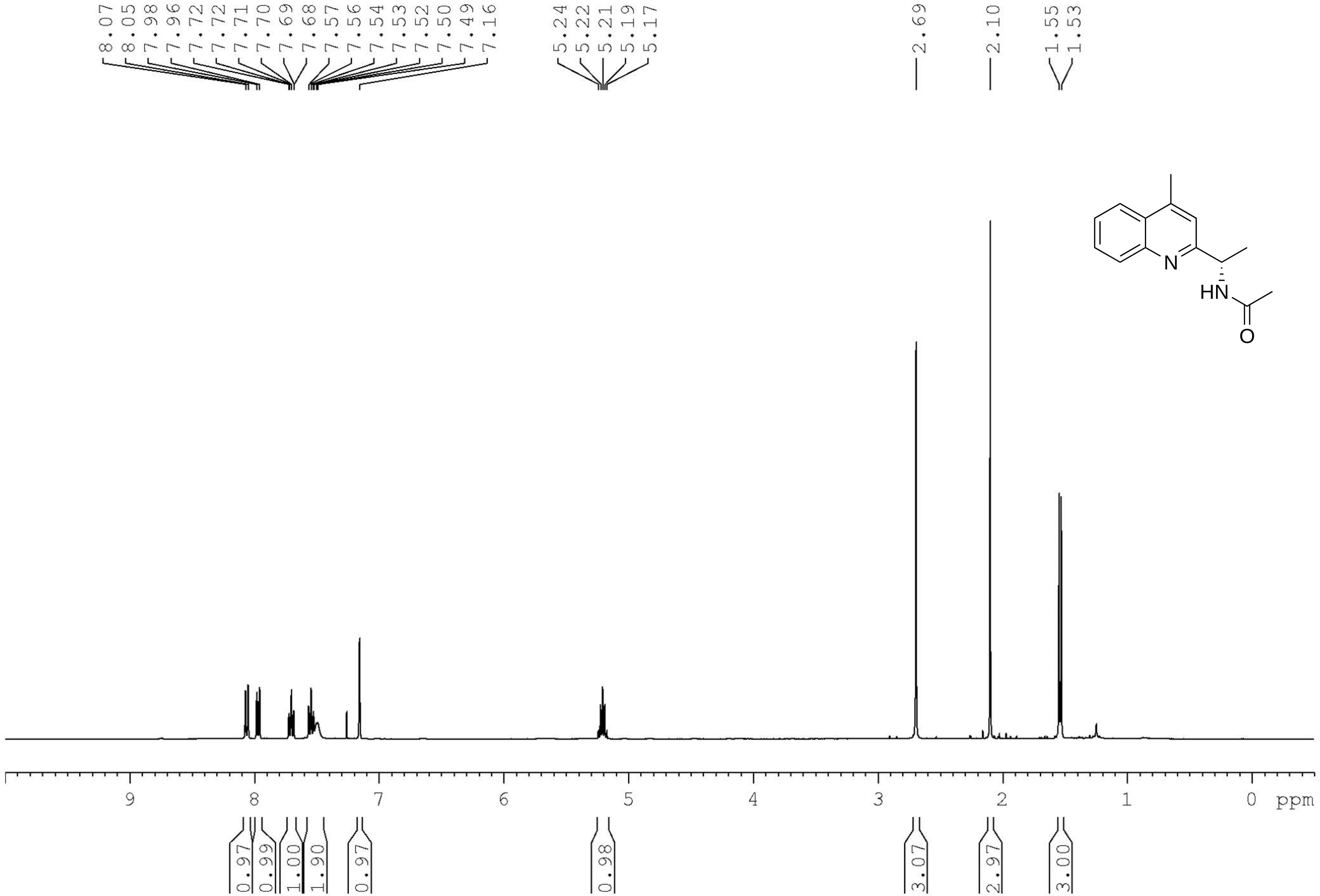

^{13}C NMR (101 MHz, CDCl$_3$) (*S*)-*N*-(1-(4-methylquinolin-2-yl)ethyl)acetamide (11)

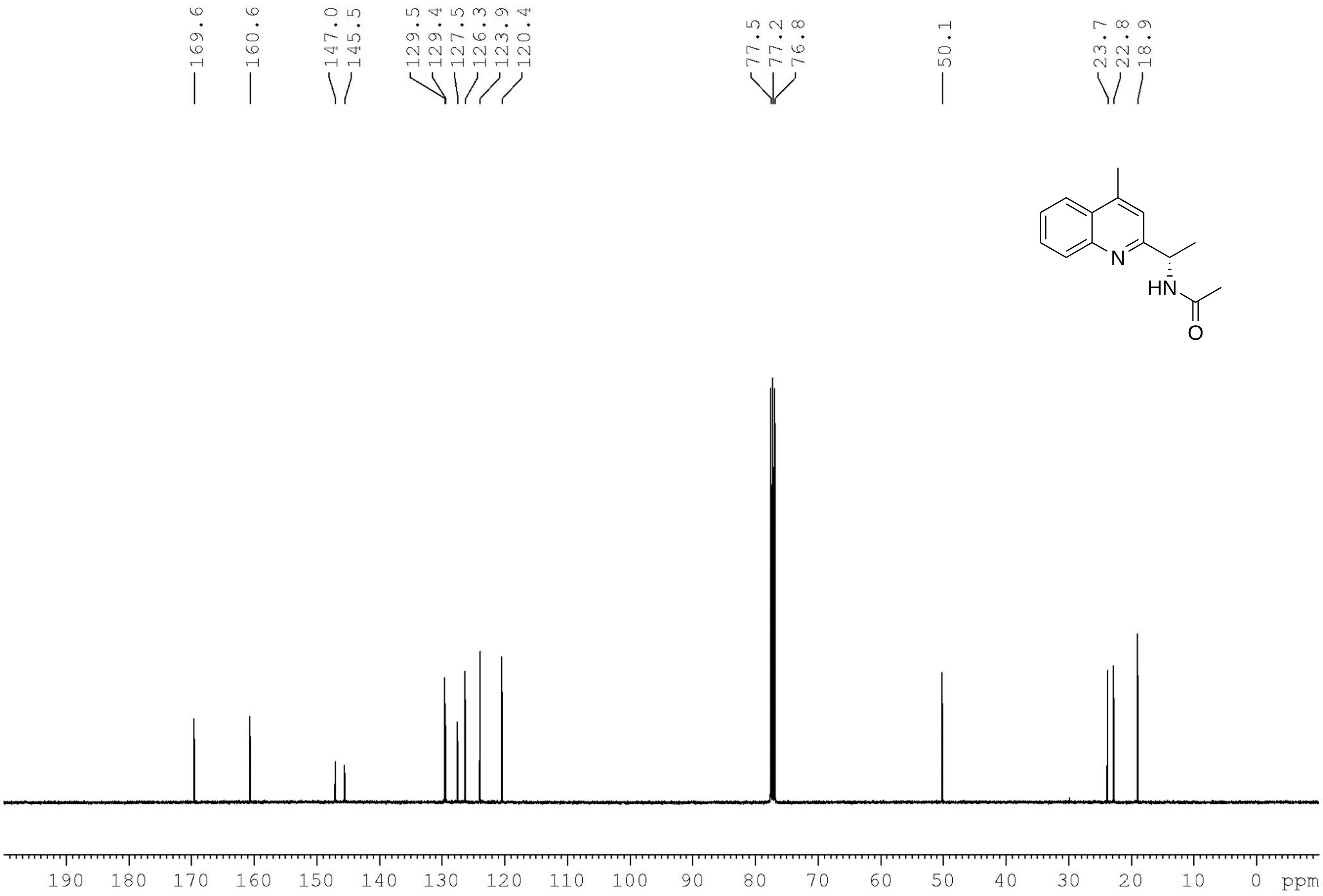

^{1}H NMR (400 MHz, CDCl$_3$) (*S*)-*N*-(1-(4-methylquinolin-2-yl)nonyl)acetamide (12)

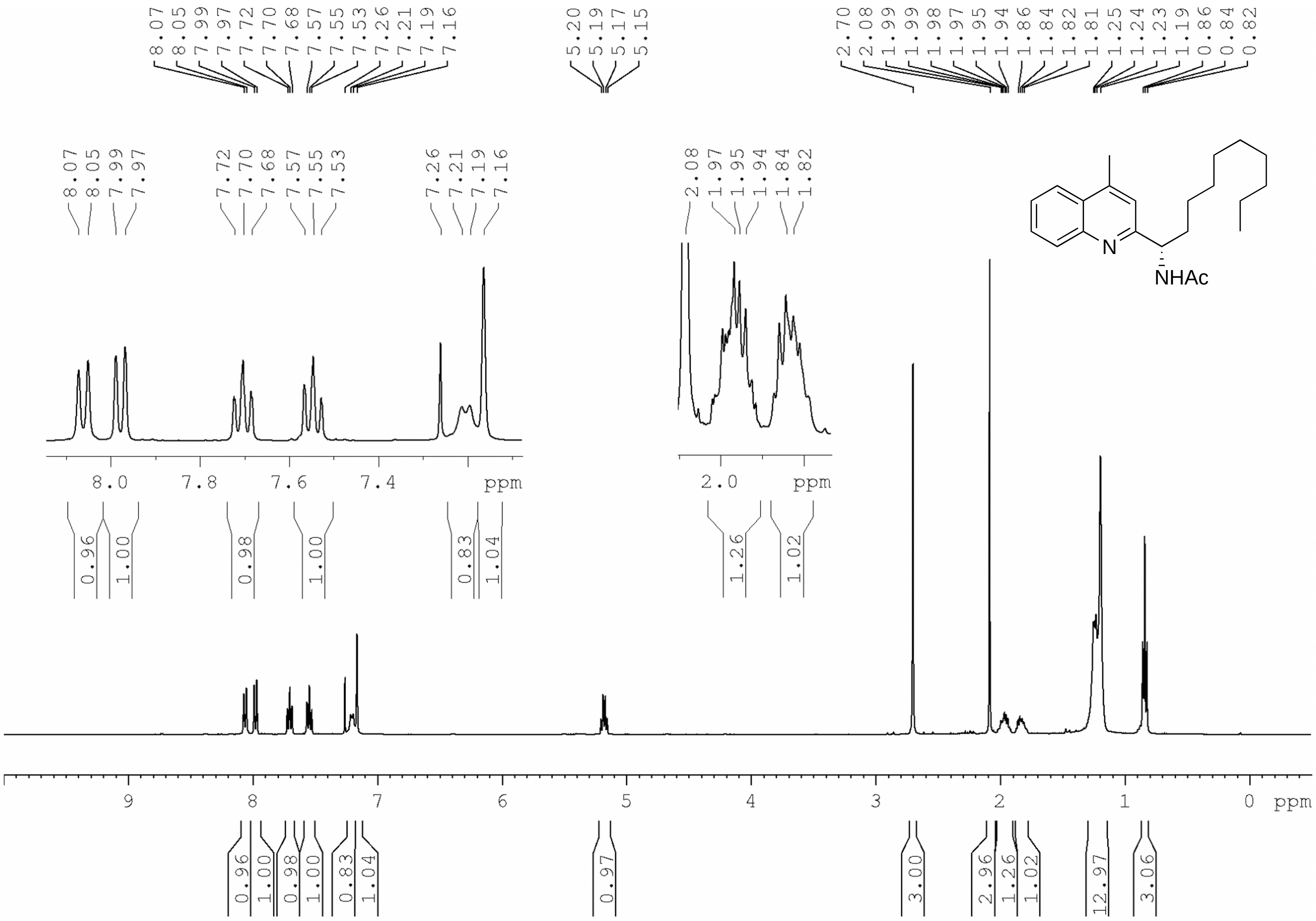

^{13}C NMR (101 MHz, $CDCl_3$) (*S*)-*N*-(1-(4-methylquinolin-2-yl)nonyl)acetamide (12)

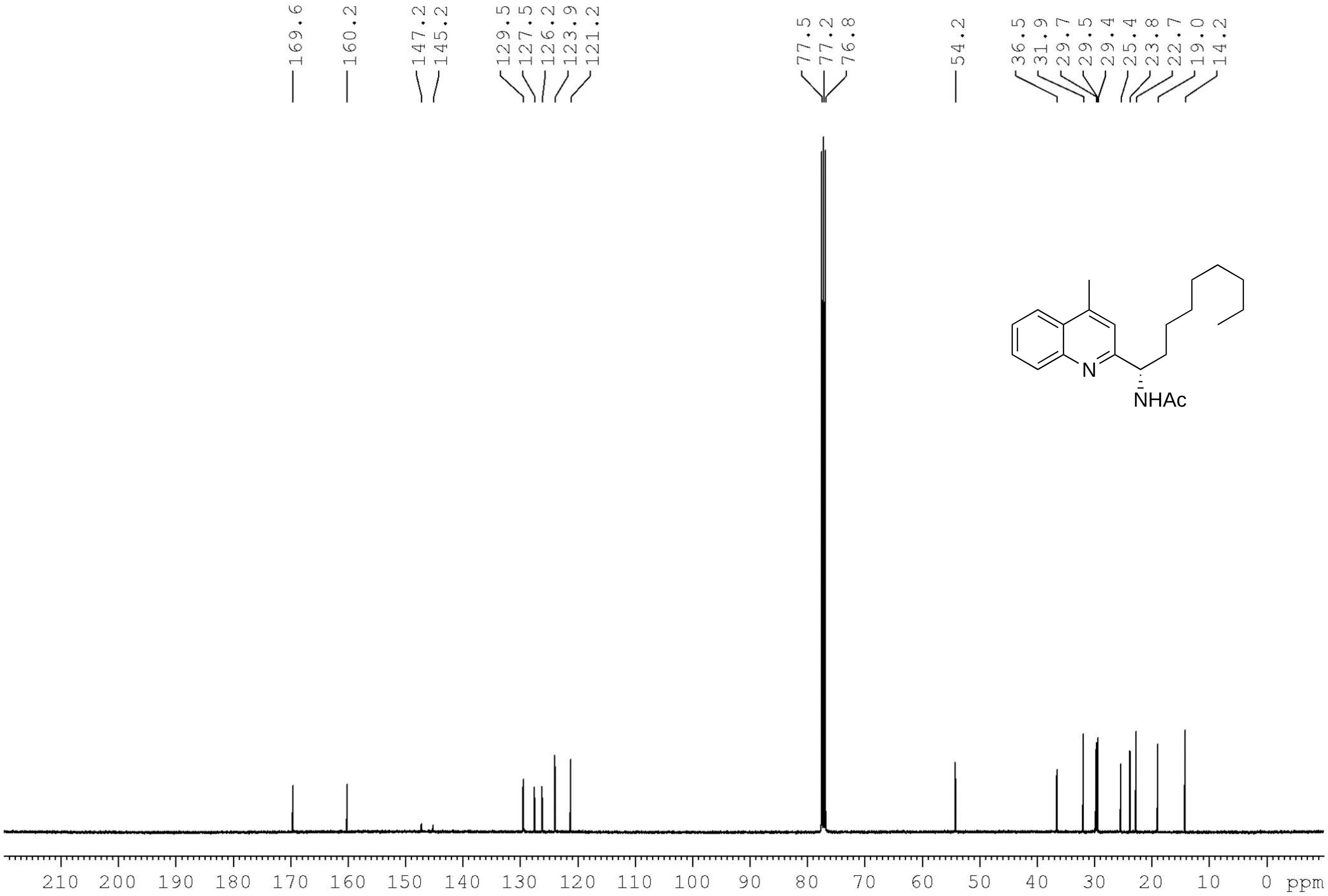

^{1}H NMR (400 MHz, $CDCl_3$) (*S*)-*N*-(2-methyl-1-(4-methylquinolin-2-yl)propyl)acetamide (13)

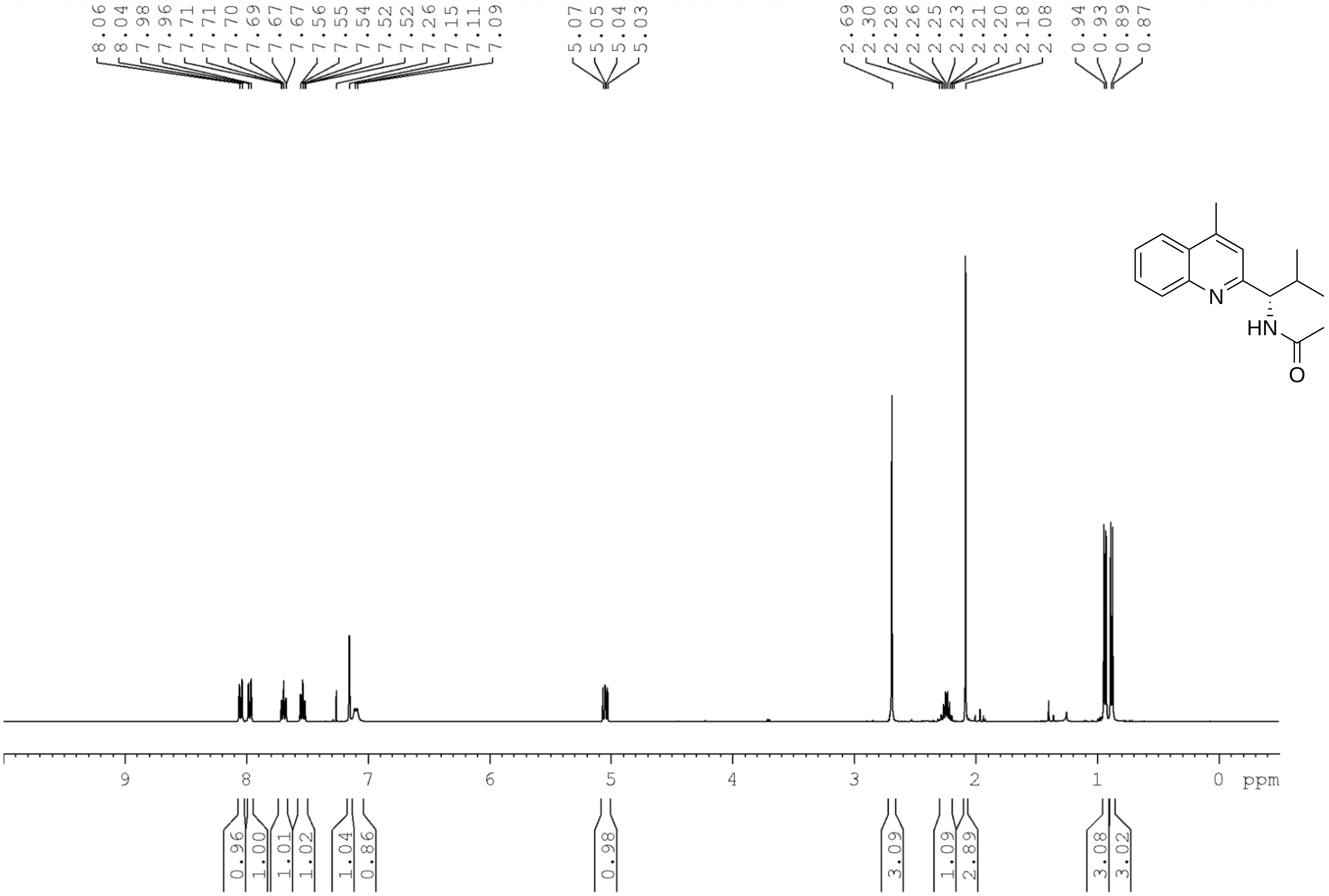

^{13}C NMR (101 MHz, $CDCl_3$) (*S*)-*N*-(2-methyl-1-(4-methylquinolin-2-yl)propyl)acetamide (13)

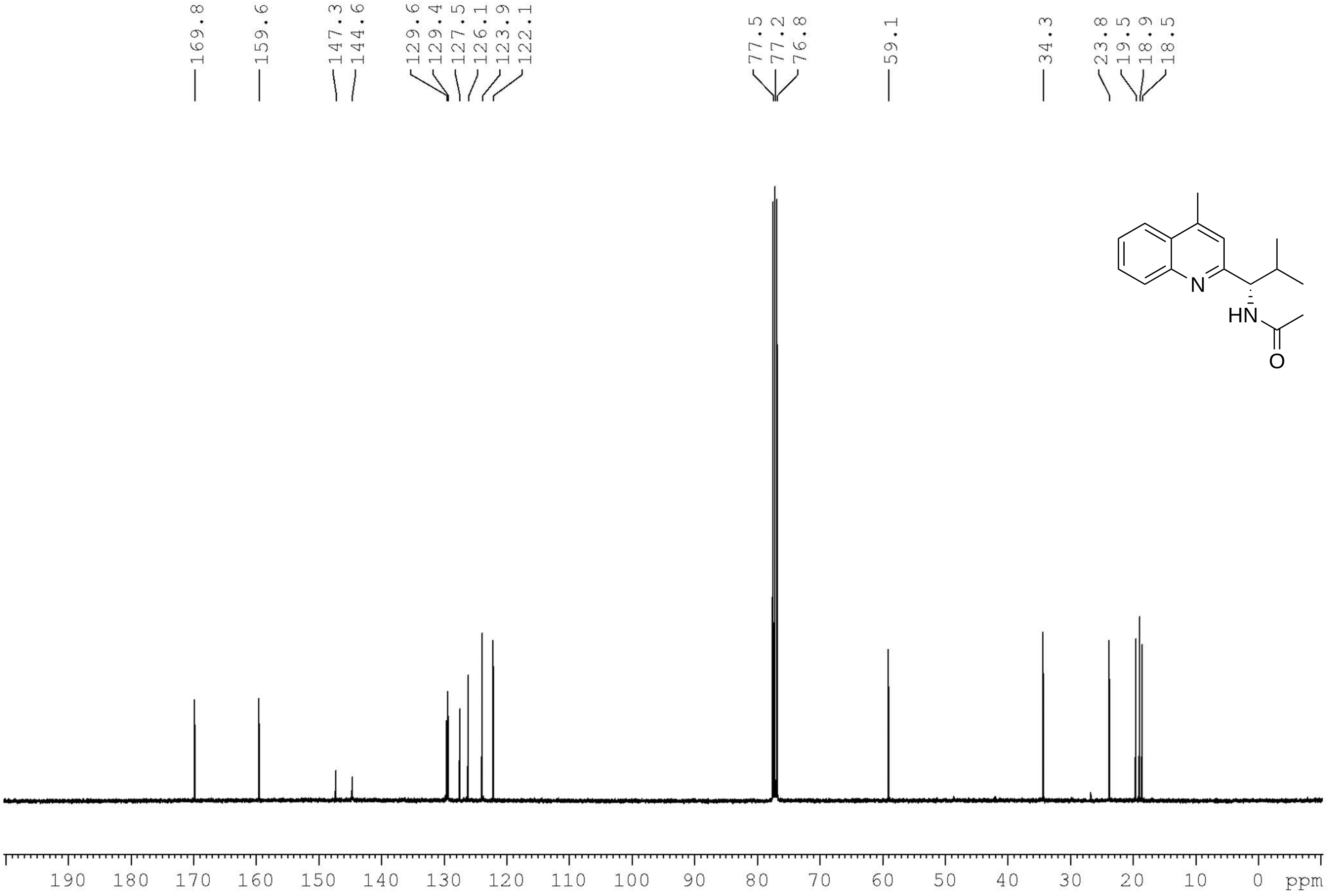

^{1}H NMR (400 MHz, $CDCl_3$) *tert*-butyl (*S*)-4-acetamido-4-(4-methylquinolin-2-yl)butanoate (15)

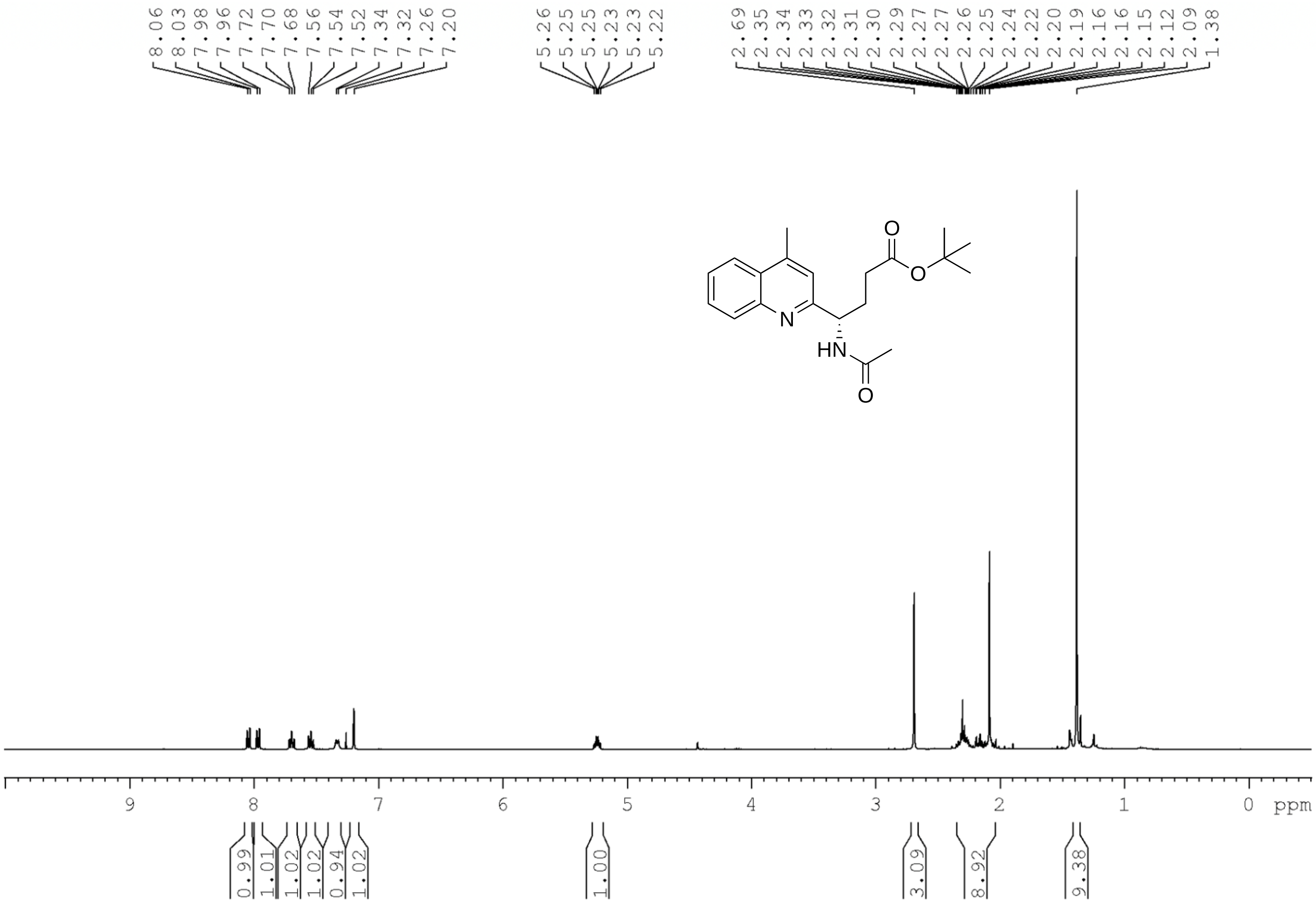

^{13}C NMR (101 MHz, $CDCl_3$) *tert*-butyl (*S*)-4-acetamido-4-(4-methylquinolin-2-yl)butanoate (15)

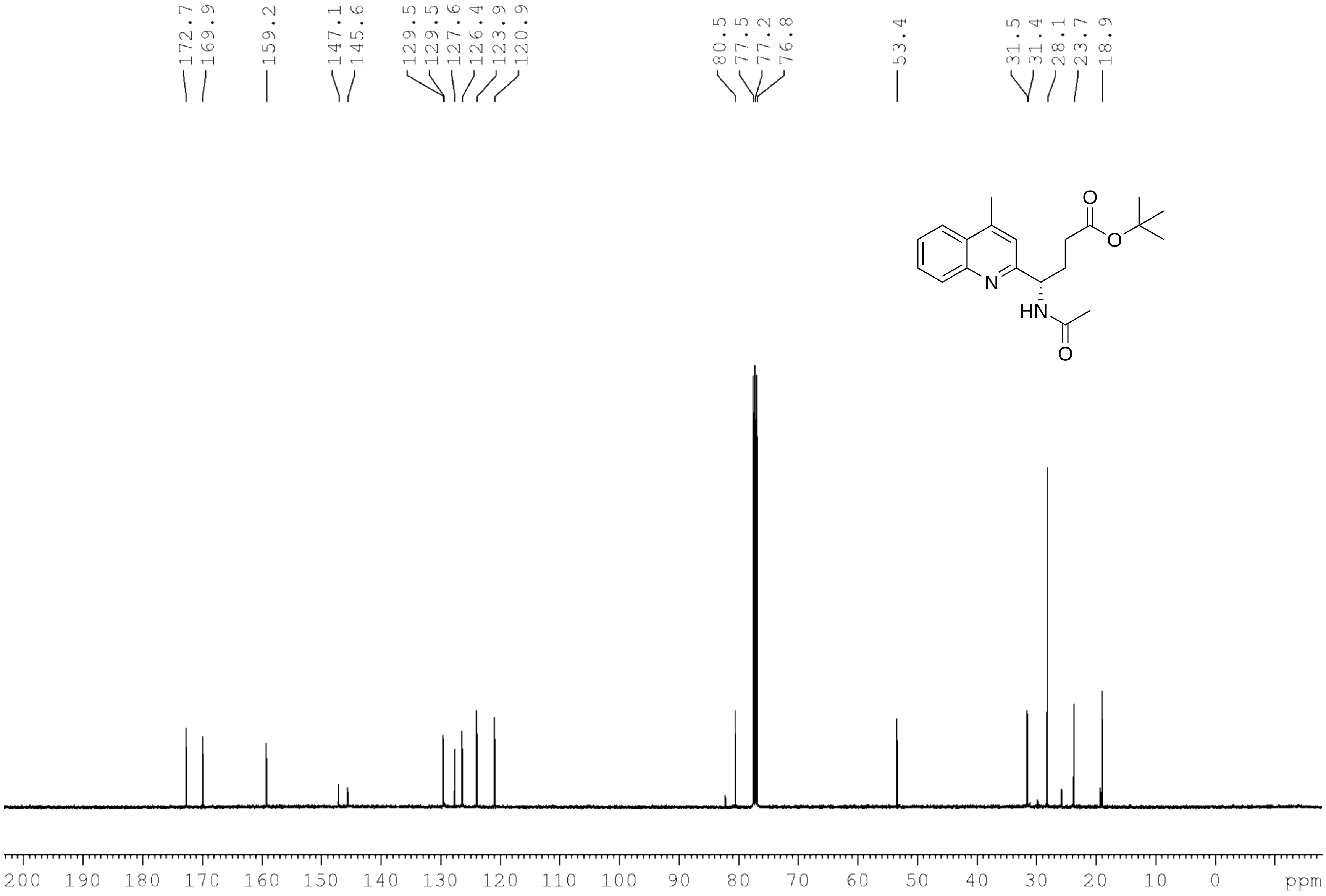

^{1}H NMR (400 MHz, $CDCl_3$) methyl (2*S*,6*S*)-6-acetamido-2-((*t*-butoxycarbonyl)amino)-6-(4-methylquinolin-2-yl)hexanoate (16)

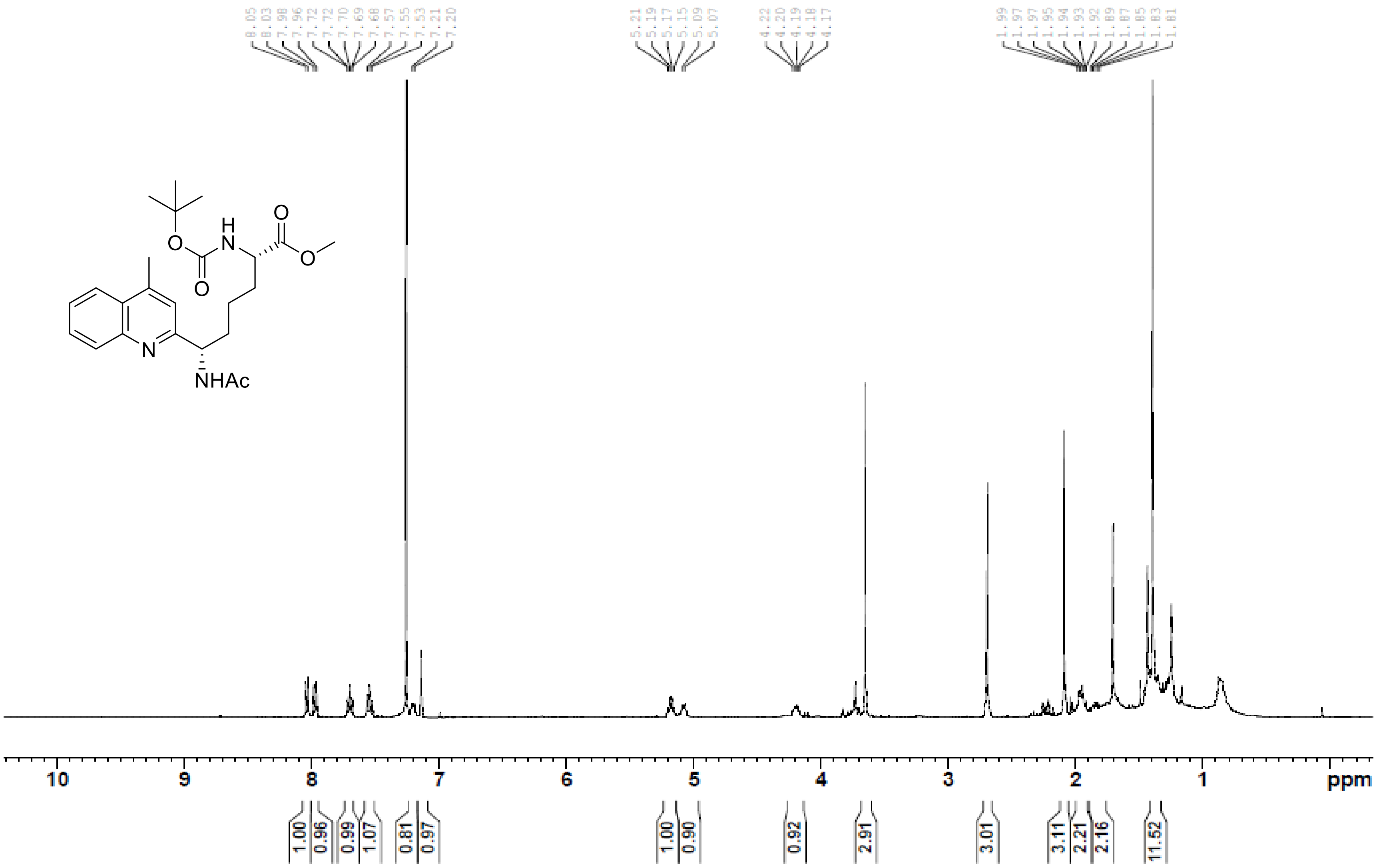

^{13}C NMR (101 MHz, CDCl$_3$) methyl (2*S*,6*S*)-6-acetamido-2-((*t*-butoxycarbonyl)amino)-6-(4-methylquinolin-2-yl)hexanoate (16)

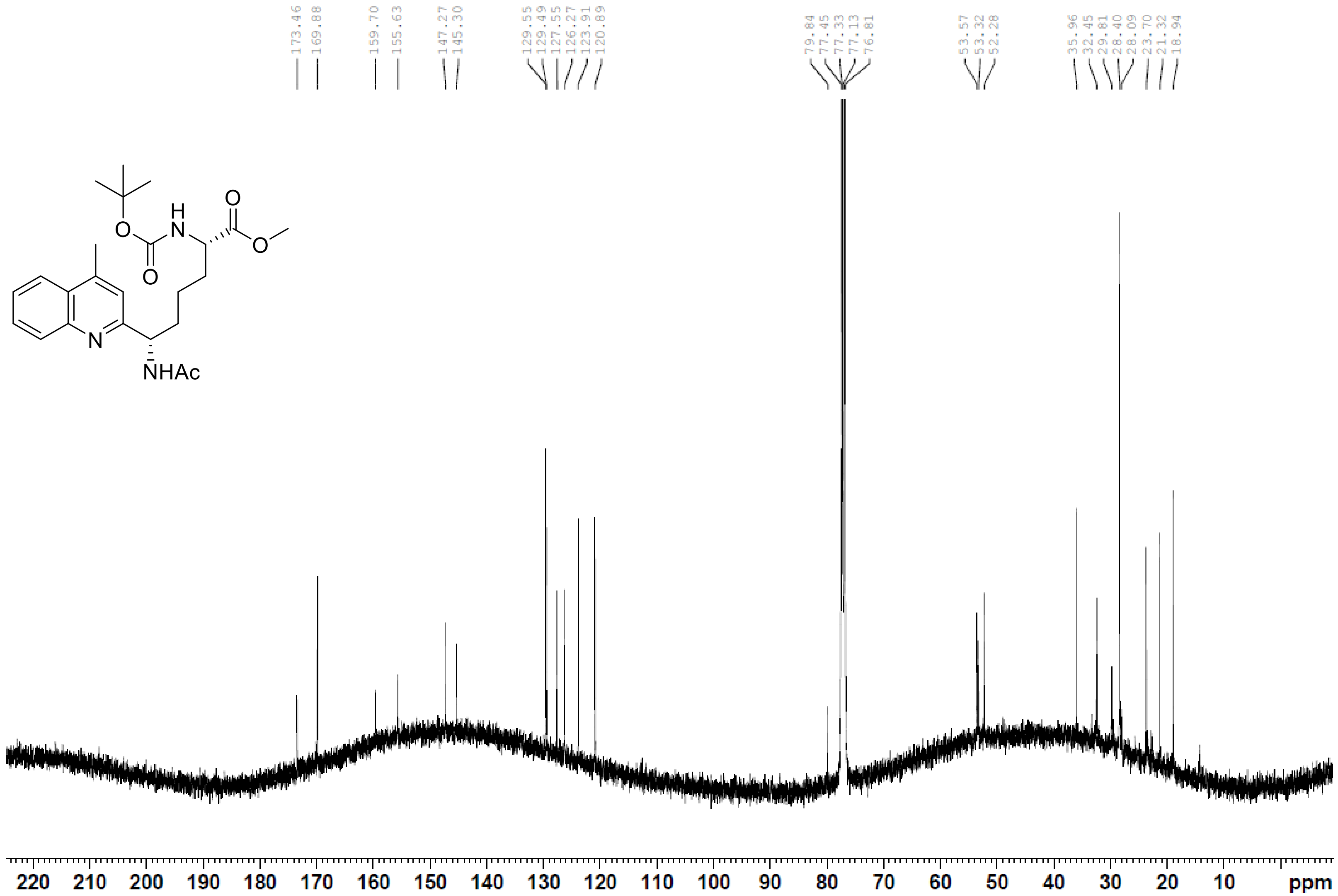

^{1}H NMR (400 MHz, $CDCl_3$) (S)-*N*-(2-phenyl-1-(quinolin-2-yl)ethyl)acetamide (17)

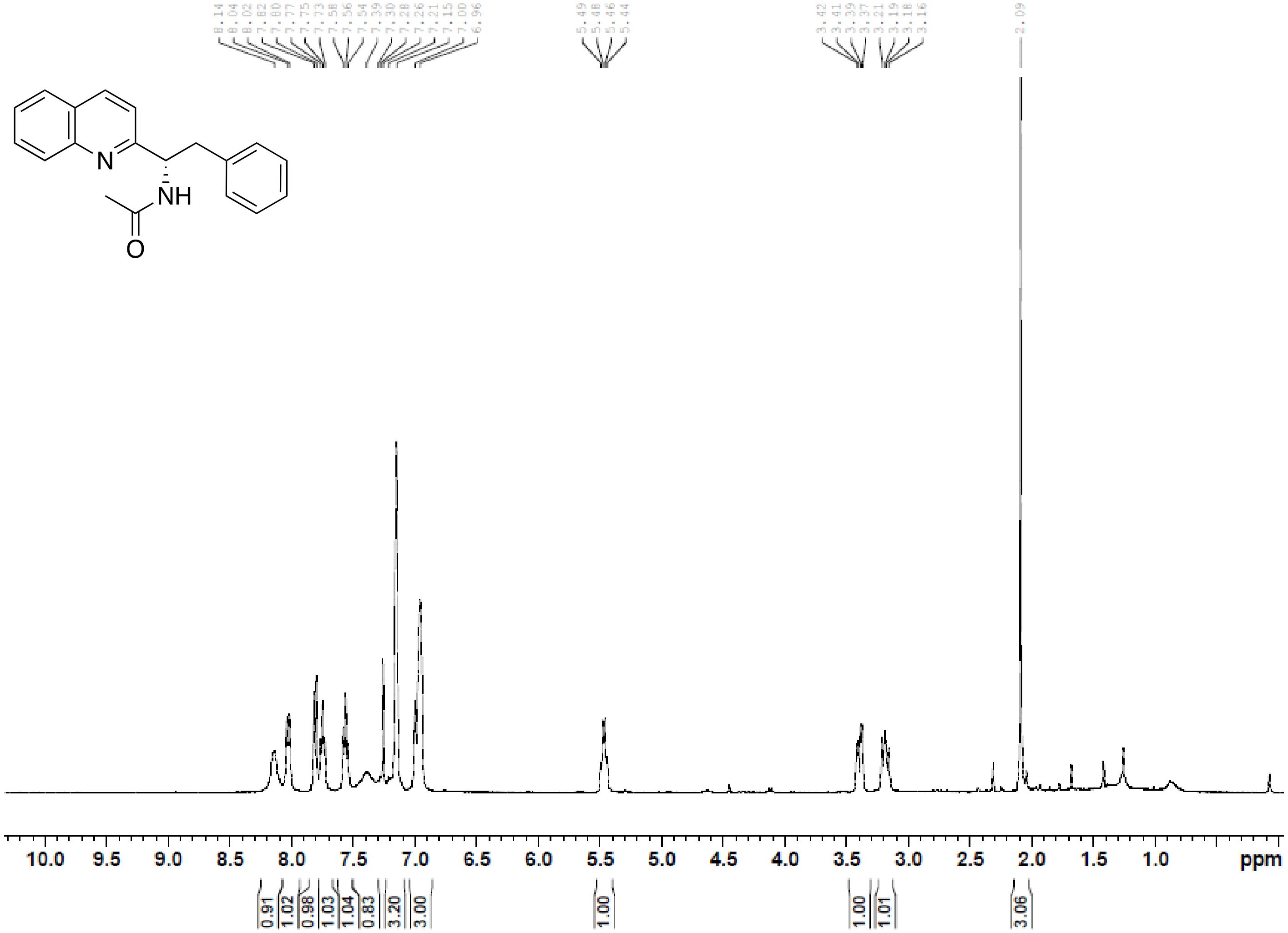

^{13}C NMR (101 MHz, $CDCl_3$) (S)-*N*-(2-phenyl-1-(quinolin-2-yl)ethyl)acetamide (17)

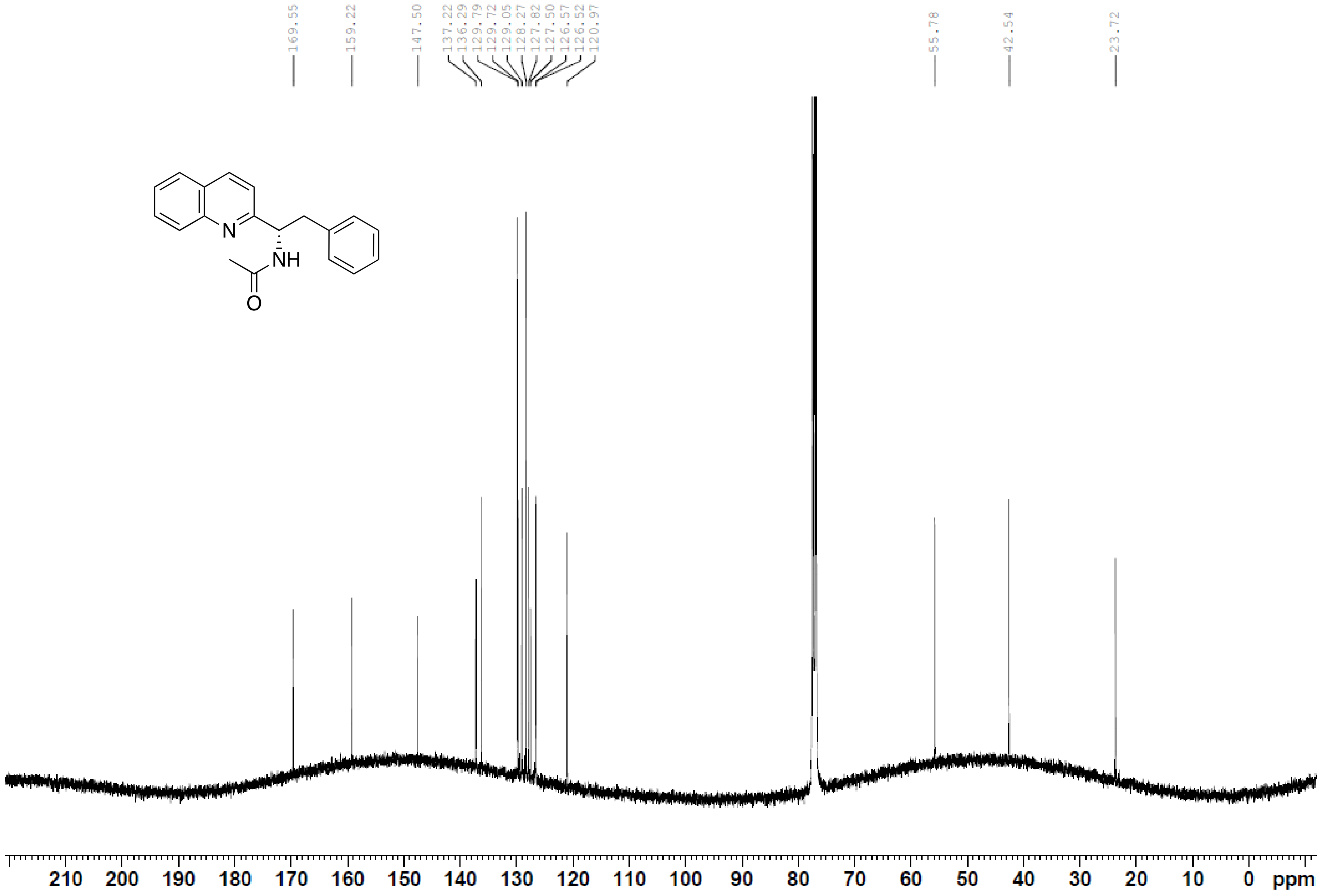

^{1}H NMR (400 MHz, $CDCl_3$) (S)-*N*-(1-(6-methoxyquinolin-2-yl)-2-phenylethyl)acetamide (18)

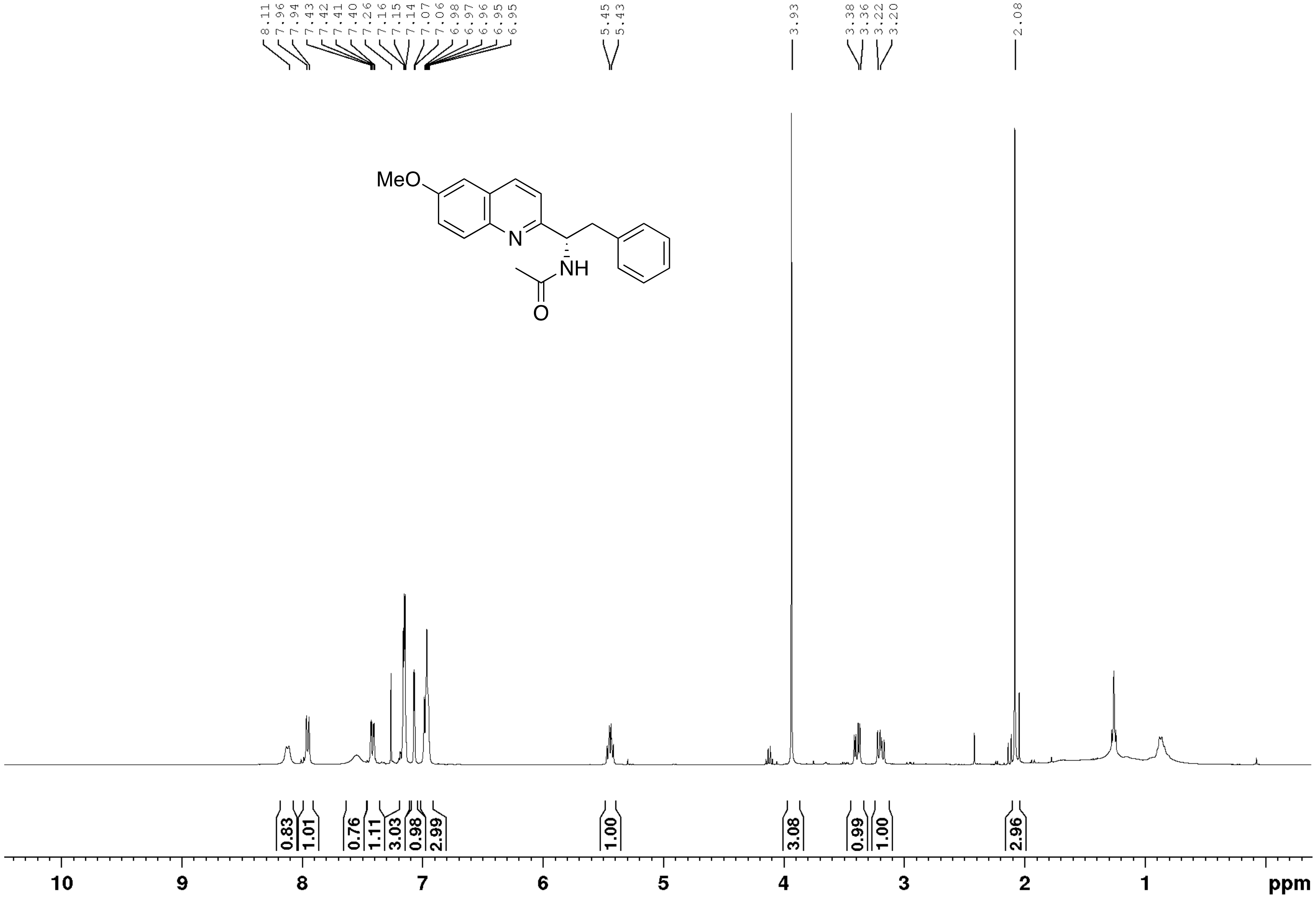

^{13}C NMR (101 MHz, CDCl$_3$) (S)-*N*-(1-(6-methoxyquinolin-2-yl)-2-phenylethyl)acetamide (18)

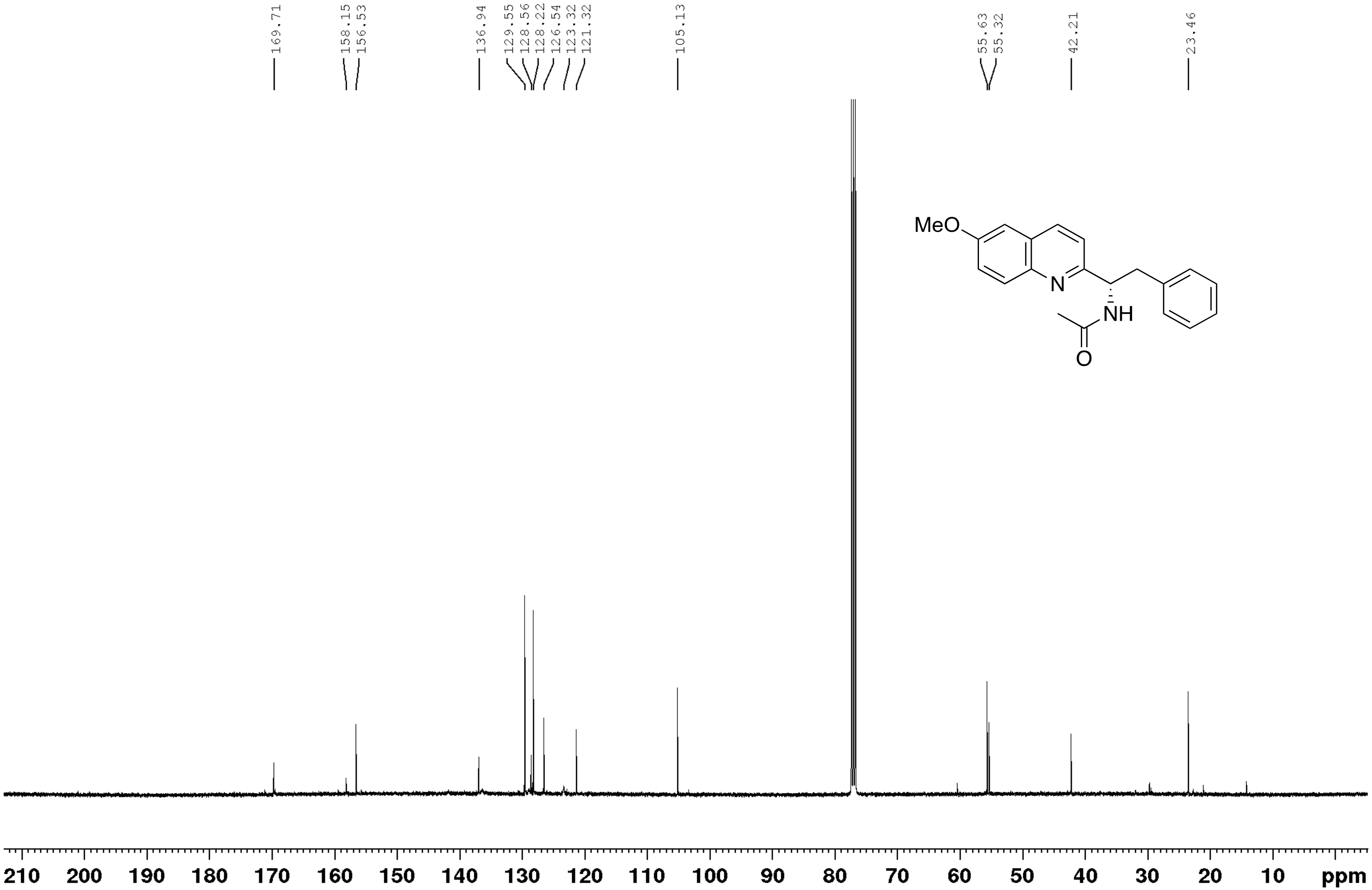

^{1}H NMR (400 MHz, $CDCl_3$) (S)-*N*-(1-(6-chloroquinolin-2-yl)-2-phenylethyl)acetamide (19)

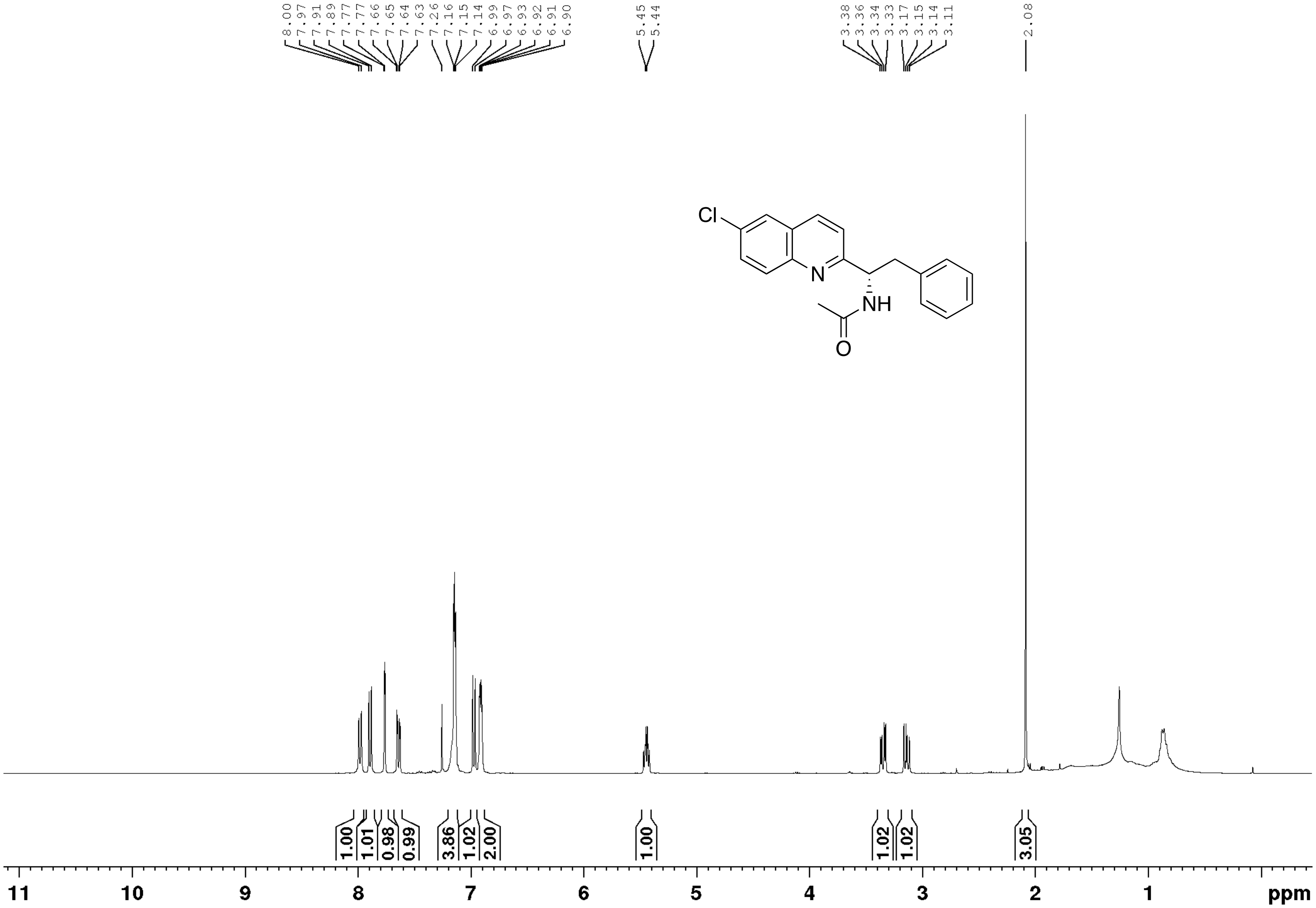

^{13}C NMR (101 MHz, $CDCl_3$) (S)-*N*-(1-(6-chloroquinolin-2-yl)-2-phenylethyl)acetamide (19)

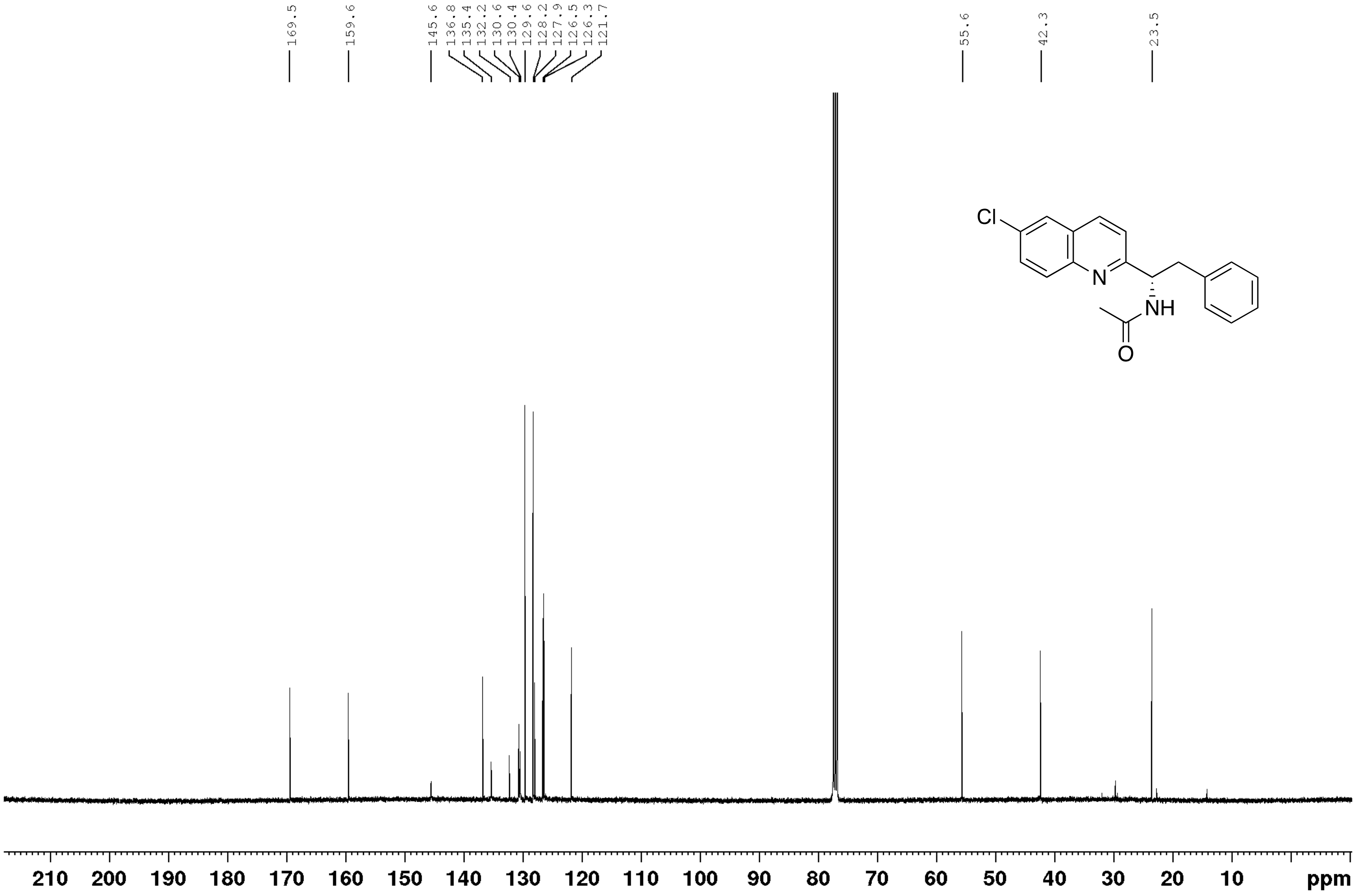

^{1}H NMR (400 MHz, $CDCl_3$) (S)-*N*-(1-(3-methylquinolin-2-yl)-2-phenylethyl)acetamide (20)

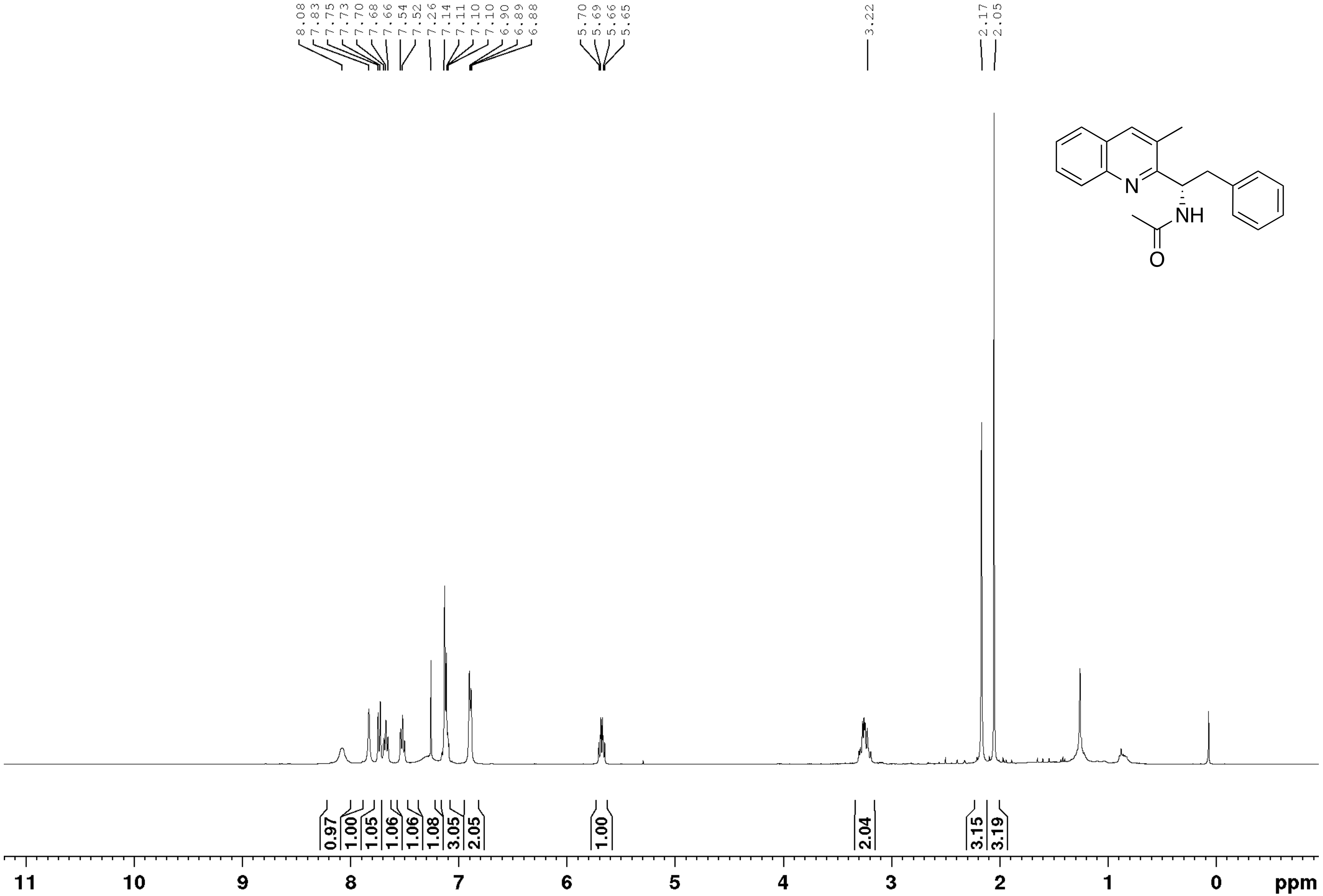

^{13}C NMR (101 MHz, $CDCl_3$) (S)-*N*-(1-(3-methylquinolin-2-yl)-2-phenylethyl)acetamide (20)

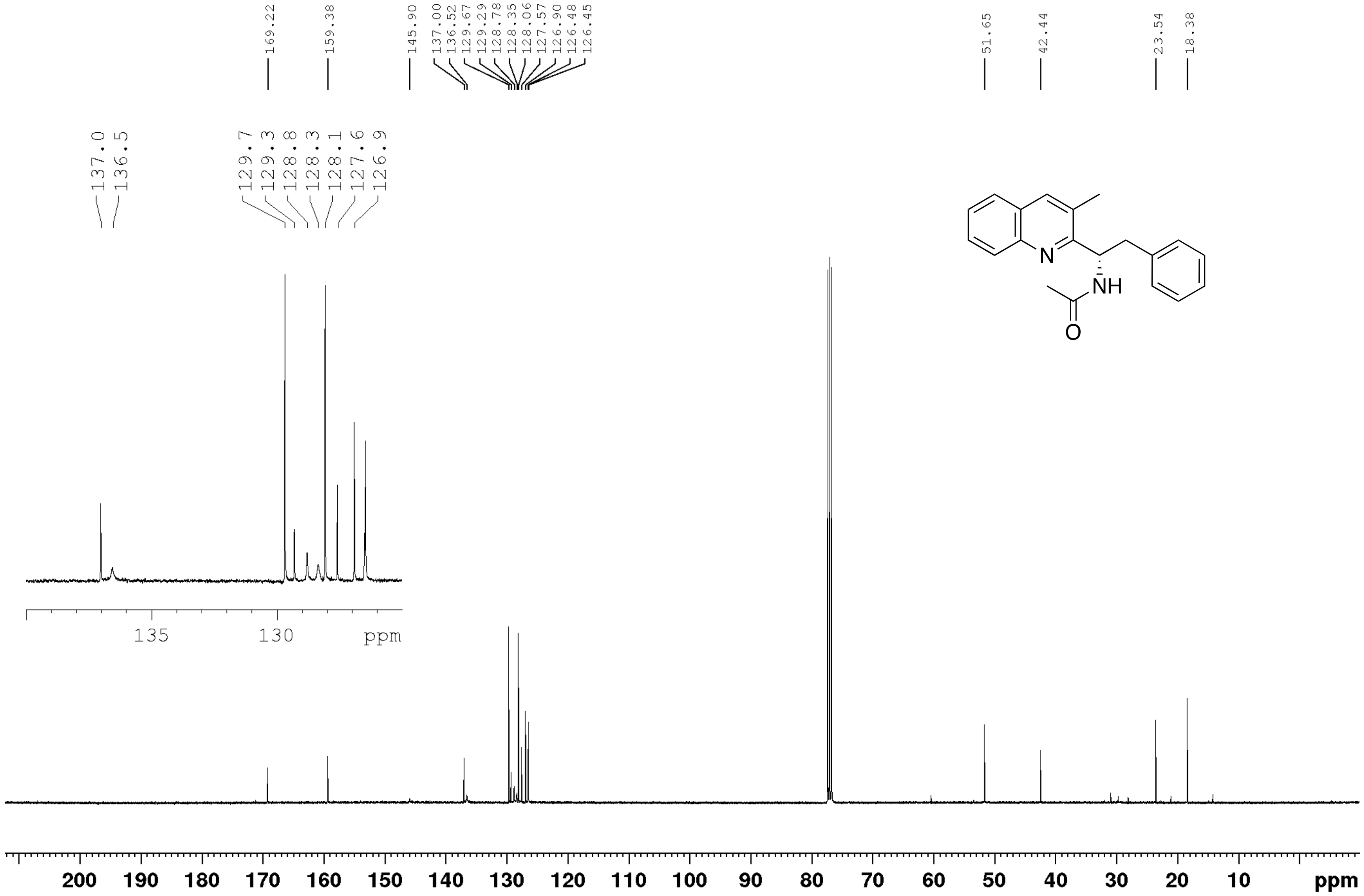

^{1}H NMR (400 MHz, $CDCl_3$) (S)-*N*-(1-(phenanthridin-6-yl)-2-phenylethyl)acetamide (21)

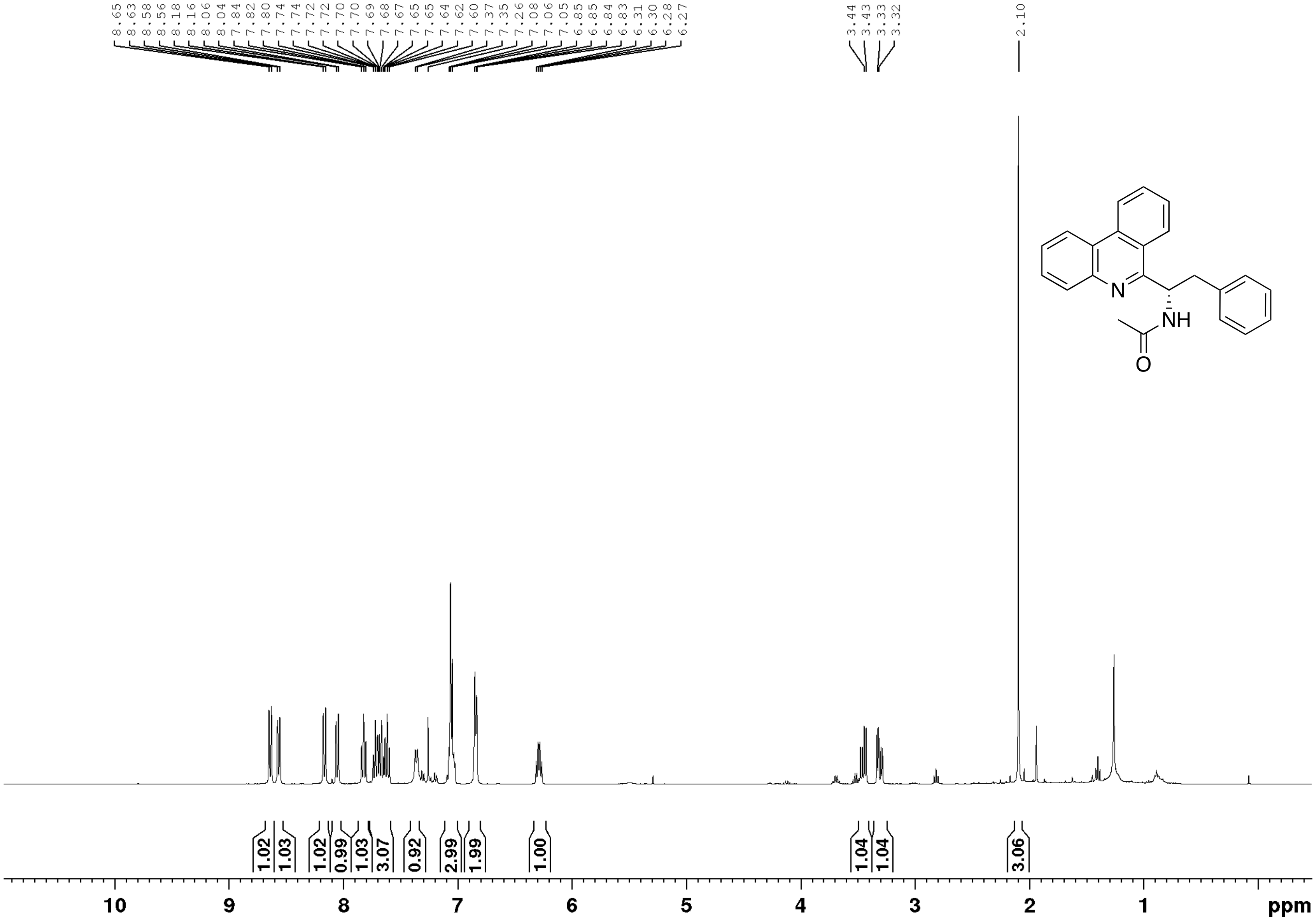

^{13}C NMR (101 MHz, $CDCl_3$) (S)-*N*-(1-(phenanthridin-6-yl)-2-phenylethyl)acetamide (21)

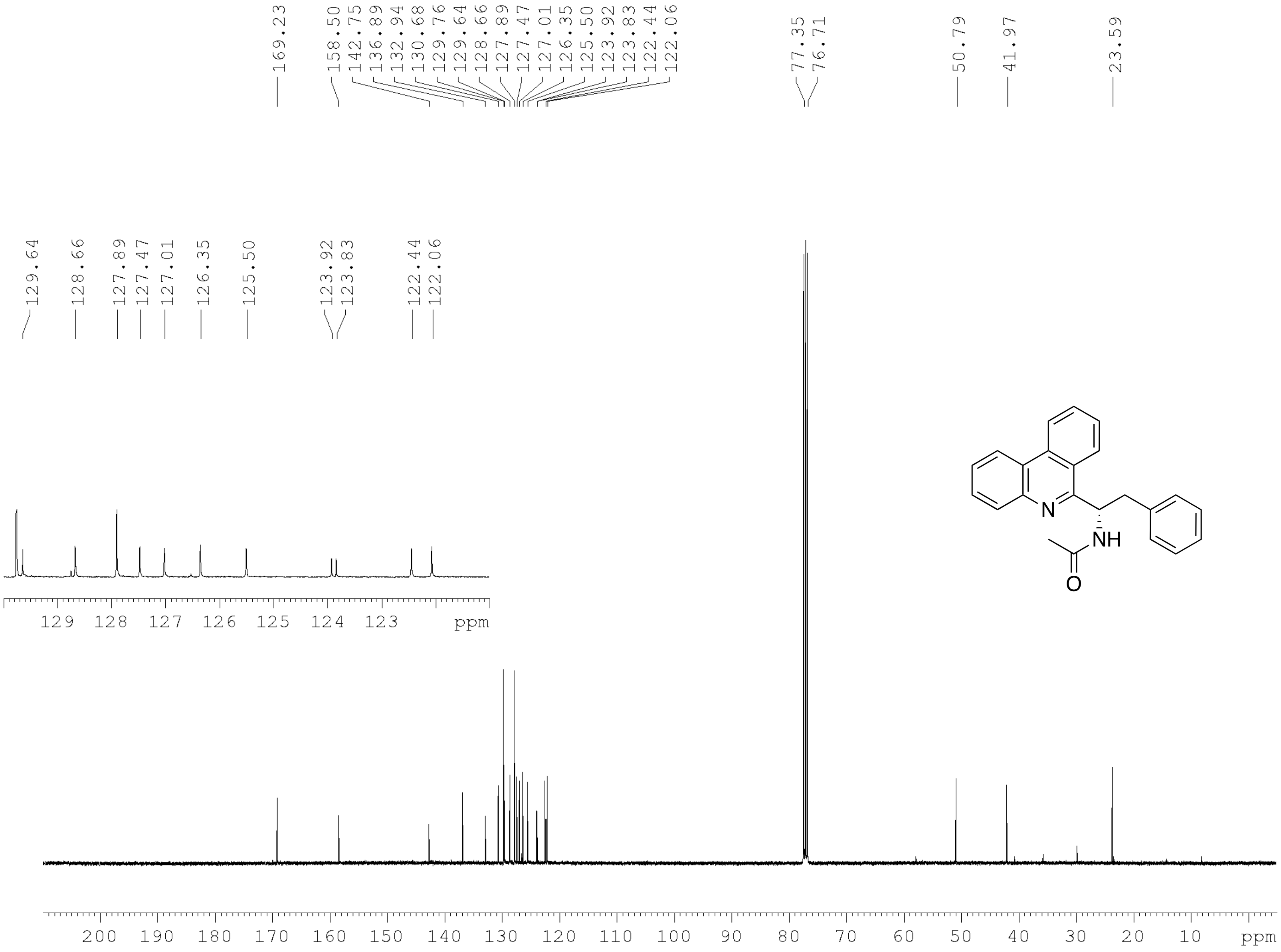

^{1}H NMR (400 MHz, $CDCl_3$) (S)-*N*-(1-(4-(4-bromophenoxy)quinolin-2-yl)-2-phenylethyl)acetamide (22)

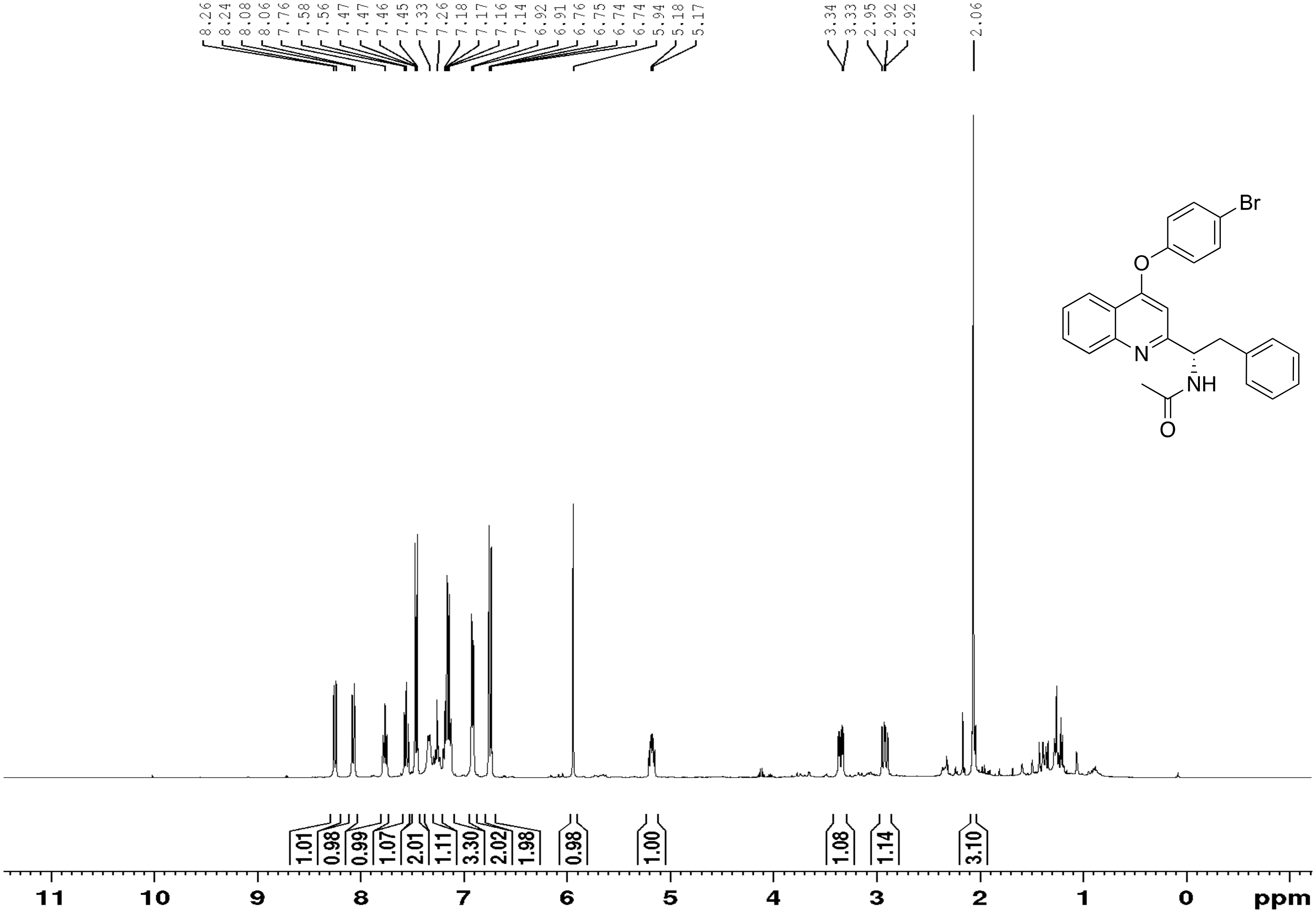

^{13}C NMR (101 MHz, CDCl$_3$) (S)-*N*-(1-(4-(4-bromophenoxy)quinolin-2-yl)-2-phenylethyl)acetamide (22)

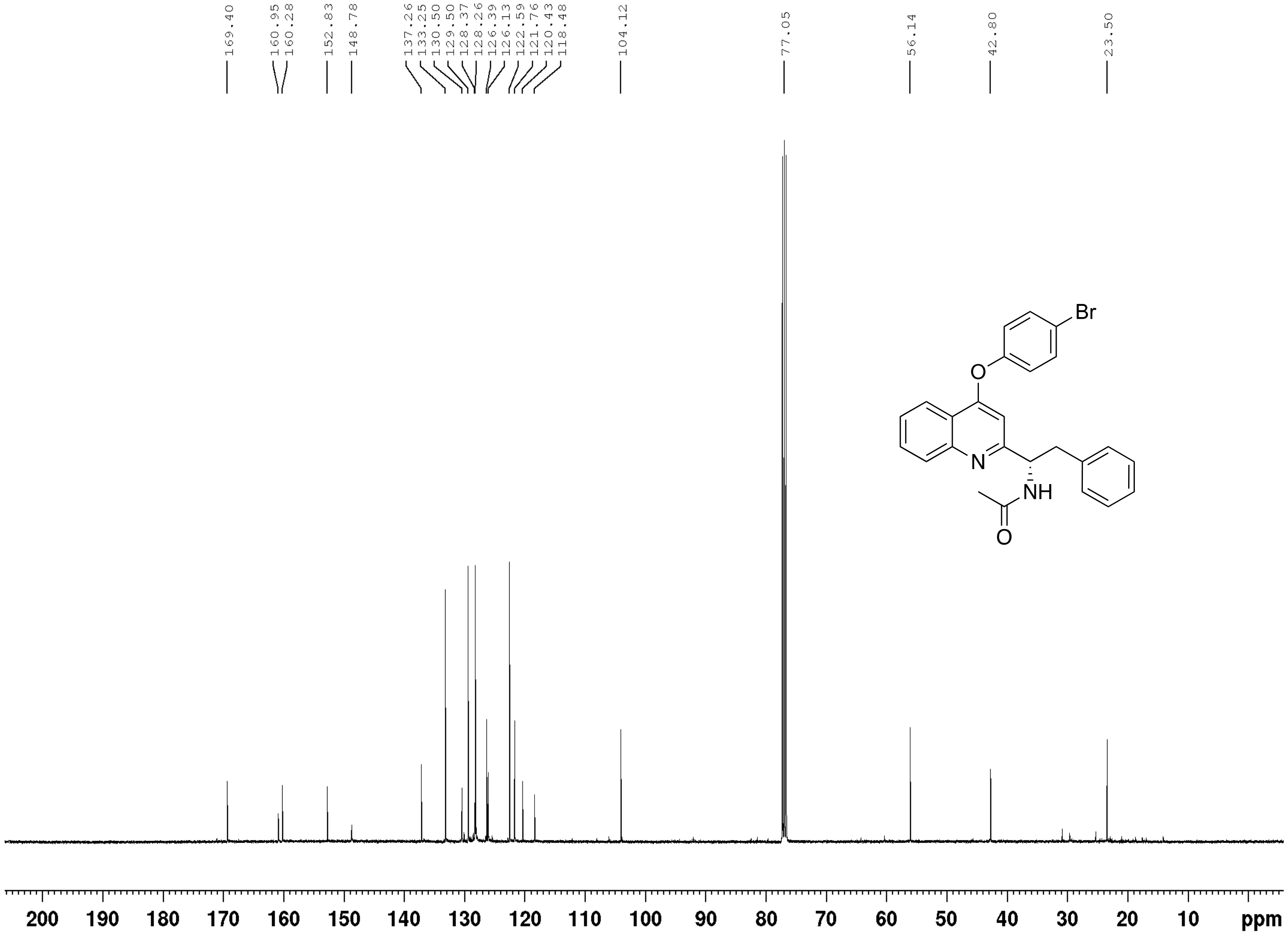

^{1}H NMR (400 MHz, $CDCl_3$) methyl (S)-6-(1-acetamido-2-phenylethyl)-2-methylnicotinate (23)

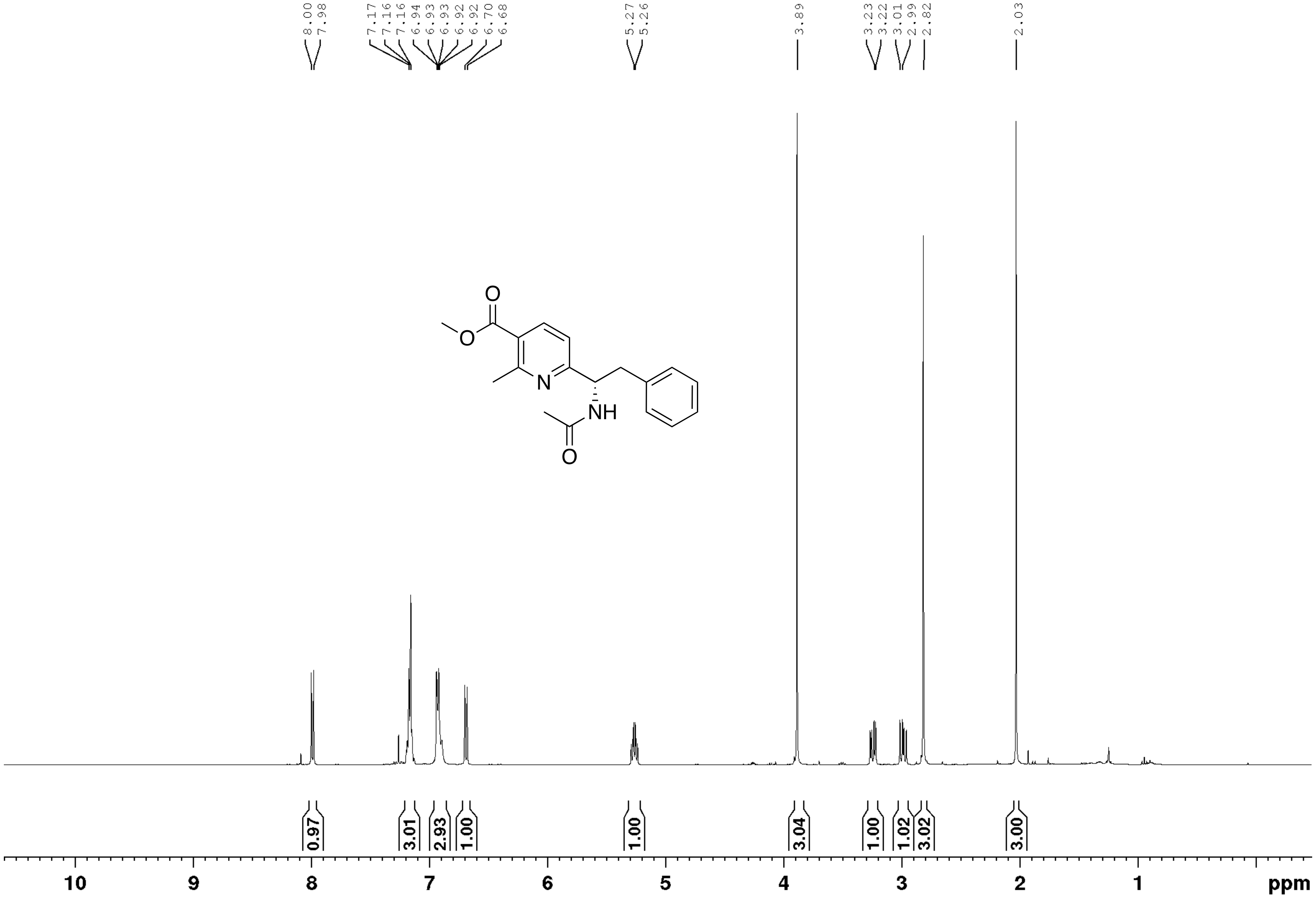

^{13}C NMR (101 MHz, CDCl$_3$) methyl (*S*)-6-(1-acetamido-2-phenylethyl)-2-methylnicotinate (23)

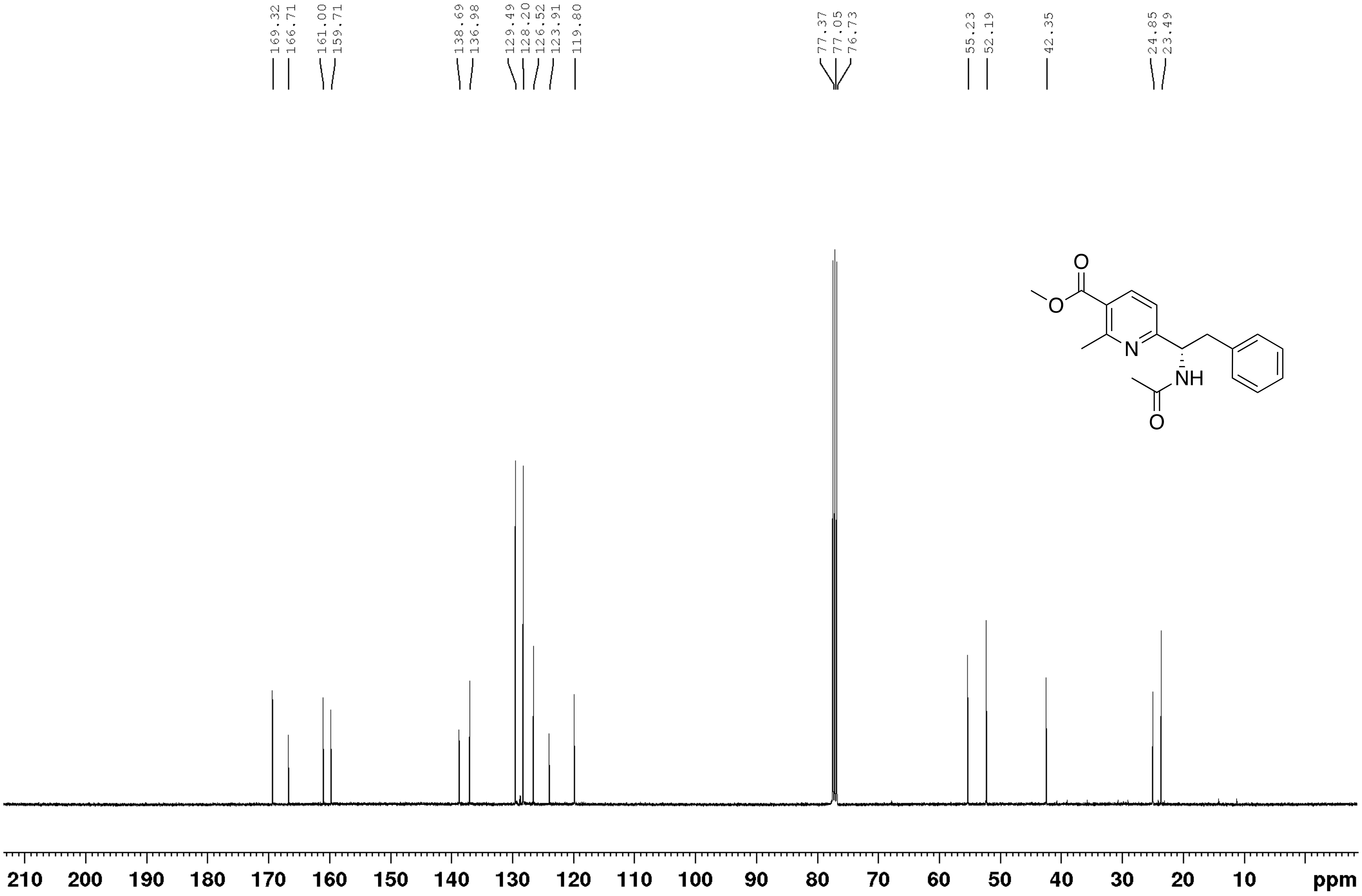

^{1}H NMR (400 MHz, $CDCl_3$) methyl (S)-6-(1-acetamido-2-phenylethyl)nicotinate (24)

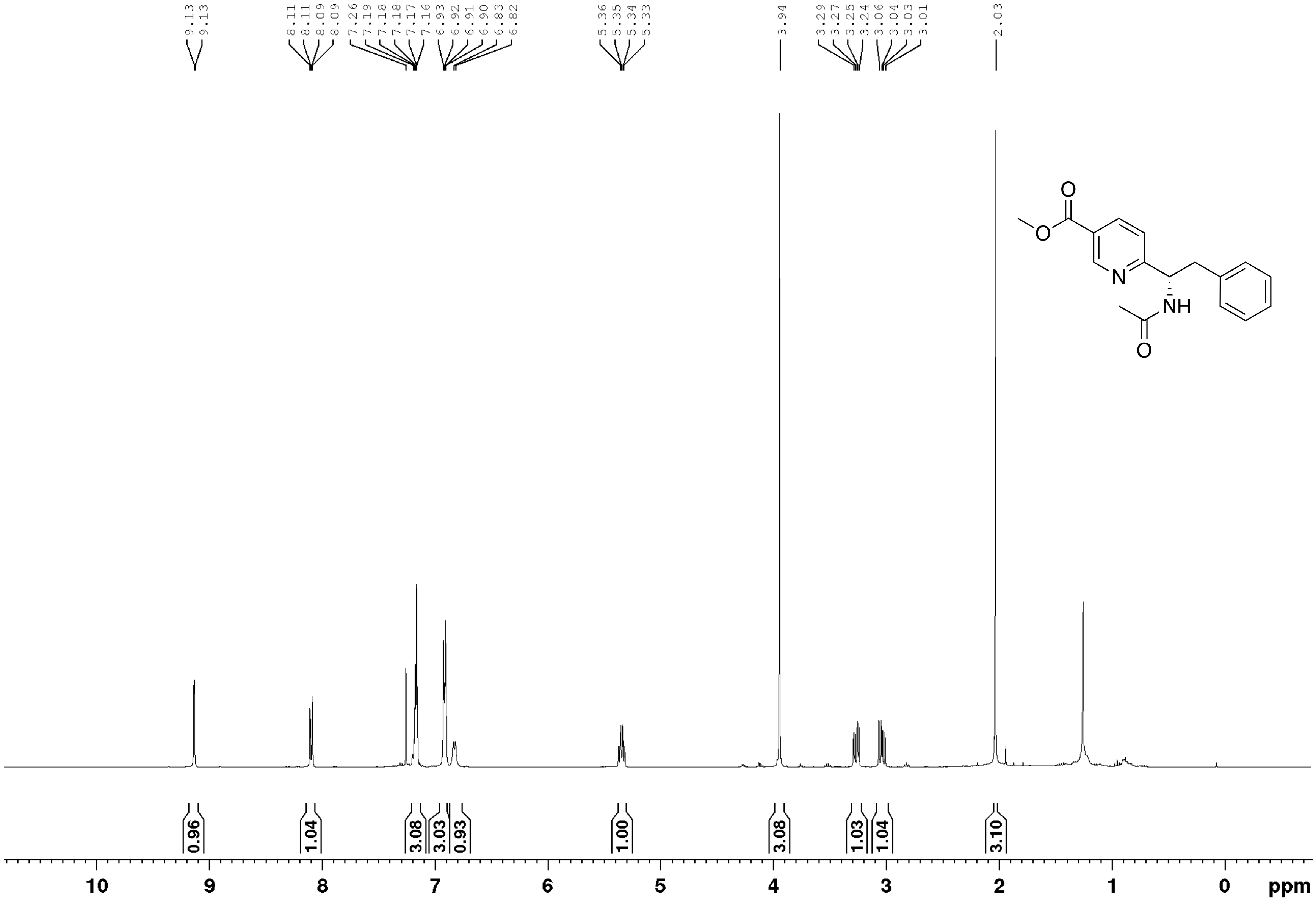

^{13}C NMR (101 MHz, $CDCl_3$) methyl (S)-6-(1-acetamido-2-phenylethyl)nicotinate (24)

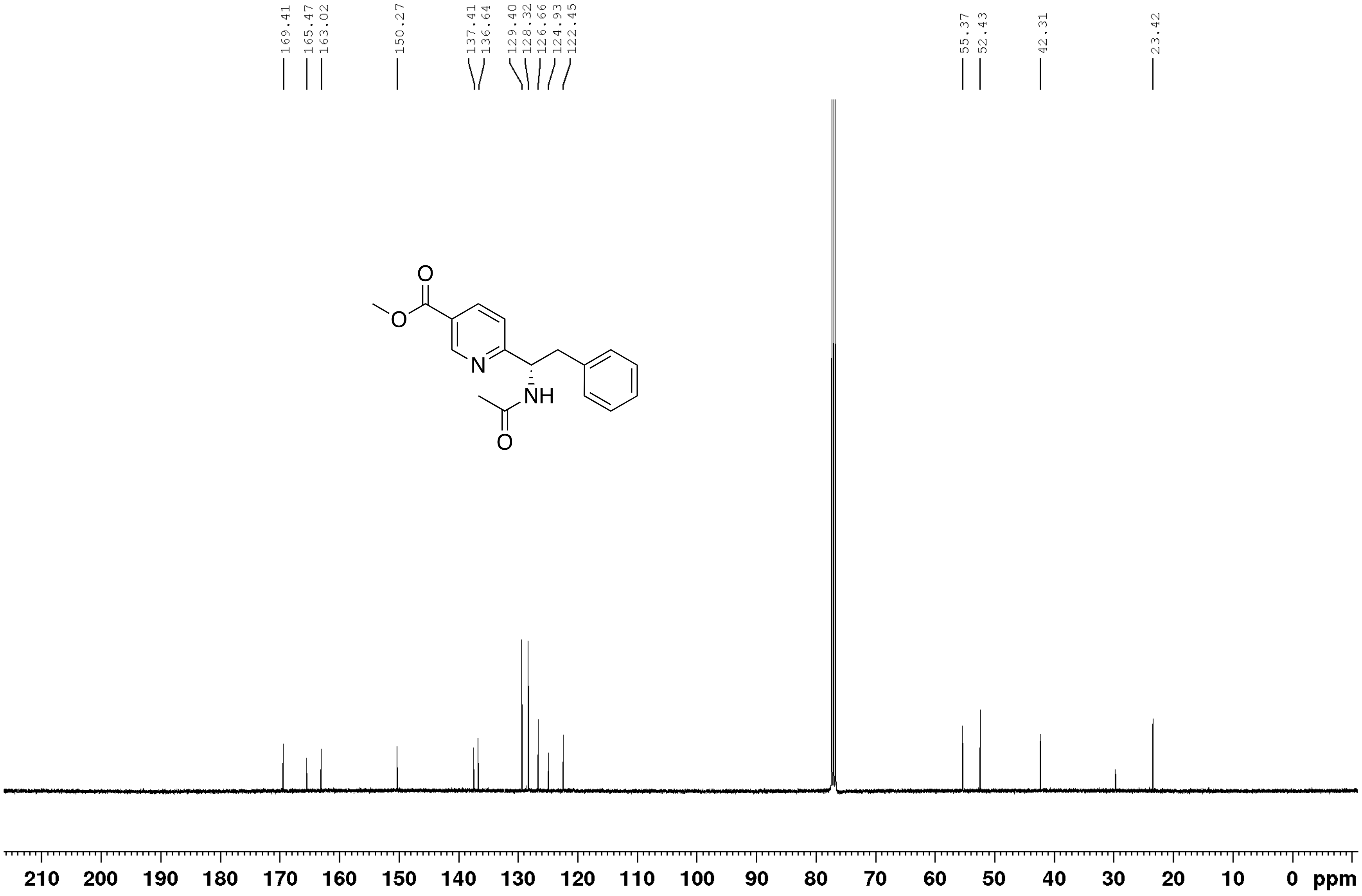

^{1}H NMR (400 MHz, CDCl$_3$) methyl (S)-6-(1-acetamido-2-phenylethyl)-4-methylnicotinate (25)

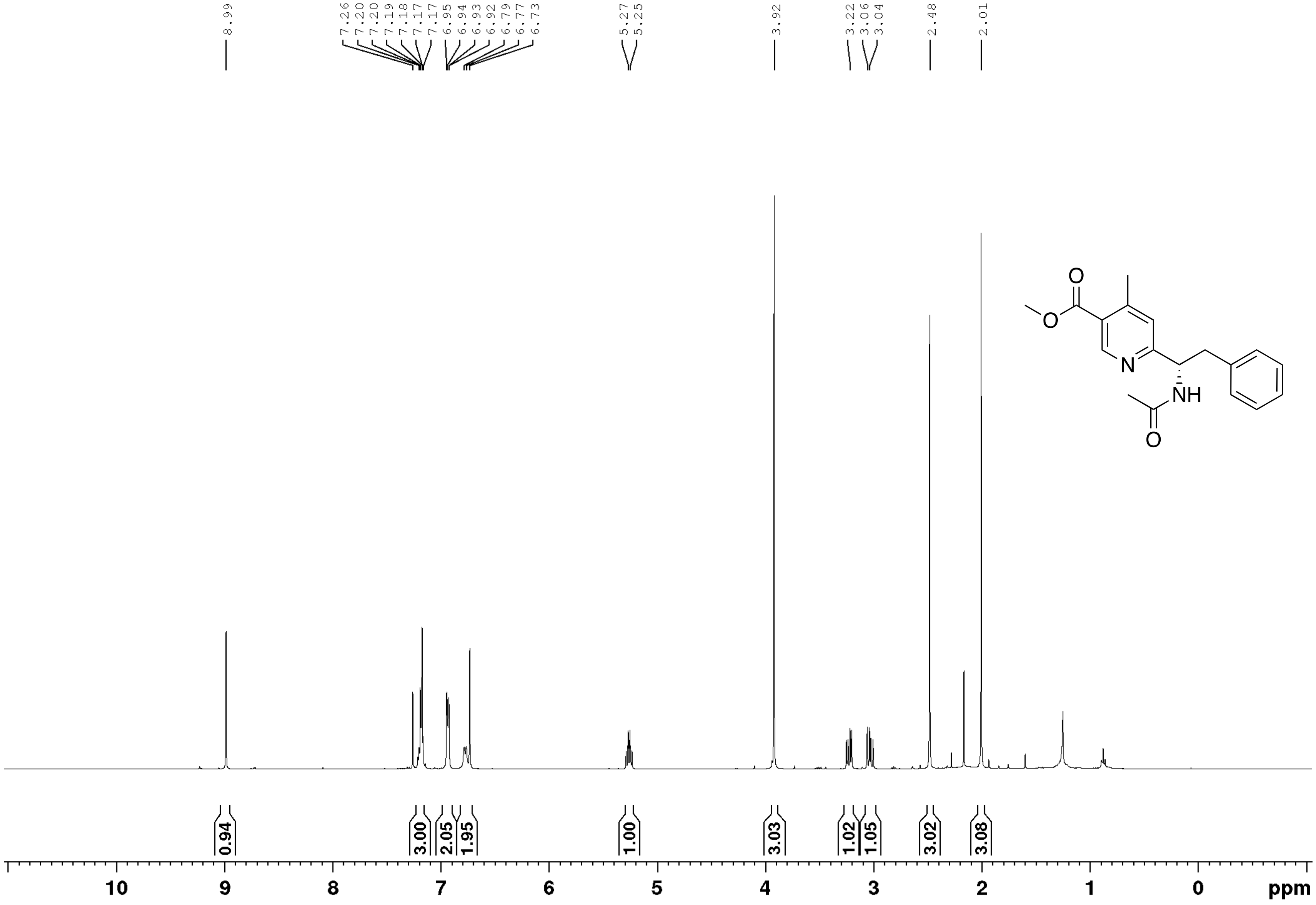

^{13}C NMR (101 MHz, $CDCl_3$) methyl (S)-6-(1-acetamido-2-phenylethyl)-4-methylnicotinate (25)

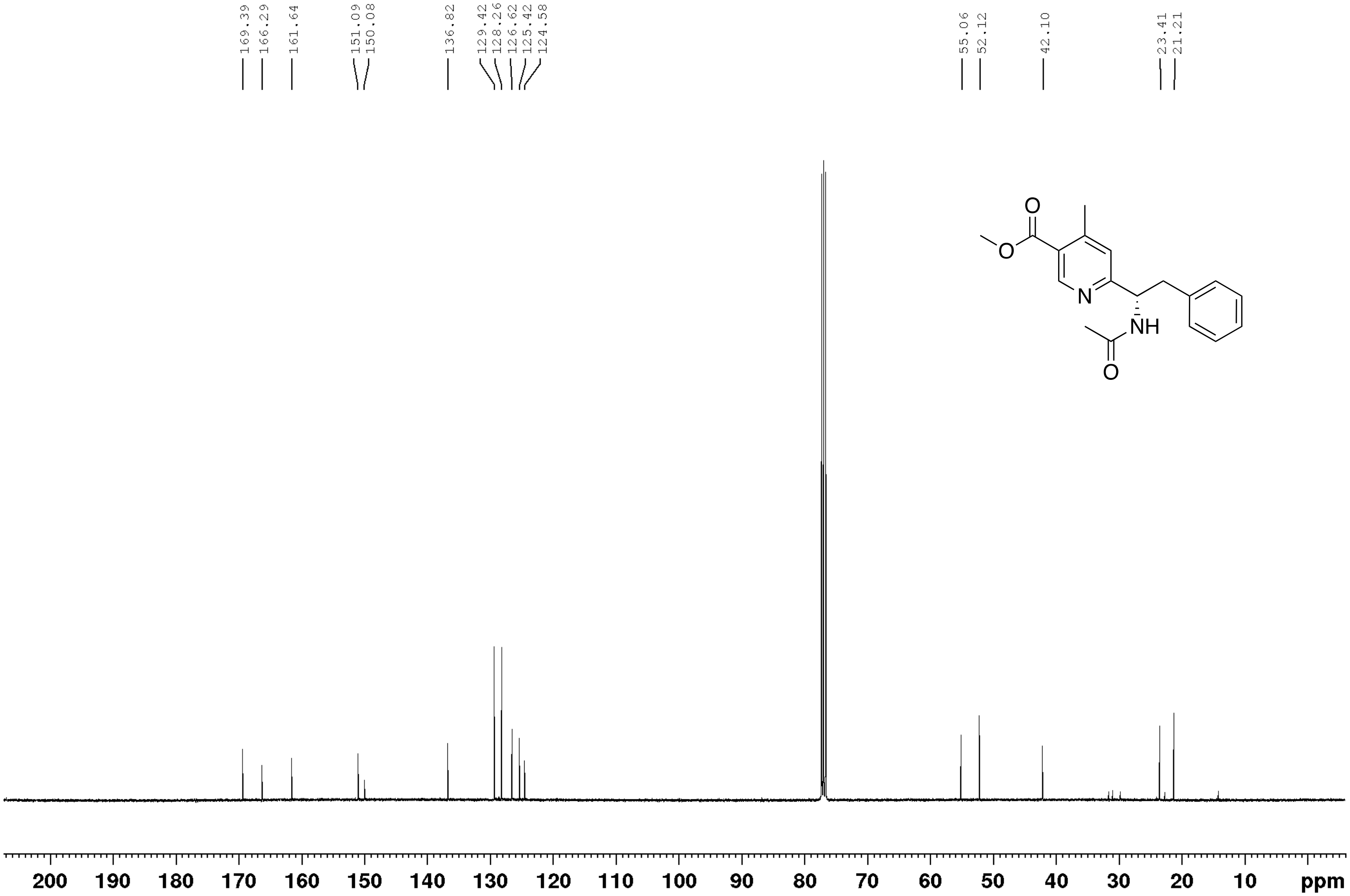

1H NMR (400 MHz, CDCl3) methyl (S)-6-(1-acetamido-2-phenylethyl)-5-methylnicotinate (26)

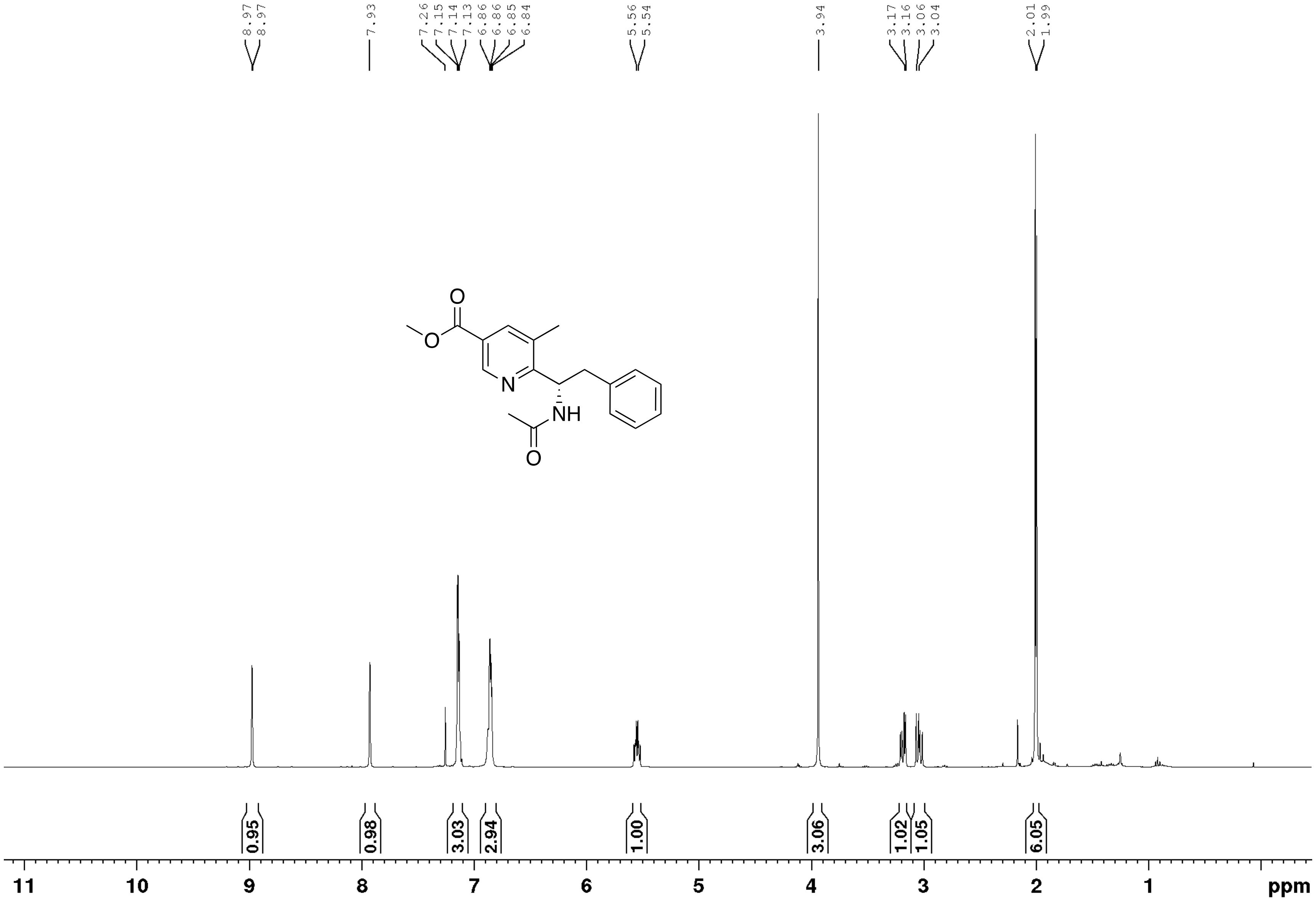

^{13}C NMR (101 MHz, $CDCl_3$) methyl (S)-6-(1-acetamido-2-phenylethyl)-5-methylnicotinate (26)

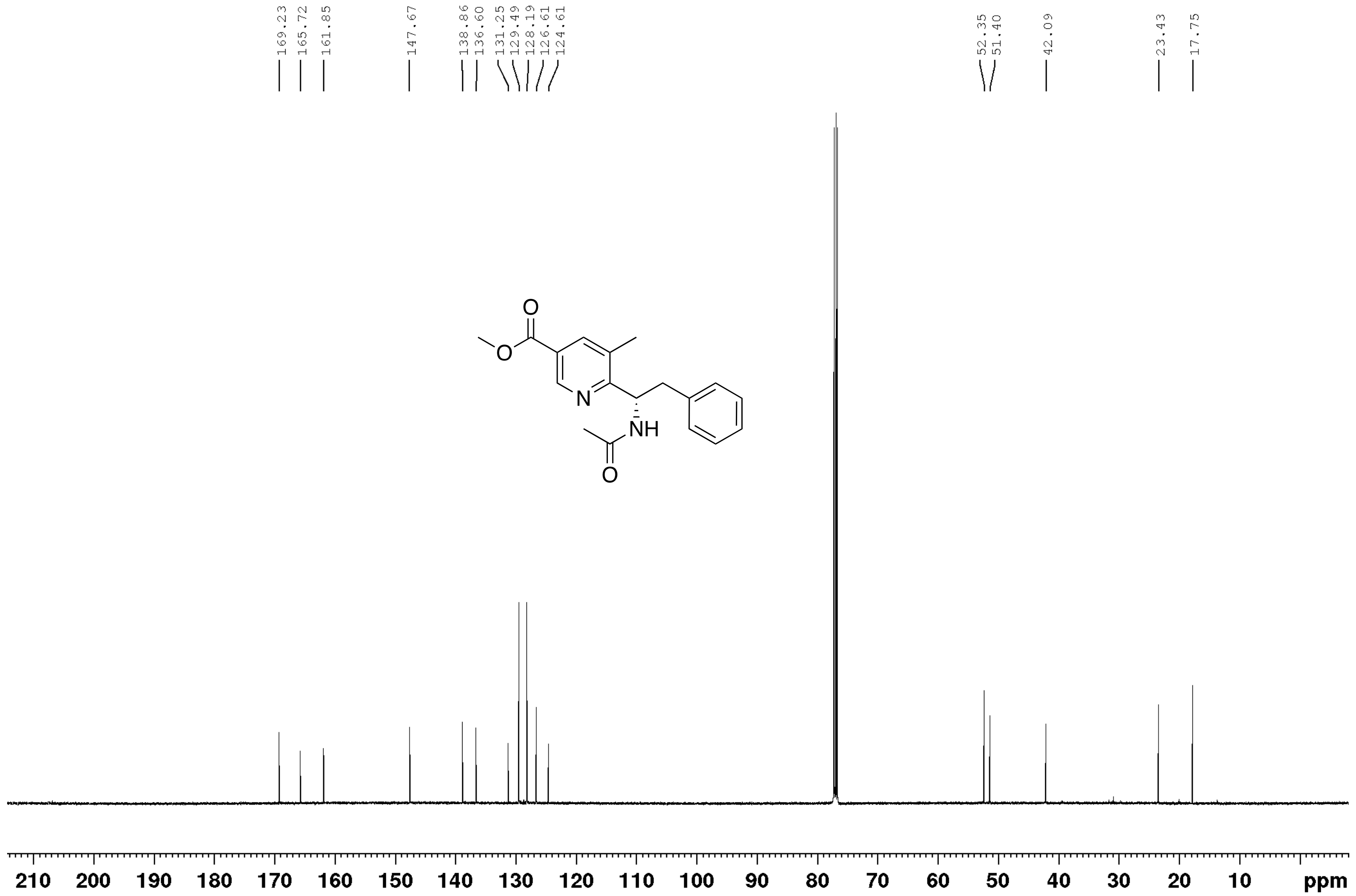

^{1}H NMR (400 MHz, $CDCl_3$) (S)-*N*-(2-phenyl-1-(5-propionylpyridin-2-yl)ethyl)acetamide (27)

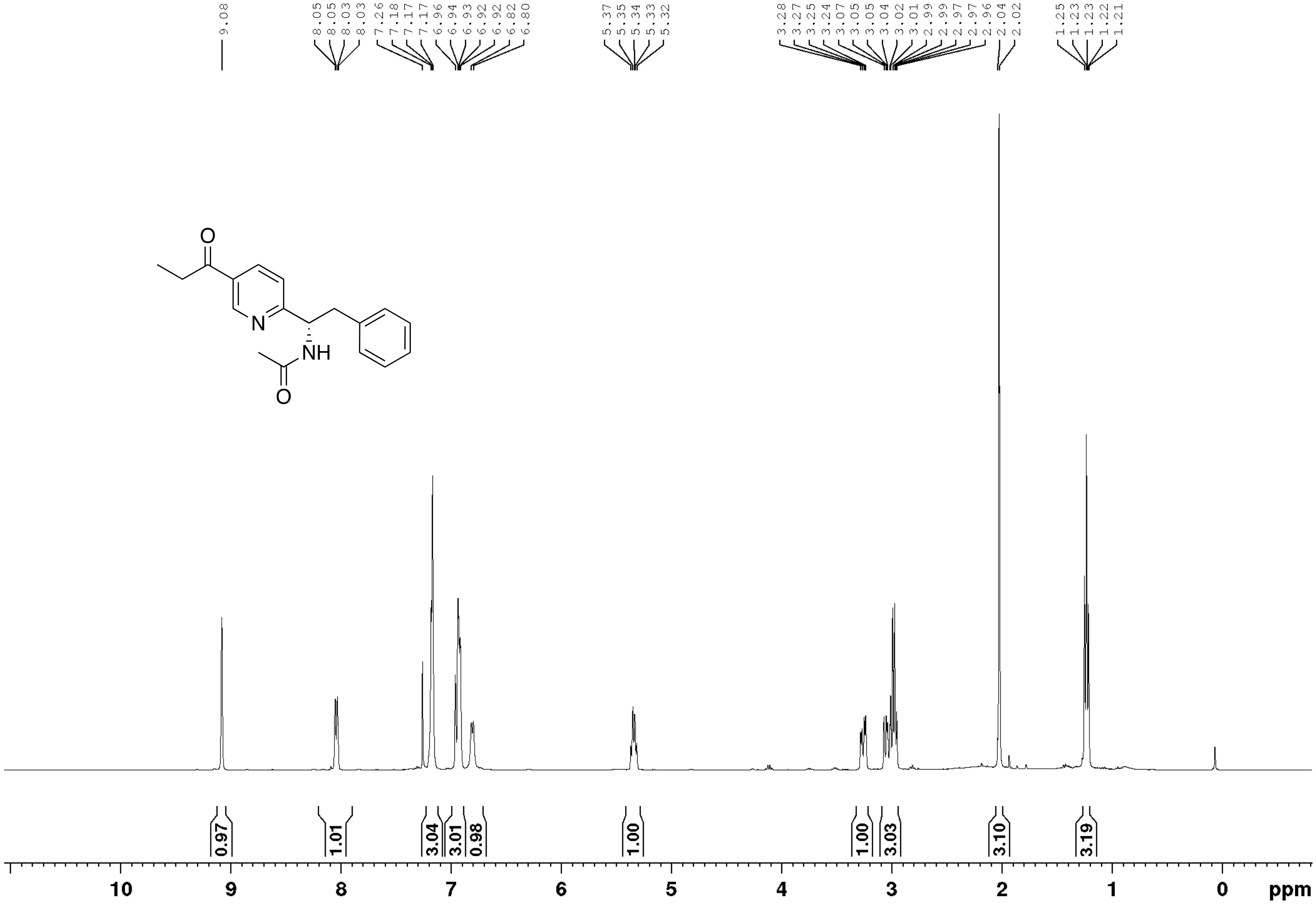

^{13}C NMR (101 MHz, $CDCl_3$) (S)-*N*-(2-phenyl-1-(5-propionylpyridin-2-yl)ethyl)acetamide (27)

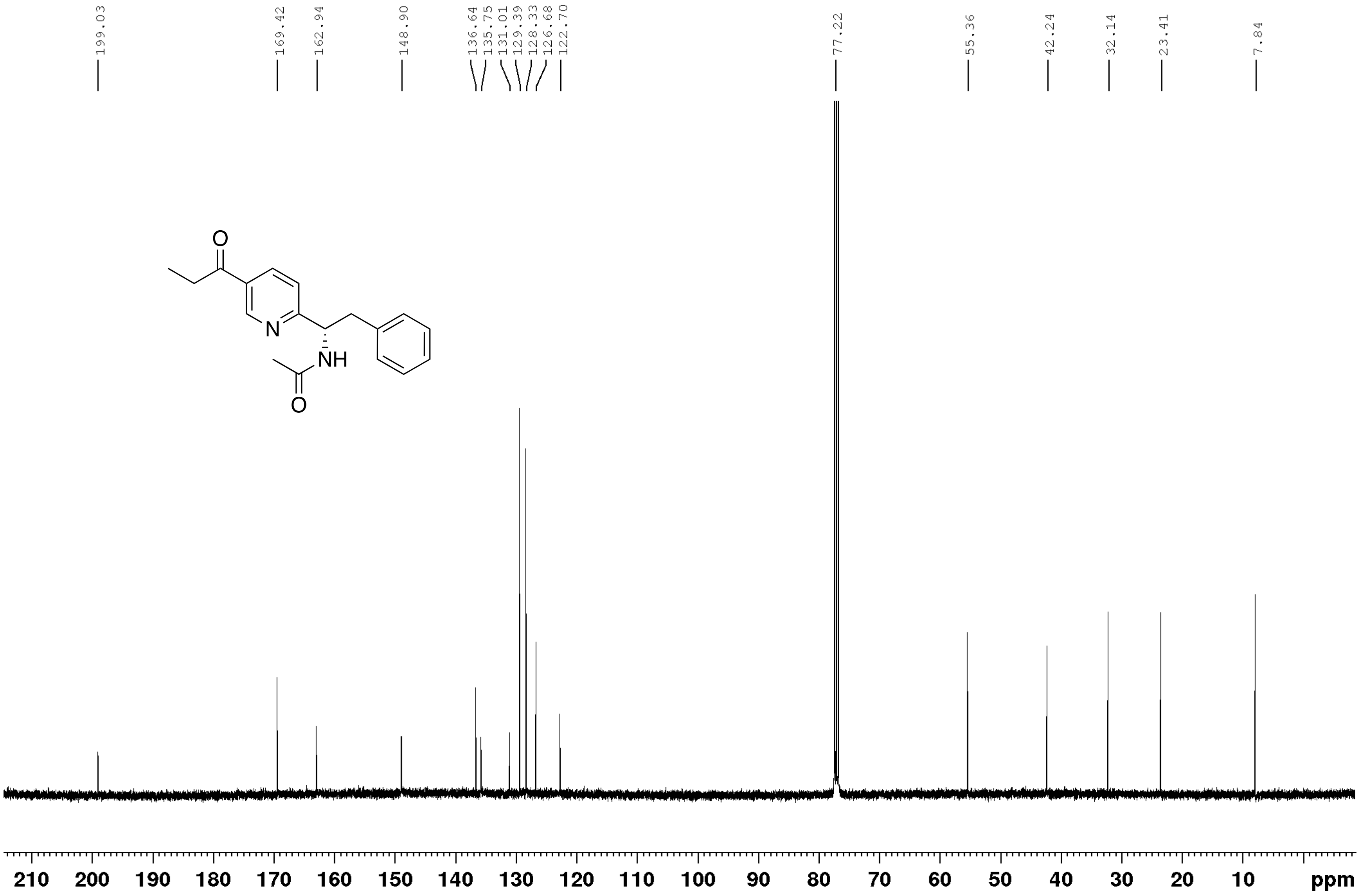

^{1}H NMR (400 MHz, CDCl$_3$) (S)-*N*-(1-(5-cyano-6-methylpyridin-2-yl)-2-phenylethyl)acetamide (28)

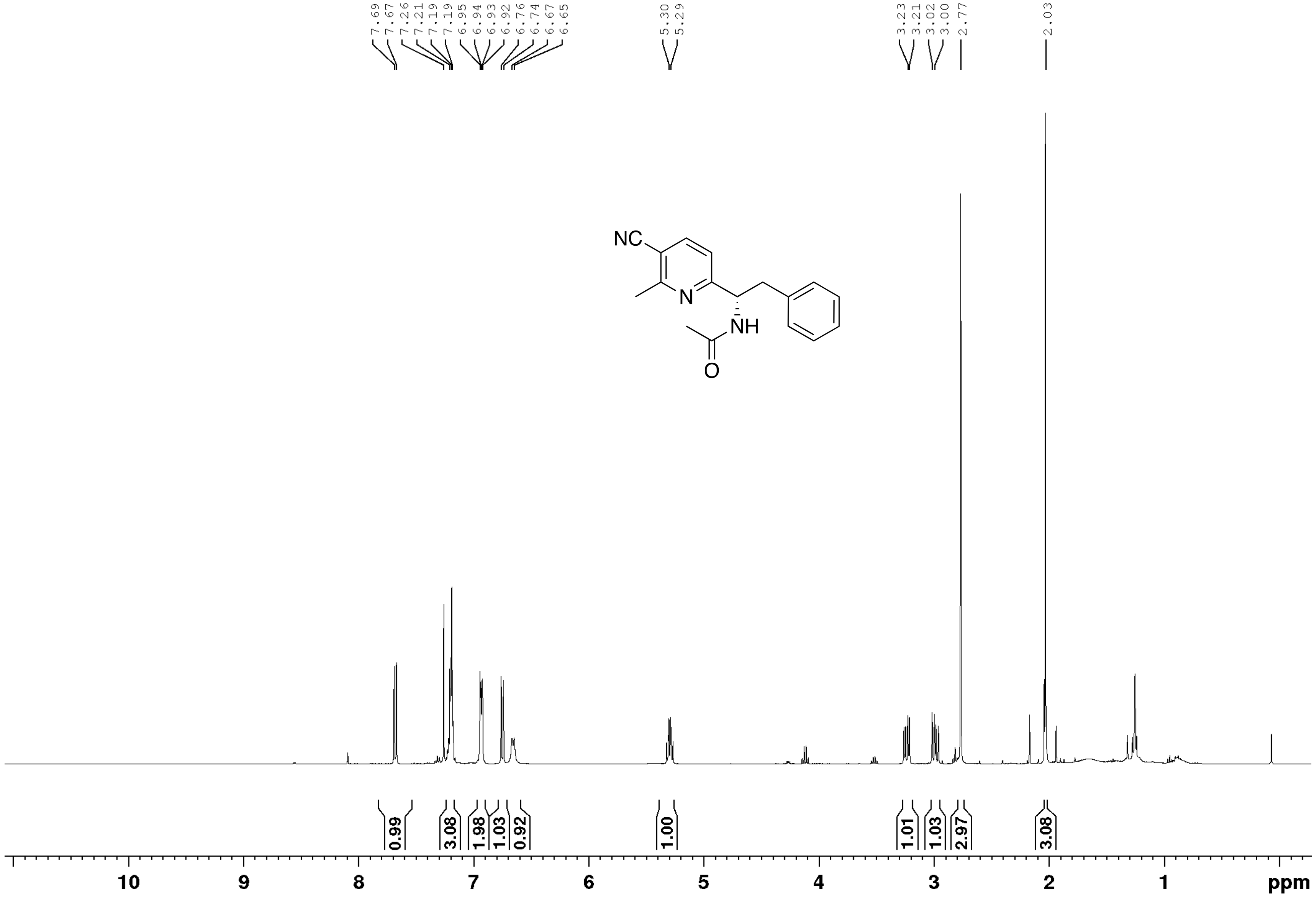

^{13}C NMR (101 MHz, CDCl$_3$) (S)-*N*-(1-(5-cyano-6-methylpyridin-2-yl)-2-phenylethyl)acetamide (28)

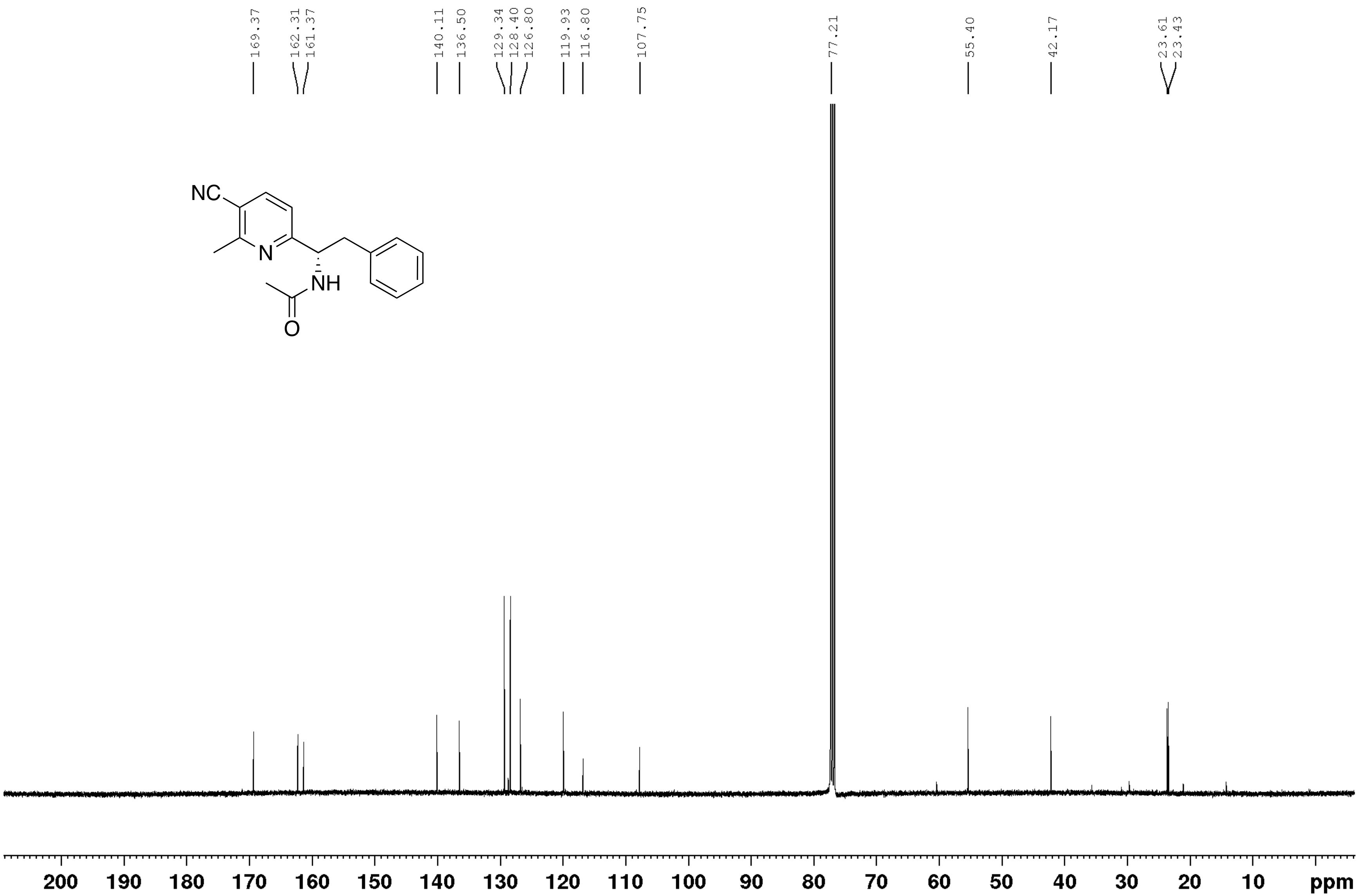

^{1}H NMR (400 MHz, $CDCl_3$) (*S*)-*N*-(1-(2-methyl-6-phenylpyrimidin-4-yl)-2-phenylethyl)acetamide (29)

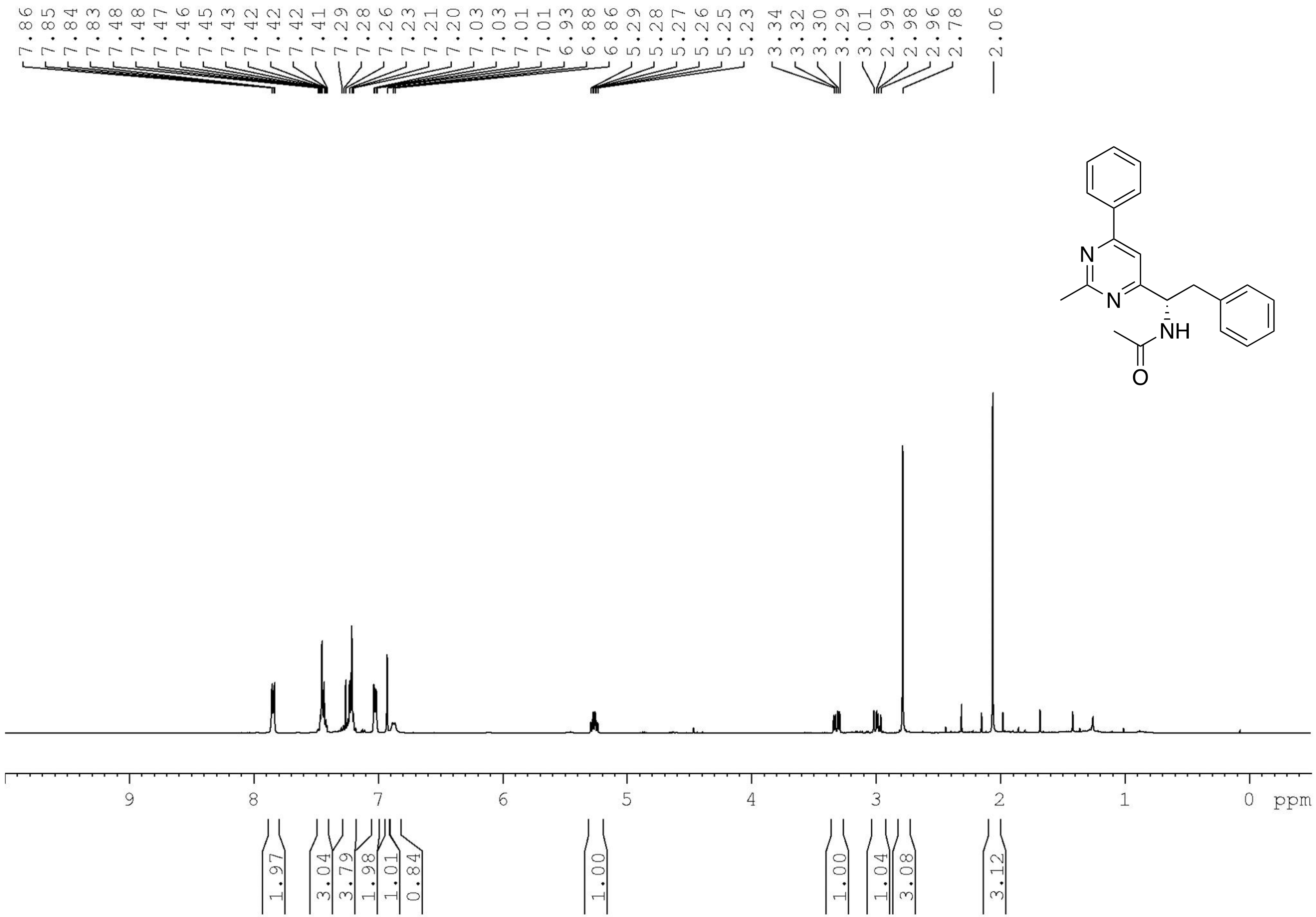

^{13}C NMR (101 MHz, $CDCl_3$) (*S*)-*N*-(1-(2-methyl-6-phenylpyrimidin-4-yl)-2-phenylethyl)acetamide (29)

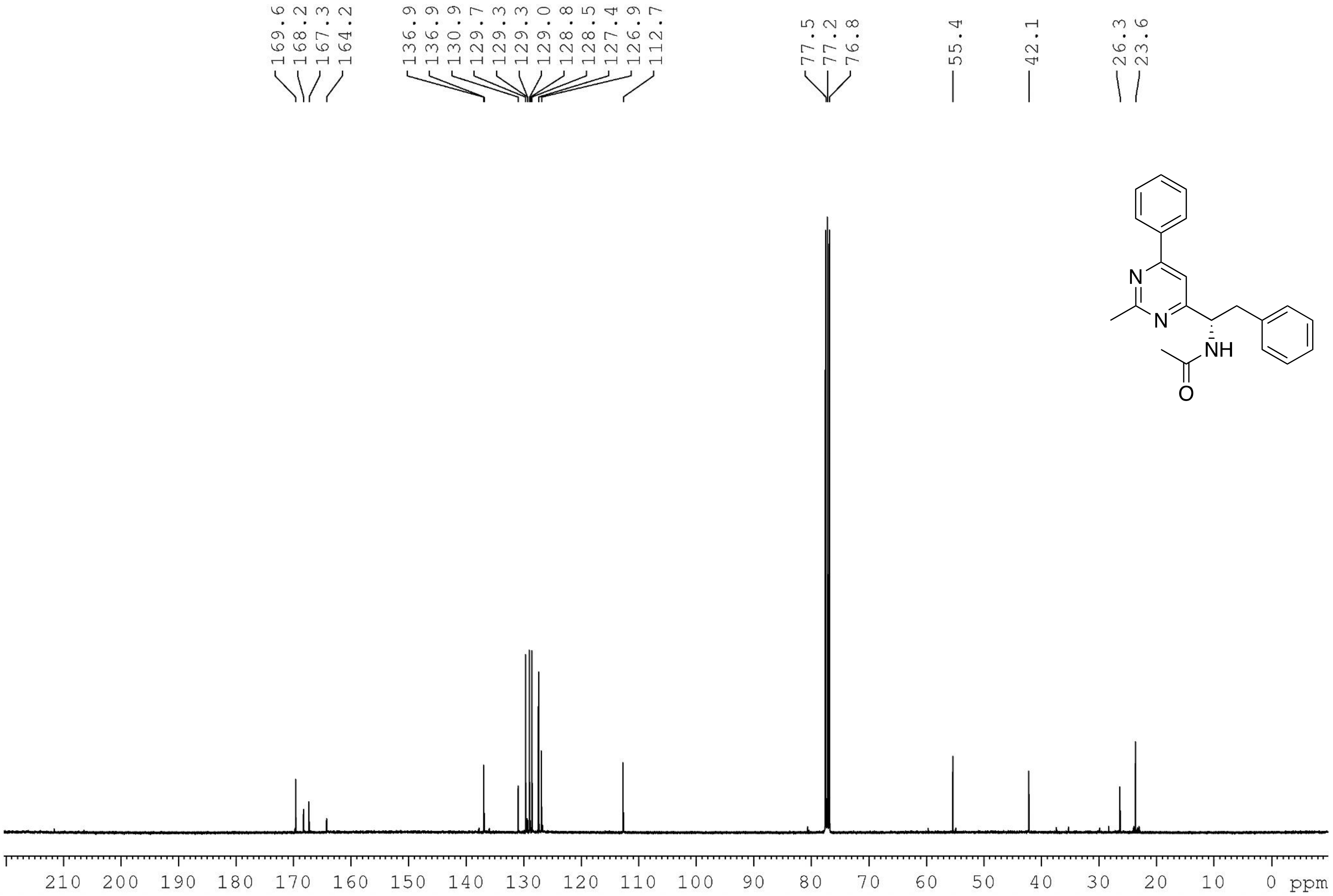

^{1}H NMR (400 MHz, CDCl₃) *tert*-butyl (*S*)-(1-(4-methylquinolin-2-yl)-2-phenylethyl)carbamate (30)

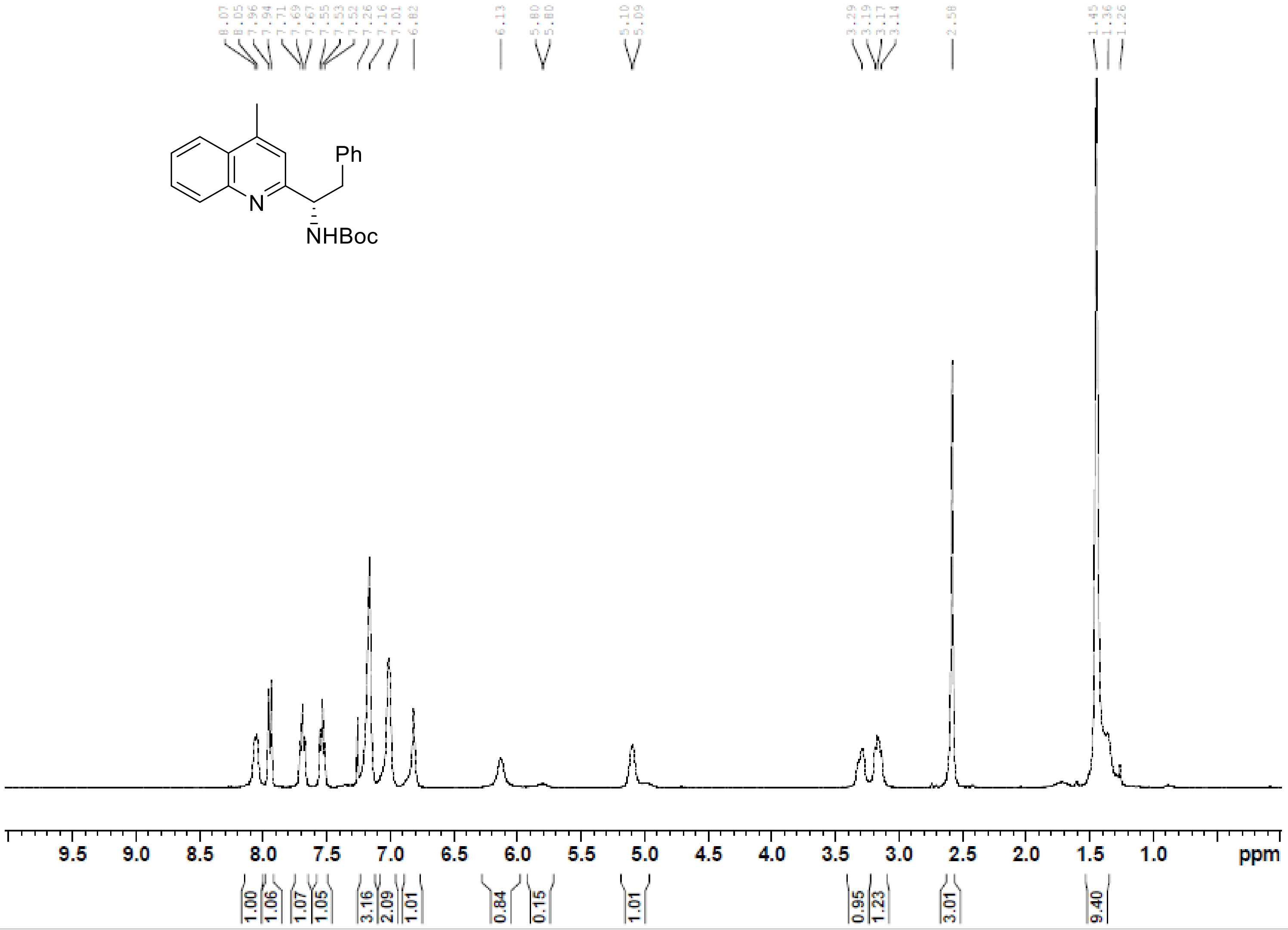

^{13}C NMR (101 MHz, CDCl$_3$) *tert*-butyl (*S*)-(1-(4-methylquinolin-2-yl)-2-phenylethyl)carbamate (30)

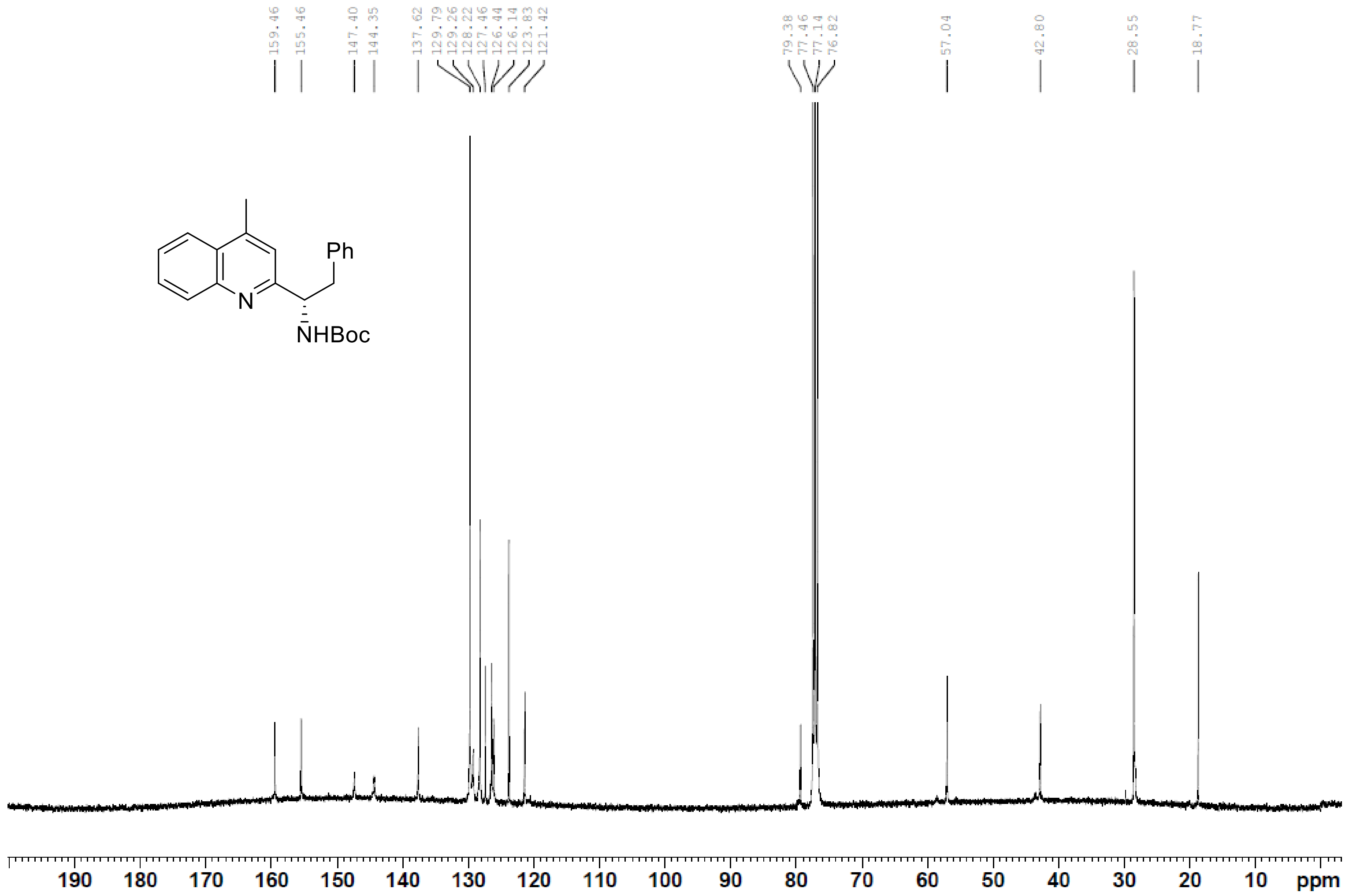

HPLC and SFC Traces

(*S*)-*N*-(1-(4-methylquinolin-2-yl)-2-phenylethyl)acetamide (3)

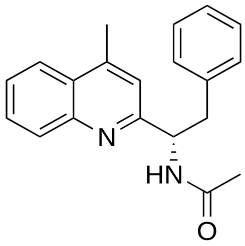

HPLC Conditions: Chiralpak IC (Hexane/^{i}PrOH = 70/30, 1.0 mL min^{-1}, 30 °C, 219 nm)

<u>95% ee</u>

t_R = 9.0 (minor), 10.4 (major) minutes

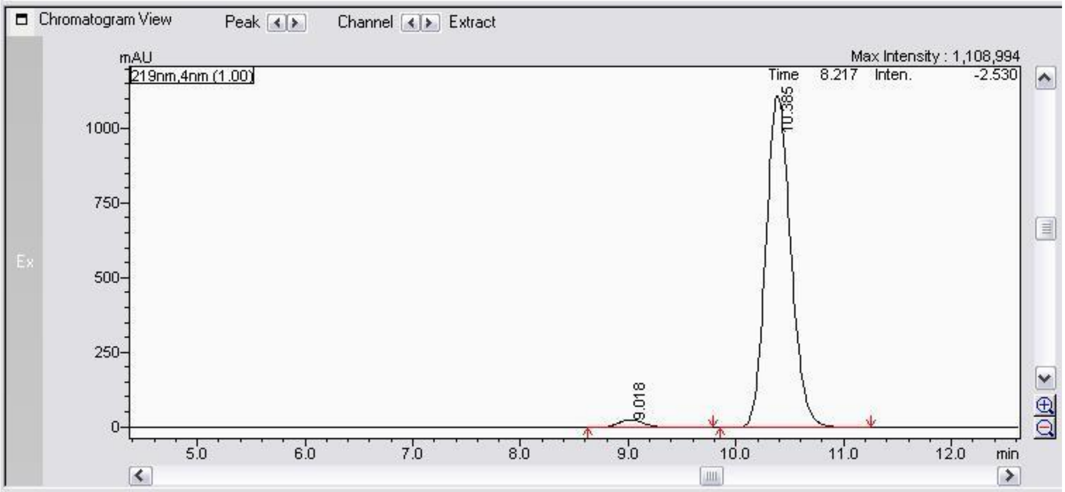

Results View - Peak Table

Peak Table | Compound | Group | Calibration Curve

Peak#	Ret. Time	Area	Height	Area%
1	9.018	467366	27076	2.546
2	10.385	17891085	1112122	97.454
Total		18358451	1139198	100.000

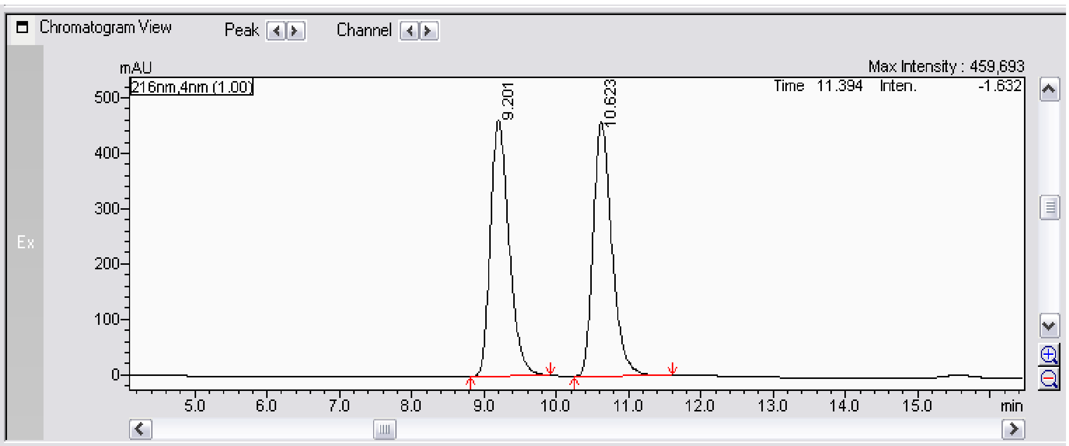

Results View - Peak Table

Peak Table | Compound | Group | Calibration Curve

Peak#	Ret. Time	Area	Height	Area%
1	9.201	8287502	462137	49.976
2	10.623	8295424	459221	50.024
Total		16582927	921358	100.000

(*S*)-*N*-(2-(4-fluorophenyl)-1-(4-methylquinolin-2-yl)ethyl)acetamide (4)

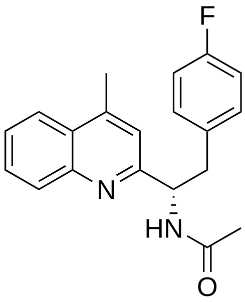

Chiralpak SC (CO_2/MeOH = 85/15, 2.5 mL min^{-1}, 40 °C, 272 nm)

95% ee

t_R = 4.2 minutes (minor), 4.8 minutes (major)

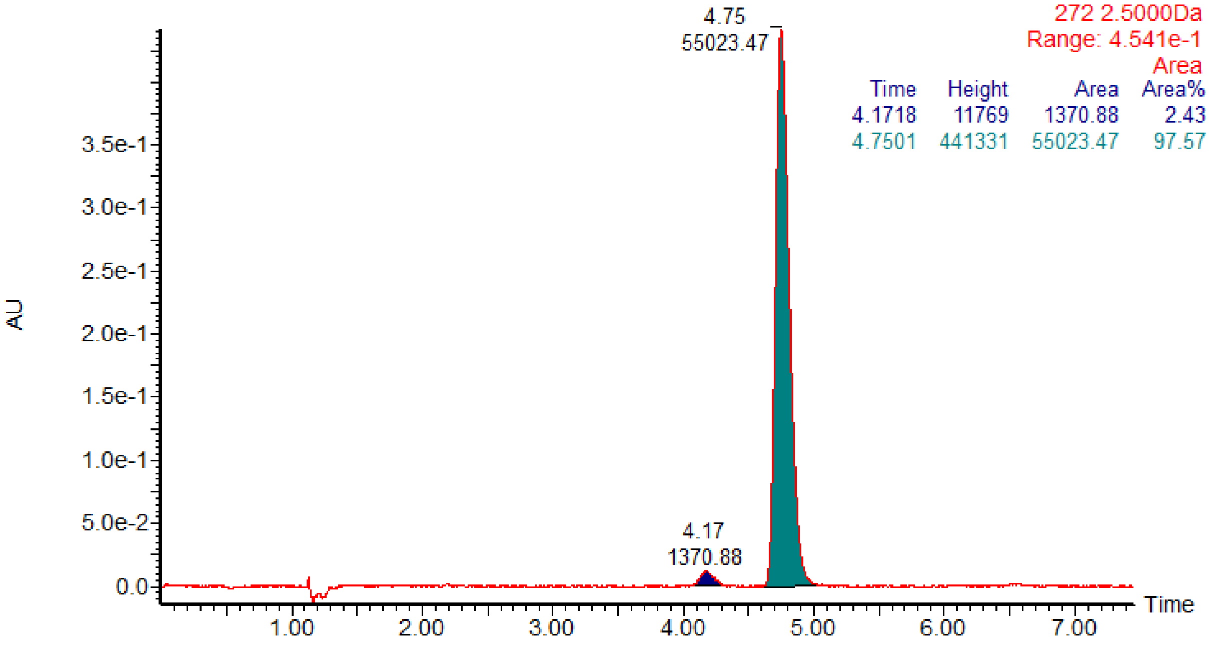

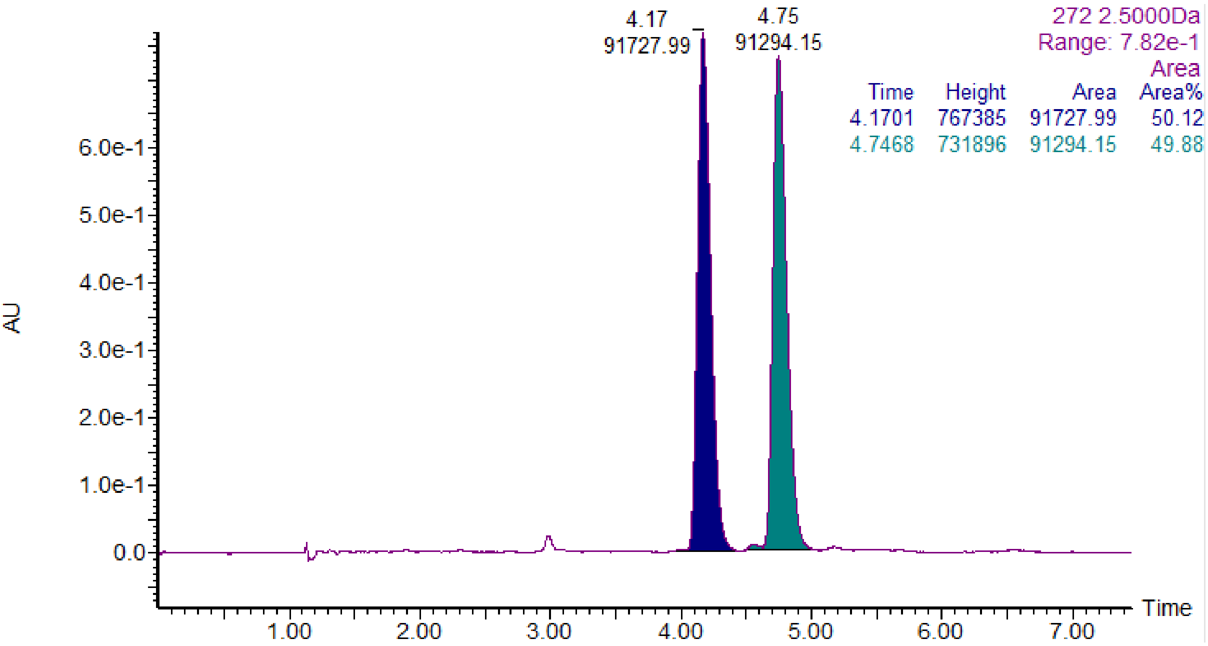

(*S*)-*N*-(1-(4-methylquinolin-2-yl)-2-(4-(trifluoromethyl)phenyl)ethyl)acetamide (5)

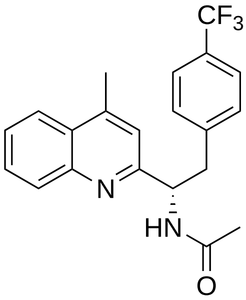

Chiralpak SC (CO_2/MeOH = 85/15, 2.5 mL min^{-1}, 40 °C, 269 nm)

91% ee

t_R = 2.7 minutes (minor), 3.0 minutes (major)

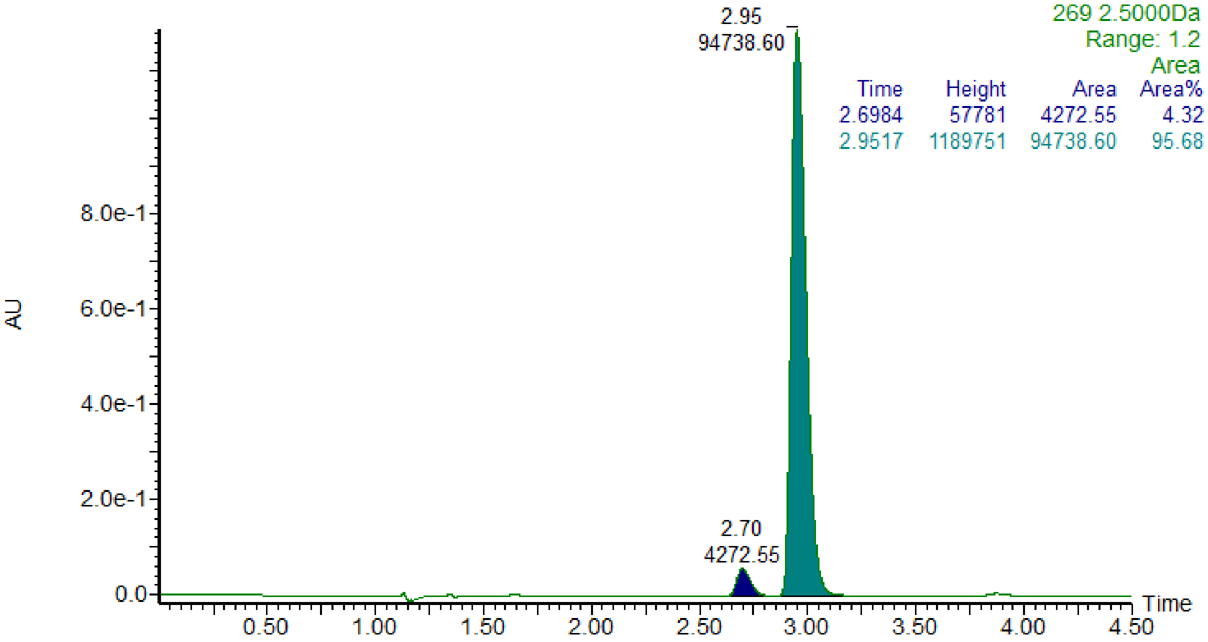

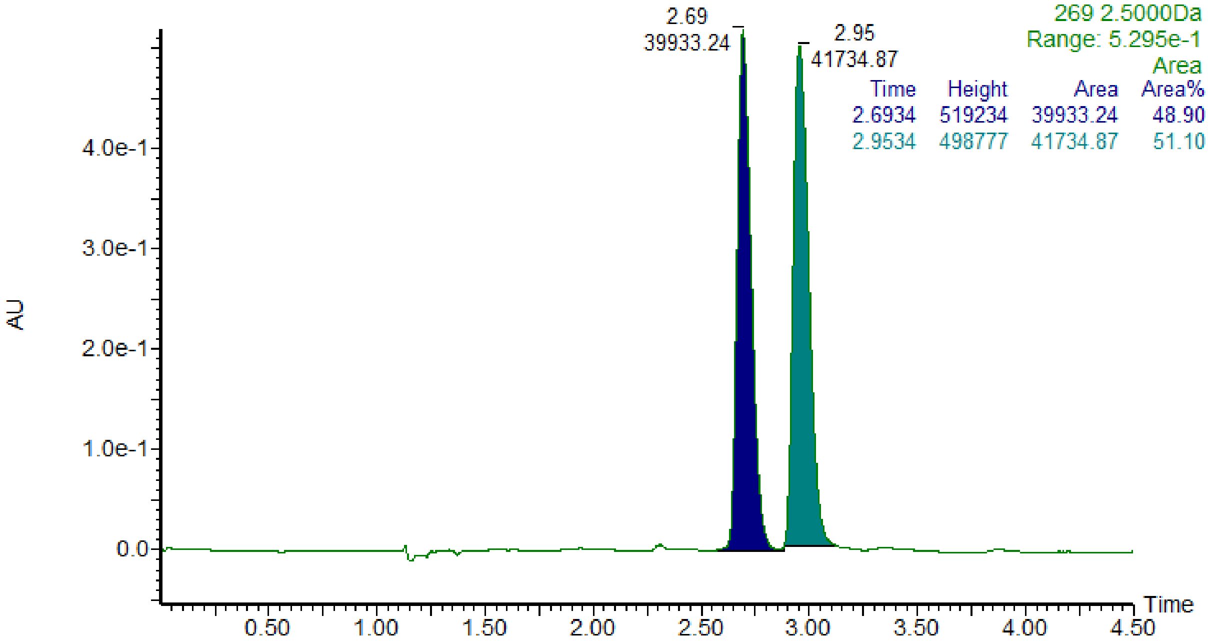

(*S*)-*N*-(2-(3-bromophenyl)-1-(4-methylquinolin-2-yl)ethyl)acetamide (7)

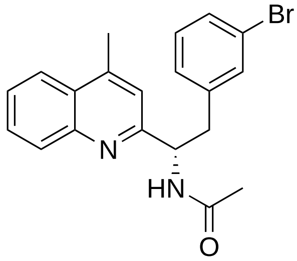

Chiralpak SC (CO_2/MeOH = 85/15, 2.5 mL min^{-1}, 40 °C, 275 nm)

94% ee

t_R = 6.4 minutes (minor), 7.4 minutes (major)

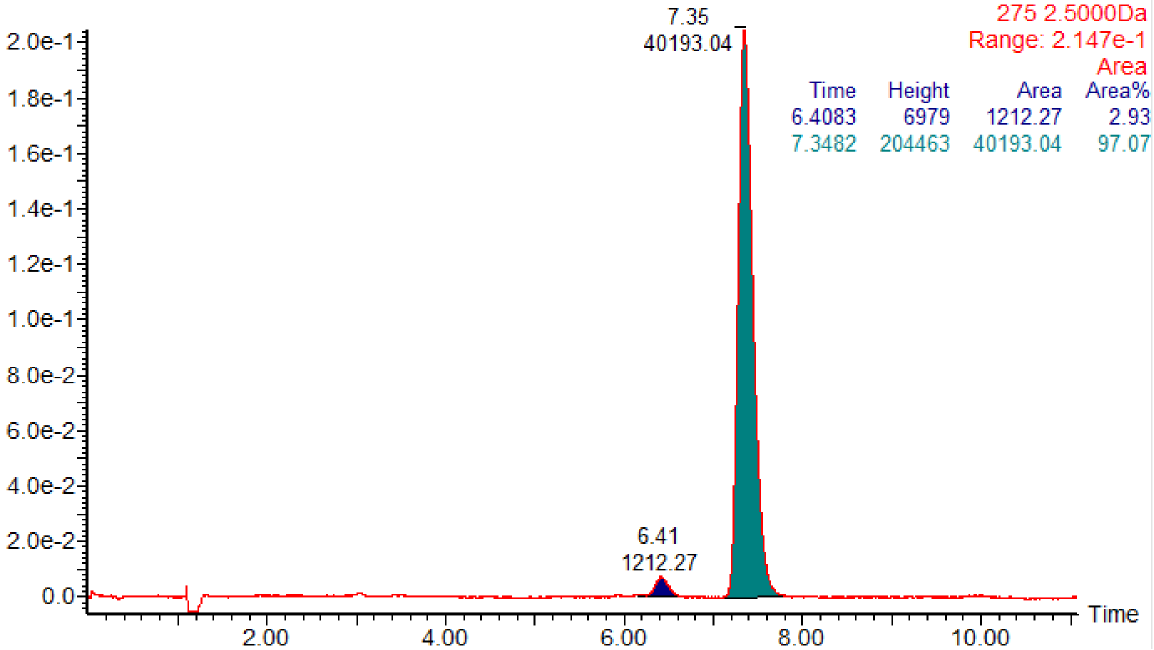

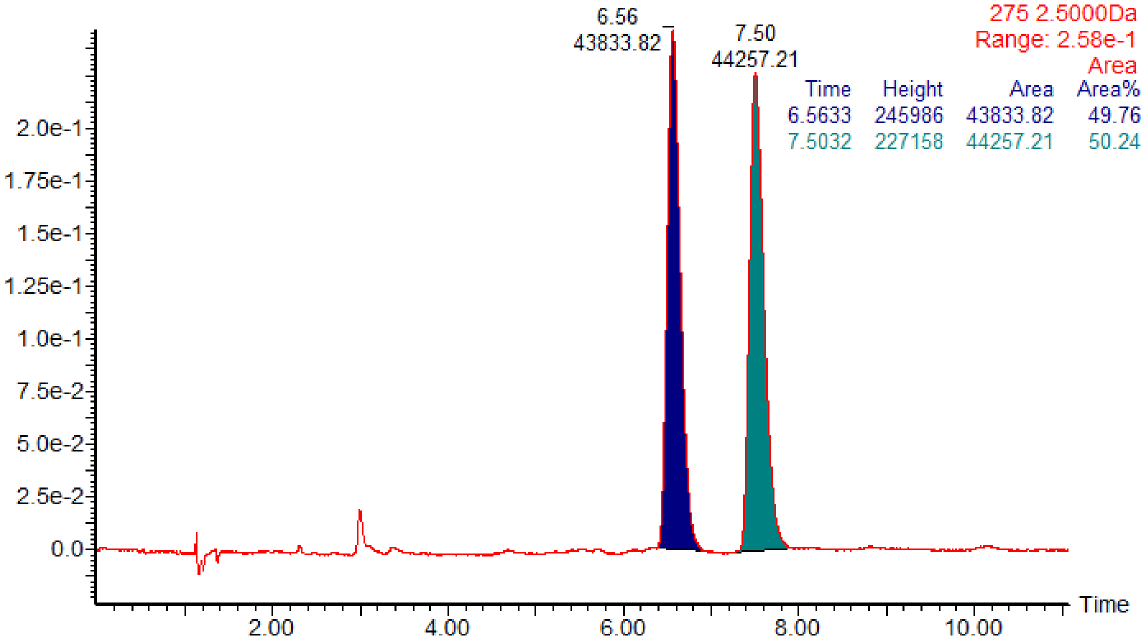

(*S*)-*N*-(2-(3,4-dichlorophenyl)-1-(4-methylquinolin-2-yl)ethyl)acetamide (8)

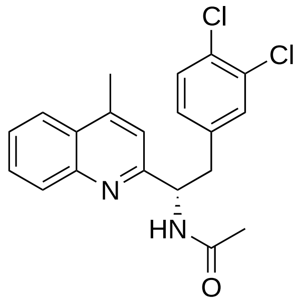

Chiralpak SC (CO_2/MeOH = 85/15, 2.5 mL min^{-1}, 40 °C, 273 nm)

98% ee

t_R = 6.8 minutes (minor), 7.8 minutes (major)

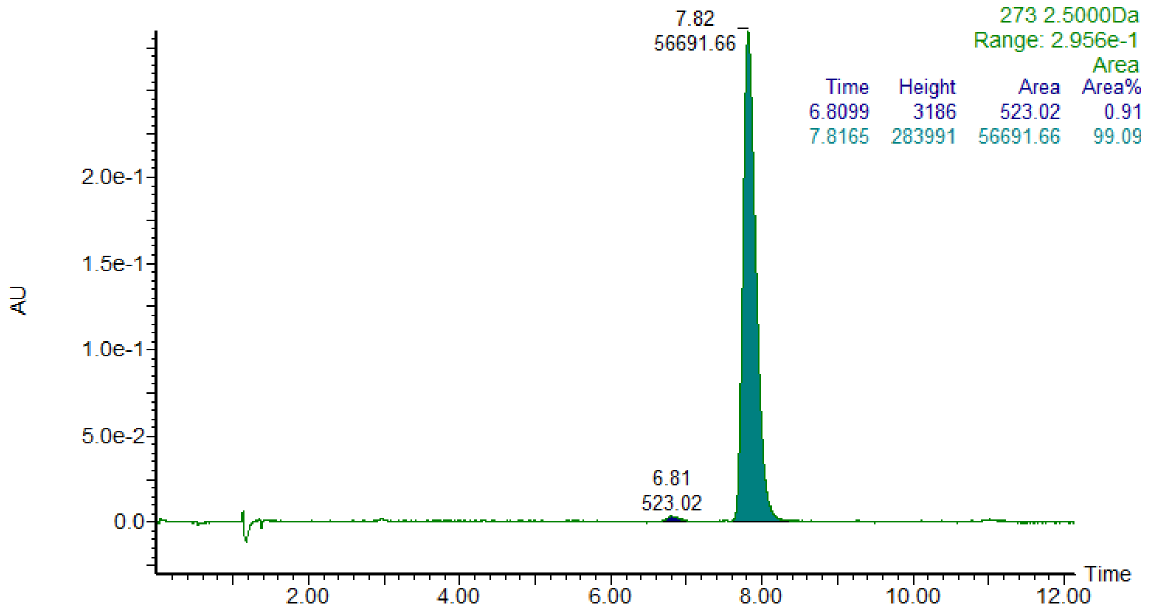

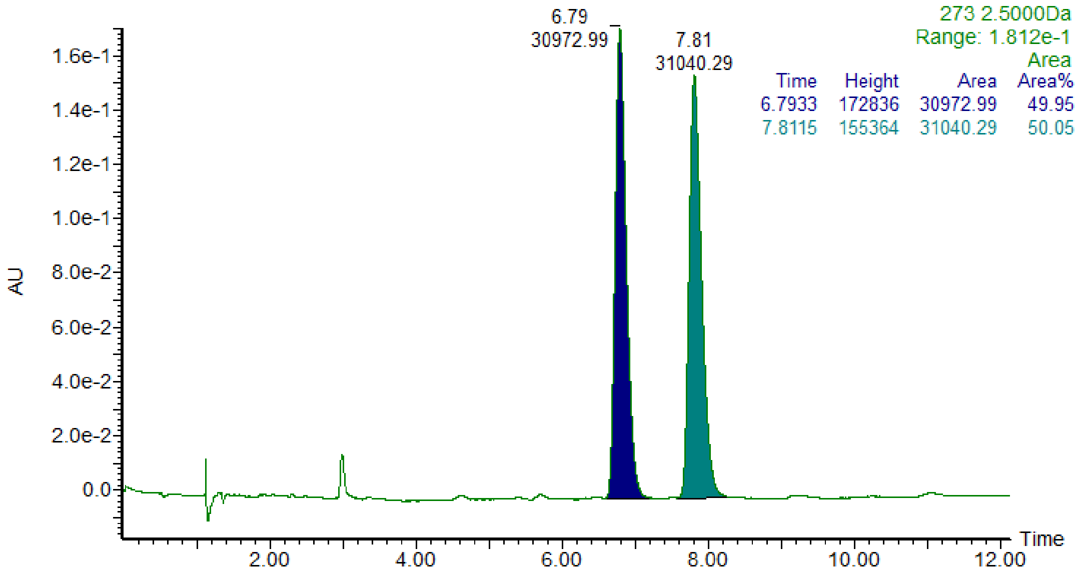

(*S*)-*N*-(1-(4-methylquinolin-2-yl)-3-phenylpropyl)acetamide (9)

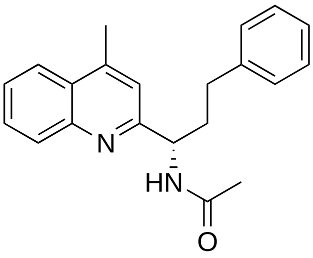

Chiralpak IC (Hexane/^{i}PrOH = 70/30, 1.0 mL min^{-1}, 30 °C, 223 nm)

87% ee

t_R = 8.7 minutes (minor), 11.5 (major) minutes

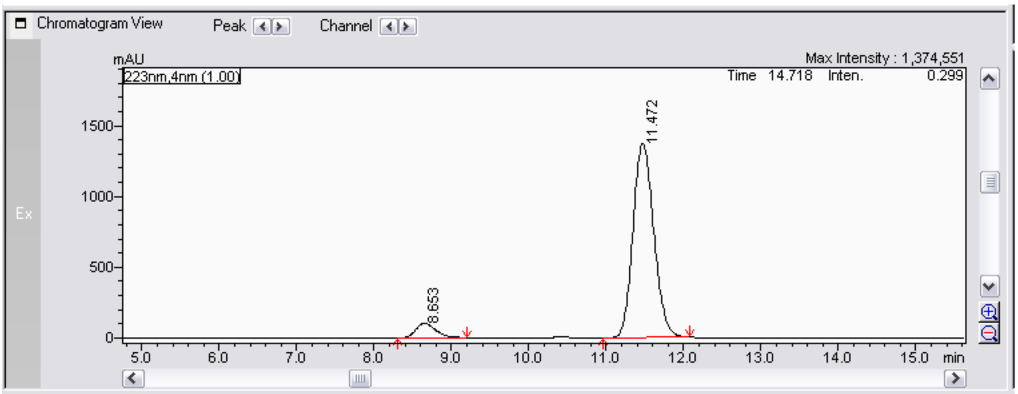

Results View - Peak Table

Peak Table | Compound | Group | Calibration Curve

Peak#	Ret. Time	Area	Height	Area%
1	8.653	1833588	102129	6.667
2	11.472	25667194	1372150	93.333
Total		27500782	1474279	100.000

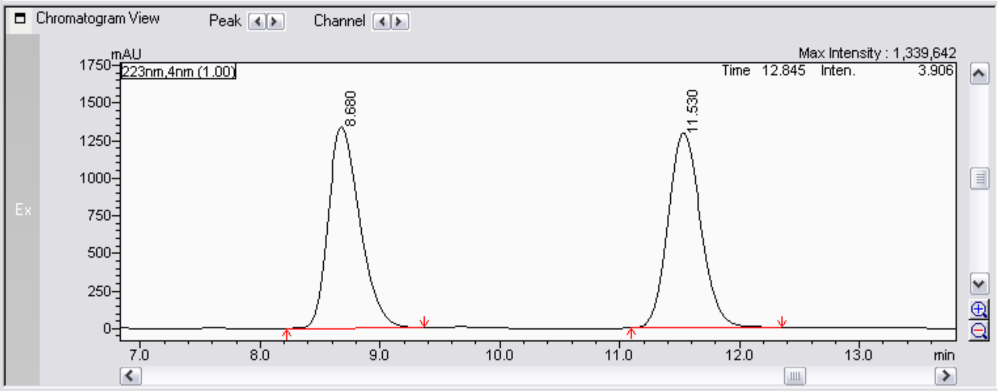

Results View - Peak Table

Peak Table | Compound | Group | Calibration Curve

Peak#	Ret. Time	Area	Height	Area%
1	8.680	24579702	1336126	49.961
2	11.530	24617650	1295780	50.039
Total		49197352	2631906	100.000

(*S*)-*N*-(1-(4-methylquinolin-2-yl)-4-phenylbutyl)acetamide (10)

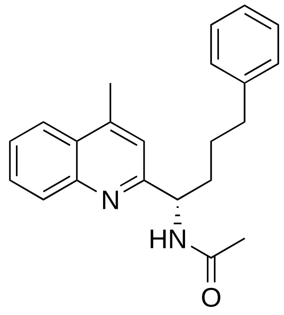

Chiralpak SC (CO_2/MeOH = 85/15, 2.5 mL min^{-1}, 40 °C, 273 nm)

<u>86% ee</u>

t_R = 6.5 minutes (minor), 7.8 minutes (major)

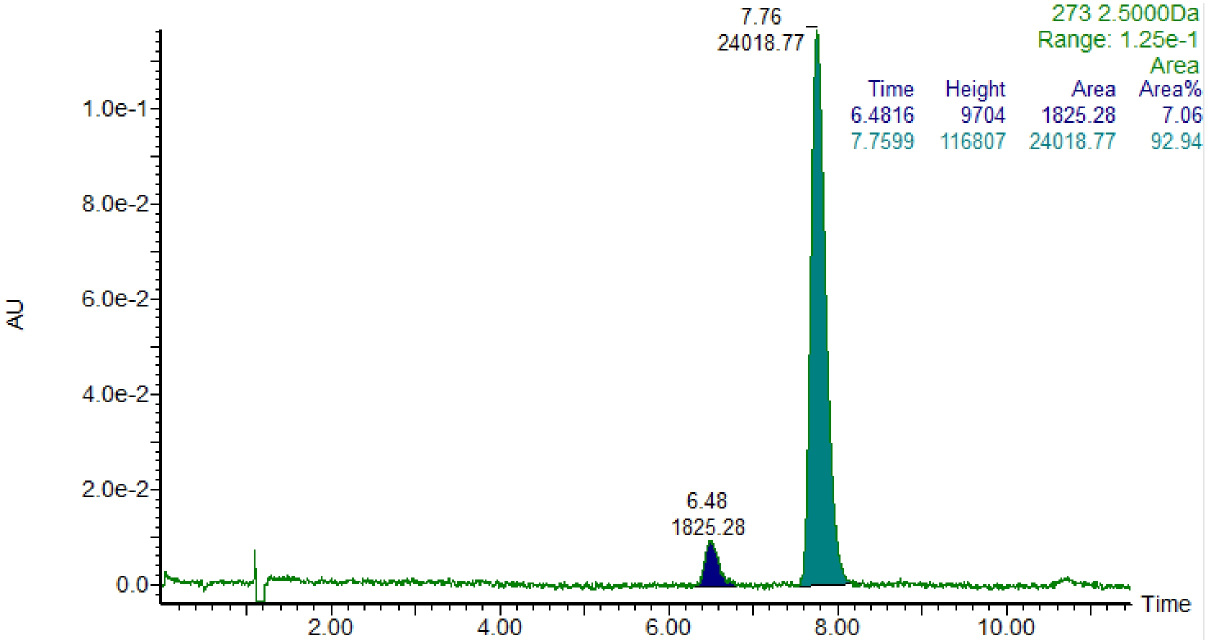

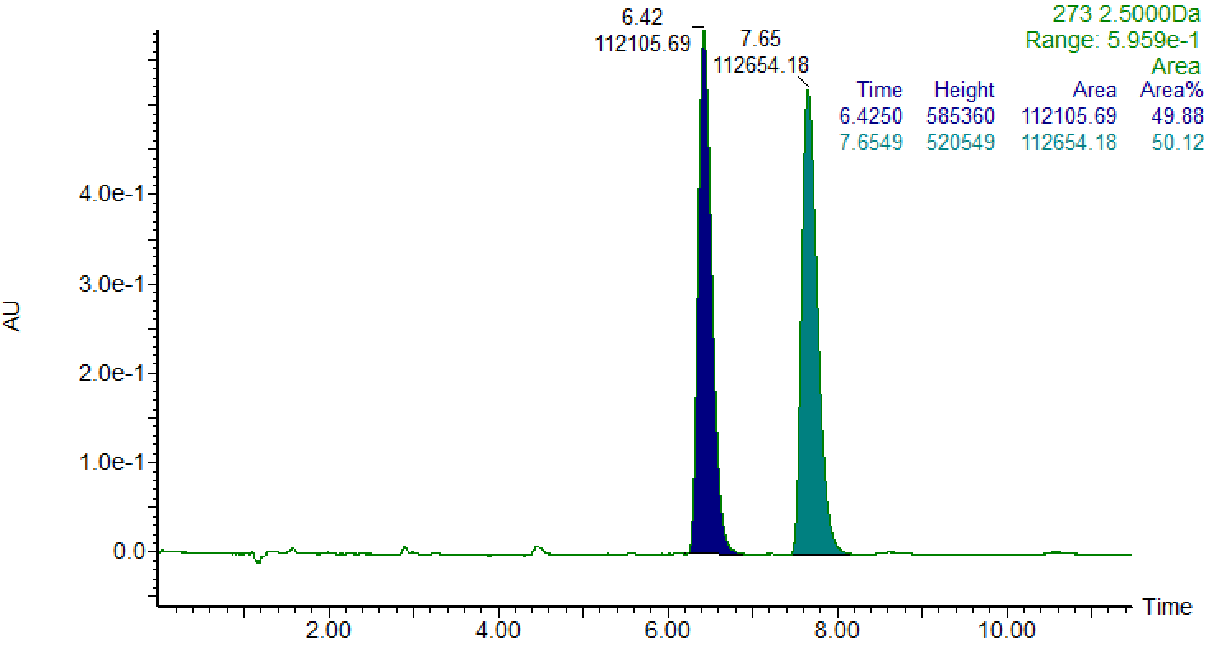

(*S*)-*N*-(1-(4-methylquinolin-2-yl)ethyl)acetamide (11)

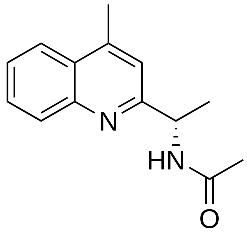

Chiralpak IC (Hexane/^{i}PrOH = 70/30, 1.0 mL min^{-1}, 30 °C, 236 nm)

86% ee

t_R = 10.6 minutes (minor), 13.1 (major) minutes

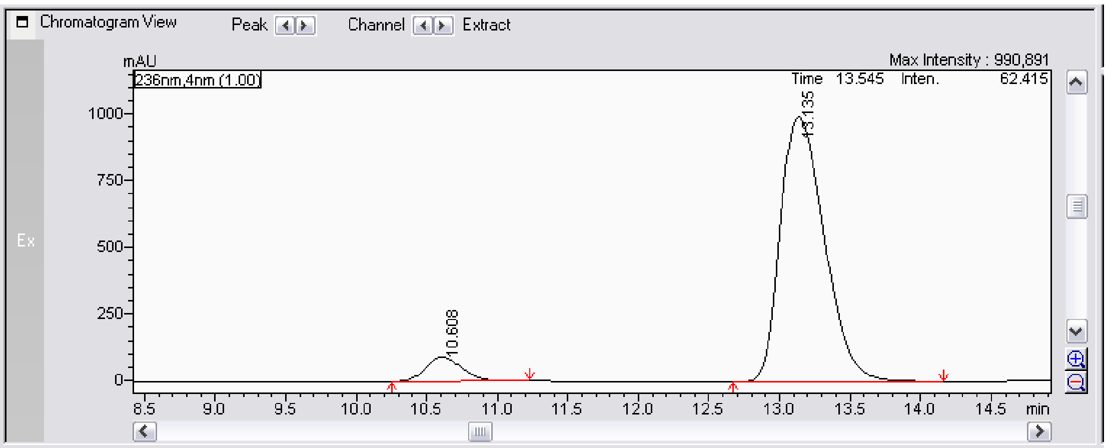

Results View - Peak Table

Peak#	Ret. Time	Area	Height	Area%
1	10.608	1607540	90873	6.963
2	13.135	21478059	994439	93.037
Total		23085599	1085313	100.000

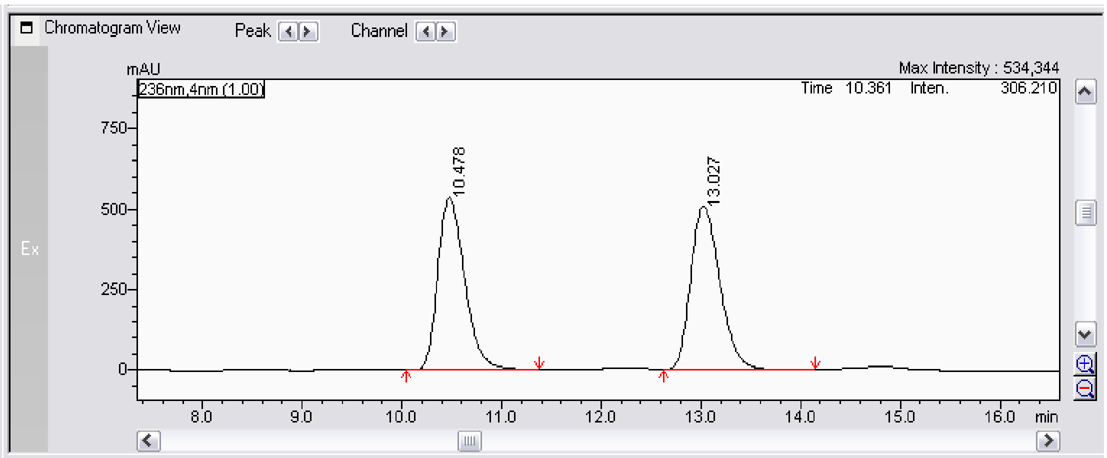

Results View - Peak Table

Peak#	Ret. Time	Area	Height	Area%
1	10.478	10076376	534208	49.295
2	13.027	10364535	507366	50.705
Total		20440911	1041574	100.000

(*S*)-*N*-(1-(4-methylquinolin-2-yl)nonyl)acetamide (12)

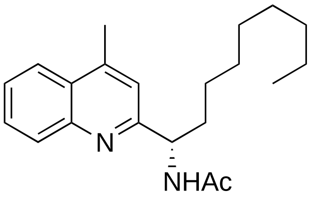

Chiralpak SC (CO_2/MeOH = 90/10, 2.5 mL min^{-1}, 40 °C, 210 nm)

85% ee

t_R = 6.2 minutes (minor), 7.3 minutes (major)

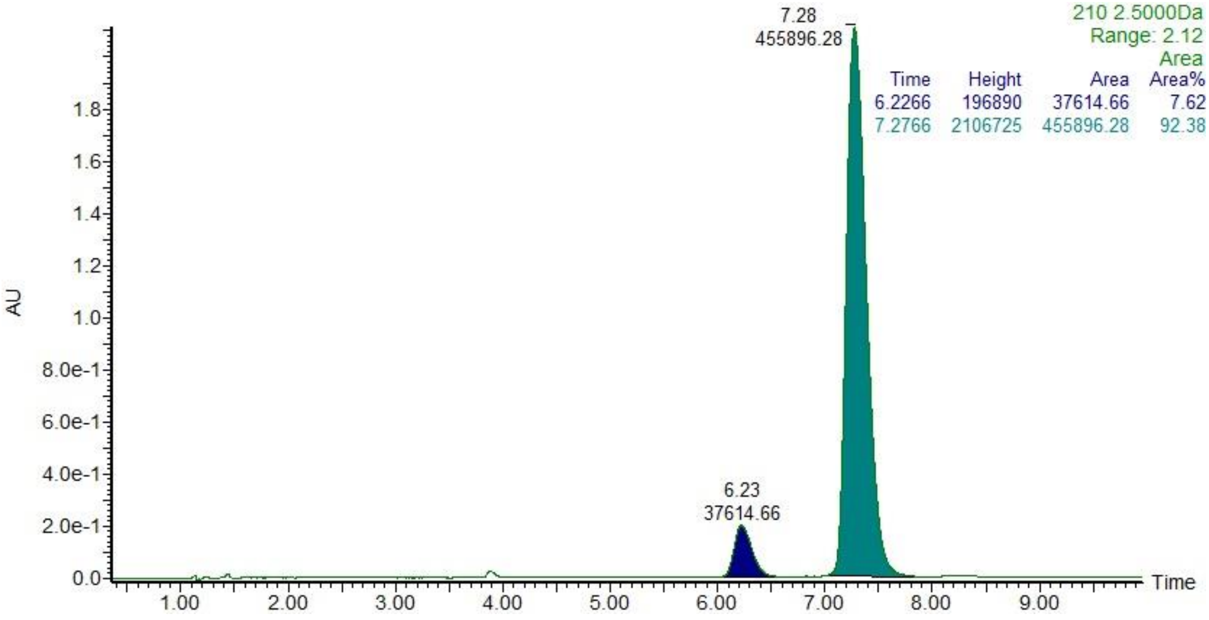

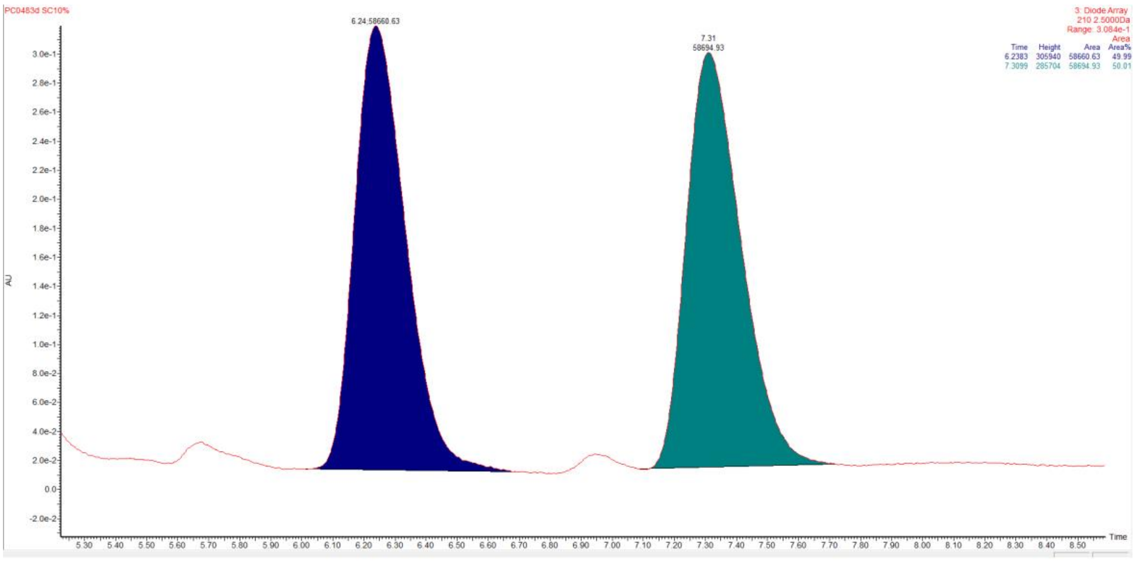

(*S*)-*N*-(2-methyl-1-(4-methylquinolin-2-yl)propyl)acetamide (13)

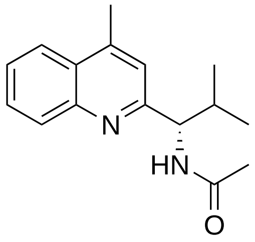

Chiralpak IC (Hexane/^{i}PrOH = 70/30, 1.0 mL min^{-1}, 30 °C, 212 nm)

97% ee

t_R = 7.1 minutes (minor), 10.3 (major) minutes

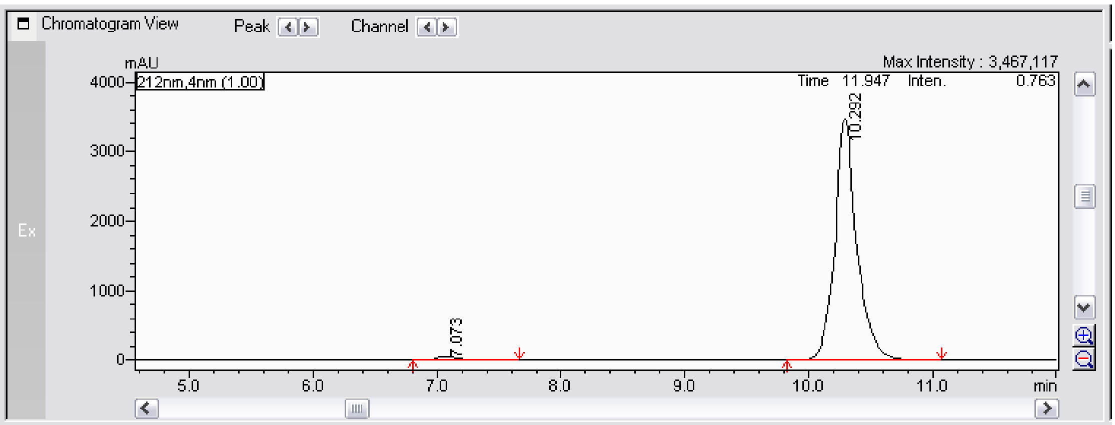

Results View - Peak Table

Peak Table | Compound | Group | Calibration Curve

Peak#	Ret. Time	Area	Height	Area%
1	7.073	602316	50762	1.371
2	10.292	43318735	3466147	98.629
Total		43921050	3516908	100.000

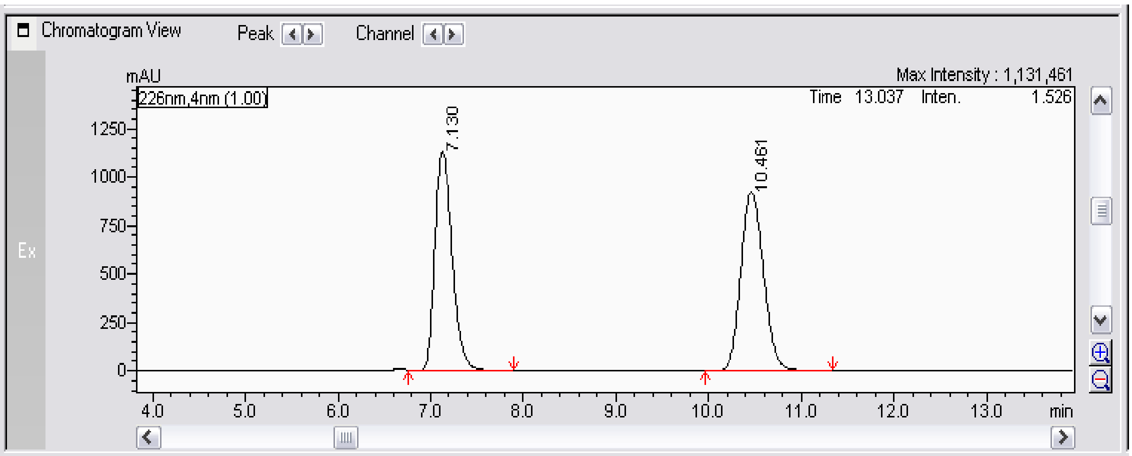

Results View - Peak Table

Peak Table | Compound | Group | Calibration Curve

Peak#	Ret. Time	Area	Height	Area%
1	7.130	14770696	1128130	48.549
2	10.461	15653903	922030	51.451
Total		30424600	2050160	100.000

(*S*)-*N*-(2-methyl-1-(4-methylquinolin-2-yl)propyl)acetamide (15)

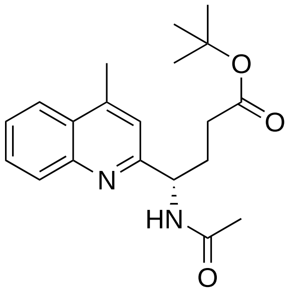

Chiralpak IC (Hexane/^{i}PrOH = 70/30, 1.0 mL min^{-1}, 30 °C, 228 nm)

97% ee

t_R = 7.1 minutes (minor), 10.3 (major) minutes

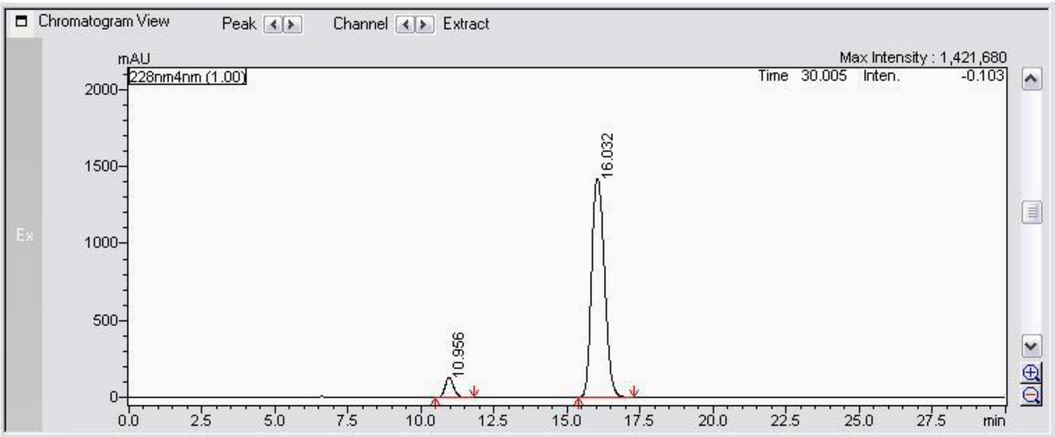

Results View - Peak Table

Peak Table | Compound | Group | Calibration Curve

Peak#	Ret. Time	Area	Height	Area%
1	10.956	2633617	131022	5.703
2	16.032	43549085	1420471	94.297
Total		46182702	1551493	100.000

Extracted 228nm | Ch1 254nm

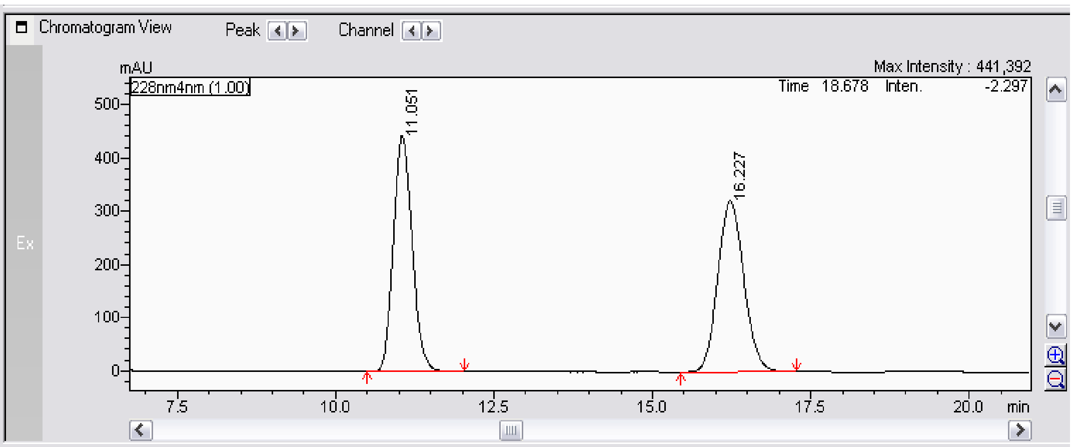

Results View - Peak Table

Peak Table | Compound | Group | Calibration Curve

Peak#	Ret. Time	Area	Height	Area%
1	11.051	9106417	442450	49.702
2	16.227	9215619	321234	50.298
Total		18322037	763684	100.000

Methyl (2*S*,6*S*)-6-acetamido-2-((*t*-butoxycarbonyl)amino)-6-(4-methylquinolin-2-yl)hexanoate (16)

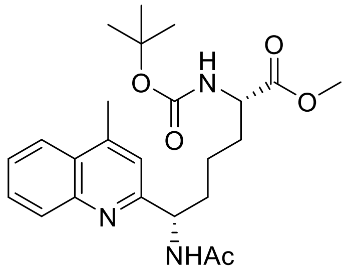

Chiralpak SC (CO_2/MeOH = 80/20, 2.5 mL min^{-1}, 40 °C, 224 nm)

23:1 d.r.

t_R = 6.1 minutes (minor), 7.2 minutes (major)

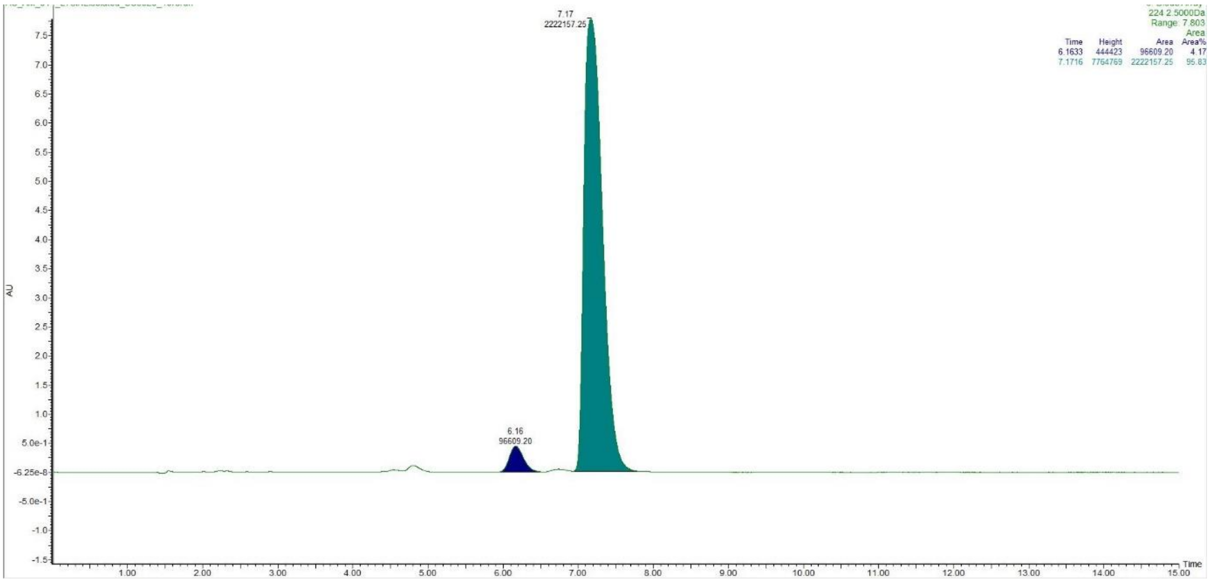

Reaction carried out using TFA instead of DIP

1.4:1 d.r.

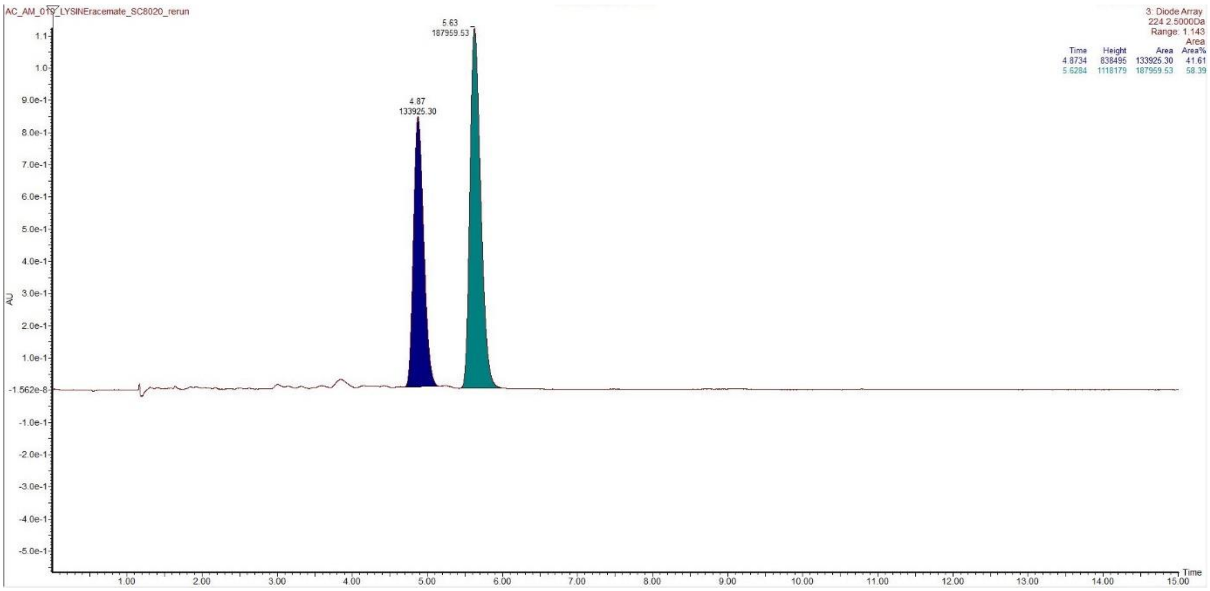

(*S*)-*N*-(2-phenyl-1-(quinolin-2-yl)ethyl)acetamide (17)

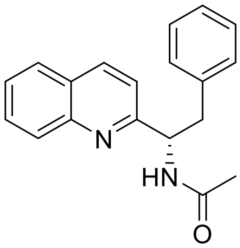

Chiralpak IG (CO_2/MeOH = 85/15, 2.5 mL min^{-1}, 40 °C, 210 nm)

95% ee

t_R = 8.1 minutes (minor), 9.1minutes (major)

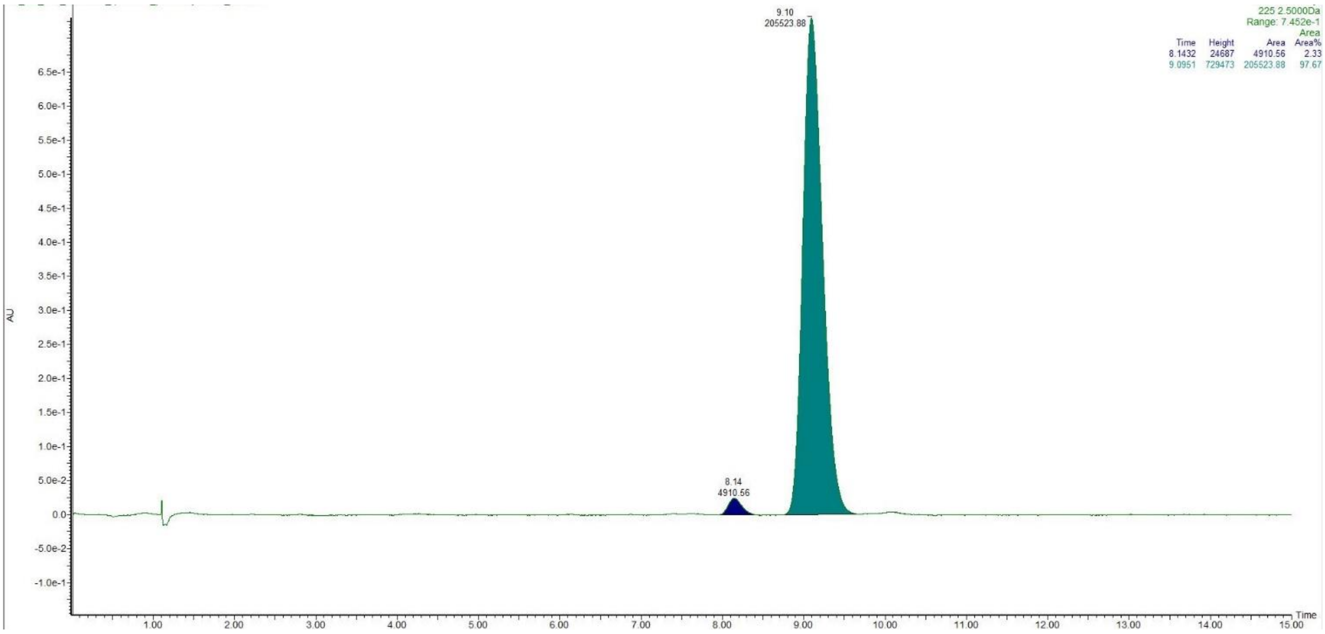

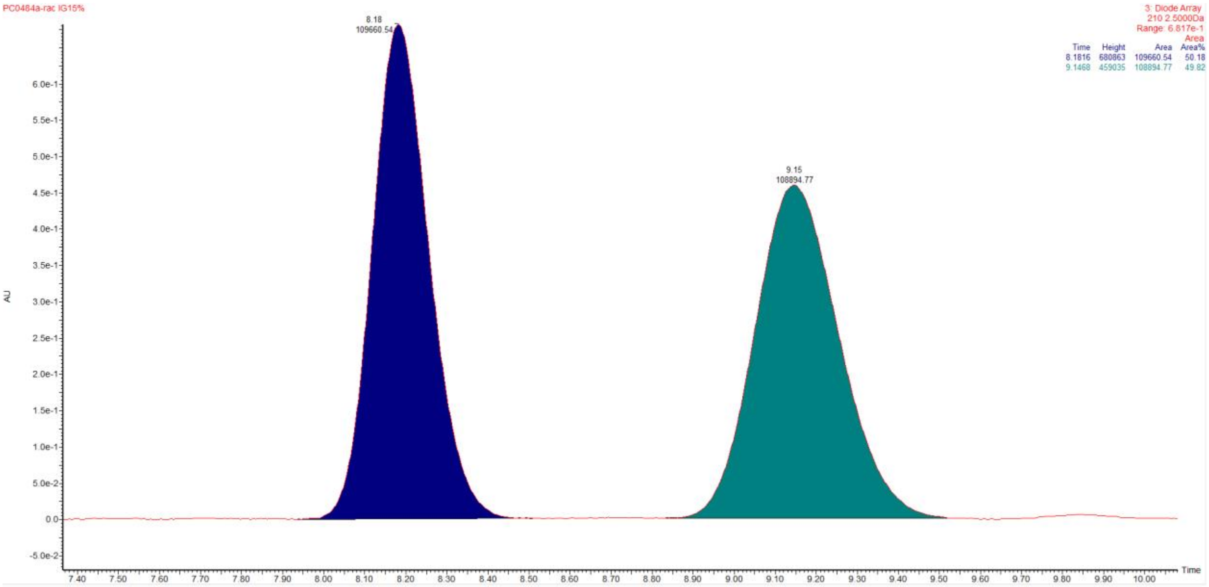

(S)-*N*-(1-(6-methoxyquinolin-2-yl)-2-phenylethyl)acetamide (18)

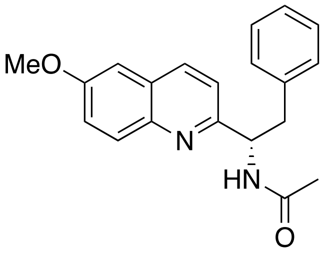

Chiralpak SC (CO_2/MeOH = 90/10, 2.5 mL min^{-1}, 40 °C, 240 nm)

90% ee

t_R = 12.5 minutes (minor), 13.2 minutes (major)

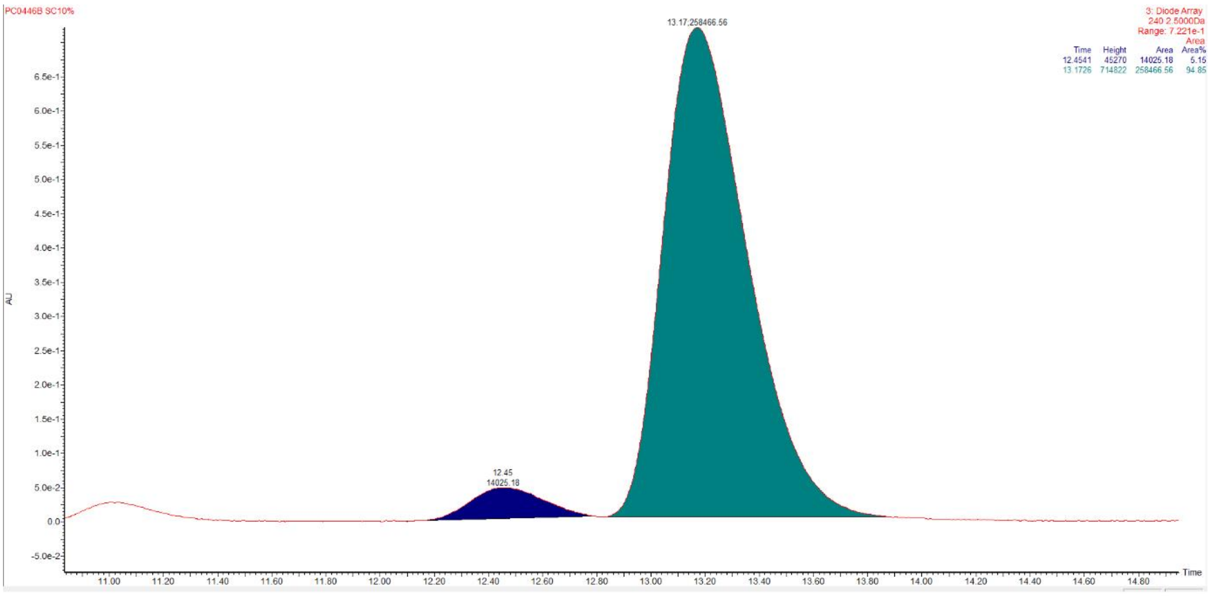

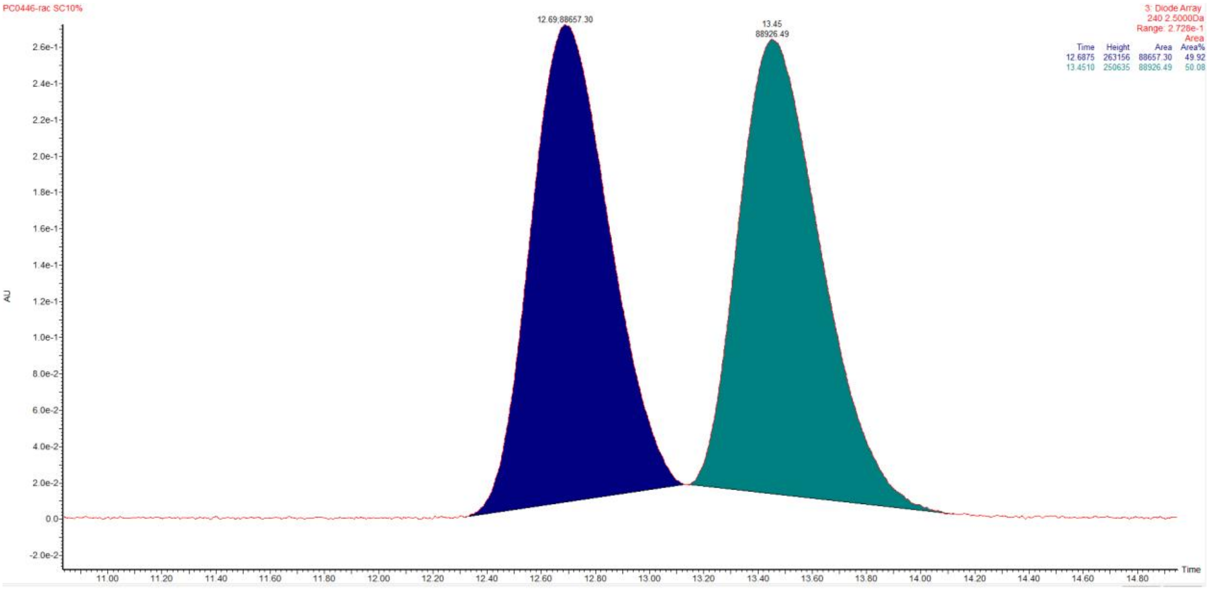

(S)-*N*-(1-(6-chloroquinolin-2-yl)-2-phenylethyl)acetamide (19)

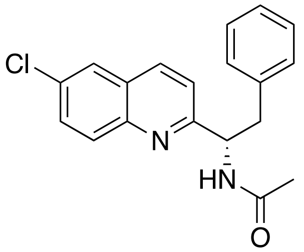

Chiralpak SC (CO_2/MeOH = 90/10, 2.5 mL min^{-1}, 40 °C, 240 nm)

<u>90% ee</u>

t_R = 10.3 minutes (minor), 11.0 minutes (major)

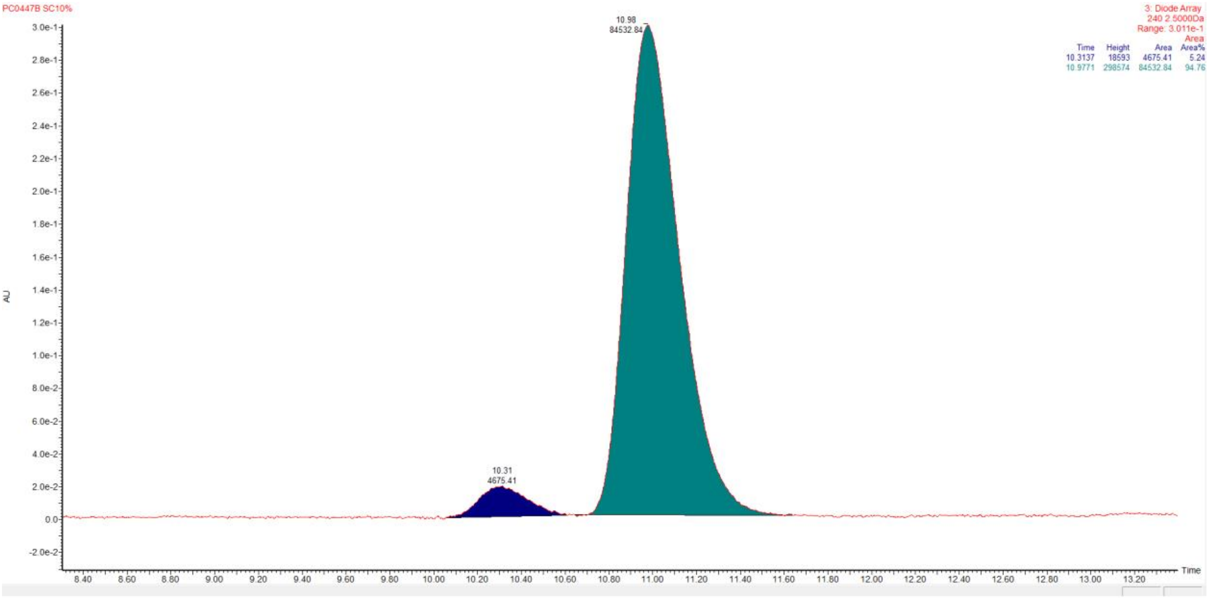

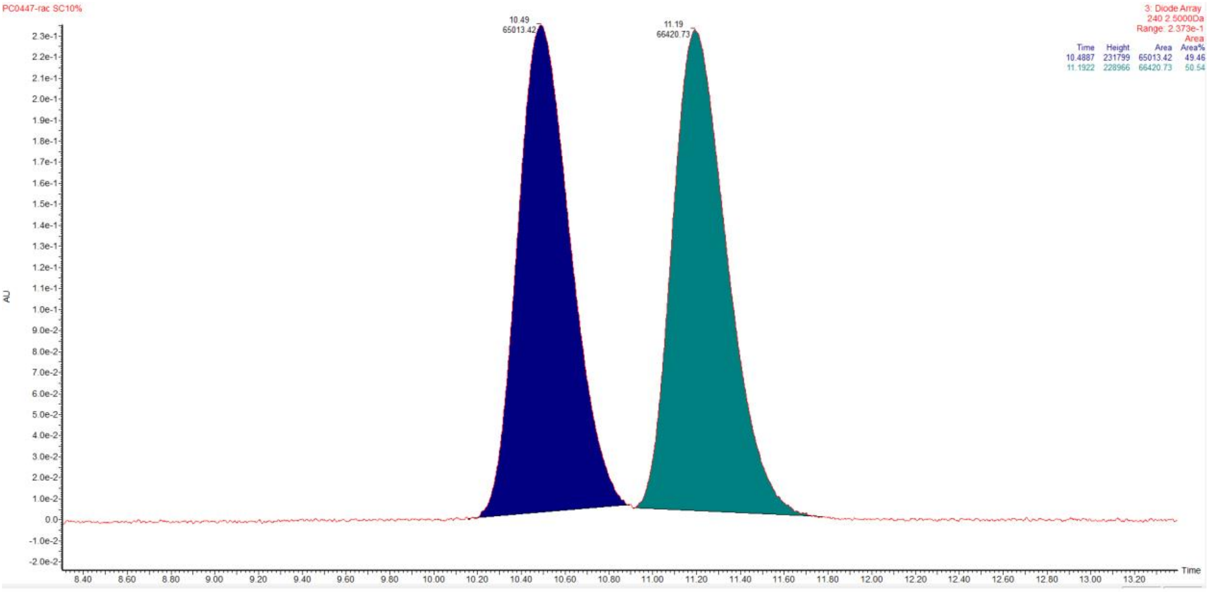

(S)-*N*-(1-(3-methylquinolin-2-yl)-2-phenylethyl)acetamide (20)

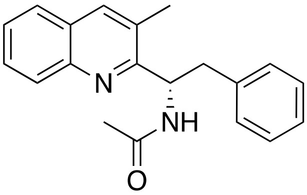

Chiralpak SC (CO_2/MeOH = 90/10, 2.5 mL min^{-1}, 40 °C, 210 nm)

<u>93% ee</u>

t_R = 6.6 minutes (minor), 7.1 minutes (major)

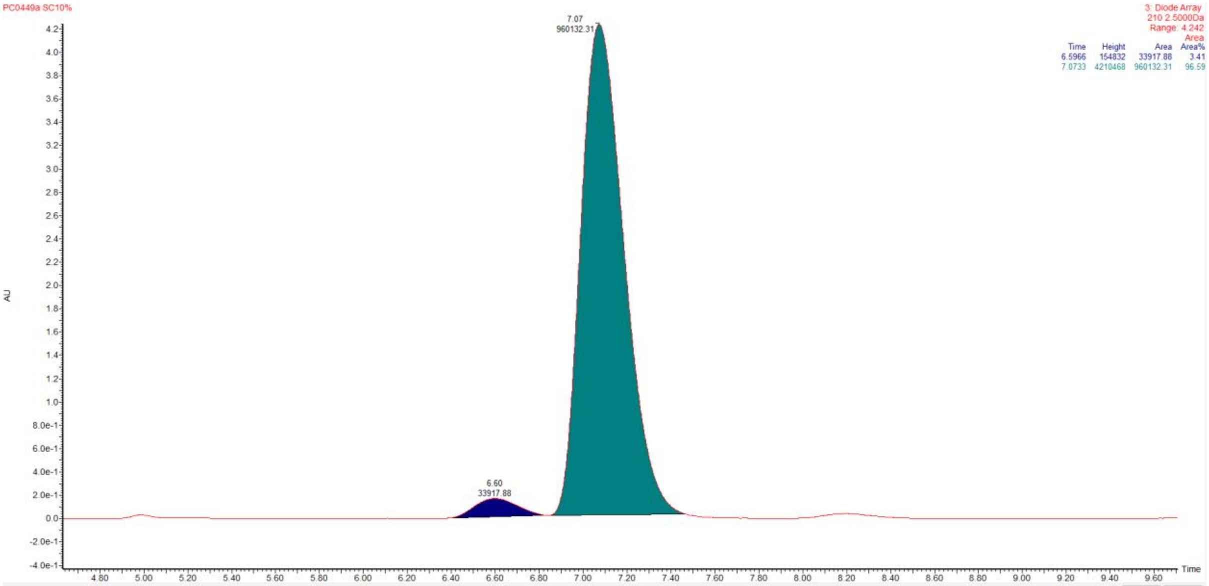

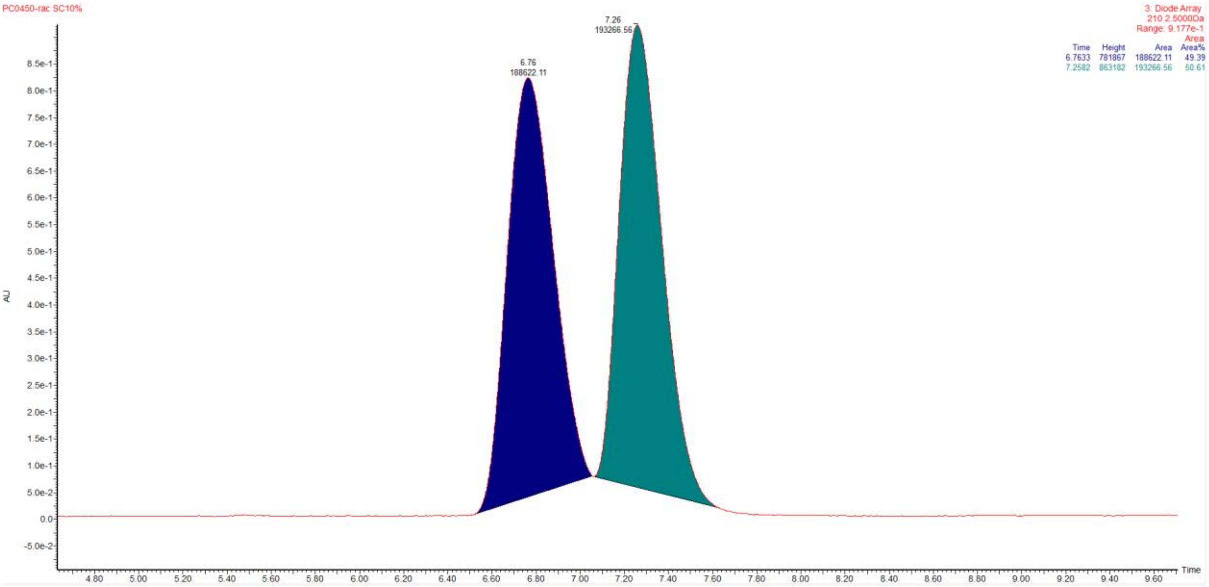

(S)-*N*-(1-(phenanthridin-6-yl)-2-phenylethyl)acetamide (21)

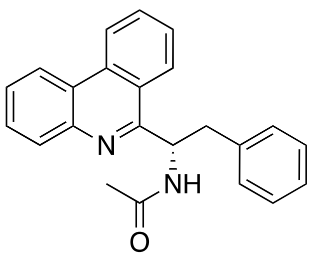

Chiralpak SC (CO_2/MeOH = 85/15, 2.5 mL min^{-1}, 40 °C, 240 nm)

<u>83% ee</u>

t_R = 8.1 minutes (minor), 8.7 minutes (major)

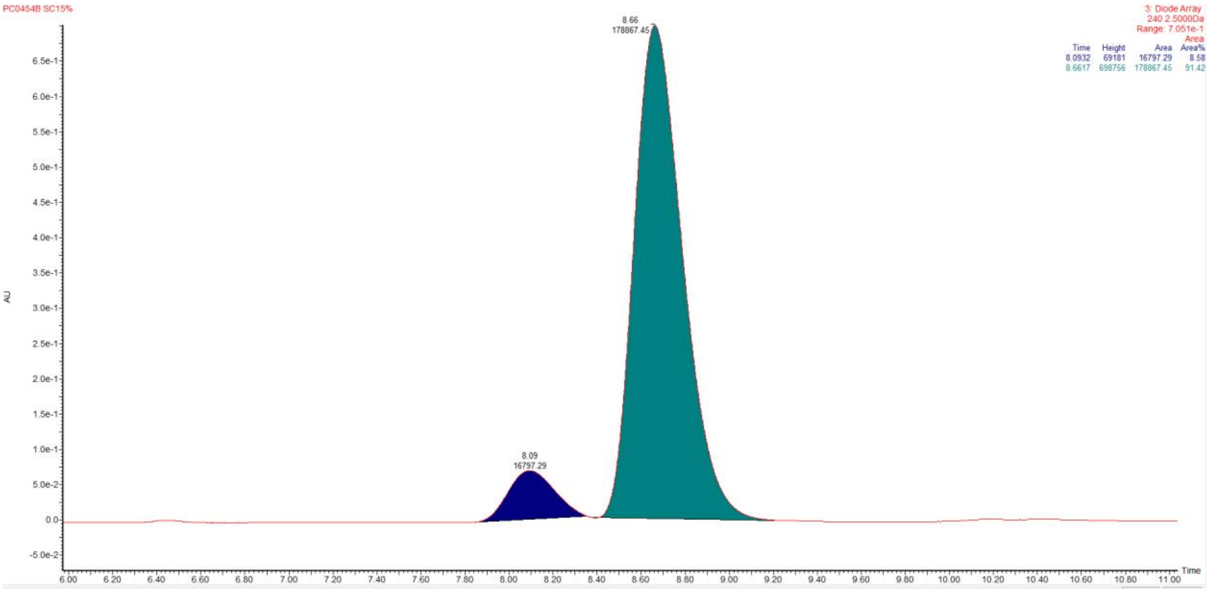

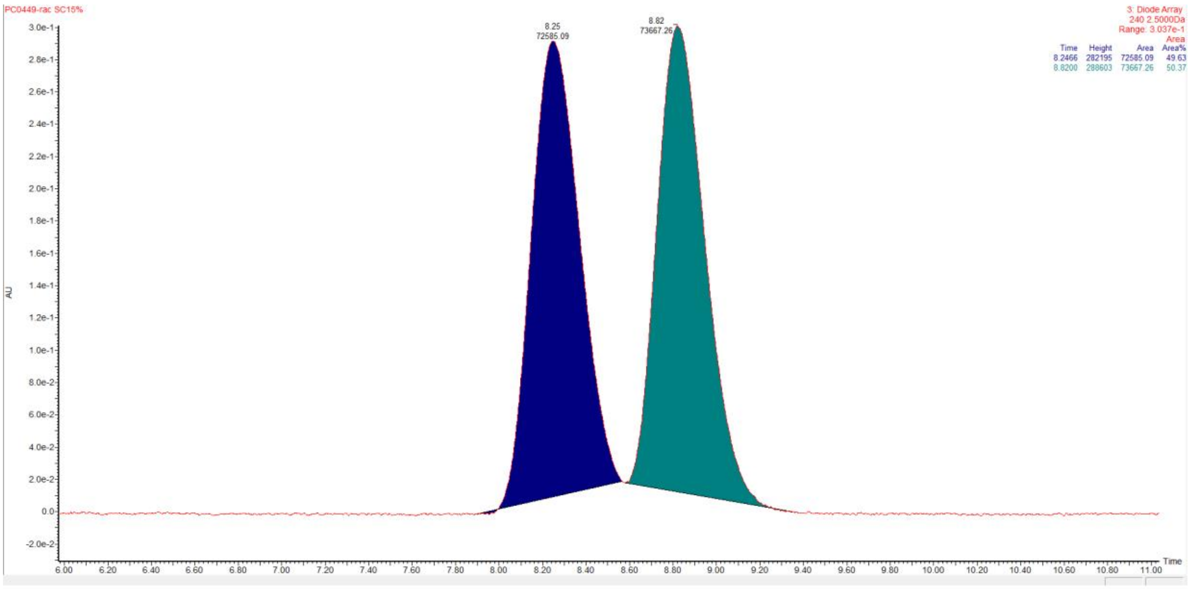

(S)-*N*-(1-(4-(4-bromophenoxy)quinolin-2-yl)-2-phenylethyl)acetamide (22)

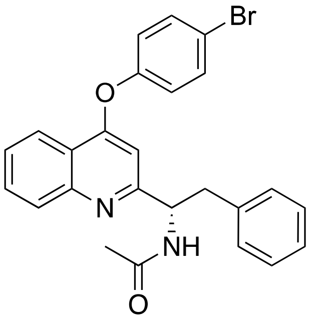

Chiralpak SJ (CO_2/MeOH = 80/20, 2.5 mL min^{-1}, 40 °C, 210 nm)

<u>96% ee</u>

t_R = 2.7 minutes (major), 2.9 minutes (minor)

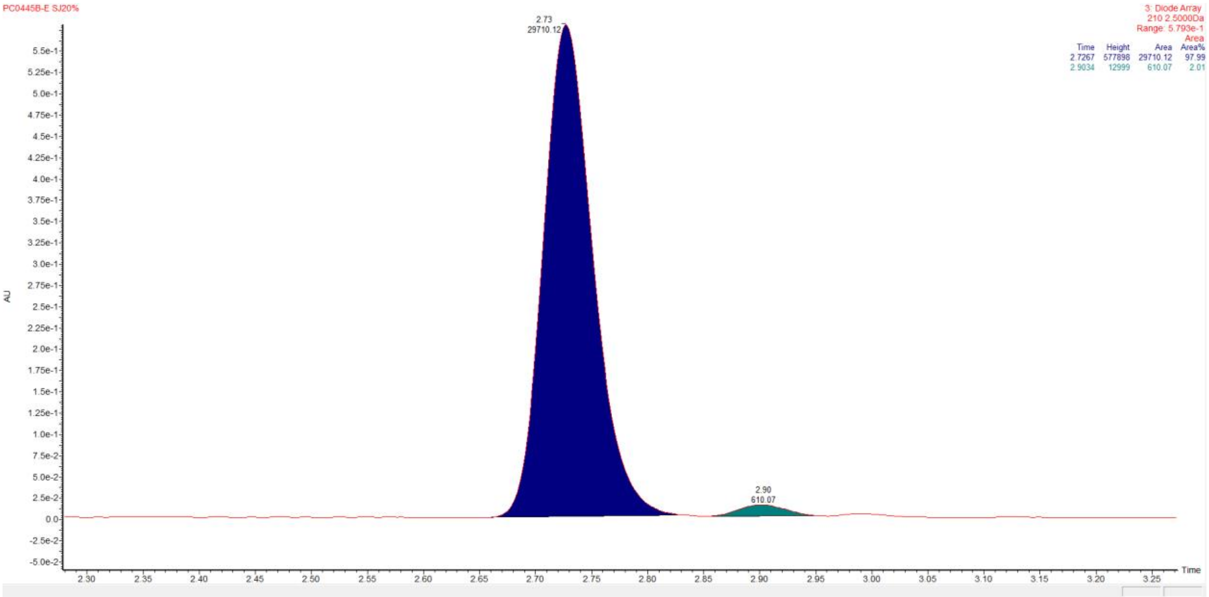

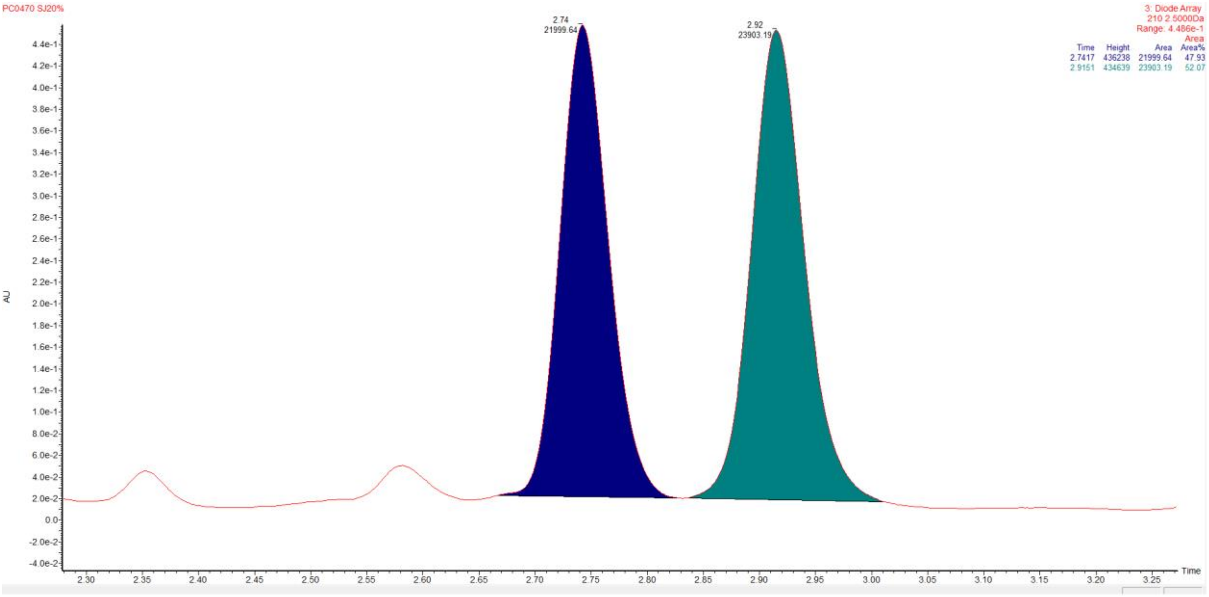

methyl (S)-6-(1-acetamido-2-phenylethyl)-2-methylnicotinate (23)

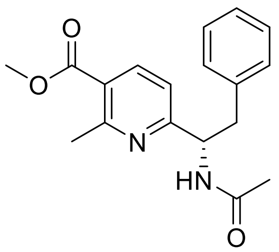

Chiralpak SC (CO_2/MeOH = 90/10, 2.5 mL min^{-1}, 40 °C, 240 nm)

<u>86% ee</u>

t_R = 6.4 minutes (minor), 7.1 minutes (major)

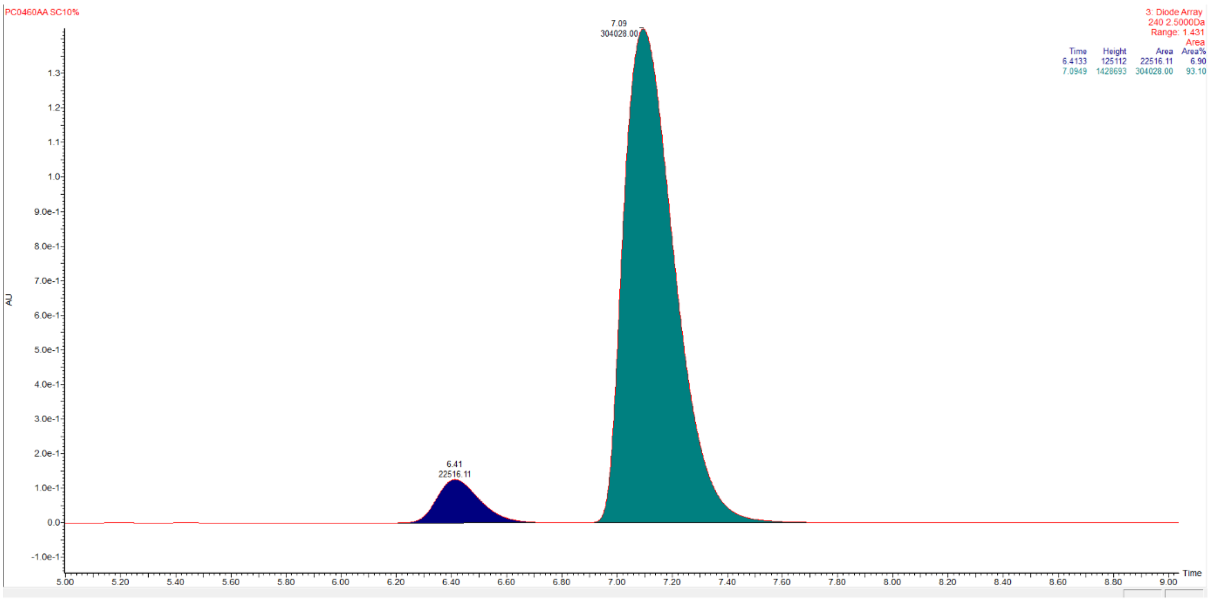

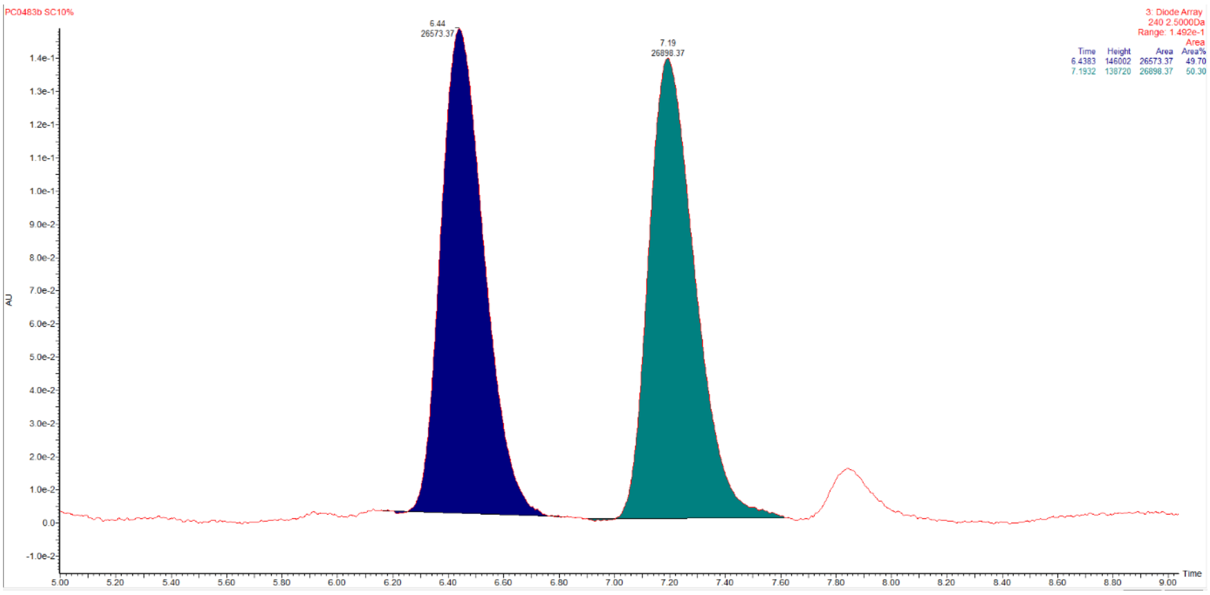

methyl (S)-6-(1-acetamido-2-phenylethyl)nicotinate (24)

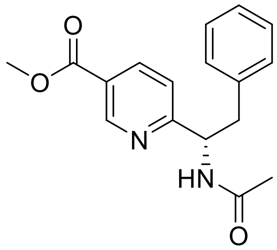

Chiralpak SC (CO_2/MeOH = 93/7, 2.5 mL min^{-1}, 40 °C, 210 nm)

<u>81% ee</u>

t_R = 12.4 minutes (minor), 13.3 minutes (major)

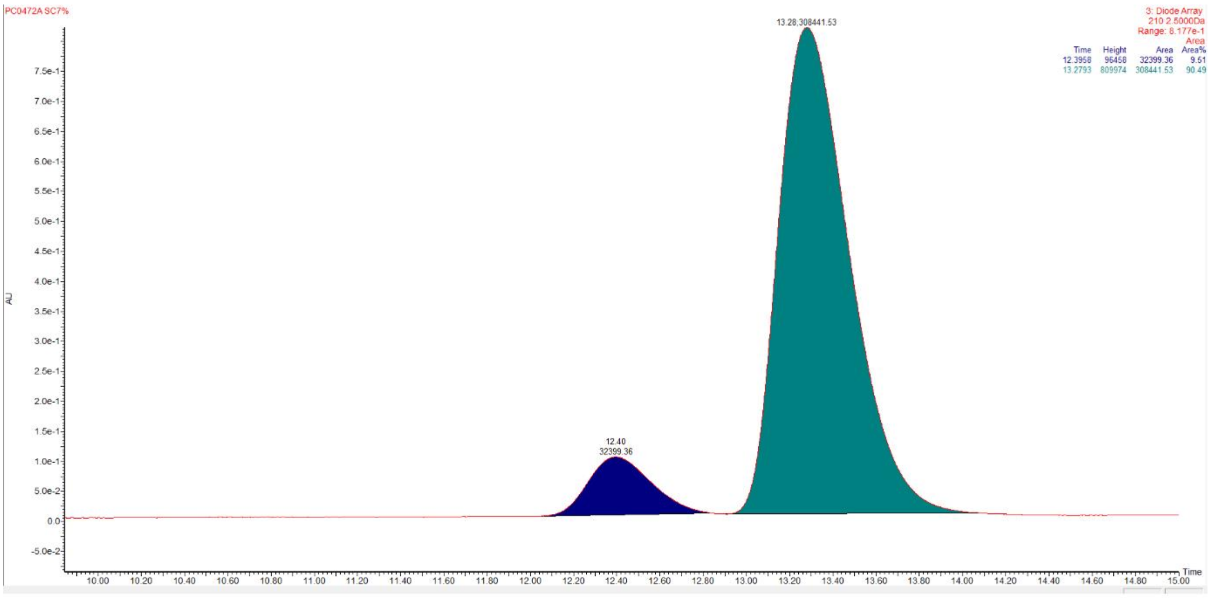

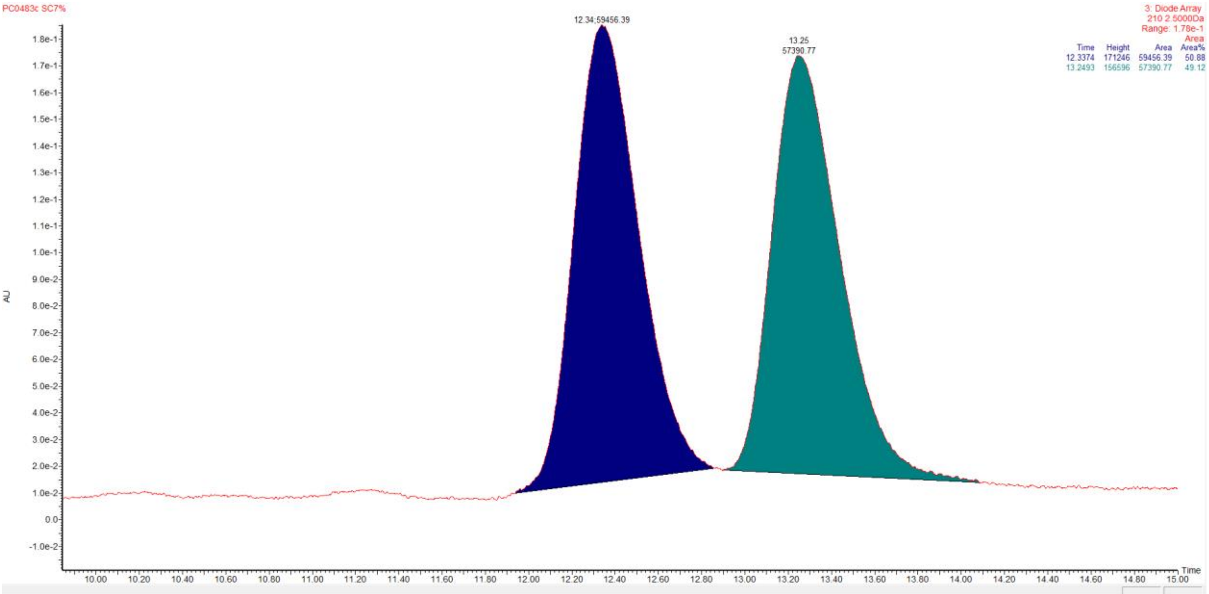

methyl (S)-6-(1-acetamido-2-phenylethyl)-4-methylnicotinate (25)

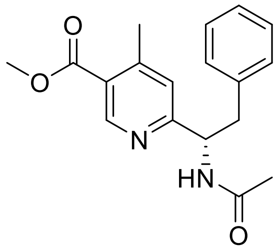

Chiralpak SC (CO_2/MeOH = 93/7, 2.5 mL min^{-1}, 40 °C, 240 nm)

<u>79% ee</u>

t_R = 10.7 minutes (minor), 11.4 minutes (major)

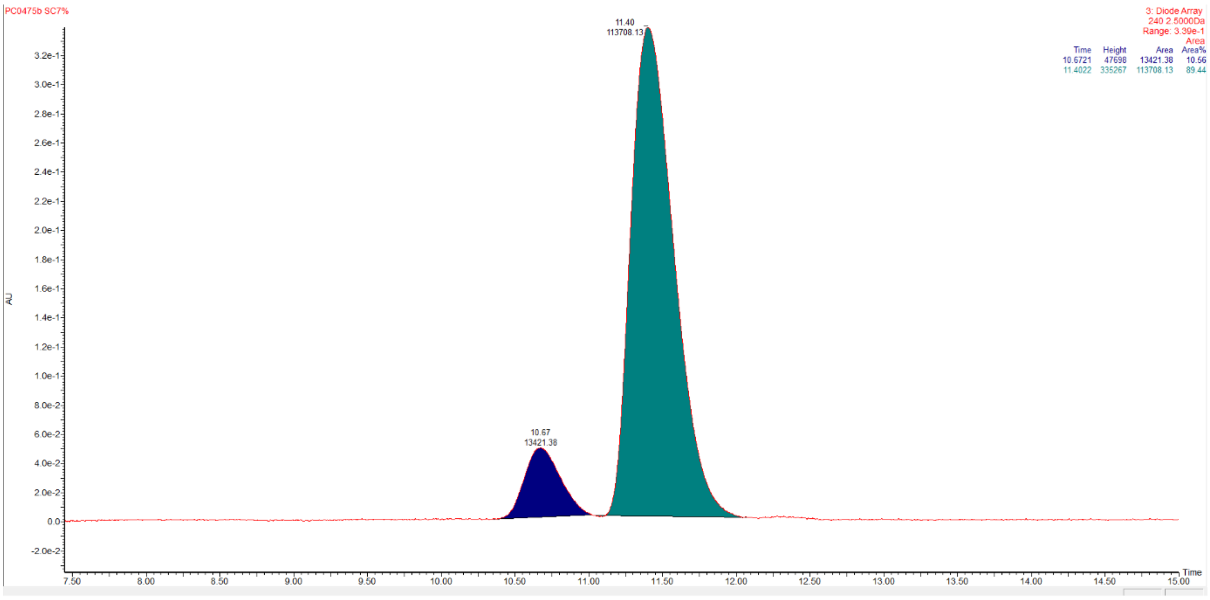

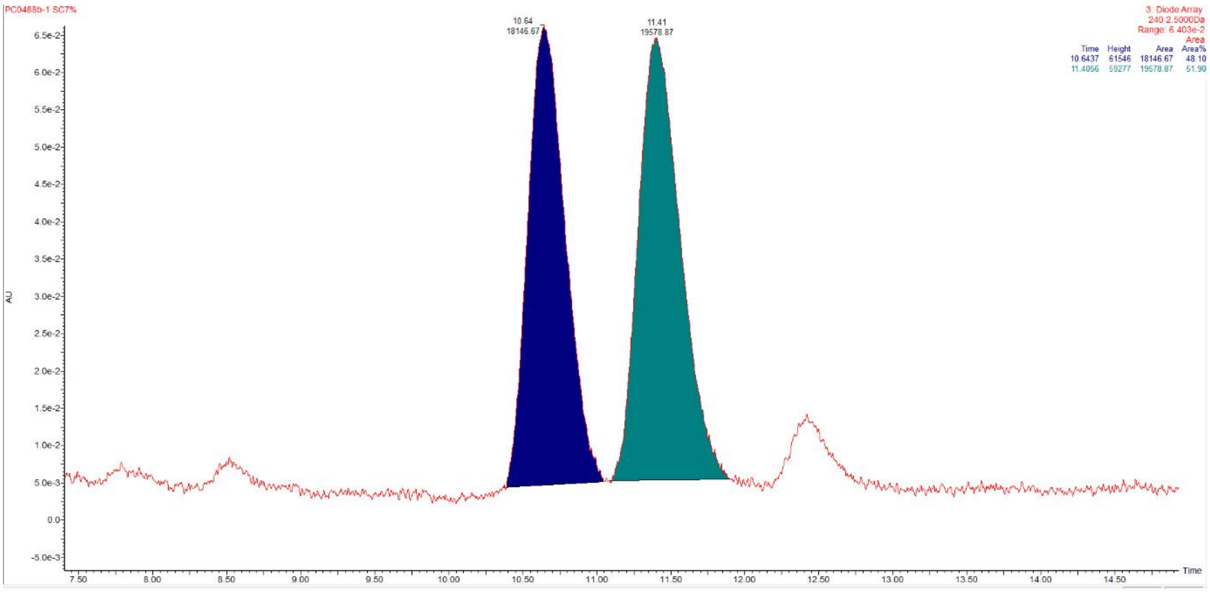

methyl (S)-6-(1-acetamido-2-phenylethyl)-5-methylnicotinate (26)

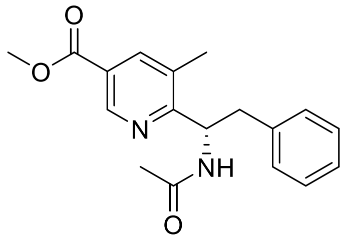

Chiralpak SC (CO_2/MeOH = 93/7, 2.5 mL min^{-1}, 40 °C, 220 nm)

73% ee

t_R = 9.3 minutes (minor), 10.2 minutes (major)

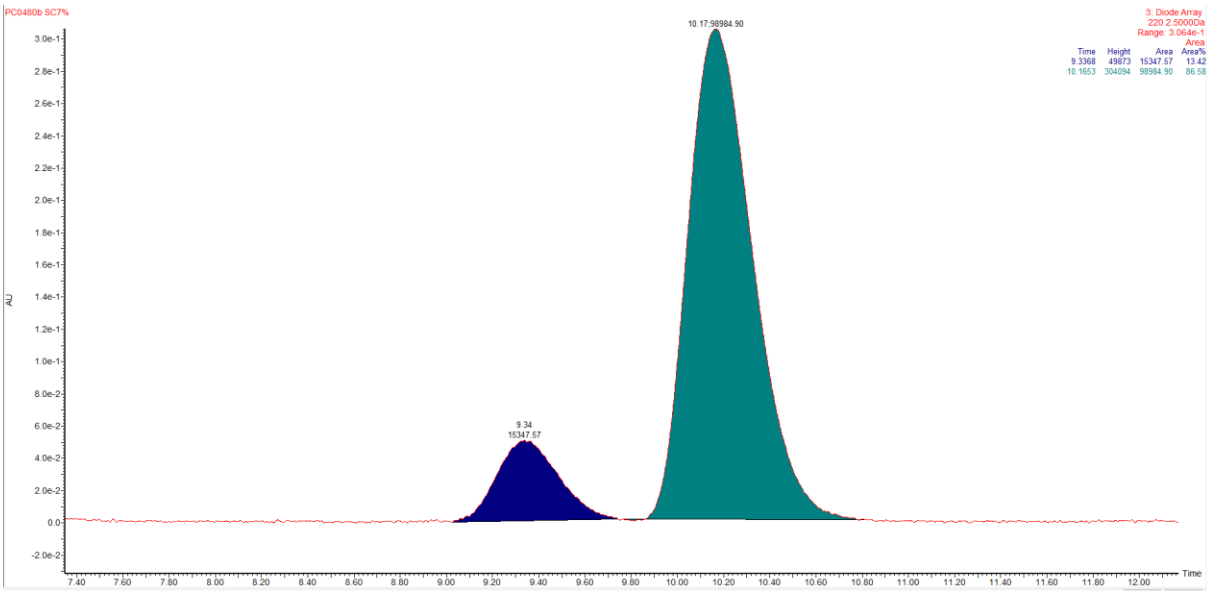

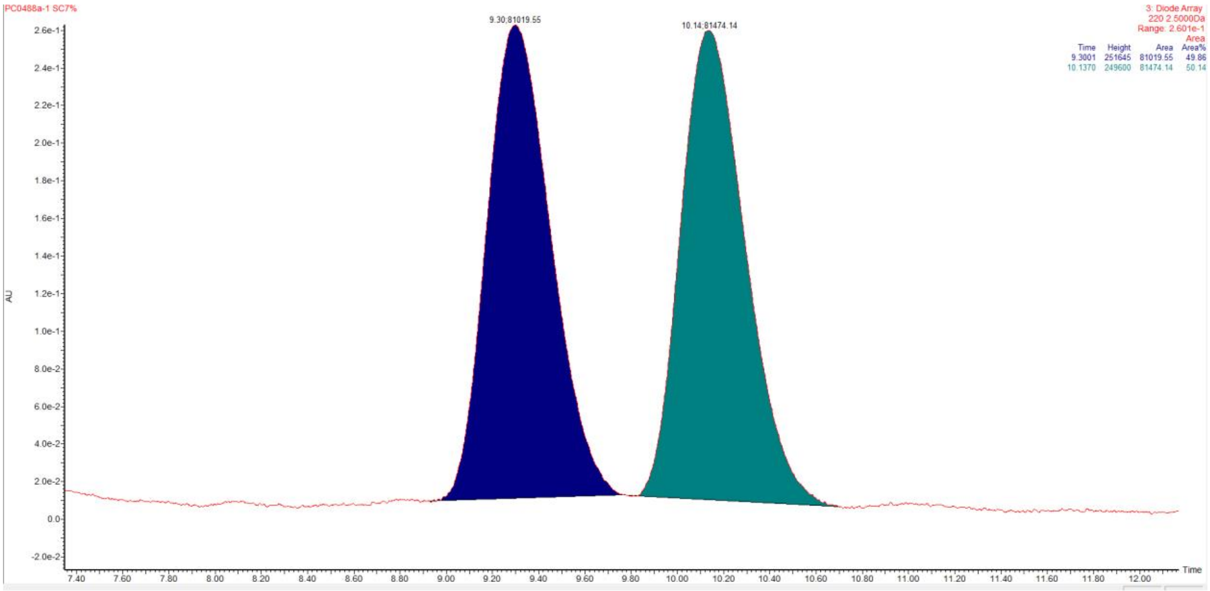

(S)-*N*-(2-phenyl-1-(5-propionylpyridin-2-yl)ethyl)acetamide (27)

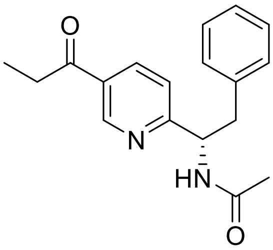

Chiralpak SC (CO_2/MeOH = 90/10, 2.5 mL min^{-1}, 40 °C, 240 nm)

<u>87% ee</u>

t_R = 9.9 minutes (minor), 10.8 minutes (major)

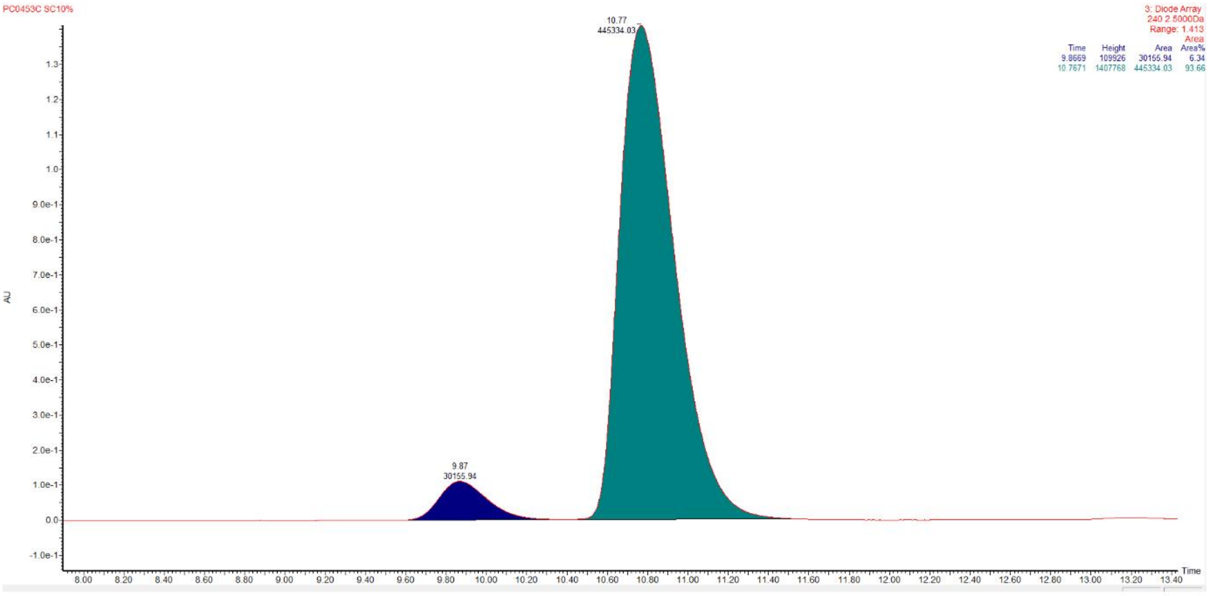

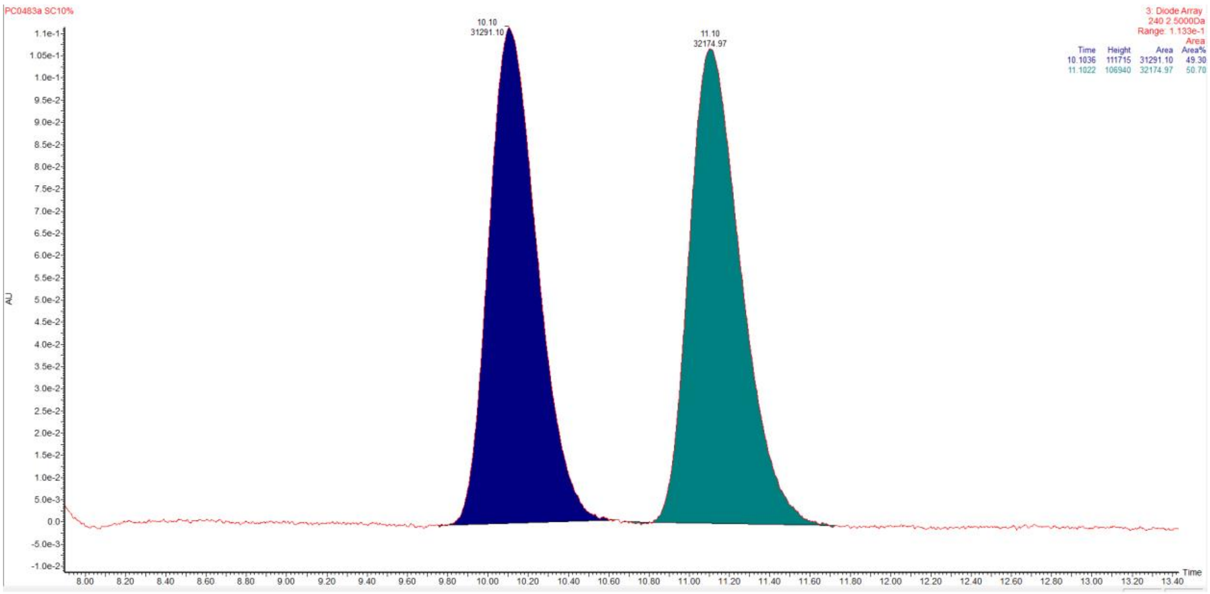

(S)-N-(1-(5-cyano-6-methylpyridin-2-yl)-2-phenylethyl)acetamide (28)

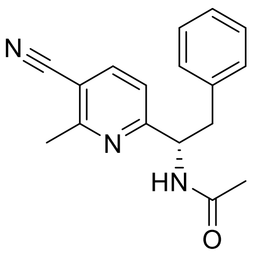

Chiralpak SC (CO_2/MeOH = 90/10, 2.5 mL min^{-1}, 40 °C, 240 nm)

92% ee

t_R = 6.9 minutes (minor), 7.6 minutes (major)

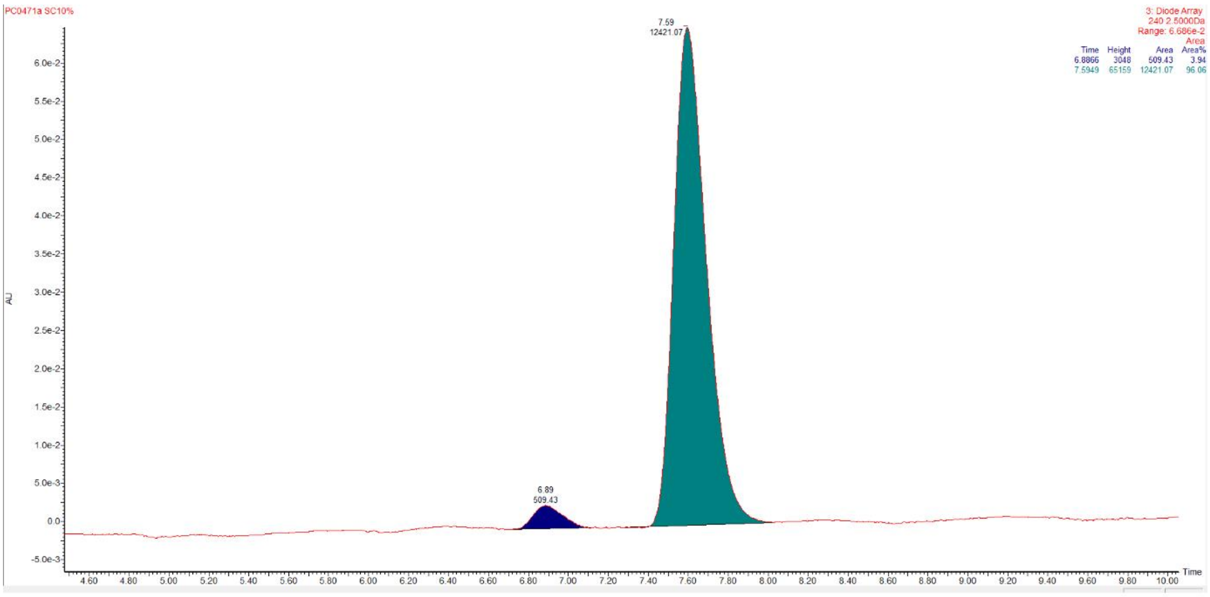

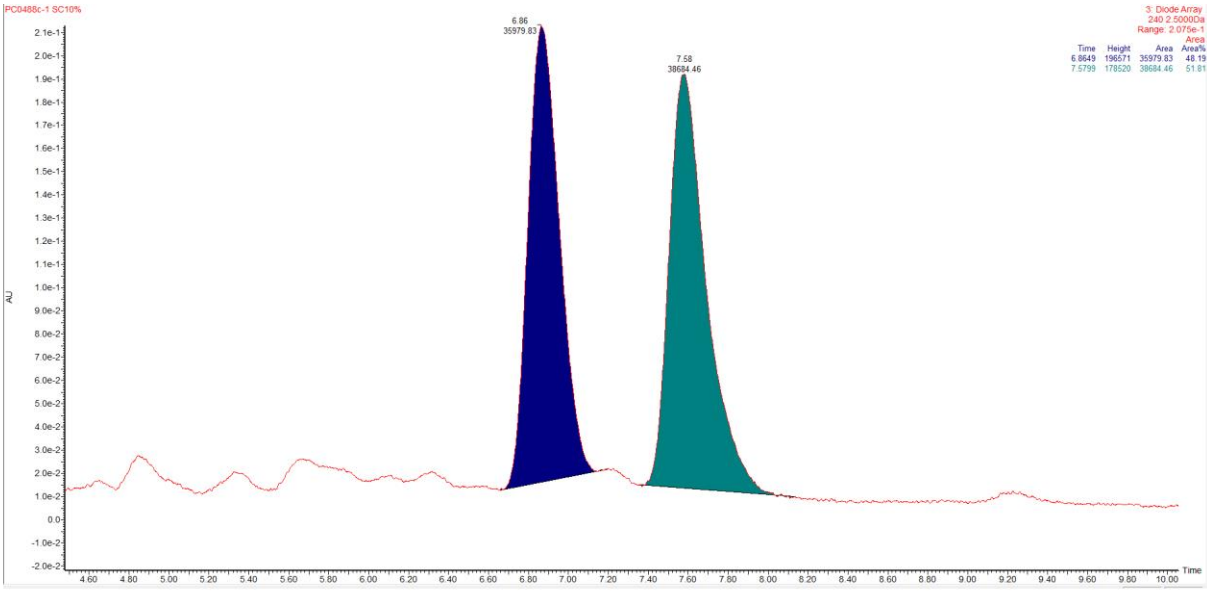

(*S*)-*N*-(1-(2-methyl-6-phenylpyrimidin-4-yl)-2-phenylethyl)acetamide (29)

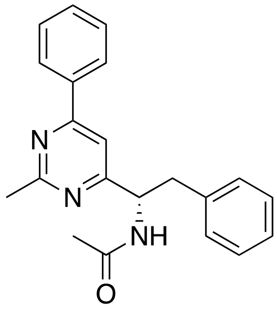

Chiralpak SC (CO_2/MeOH = 90/10, 2.5 mL min^{-1}, 40 °C, 275 nm)

95% ee

t_R = 8.1 minutes (minor), 8.9 minutes (major)

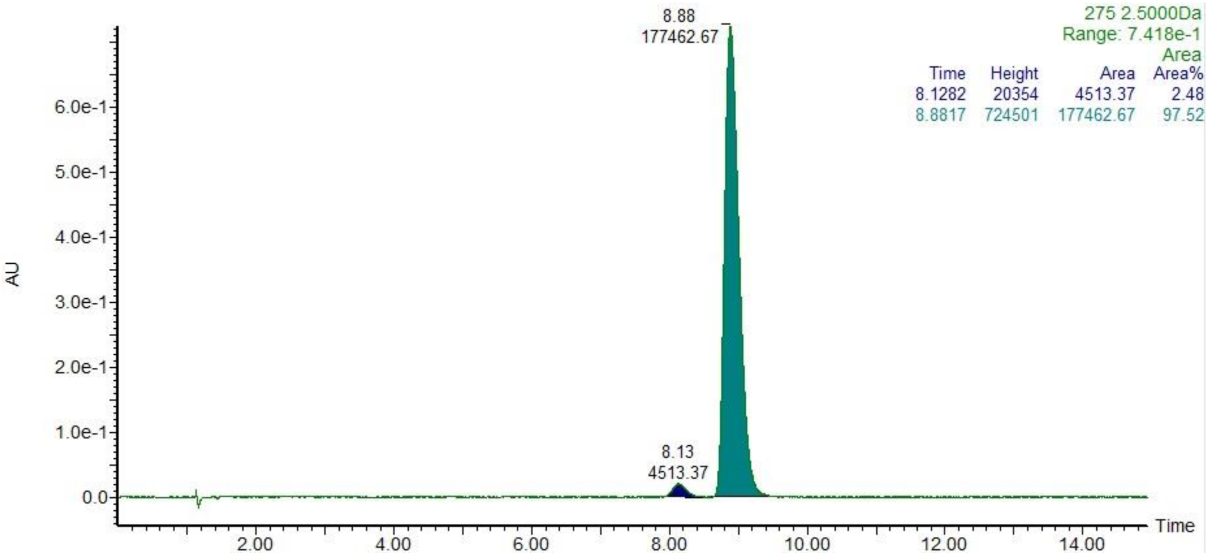

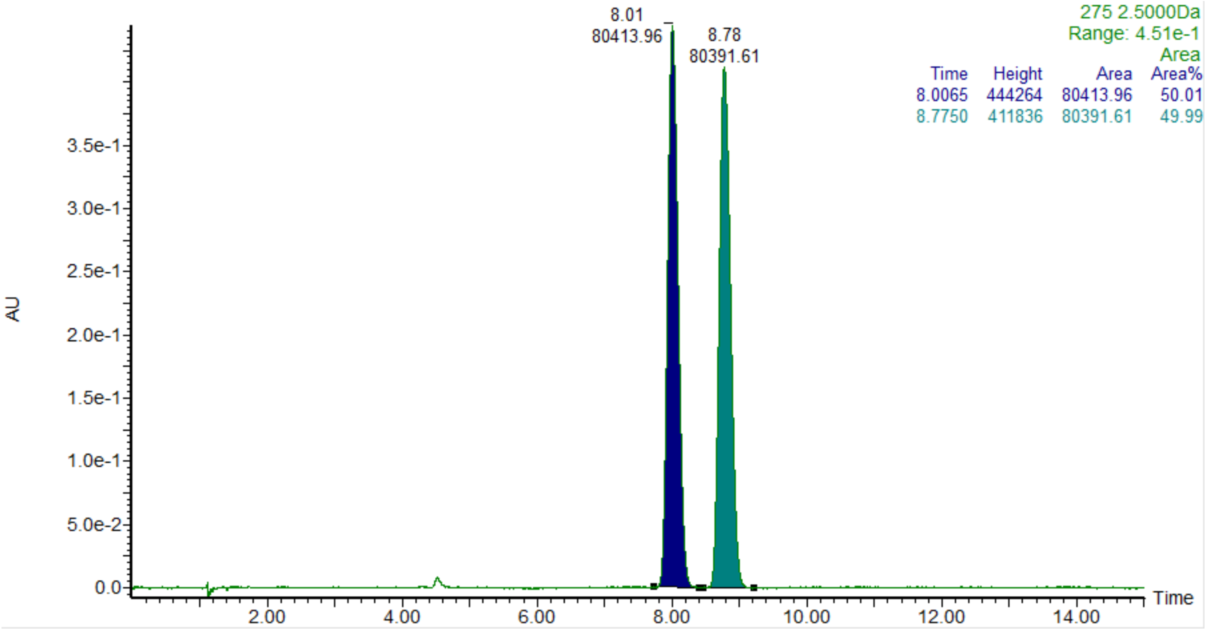

1 mmol scale: (*S*)-*N*-(1-(4-methylquinolin-2-yl)-2-phenylethyl)acetamide (3)

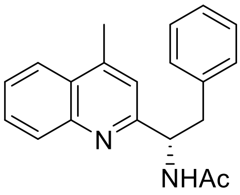

SFC Conditions: Chiralpak IC (CO_2/MeOH = 85/15, 2.5 mL min^{-1}, 40 °C, 224 nm)

<u>95% ee</u>

t_R = 5.8 (minor), 6.6 (major) minutes

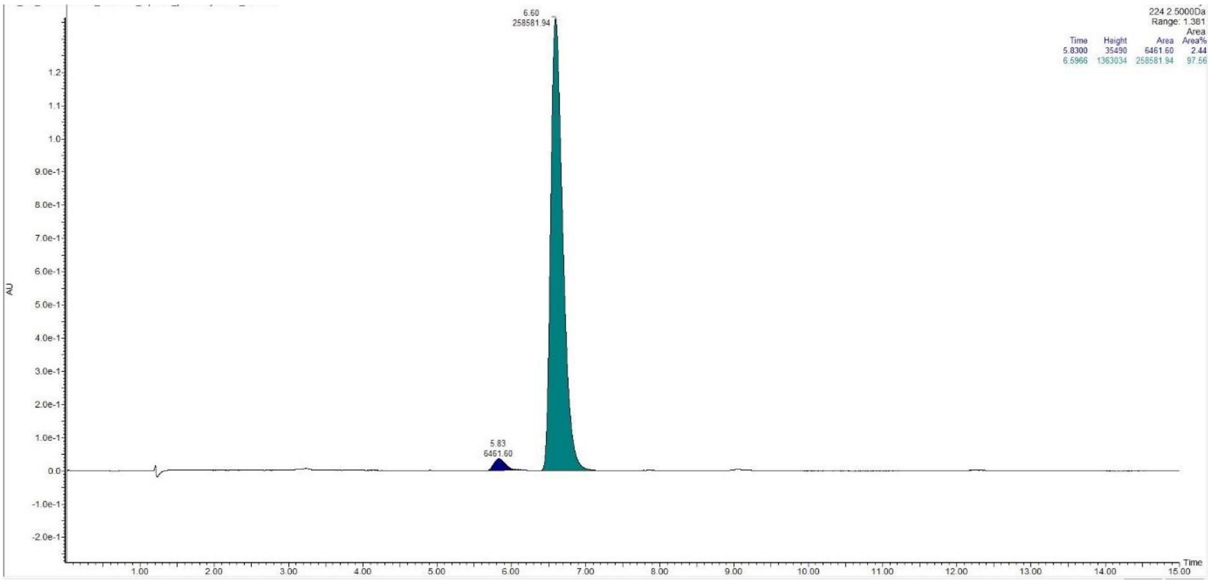

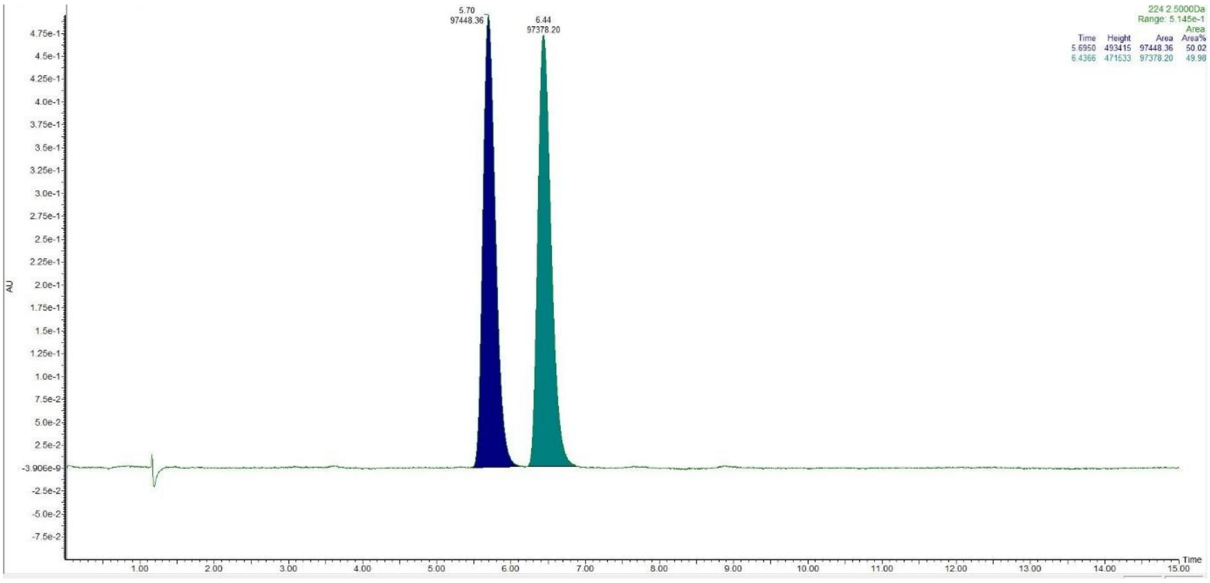

(S)-N-(1-(2-methyl-6-phenylpyrimidin-4-yl)-2-phenylethyl)acetamide (30)

Chiralpak SC (CO_2/MeOH = 95/05, 2.5 mL min^{-1}, 40 °C, 275 nm)

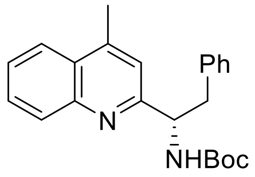

95% ee

t_R = 9.5 minutes (minor), 10.8 minutes (major)

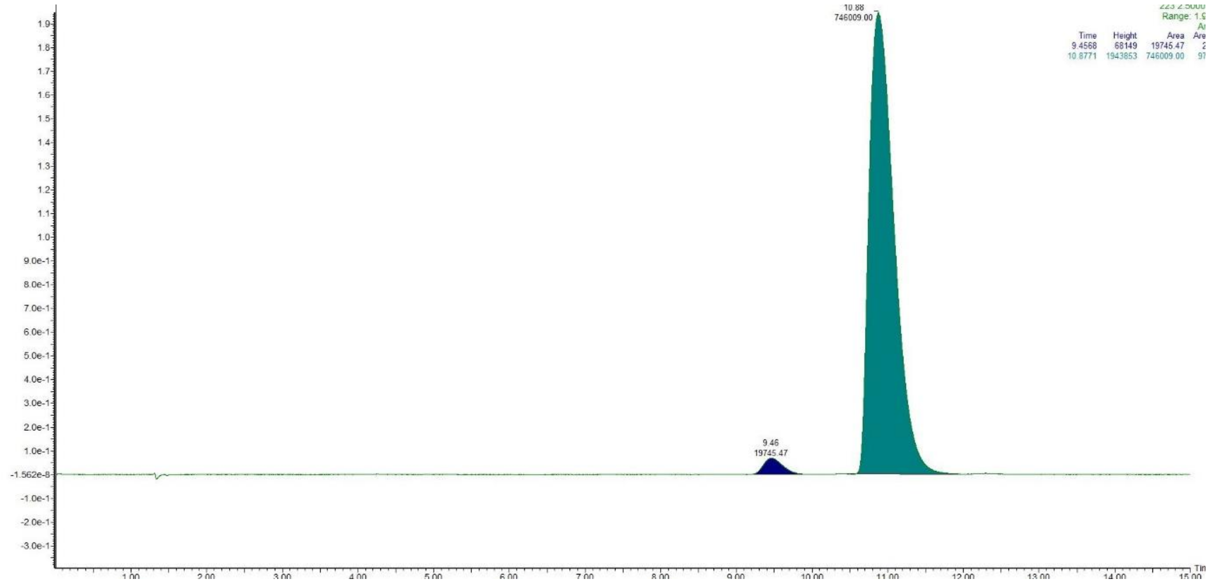

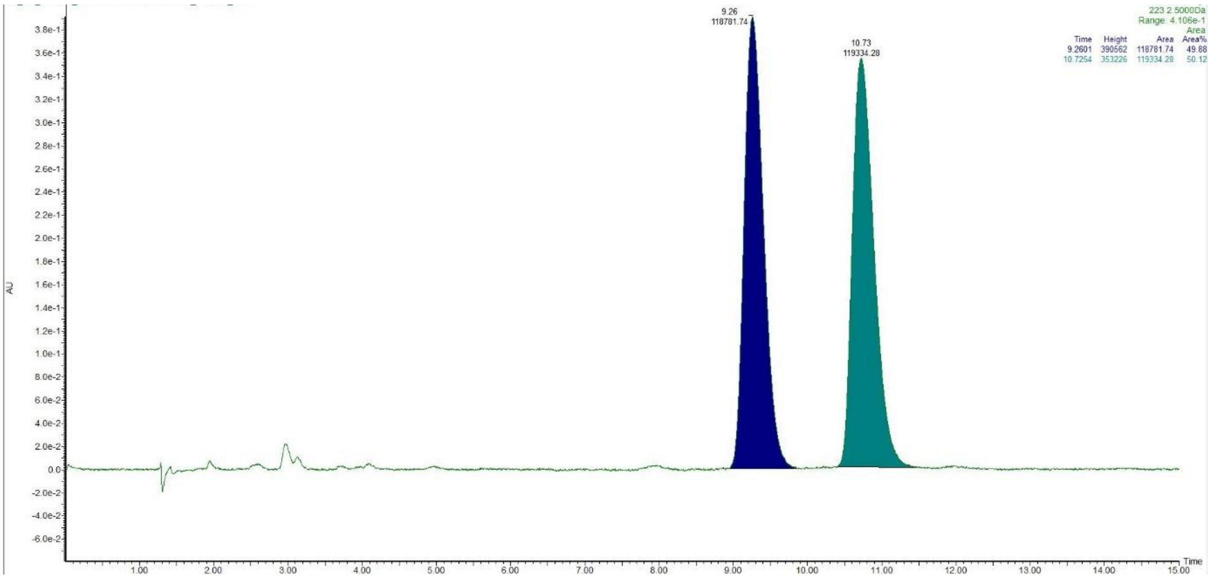

References

(1) Klussmann, M.; Ratjen, L.; Hoffmann, S.; Wakchaure, V.; Goddard, R.; List, B. *Synlett* **2010**, *2010*, 2189–2192.

(2) Rauniyar, V.; Wang, Z. J.; Burks, H. E.; Toste, F. D. *J. Am. Chem. Soc.* **2011**, *133*, 8486–8489.

(3) Mandai, H.; Murota, K.; Mitsudo, K.; Suga, S. *Org. Lett.* **2012**, *14*, 3486–3489.

(4) Gribkov, D. V; Hultzsch, K. C.; Hampel, F. *Chem. Eur. J.* **2003**, *9*, 4796–4810.

(5) Singh, A.; Teegardin, K.; Kelly, M.; Prasad, K. S.; Krishnan, S.; Weaver, J. D. *J. Organomet. Chem.* **2015**, *776*, 51–59.

(6) Szostak, M.; Sautier, B.; Spain, M.; Procter, D. J. *Org. Lett.* **2014**, *16*, 1092–1095.

(7) Proctor, R. S. J.; Davis, H. J.; Phipps, R. J. *Science* **2018**, *360*, 419–422.

(8) Reid, J. P.; Proctor, R. S. J.; Sigman, M. S.; Phipps, R. J. *J. Am. Chem. Soc.* **2019**, *141*, 19178–19185.
